# Multimodal single-cell profiling identifies nclTECs and links chromatin remodeling to TEC differentiation and self-antigen expression modes

**DOI:** 10.1016/j.isci.2026.116916

**Published:** 2026-07-28

**Authors:** Hanne Sagsveen Hjorthaug, Marte Heimli, Siri Tennebø Flåm, Benedicte Alexandra Lie

**Affiliations:** 1 Department of Medical Genetics, Oslo University Hospital and University of Oslo, Oslo, Norway

**Keywords:** thymic epithelium differentiation, chromatin remodeling, TRA expression, NEURL2, nclTEC, thymocyte maturation

## Abstract

The thymic epithelium consists of a diverse set of cells that generate tissue-restricted antigens (TRAs) for presentation to developing thymocytes, ensuring selection of functional and self-tolerant T cell receptors. Combining single-cell multimodal profiling and immunofluorescence microscopy, we characterize the cellular heterogeneity and chromatin accessibility landscape of human pediatric thymic epithelial cells (TECs) and infer regulatory programs underlying their differentiation and TRA expression. We describe a NEURL2^+^ cTEC-like population (nclTECs), which we suggest facilitates thymocyte maturation and comprises both progenitors and thymic nurse cells. We also report two mTEC(III) populations, exhibiting different localization and morphological features. Further, we reveal substantial changes in chromatin accessibility along predicted TEC differentiation trajectories and propose a model in which distinct chromatin landscapes reflect regulatory mechanisms controlling TRA expression. Our results contribute to a framework for understanding TEC differentiation and function in immune disease and cancer and may guide thymus-based therapeutic strategies.

## Highlights

• Distinct chromatin accessibility landscapes accompany TEC differentiation
• Distal enhancers open along mTEC(III) maturation and close toward mimetic mTECs
• Enhancer accessibility divergence suggests distinct self-antigen expression modes
• NEURL2+ TECs are predicted to include progenitors and thymocyte-supporting nurse cells

## Graphical abstract

**Figure:**
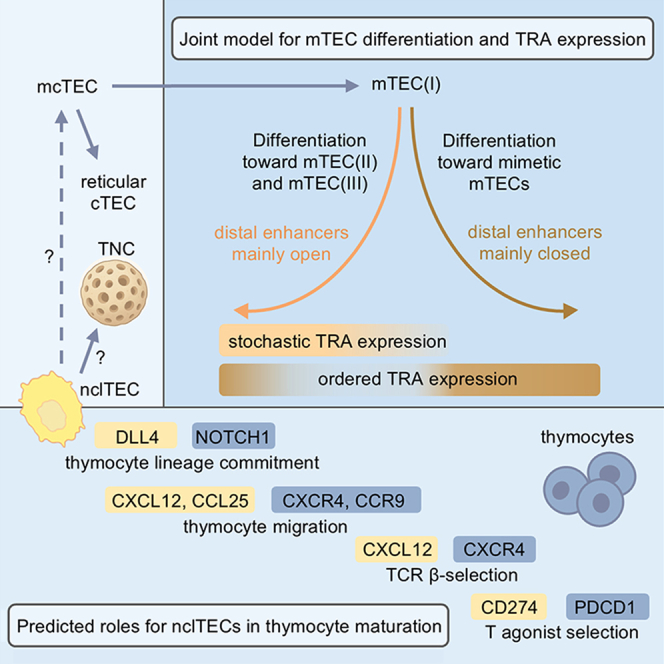

## Teaser

Immunology; Biological sciences

## Introduction

The thymic epithelium is unique in its architecture and function, holding networks of reticular cortical and medullary epithelial cells (cortical thymic epithelial cells [cTECs] and medullary thymic epithelial cells [mTECs]) that present endogenous peptides to developing thymocytes. The thymus is organized in lobules, separated from each other by the perivascular space (PVS). The PVS is lined by a single layer of squamous epithelium and extends into the corticomedullary junction (CMJ) of each lobule, where blood vessels mediate the entry of thymocyte progenitors and the exit of mature thymocytes from the thymus.^1^^,^^2^ Thymic epithelial cells (TECs) release chemokines to guide developing thymocytes through the cortical and medullary regions,^1^ and the differentiation of both TECs and thymocytes depends on thymic crosstalk, i.e., bidirectional signaling essential for both TEC and thymocyte differentiation.^3^^,^^4^

In addition to reticular cTECs, the cortical epithelium contains cage-like thymic nurse cells (TNCs), each enclosing many thymocytes that are in contact with the TNC’s outer membrane.^5^^,^^6^ TNCs are reported to be important for secondary T cell receptor (TCR) rearrangement,^7^ and together with reticular cTECs, they ensure that mature thymocytes have a functional TCR (positive selection).^8^^,^^9^ In the medulla, thymocytes are presented to tissue-restricted antigens (TRAs), and self-reactive thymocytes are eliminated (negative selection).^9^ The medulla contains additional mTECs organized in keratinized structures called Hassall’s corpuscles (HCs), suggested to mirror epidermal differentiation, as well as “mimetic” TECs resembling various peripheral cell types.^2^^,^^10^^,^^11^^,^^12^^,^^13^^,^^14^^,^^15^^,^^16^^,^^17^ Both HCs and mimetic mTECs are believed to produce TRAs,^18^ thereby adding to those produced by AIRE^+^ reticular mTECs.^19^ Over the years, reports have argued for both stochastic and ordered/coordinated expression of TRAs, or for a combination of the two.^20^ Lately, single-cell sequencing studies of murine thymus have assigned stochastic TRA expression to Aire^+^ TECs and coordinated, transcription factor (TF)-driven expression to mimetic mTECs.^11^^,^^15^^,^^16^^,^^20^^,^^21^

In contrast to the rapidly expanding fetal TEC compartment, the postnatal compartment transitions from proliferation and tissue growth to involution, starting in early life and accelerating during adolescence.^22^^,^^23^ The thymus can produce new TECs in response to damage,^24^^,^^25^ but this capacity and its functional outcome decline with age.^26^ It is not settled whether new human TECs originate from long-lived stem cells with the ability to self-renew and to give rise to all TECs, and/or from progenitor populations with restricted lineage potential and a limited proliferative capacity.^27^^,^^28^ Ragazzini et al. have identified putative postnatal stem cells within the PolyKRT TEC population, localized to the subcapsular and perivascular regions.^29^ Transcriptionally, PolyKRT TECs correspond to the mcTEC and mTEC(I) populations reported by Park et al.,^13^ with mcTEC suggested as precursors of both cTECs and mTECs. According to Yayon et al., pediatric mcTECs are skewed toward a cTEC fate in capsular regions and toward an mTEC fate in CMJs, with the proportion of mcTECs in CMJs being higher in pediatric than in fetal thymi.^30^

In the murine thymus, bipotent TEC progenitors seem to be present throughout life.^31^^,^^32^^,^^33^^,^^34^^,^^35^^,^^36^ Particularly, two distinct progenitors arise during gestation, with a cTEC-biased progenitor being most abundant prenatally, while an mTEC-biased progenitor dominates postnatally and into adulthood.^36^ The latter is suggested to develop from the cTEC-biased progenitor,^36^ supported by experiments showing that nearly all mTECs differentiate from a lineage that has once expressed the cTEC marker β5t (encoded by *Psmb11*).^37^^,^^38^ Importantly, differences between human and murine stem cell and progenitor compartments have been reported^39^^,^^40^^,^^41^; therefore, studies of human TECs are highly warranted. An example of such divergence is found in esophageal and oral squamous epithelia, where proliferation is largely restricted to the basal layer in mice, but locates to the para- and suprabasal layers in humans.^41^^,^^42^

In both murine and human epidermis, the suprabasal layer contains post-mitotic keratinocytes, differentiating toward keratinized corneocytes.^43^ Similarly, HCs are suggested to differentiate from post-mitotic AIRE^+^ mTECs.^44^ In contrast to the well-established trajectory toward HCs (mcTEC to mTEC(I), mTEC(II), and HC-associated mTEC(III)^11^^,^^12^^,^^30^^,^^44^^,^^45^^,^^46^), mimetic TEC trajectories remain elusive. The differentiation of mimetic mTECs depends on lineage-determining factors known from their peripheral counterparts^11^^,^^12^^,^^15^^,^^16^^,^^47^^,^^48^ and is expected to involve changes in chromatin architecture, as generally observed in cell differentiation.^49^^,^^50^^,^^51^ Such changes often occur within megabase-sized topologically associated domains (TADs), chromatin loops characterized by frequent internal interactions and insulation by the CCCTC-binding factor (CTCF) at their boundaries.^52^ Chromatin loops are also present at the sub-TAD level, e.g., to facilitate gene regulation by super-enhancers (SEs), which are consecutive active enhancers driving the transcription of genes involved in cell identity and lineage determination.^53^ Aire is shown to induce chromatin remodeling at SEs, suggesting their involvement in stochastic TRA expression.^54^^,^^55^ Further, Brg1 has been proposed to increase accessibility at distal enhancer-like regions targeted by Aire.^56^ Besides these reports, which focus on Aire^+^ mTECs, studies linking the chromatin accessibility landscape to TRA expression remain scarce, particularly in human TECs.

The present work aimed to characterize the chromatin accessibility landscape and cellular heterogeneity in the human pediatric thymic epithelium to gain insight into TEC differentiation trajectories and TRA expression. By using multimodal single-cell sequencing and immunofluorescence (IF) microscopy, we distinguish three mcTEC clusters with different chromatin profiles and characterize a population of NEURL2^+^ cTEC-like TECs, suggested to interact with thymocytes and to include progenitors and TNCs. We also describe two different populations in each of TEC(myo), TEC(neuro), and mTEC(III). Finally, our analyses support a model in which TRA expression is coupled to dynamic chromatin remodeling during TEC differentiation, with stochastic enhancer-driven expression emerging along the trajectory toward mTEC(III) and coordinated transcription factor-mediated programs becoming particularly evident in mTEC(III) and mimetic mTECs.

## Results

### scATAC-seq reveals distinct chromatin landscapes across TEC populations

**Figure 1:**
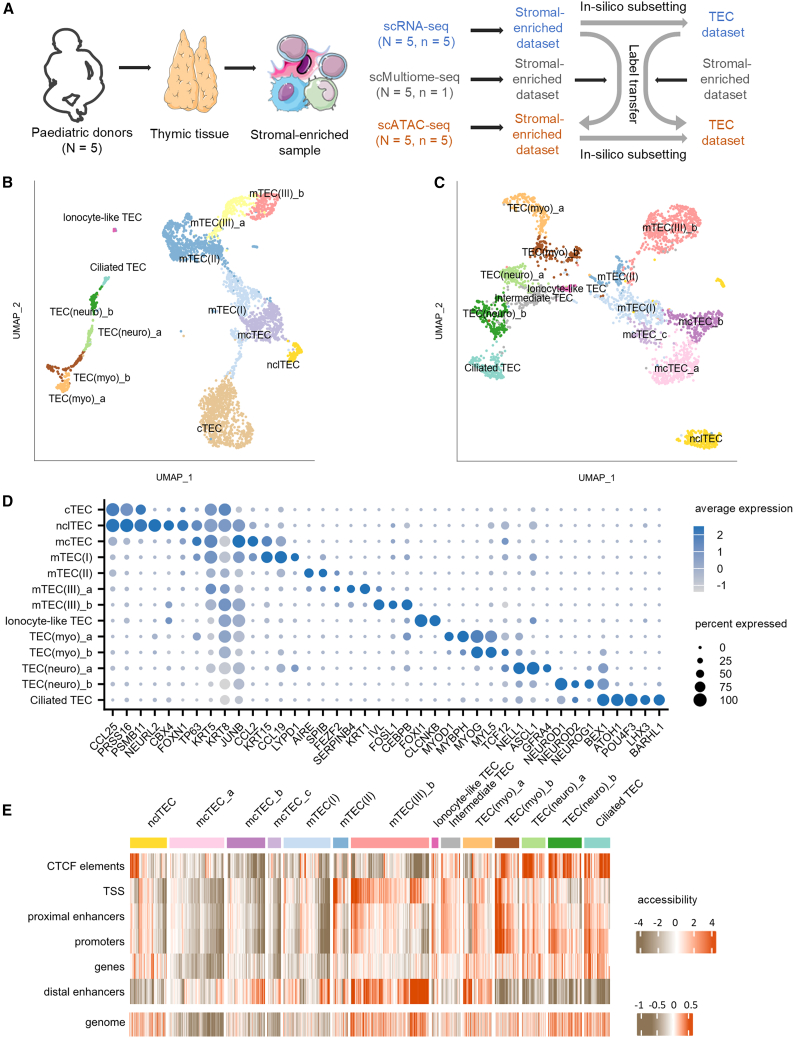
Multimodal single-cell profiling of pediatric TECs (A) Overview of the experimental setup, generating scRNA-seq and scATAC-seq data from stromal-enriched cells, from which TEC datasets were subsetted. An scMultiome-seq library was prepared from a pool of all donors and served as a bridge for transferring cell type labels. *N* = number of donors, *n* = number of libraries. Figure created with Servier Medical Art. See also Tables S1 and S2. (B) Annotated cell populations in the scRNA-seq TEC dataset. See also Figures S1 and S2. (C) Annotated cell populations in the scATAC-seq TEC dataset. See also Figures S1–S3. (D) Expression of selected markers across TEC populations in the scRNA-seq TEC dataset. See also Figure S5. (E) Genome-wide chromatin accessibilities across TEC populations at the single-cell level. Chromatin accessibility in annotated genomic regions was assessed using chromVAR and given as deviations *Z* scores. Higher scores correspond to increased accessibility, and lower scores correspond to decreased accessibility, as compared to all other cells. See also Figure S4.

In the present study, scATAC-seq data were explored together with our previously published single-cell RNA sequencing (scRNA-seq) TEC dataset,^57^ which was re-annotated at increased resolution (Figures 1A–1D, S1A–S1C, and S2; Tables S1 and S2). Cell type labels were transferred using a scMultiome-seq reference dataset and bridge integration^58^ (Figure S3). NEURL2^+^ cTEC-like TECs were redefined as “nclTECs”, and mTEC(III) populations were addressed separately from mimetic mTECs.

Most scRNA-seq-defined cell types were well recovered by scATAC-seq and scMultiome-seq, both performed on cryopreserved cells. However, cTEC and mTEC(III)_a were not recovered, and there was a notable reduction in mTEC(II) (Figures 1B vs. 1C). These cell types presented with low mitochondrial content in the scRNA-seq dataset (obtained from freshly isolated cells) (Figure S1A), a feature they shared with corresponding cells from other single-cell studies on pediatric thymi (Figure S1D). With some TECs lost, other populations were more prominent as compared to the scRNA-seq TEC dataset, e.g., Ionocyte-like TECs, which also seemed to comprise Tuft-like TECs (Figure S1E). In addition, an ATAC-specific cluster was revealed, which we termed Intermediate TEC. This cluster was predicted to contain mcTECs, mTEC(I), TEC(neuro)_a, and TEC(neuro)_b, but with relatively low prediction scores (Figure S1B). Differential peak accessibility analysis implied that this cluster was defined by common regions of relatively closed chromatin rather than by shared accessibility in cluster-specific regions (Table S3). Peaks missing or being less accessible in Intermediate TECs were associated with processes such as epithelial/epidermal cell development and differentiation (Table S4). Taken together, Intermediate TECs could represent cells in transition between early and late TECs.

When assessed at the genome-wide level, chromatin conformation was more open in mTEC(III)_b and most mimetic mTECs compared to less mature mTECs (Figure 1E). This observation contrasted with the expected restriction of chromatin accessibility as cells differentiate^59^^,^^60^ and could be related to the regulation and expression of TRA genes, supported by open distal enhancers and transcription start sites in mTEC(III)_b and accessible promoters and proximal enhancers in mimetics. mTEC(III)_b presented with low accessibility over CTCF elements and was particularly open over distal enhancers. While TEC(myo)_a resembled mTEC(III)_b in this regard, other mimetics had the opposite pattern. mcTEC and mTEC(I) were relatively closed genome-wide, but distal enhancers were partly accessible. Given the suggested role for SEs in Aire-driven TRA expression,^54^^,^^55^ we hypothesized that SEs could contribute to the observed accessibility over distal enhancers. To this end, we assessed chromatin accessibility over SEs reported from peripheral tissues (Table S5). This revealed accessibility over SEs from nearly all tissues examined for conventional mTECs and mTEC(III)_b (Figure S4). SEs were relatively closed in TEC(myo)_b, TEC(neuro)s, and Ciliated TECs, except for a few SE categories related to neural tissues. TEC(myo)_a was accessible over several SEs, including SEs presumably not related to mimetic phenotypes.

**Figure 2:**
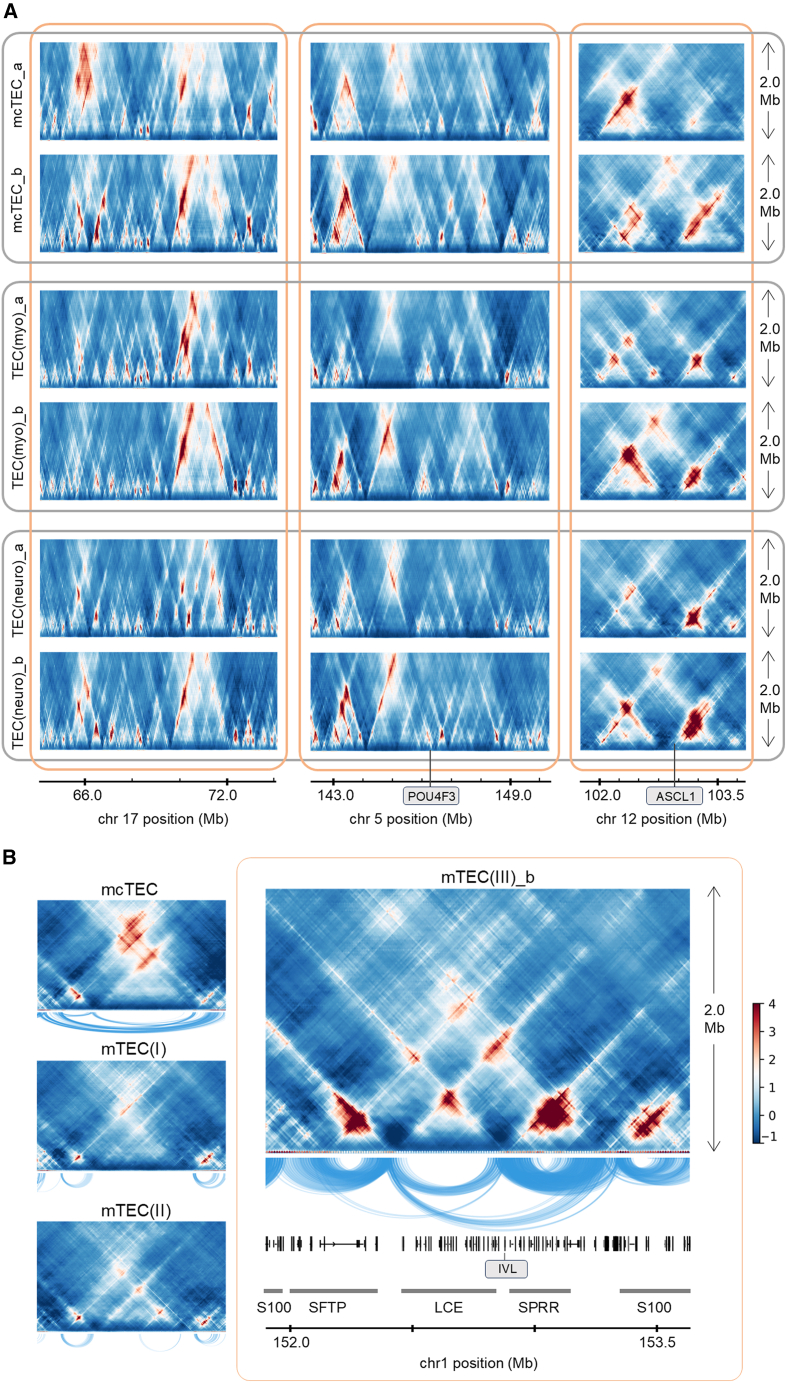
Predicted chromatin interactions Interactions are visualized by a blue-white-red color scale where dark blue corresponds to low contact frequencies and dark red to high contact frequencies. (A) Predicted chromatin contact maps for comparisons of mcTEC_a vs. mcTEC_b, TEC(myo)_a vs. TEC(myo)_b and TEC(neuro)_a vs. TEC(neuro)_b. The TADs indicated on either side of ASCL1 were present in most TEC populations, including those not shown in this figure. See also Figure S7 for additional comparisons of TEC(myo)_a vs. TEC(myo)_b and TEC(neuro)_a vs. TEC(neuro)_b. (B) Chromatin contact maps covering the EDC locus on chromosome 1, predicted for cell types along the mTEC(III) differentiation trajectory. The mcTEC map was predicted based on all mcTEC clusters (mcTEC_a, mcTEC_b, and mcTEC_c). Frequent interactions are visualized as arcs (scores above 3). The region encodes several structural and non-structural proteins expressed in the epidermis, including members of the S100, S100-fused type (SFTP), late cornified envelope (LCE), and small proline-rich (SPRR) protein families. Contact maps and arcs by ChromaFold, and gene track by 3D Genome Browser 2.0. See also Figure S6.

scATAC-seq separated mcTECs into three distinct clusters (mcTEC_a, mcTEC_b, and mcTEC_c), and chromatin contact maps were predicted for the two largest clusters. These maps revealed substantial differences in chromatin organization between mcTEC_a and mcTEC_b throughout the genome (Figure 2A), e.g., in the regions containing *POU4F3*, essential for cochlear hair cells,^61^ and *ASCL1*, a pioneering neuronal and oligodendroglial lineage-determining factor^62^ reported to be turned on during murine TEC differentiation.^14^ Also, the mcTEC clusters differed in distal enhancer (Figure 1E) and motif accessibility, e.g., with open motifs for members of activator protein 1 (AP-1) complexes (FOSL1, FOSL2, JUN, JUNB, and JUND) in mcTEC_b and open motifs for NF-κB family members in mcTEC_c (REL, RELA, and NF-κB1) (Table S6). Notably, progenitor populations are reported to possess heterogeneity not explained by gene expression,^63^^,^^64^ and we hypothesize that the mcTEC clusters represent different chromatin states destined for distinct differentiation paths.

The most significantly differentially expressed (DE) gene in the nclTEC cluster was *NEURL2* (*Ozz-E3*) (Table S7), which encodes a neuralized homology repeat (NHR) family protein^65^ known to participate in an E3-ubiquitin ligase complex.^66^ nclTECs shared several markers with mature cTECs, but they also expressed genes involved in epithelial and thymic progenitor functions, such as *TP63*,^67^ *CBX4*,^68^^,^^69^ and *FOXN1*^32^^,^^70^^,^^71^^,^^72^ (Figure 1D; Table S8). However, nclTECs did not match the expression profile of TEC progenitors reported in recent scRNA-seq studies^29^^,^^36^ (data not shown), but they did share some markers with other populations reported in such studies: murine perinatal cTECs (e.g., *Dll4* and *Neurl2*),^73^ murine TNCs (e.g., *Ly75* and *Tbata*),^74^ and human postnatal cTEC-IEGs (e.g., *CD274* and *FOXN1*)^29^ (Figure S5A). nclTECs also aligned with a subset of murine embryonic and neonatal TNCs (Foxn1^+^Tp63^+^ Krt5^+^Krt8^+^, Figure 1D) reported to reside in the CMJ^5^^,^^75^ and to share markers with progenitors in this area.^3^^,^^24^ Both cTECs and nclTECs presented with Gene Ontology (GO) terms related to antigen presentation, whereas terms concerning cell adhesion, proliferation, and stem cell differentiation were unique to nclTECs (Table S9).

**Figure 3:**
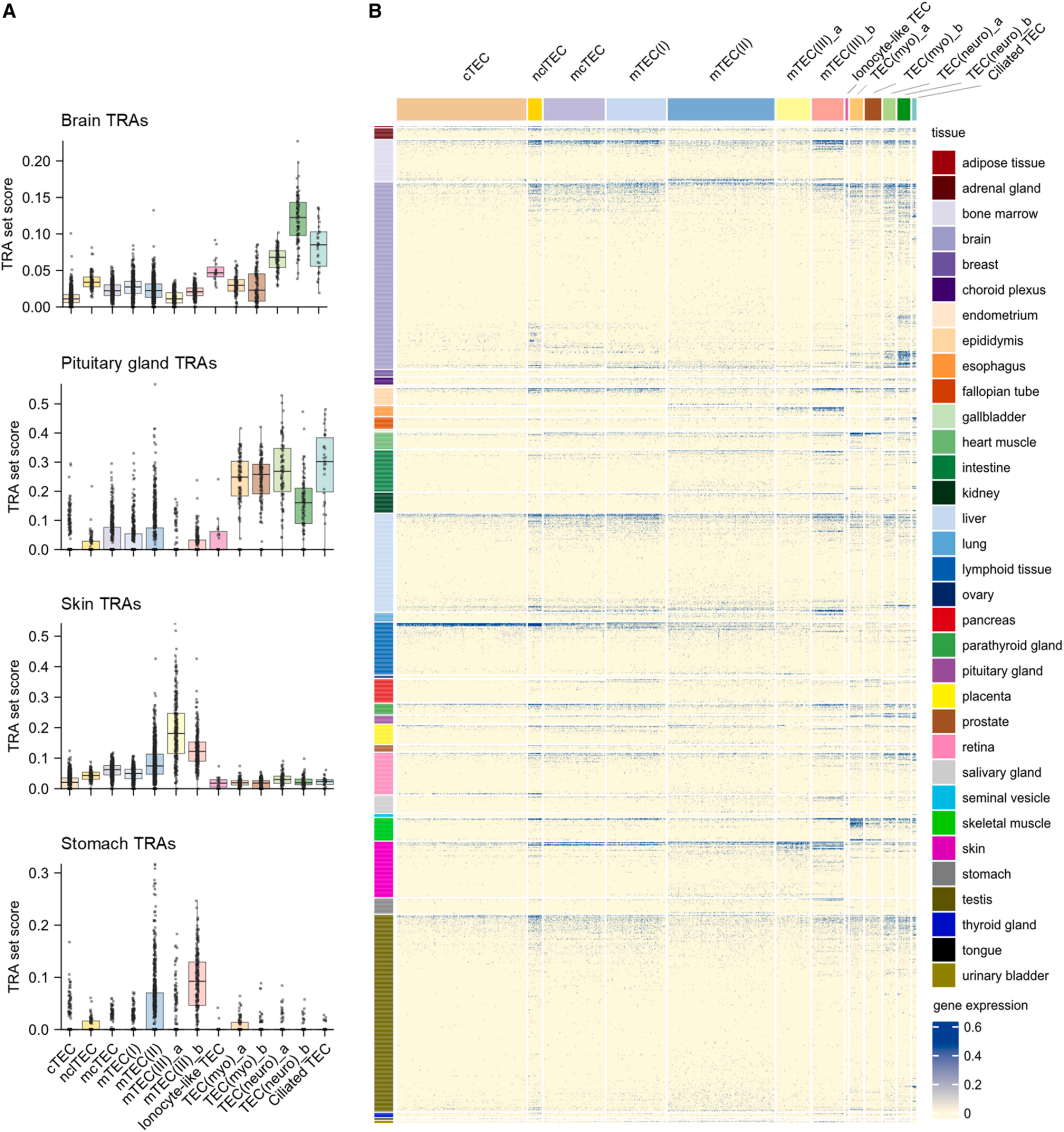
Expression of tissue-enriched genes in the pediatric thymus at the single-cell level (A) Mean expression of tissue-enriched genes assigned by the Human Protein Atlas to the brain (*n* = 360 genes), pituitary gland (*n* = 15 genes), skin (*n* = 107 genes), and stomach (*n* = 29 genes) across TEC populations. One dot is one cell. (B) Expression of tissue-enriched genes from the Human Protein Atlas across TEC populations (1,841 tissue-enriched genes expressed).

The two mTEC(III) clusters were separated by the expression of, e.g., *KRT1* in mTEC(III)_a and *IVL* in mTEC(III)_b (Figure 1D). Of all TECs, mTEC(III)_a showed the highest expression of *FEZF2* (Figure 1D), important for the development of murine Tuft-like TECs.^76^ mTEC(III)_b was among the TEC populations showing the most pronounced expression of genes involved in antigen presentation on HLA class II (Figure S5B) and presented with several GO terms concerning immune system/responses (Table S9). GO terms related to intra- and extracellular vesicles were evident in both mTEC(III)_a and mTEC(III)_b (Table S10). Furthermore, both clusters resembled skin, but mTEC(III)_b was also similar to other epithelium-rich/epithelial tissues (Table S11). Accordingly, high expression of skin TRAs was evident in mTEC(III)_a, and enrichment of stomach, esophagus, and salivary gland TRAs was observed in mTEC(III)_b (Figure 3).

The epidermal differentiation complex (EDC) locus^77^ was inferred to undergo extensive reorganization along the trajectory toward HCs (Figure 2B). The locus was constrained within a 1.5-Mb loop in mcTECs, gradually replaced by smaller loops and accumulating in extensive contacts in mTEC(III)_b. These interactions were mainly predicted within each of the four EDC gene clusters, similar to the architecture found in epidermal keratinocytes (Figure S6).

The two TEC(myo) clusters presented with genome-wide differences in chromatin architecture (Figures 2A and S7A). More chromatin contacts were inferred in TEC(myo)_a than in TEC(myo)_b for the region encompassing *MYOG* and *MYOPARR* (Figure S7A), important regulators in skeletal muscle differentiation.^78^ Further, predicted interactions upstream of *MYBPH*, a structural constituent of muscle fibers,^79^ were frequent only in TEC(myo)_a (Figure S7A).

**Figure 4:**
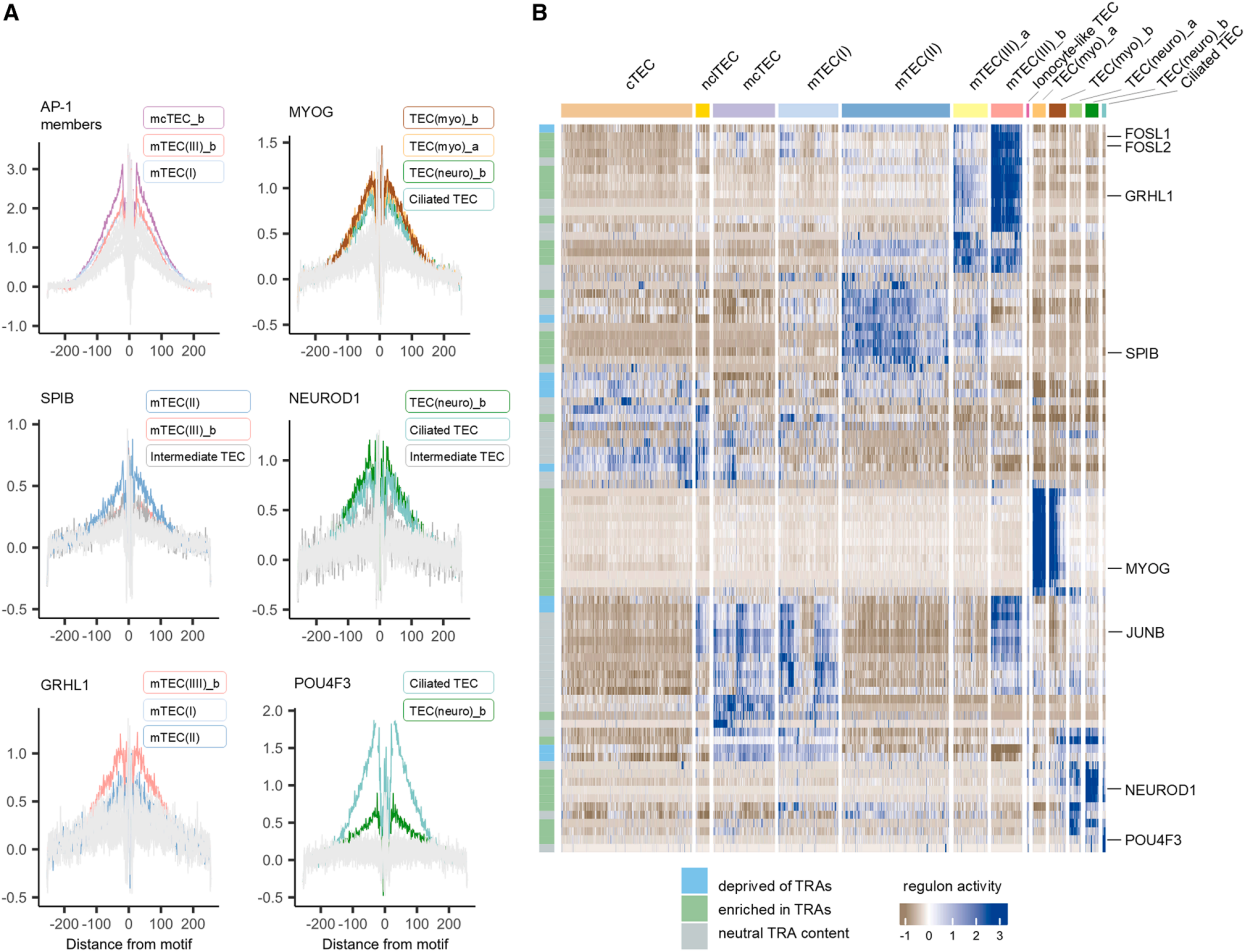
Gene expression modules (regulons) and their TF activators across TEC populations (A) Footprints of selected regulon activators. Transposase insertion enrichment is given on the *y* axis and distance from the motif on the *x* axis. Top footprints are colored by cell type. The “AP-1 members” footprint equals the JUNB footprint, which was also representative for JUN, JUND, FOS, FOSL1, and FOSL2, in line with similar motif sequences and a shared role as subunits of AP-1 complexes. See also Figure S8. (B) Regulon activity inferred by SCENIC across TEC populations at the single-cell level (details in Table S12), with regulons enriched in TRAs in green and regulons deprived of TRAs in blue (details in Table S13).

Both clusters expressed *MYOG* and skeletal muscle TRAs (Figures 1D and 3B) and presented with clear MYOG footprints (Figure 4A). TEC(myo)_a expressed *MYOD1* (Figure 1D), essential for commitment to the myogenic lineage and a prerequisite for *MYOG* expression in the periphery.^78^ Furthermore, TEC(myo)_a had the most pronounced expression of key skeletal muscle components (Table S7). Consistently, TEC(myo)_a showed a highly significant transcriptional resemblance to skeletal muscle myocytes, while TEC(myo)_b was somewhat similar to neuronal, endocrine, and skeletal muscle cells (Table S11). The few genes that were clearly more expressed in TEC(myo)_b than in TEC(myo)_a were also expressed in TEC(neuro)s and/or Ciliated TECs (Table S7), including *BEX1* (Figure 1D), an inhibitor of neuronal differentiation.^80^

Differences in chromatin architecture were also inferred between the TEC(neuro) clusters, e.g., in the regions holding *ASCL1* and the neuronal differentiation regulator *NEUROD1*^62^ (Figures 2A and S7B). In most TECs, a TAD was indicated on both sides of *ASCL1*, with a 1.5- to 2-Mb loop enclosing the TADs to varying extents (Figure 2A). This loop was infrequent in TEC(neuro)_a and mcTEC_a, suggesting that access to *ASCL1* was enhanced in these cells. While TEC(neuro)_a expressed *ASCL1*, downstream neuronal differentiation regulators (*NEUROD1*, *NEUROD2*, and *NEUROG1*)^62^ were expressed in TEC(neuro)_b (Figure 1D). Differential expression analysis between the two clusters (Table S7) revealed genes related to cell proliferation and endocrine cells for TEC(neuro)_a, and to neuronal development and neuronal cells for TEC(neuro)_b (Tables S9 and S11). Accordingly, pituitary gland TRAs showed higher expression in TEC(neuro)_a than in TEC(neuro)_b, while the opposite was true for brain TRAs (Figure 3).

### TF-driven TRA expression modules are inferred to be prominent mimetic mTECs and mTEC(III)_b

Previous studies of the murine thymus suggest that master TFs governing TEC identities also control cell-type-specific TRA expression programs,^18^ mirroring the regulation of cell-type-specific genes in the periphery.^81^ To investigate TF-driven TRA expression in the pediatric thymus, we predicted gene regulatory modules (regulons), each consisting of a TF activator and target genes (Table S12). In the resulting 117 regulons, 37% of the 8,139 target genes encoded TRAs, accounting for 35% of all TRAs expressed in our dataset.

Focusing on regulons with the highest mean activities per cell type, we observed that cell-type-specific TRA expression modules were most prominent in mimetic mTECs and mTEC(III)_b, yet also present in mTEC(II) and mTEC(III)_a (Figure 4B; Table S13). Several TRA-enriched regulons were inferred to be regulated by lineage-determining factors and other cell-type-specific TFs (e.g., GRHL1,^82^ MYOG,^78^ NEUROD,^62^ and POU4F3^61^). Generally, cell-type-specific genes are regulated by distal intronic enhancers,^83^ which may appear to contradict the low distal enhancer accessibility observed in most mimetic mTECs (Figure 1E). Notably, these scores reflect the genome-wide accessibility per cell relative to all TECs; therefore, a low score does not imply inaccessibility of all distal enhancers. Consistently, we detected distal footprints for TFs inferred to drive TRA expression in mimetic mTECs, including POU4F1 in Ciliated TECs, MYOG in TEC(myo)s, and NEUROD1 in TEC(neuro)_b (Figure S8).

The FOSL1 and FOSL2 regulons were particularly active in mTEC(III)_b (Figure 4B; Table S12 [mean regulon activities]), in line with high TF footprints for members of AP-1 complexes (Figure 4A). FOSL2 was inferred to regulate genes located in the EDC locus (*IVL*, *RPTN*, and *S100A8*),^84^ in addition to other key epidermal genes (e.g., *CLDN1*, *SERPINB2*, *SERPINB13*, and keratins)^85^^,^^86^^,^^87^ (Table S12 [regulons]). CEBPB, a regulator of inflammation,^88^ was also active in mTEC(III)_b and was predicted to induce *FOSL1* and *FOSL2* expression, consistent with reported roles for AP-1 members in inflammatory responses.^89^ The regulon inferred to be regulated by POU2F3, indispensable for the development of Tuft-like TECs,^11^^,^^12^ had its highest activity in mTEC(III)_a. SPIB, essential for the development of murine-specific microfold TECs,^15^^,^^16^ was predicted to regulate a regulon active in mTEC(II), consistent with the SPIB footprint in this cluster.

To summarize, we observed that TRA-enriched expression modules were particularly prominent in mimetic mTECs and mTEC(III)_b. Notably, several of these modules were inferred to be regulated by lineage-determining and other cell-type-specific transcription factors. Together, our results support that factors governing mTEC identities are tightly linked to the expression of TRA programs in the pediatric epithelium.

### mTEC(III) contains two populations with distinct morphology and localization

**Figure 5:**
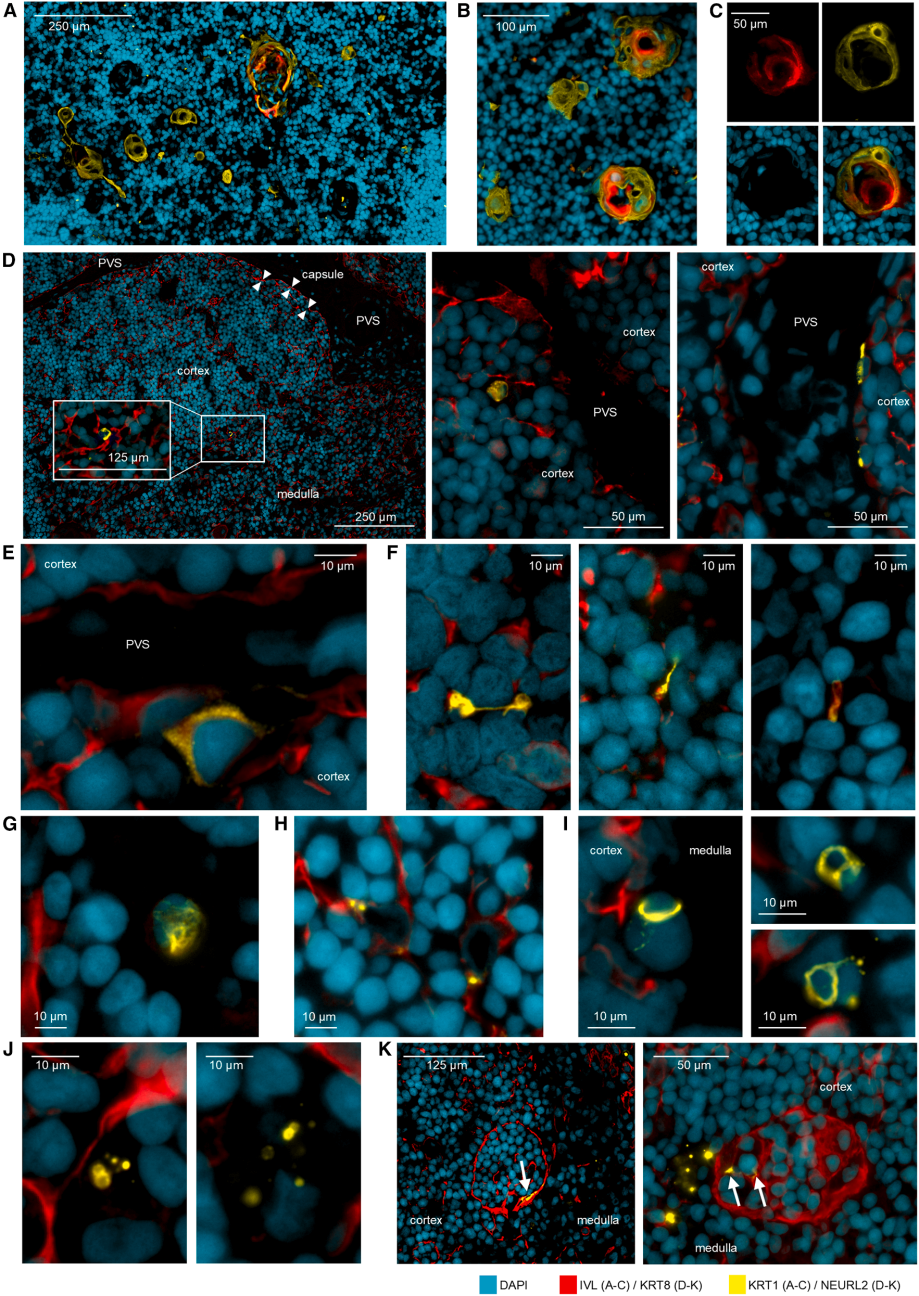
IF staining of pediatric thymi to characterize mTEC(III) and nclTEC (A–C) Localization of KRT1 (mTEC(III)_a marker) and IVL (mTEC(III)_b marker) in donors 6 (A) and 7 (B and C). (D) NEURL2^+^KRT8^+^ cells in the CMJ (left, donor 6, arrowheads indicate location of the capsule), in the subcapsular zone (middle, donor 7), and lining the PVS (right, donor 6). (E) NEURL2 signal as small spots, suggesting localization to vesicles (donor 6). (F) NEURL2 signal in narrow cytoplasmic connections, possibly indicating dividing cells. (Left) Donor 6, medulla (close to CMJ); (middle) donor 6, CMJ; (right) donor 7, CMJ. (G) NEURL2 signal locating to filament-like structures, presumably extending from, or toward, one side of the cell/nucleus (donor 7, CMJ). (H) KRT8^+^-positive cells with NEURL2 signals adjacent to a ring-shaped DNA structure (indicative of cells in prometaphase) (donor 6, CMJ). (I) NEURL2 signal as lasso-like structures (donors 6 [left and upper right] and 8 [lower right], CMJ). (J) NEURL2 signals appearing as small ring-shaped structures and dots. (Left) Donor 8, CMJ; (right) donor 7, CMJ. (K) NEURL2^+^ TNC-like structures at the cortical side of the CMJ (donor 6 [left], donor 7 [right]). Arrows point to NEURL2 signal within the structures. Scale bars: (A) 250 µm; (B) 100 µm; (C) 50 µm; (D) 250 µm (left), 125 µm (left insert), 50 µm (middle and right); (E-J): 10 µm; (K) 125 µm (left), 50 µm (right). DAPI was used as a DNA counterstain to visualize nuclei. Images are displayed using linear lookup tables. Per-channel grayscale images are available in Document S1. Control stainings and additional images are given in Figure S9. Donor ages are listed in Table S1.

mTEC(III)_a, but not mTEC(III)_b, had near-zero levels of mitochondrial transcripts (Figure S1A), which could be a consequence of cell membrane rupture during tissue dissociation. Accordingly, mTEC(III)_a was not recovered after cryopreservation. We hypothesized that the two mTEC(III) clusters have different morphology and/or localization *in situ*, which could result in divergent tolerance to dissociation. To this end, we performed IF staining of pediatric thymi using the mTEC(III)_a marker KRT1 and the mTEC(III)_b marker IVL (Figures 5A–5C and S9A). KRT1 signal was detected in HCs of varying sizes and morphologies, consistent with different maturation stages,^90^ and was also associated with nuclei elsewhere in the medulla, suggesting localization to pre-HC stages. IVL was mainly detected in the middle of large, presumably mature HCs, but also partially overlapped with KRT1 at the periphery of HCs. One could speculate if this staining pattern reflects a transition from mTEC(III)_a into mTEC(III)_b at the final stage of mTEC differentiation, agreeing with the reported expression of KRT1 in early and IVL in late epidermal development.^87^

### nclTECs appear to comprise progenitors and TNCs

Having revealed that nclTECs expressed known progenitor markers, displayed GO terms relevant to stem cells and progenitors, and shared markers with TNCs, we performed IF to determine their localization and morphology. To this end, we targeted keratin 8 (KRT8) and NEURL2, a combination restricted to nclTECs in our full thymic scRNA-seq dataset (Figure S9B).

We observed NEURL2^+^KRT8^+^ cells in subcapsular zones and in the PVS, clearly skewed toward CMJs (Figures 5D–5H, 5K, and S9H), confirming their localization to TEC progenitor niches.^25^^,^^29^^,^^30^^,^^35^ In these niches, the NEURL2 signal displayed distinct patterns that may indicate specific functions of this protein in the thymic epithelium (Table S14). Notably, some NEURL2^+^KRT8^+^ cells showed features indicative of cell division, a property generally restricted to stem cells and progenitors in healthy human epithelia.^91^ We also observed rare NEURL2^+^KRT8^+^ TNC-like structures in the cortex, near the CMJ (Figures 5K and S9H). Since TNCs have a role in positive selection,^8^ we assessed the expression of genes involved in antigen processing and presentation,^92^ revealing that nclTECs had the most pronounced expression among all TECs, particularly of HLA class II genes (Figure S5B).

**Figure 6:**
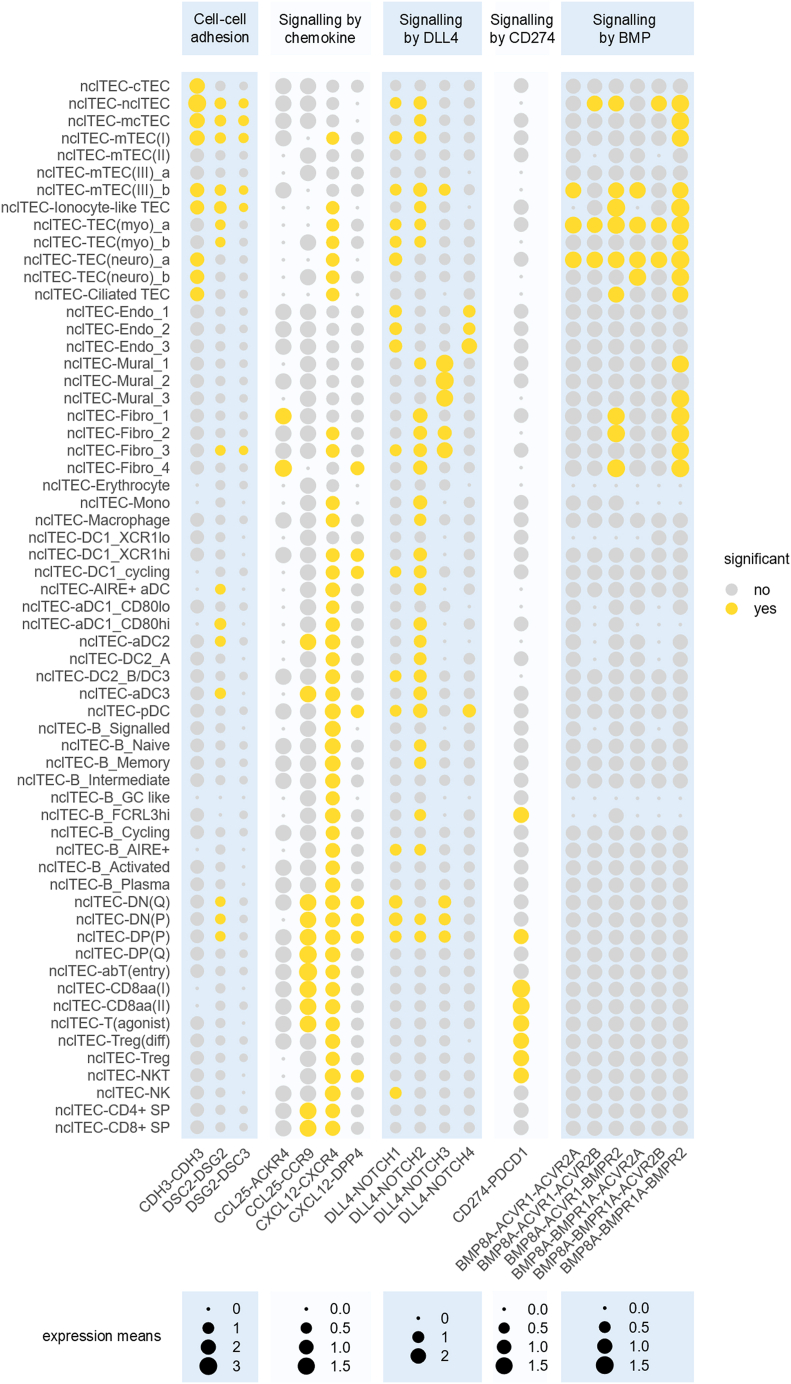
Predicted interactions between selected nclTEC ligands (or adhesion molecules) and the corresponding receptors (or adhesion molecules) on thymic cells Predictions were performed on our full thymic scRNA-seq dataset using CellPhoneDB (see Figure S10) and visualized for selected interactions for which the ligand (or adhesion molecule) showed increased expression in nclTECs. Yellow dots refer to interactions predicted with a *p* value of 0.001, while the remaining interactions were inferred as non-significant/non-existing. Dot size corresponds to scaled mean RNA expression values of interacting partners. Expression of individual ligands, adhesion molecules, and receptors is shown in Figure S11.

In contrast to cTECs, nclTECs appeared to be among the most actively communicating cell types, predicted to signal both autocrine and paracrine with several TEC and stromal populations (Figure S10). BMP8A, mainly expressed by nclTECs, was inferred to signal to fibroblasts, mural (vascular) cells, other TECs, and nclTECs themselves (Figure 6). BMPs are secreted ligands that activate SMADs upon BMP-receptor binding, with essential roles in proliferation and differentiation.^93^ Overall, nclTECs had the most pronounced expression of SMAD transcripts (Figure S11A). Further, signaling between nclTECs and thymocytes at different developmental stages was inferred, e.g., via CCL25, CXCL12, and DLL4 (Figure 6). CCL25 and CXCL12 direct thymocyte migration,^1^ with CXCL12 also implicated in TCR β-selection,^94^ while Dll4-Notch1 signaling is essential for thymocyte lineage commitment.^95^ nclTECs were also inferred to interact through CD274 (PDL1) with PDCD1 (PD-1), expressed on minor proportions of double-positive (DP) proliferating thymocytes and agonist-selected T cells in our full thymic scRNA-seq dataset (Figure S11B). This suggests that our previously inferred agonist trajectories, partly defined by PDCD1 expression and diverging before CD4^+^ or CD8^+^ lineage commitment,^57^ might be dependent on interactions with CD274^+^ nclTECs. This hypothesis fits with reported roles for PDCD1:CD274 signaling in the regulation of autoreactive T cells^96^ and in inhibition of TCR signaling in DP thymocytes,^97^ and also with our previous analysis locating thymocytes with a strong TCR signaling signature to the CMJ.^57^

**Figure 7:**
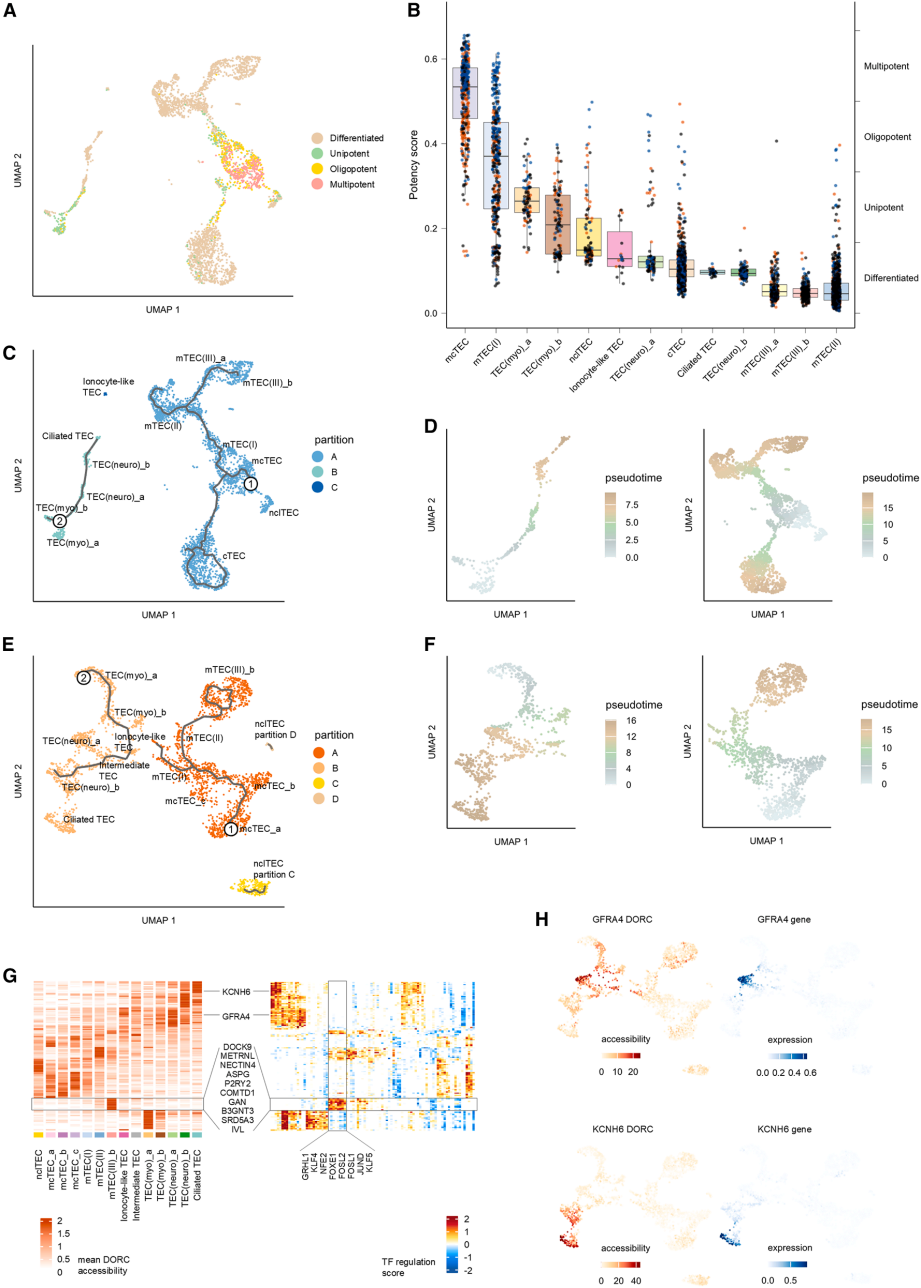
TEC differentiation in the pediatric thymus (A and B) Developmental potential (inferred by CytoTRACE 2). (A) Potency categories. See Table S15 for the proportions of cells in each CytoTRACE 2 potency category, per TEC cluster. (B) Potency scores, with scores for the youngest donor (7 days, prematurely born) in orange and for the second youngest donor (7 weeks) in blue. (C–F) Trajectory analysis (inferred by Monocle3). (C) The scRNA-seq TEC dataset was separated into three partitions, i.e., groups of cells for which trajectory graphs are learned. Mimetic mTECs, except Ionocyte-like TECs, were considered as one partition and conventional TECs (including mTEC(III) and nclTECs) as another. (D) Pseudotime values for mimetic mTECs and conventional TECs in the scRNA-seq dataset, given user-defined roots marked as 2 and 1 in (C). (E) The scATAC-seq TEC dataset was separated into four partitions. Mimetic mTECs were considered one partition and conventional TECs another. nclTEC was divided into two partitions C and D, with partition D composed mainly of cells from the prematurely born donor. (F) Pseudotime values for mimetic mTECs and conventional TECs in the scATAC-seq dataset, given user-defined roots marked as 2 and 1 in (E). (G and H) Regulatory chromatin domains (inferred by FigR). (G) Mean accessibility and inferred regulators for the top differentially accessible (DA) DORCs. Top DA DORCs (left) were defined as the union of the 10 most significantly DA DORCs for each cell type. Results from differential DORC accessibility analysis are given in Table S18. Regulators of the DORCs (Table S21) are shown in the right plot (regulators along the *x* axis and DORCs along the *y* axis). Positive regulation scores correspond to inferred activation of the DORC gene, while negative scores indicate repression. See Figure S13 for a larger version of these plots, with DORC and regulator names included. (H) For several DORCs, chromatin accessibility preceded expression of the DORC gene along our inferred differentiation trajectories, indicative of primed chromatin, as exemplified by GFRA4 and KCNH6 DORCs. See also Figure S14.

Next, we inferred developmental potential from the scRNA-seq data (Figures 7A and 7B). 11% of nclTECs were predicted as oligopotent, 25% as unipotent, and 64% as differentiated (Table S15). Despite a low number of cells (*n* = 97), the nclTEC potency categories showed differential gene expression (Tables S16 and S17). The genes that were more expressed in differentiated nclTECs included cTEC markers (e.g., *PSMB11*). Genes showing higher expression in oligopotent nclTECs were also increased in mcTECs/mTEC(I), including *KRT17*, a marker seen in tracheal epithelial progenitor cells.^98^

In short, IF stainings confirmed the heterogeneous nature of nclTECs, containing both conventional cells, of which some appeared to be dividing, and TNC-like structures. Data analyses inferred developmental potential within the nclTEC cluster and pointed to nclTECs as signaling partners in interactions previously shown to be important for thymopoiesis, including lineage commitment and positive selection. Further, we emphasize that our IF stainings reveal hitherto unrecognized expression of NEURL2 in a human epithelium, with distinct expression patterns indicative of roles in intracellular trafficking and cell division.

### TEC differentiation is accompanied by remodeling of regulatory chromatin

To predict TEC differentiation routes using our scATAC-seq and scRNA-seq datasets, we applied two different strategies. RNA velocity analysis returned results partly in conflict with established knowledge of mTEC differentiation, as well as showed disagreement between the steady state and dynamical model implemented in the package (Figure S12). Others have also reported issues with this tool,^99^^,^^100^ and we concluded that RNA velocity analysis was not suitable for our dataset. Trajectory analysis, on the other hand, with ordering of cells along pseudotime, returned the expected trajectory for the conventional mTEC lineage, from mcTEC to mTEC(I), mTEC(II), and mTEC(III)^11^^,^^12^^,^^30^^,^^44^^,^^45^^,^^46^ (Figures 7C–7F). For mimetic mTECs, a trajectory from TEC(myo) to TEC(neuro)_a, TEC(neuro)_b, and Ciliated TECs was inferred. In the RNA-based analysis, nclTECs were given pseudotime values similar to those of mcTECs, but were not included in a trajectory graph. Relying on the scATAC-seq dataset, the nclTEC cluster was considered a separate trajectory.

To infer genes and regulatory mechanisms driving TEC differentiation, we applied the concept of domains of regulatory chromatin (DORCs), an approach previously revealing lineage-determining genes in murine skin.^101^ Using computationally paired cells from our scRNA-seq and scATAC-seq TEC datasets, we identified 443 DORCs, each named after the gene they were inferred to regulate (Table S18). DORC genes were clearly skewed toward GO terms related to development and differentiation (Table S19). However, other processes were also implicated, e.g., cell migration and leukocyte activation, in line with a previous report showing involvement of DORCs upon immune cell stimulation.^102^

To pinpoint DORCs specific for each cell type, we performed differential testing of DORC accessibility scores (Figures 7G and S13A; Table S18). Cell-type-specific DORCs encompassed several DE genes, including genes known from development/differentiation of TECs and other epithelial cells, such as *FOXN1*^32^^,^^70^^,^^71^^,^^72^ and *POU4F3*^61^^,^^103^ (Figures 1D and S13A). Of note, the *FOXN1* DORC was one of several DORCs showing diverging accessibility between the mcTEC clusters. mTEC(III)_b displayed particularly high accessibility across DORCs (Table S18). While this finding might reflect increased power due to cluster size, it also supports the involvement of DORCs in processes beyond development/differentiation, given the differentiated state of mTEC(III)_b. In this regard, we noted that GO terms for the top 100 accessible mTEC(III)_b DORCs included antibacterial peptide production and glycosylation (Table S20).

Most of the TFs inferred to regulate the DORCs were predicted as either activators or repressors, while a minority, like JUNB and FOXN1, appeared to have divergent roles in different TEC populations (Figure S13B; Table S21). 57% of DORC regulators were classified as TRAs, and collectively, DORC regulators and DORC genes accounted for 4.9% of TRAs expressed by the TECs. DORC activators comprised the majority of TFs predicted to regulate TRA-rich regulons (Figure S13B vs. Table S13), supporting the notion that master regulators of TEC identity also control cell-type-specific TRA expression programs. GRHL1 was one of the factors inferred to activate both TRA expression and DORCs and had a clear footprint in mTEC(III)_b (Figure 4A). This factor, known to possess pioneering activity^104^ and to regulate key epidermal genes,^82^ was inferred to activate the *IVL* DORC, among others (Figure 7G). KLF4 and AP-1 members, reported regulators of the EDC locus,^77^ were also *IVL* DORC activators. Similarly, several DORCs specific to mimetic mTECs were predicted to be regulated by TFs known from peripheral counterparts, such as MYOG, ASCL1, NEUROD1, and POU4F3 (Figure S13). DORCs that were particularly open in nclTEC and mcTEC had TP63 among their activators. The NF-κB family members RELB and NF-κB1 were also inferred to activate nclTEC and mcTEC DORCs, yet other members of this family (REL, RELA, and NF-κB2) were more activating toward mTEC(II)-specific DORCs. The involvement of NF-κB family members is in line with their established role in mTEC differentiation.^105^^,^^106^^,^^107^

To indicate lineage priming, we identified DORCs with a large difference between DNA accessibility and RNA expression of the DORC gene (“high residual DORCs”), shown to correlate with a bivalent chromatin state.^101^ We detected 17 high-residual DORCs for which chromatin accessibility preceded DORC gene expression (Figures 7H and S14) along our inferred differentiation trajectories (Figures 7C–7F). These DORCs showed high accessibility in mimetics and mTEC(III)_b and included genes known from development/differentiation of various neuronal, glial, and sensory cells (*NKX6-2*,^108^ *SOX2*,^109^^,^^110^ *GFRA4*,^111^ *POU4F3*,^61^^,^^103^ *BARHL*,^112^ and *LHX3*^113^). Of note, LHX3 is implicated in the development of the pituitary gland, and TRAs from this tissue were highly expressed in Ciliated TECs (Figure 3).

To sum up, we inferred cell-type-specific regulation of chromatin domains across TEC populations, involving TFs and other genes described in lineage choice and differentiation. An open question is whether these genes are truly engaged in these processes or expressed solely for TRA presentation. The skewness of DORC GO terms toward development and differentiation, and the fact that mimetic mTECs phenotypically resemble their peripheral counterparts,^11^^,^^15^^,^^17^ argue for a genuine engagement; however, additional roles were also indicated. Particularly, the high accessibility across DORCs in mTEC(III)_b fits with accessibility over distal enhancers/SEs in these cells and a putative role in the regulation of TRA expression.

## Discussion

### Putative progenitors among nclTECs

Our findings suggest that nclTECs could include progenitors not previously described in the human thymus. The lack of previous reports could be due to the heterogeneous nature of this cluster and differences in sample preparation or donor age distributions. In our study, the nclTECs with the highest developmental potential were inferred in the youngest donors, implying that the most potent nclTECs might be depleted or lose potency with age. This raises the question of whether putative multipotent nclTECs could be the human counterpart of the murine cTEC-biased embryonic/early bipotent progenitor,^33^^,^^34^^,^^36^ shown to decrease in numbers and/or potency after birth.^36^^,^^37^^,^^38^ The scenario that nclTECs could be present prenatally fits with *NEURL2* expression alongside other nclTEC markers in human prenatal TECs (Figure S15). Furthermore, their bipotency is supported by markers shared with a cTEC-like murine progenitor capable of generating mTECs in reaggregate thymic organ cultures (Figure S16). Of note, these markers were found to be expressed through pre- and postnatal murine development.^114^ Furthermore, the reported reduction in both mTEC and cTEC numbers upon depletion of *Cbx4*,^115^ a marker of nclTECs, could be consistent with bipotency, but may also reflect an essential role of nclTECs in thymopoiesis. In the latter scenario, impaired nclTEC function would disrupt thymocyte maturation, leading to reduced thymic crosstalk and compromised TEC differentiation. Interestingly, depletion of Cbx4 is shown to block both TEC and thymocyte differentiation postnatally but not prenatally,^115^ raising the possibility that postnatal thymopoiesis depends on nclTEC-derived TNCs, consistent with the reported increase in TNC numbers and thymocyte output after birth.^7^^,^^74^^,^^116^^,^^117^

Given the proposed role for nclTECs in T cell maturation, identifying factors that increase their numbers or boost their function might aid therapies aimed at inducing or enhancing T cell function through engineered or transplanted thymic tissue.^118^ Such therapies include thymic transplantation in congenital athymia,^119^ co-transplantation of thymic tissue to promote tolerance in organ transplantation,^120^ and regeneration of the thymus after chemotherapy or radiation. Interestingly, nclTECs share features with progenitors reported to have regenerative capacity in adult thymus, e.g., localization near the CMJ and expression of stromal signaling genes.^24^^,^^25^ Hypothetically, regenerative capacity could be provided by a scarce population of quiescent multipotent nclTECs in adults, supported by results showing that CBX4 maintains a quiescent, undifferentiated state in human epidermal stem cells.^68^

Many cancers are found to originate from progenitor compartments,^121^ and accordingly, impaired differentiation of bipotent progenitors has been implicated in disease progression using a thymoma mouse model.^122^ Notably, nclTEC markers are expressed in various thymic epithelial tumors at the protein level (CD274, TP63, LY75, and FOXN1^123^^,^^124^^,^^125^). Further, a recent thymus single-cell study found *DLL4*, *LY75*, *CCL25*, and *TBATA* to be upregulated in a tumor-specific cluster.^126^ In T cell acute lymphoblastic leukemia, TECs have been shown to promote cancer development, with potential roles for CXCL12,^127^ DLL4,^128^ and FOXN1,^129^ leaving nclTECs as putative targets in future therapy.^130^

### Mimetic TEC differentiation

As TEC(myo)_a expressed genes involved in myogenesis and skeletal muscle, and displayed specific chromatin conformations around key skeletal muscle differentiation genes, we hypothesize that this cluster overlaps with previously reported cross-striated thymic cells^2^^,^^17^ and their progenitors. According to inferred developmental potential, the individual TEC(myo) and TEC(neuro) clusters encompassed both potent and differentiated cells, with an increasing proportion of differentiated cells along the mimetic trajectory from TEC(myo)_a, via TEC(myo)_b, TEC(neuro)_a, and TEC(neuro)_b to Ciliated TECs (Figures 7A and 7B; Table S15). This pattern was also observed for the trajectory toward mTEC(III)_b and implies that within each TEC population, a subset has reached its final maturation stage, while other cells continue to differentiate. It remains to be explored if the developmental potential inferred within mimetic populations reflects putative dividing progenitors, post-mitotic plasticity, or both.

A common differentiation trajectory for TEC(myo)s, TEC(neuro)s, and Ciliated TECs is not previously reported and warrants experimental validation. However, we found support for this model in inferred lineage priming of relevant genes, e.g., for *NKX6-2*, involved in postnatal oligodendrocyte differentiation,^108^ where chromatin became accessible in TEC(myo)_b and TEC(neuro)_a and was followed by expression of the gene in TEC(neuro)_b (Figure S14B). Another example was for *BARHL1*, reported from development/differentiation of sensory cells,^112^ with inferred chromatin priming in TEC(neuro)_b and expression in Ciliated TECs. Further, transition from TEC(myo)_a to TEC(myo)_b, and from TEC(neuro)_a to TEC(neuro)_b, was indicated by sequential expression of genes associated with lineage choice and differentiation in peripheral tissues (*MYOD1* to *MYOG*^78^ and *ASCL1* to *NEUROD1*/*NEUROD2*/*NEUROG1*,^62^ respectively).

Increased heterogeneity in the muscle and neuroendocrine TEC compartment has recently been reported by Huisman et al.^17^ Their “muscle mimetics” expressed *MYOG* along the inferred trajectory, whereas *MYOD1* was lacking in the early cluster—somewhat surprisingly since MYOD1 acts upstream of MYOG during myogenesis.^78^ While their trajectory from intermediary to late muscle mimetics could fit with differentiation within our TEC(myo)_a cluster, their early cluster does not match either of our TEC(myo) clusters, but shares some markers with our TEC(neuro)s and Ciliated TECs (e.g., *PAM* and *SPOCK1*). Further, their “cochlear-hair mimetics” align with our Ciliated TECs, but none of their clusters match well with our TEC(neuro)_a or TEC(neuro)_b populations. Disagreements between our findings and those of Huisman et al. may arise from differences in study resolution, sample processing, and/or analytical methodology.

### Suggested roles for mTEC(III)

HCs are substantially more frequent in human thymi compared to mice,^131^ suggesting a more prominent role in humans. According to our results, mTEC(III)_b, located in HCs, appears to be an essential player in negative selection. These cells expressed a broad repertoire of epithelial tissue TRAs, in addition to genes otherwise seen upon inflammation and infection, suggesting that they mimic these responses to produce specific TRAs. Further, as top DORC genes were associated with GO terms related to glycosylation, it would be interesting to explore whether mTEC(III)_b has a dedicated role in the production of glycosylated TRAs.

In contrast to the reported downregulation of HLA class II in post-Aire/AIRE mTECs,^44^^,^^46^ mTEC(III)_b showed broad expression of class I and II genes, indicating TRA presentation to both CD4^+^ and CD8^+^ thymocytes. On the other hand, our IVL stainings indicate that mTEC(III)_b locates toward the center of HCs, which would limit interactions with other cells. Hence, mTEC(III)_b might release TRAs to the extracellular space for uptake and presentation on professional antigen-presenting cells, as reported for AIRE^+^ mTECs and dendritic cells.^132^ Putative secretion of TRAs from mTEC(III) would be in line with significant GO terms related to intra- and extracellular vesicles in these cells.

Our analyses suggest that mTEC(III)_b has two modes of TRA expression, both coordinated expression induced by specific TFs and stochastic expression mediated by yet unknown factors in interplay with distal regulatory elements. While mTEC(III)_b could be regarded as an epithelial mimetic mTEC, it strongly contrasts with other mimetics in its genome-wide chromatin profile, supporting our choice to address mTEC(III) separately from the remaining mimetic mTECs.

Differentiation from mTEC(III)_a to mTEC(III)_b was suggested by IF staining and trajectory analysis, but no developmental potential was inferred in either population (except for a single oligopotent outlier in mTEC(III)_a). This could indicate that potent mTEC(III)_a was lost during tissue dissociation; however, separate trajectories toward the two mTEC(III) clusters cannot be excluded. Interestingly, Yayon et al. report diverging trajectories toward “mTECIII-muc” (mucosal) and “mTECIII_skn” (skin).^30^ While their mTECIII-muc resembles mTEC(III)_b (e.g., *MUC4*, *CXCL17*, and *SERPINB7*), mTECIII_skn markers are found in both of our mTEC(III) clusters (e.g., *SERPINB3* and *SERPINB4* more DE in mTEC(III)_a; *S100P* and *KLK10* more DE in mTEC(III)_b) (Table S7). These discrepancies could reflect that mTEC(III) is particularly sensitive to the choice of dissociation protocol, with different protocols capturing different cell states to variable extents. In contrast to our findings, several studies, particularly those that isolate TECs by fluorescence-activated cell sorting,^17^^,^^29^ show a relatively scarce mTEC(III) population in the human postnatal thymus. In our experience, the best way to retrieve these cells is to discard non-TECs by gradient centrifugation and CD45 depletion, to include digestion of undissolved HCs, and to separate them from other stromal cells bioinformatically after single-cell sequencing.

### Stochastic and coordinated TRA expression

The widespread SE accessibility observed in differentiated mTEC(III)_b may appear surprising, given the classical role of SEs in controlling cell fate and identity programs. Likewise, early TECs showed accessibility at SEs unlikely to participate in TEC differentiation, e.g., at osteoblast-associated SEs in mcTEC_b (Figure S4). These findings raise the possibility that accessible SEs contribute to the induction of TRA expression. In murine mTECs, a role for Aire-enhanced SE loops in stochastic TRA expression has been suggested,^54^ where recruitment of Aire to SEs is followed by eviction of CTCF from TAD boundaries, favoring transcriptional loops over architectural loops.^55^ Taken together, we hypothesize that SEs and other distal enhancers are involved in stochastic TRA expression in conventional mTECs and mTEC(III), possibly also in TEC(myo)_a. Alternatively, TEC(myo)_a includes cells in transition from conventional mTECs (mainly accessible over SEs) to mimetic mTECs (mainly closed over SEs). A model where stochastic TRA expression is present along the trajectory from mcTEC toward mTEC(III), but not in mimetic mTECs, would also fit the observed differences in CTCF accessibility. Low accessibility over CTCF elements in conventional mTECs and mTEC(III)_b could reflect widespread eviction of CTCF to induce transcriptional loops, while high accessibility in mimetics could reflect re-establishment of architectural loops and increased CTCF insulation, stabilizing cell identities.^133^^,^^134^

Stochastic gene expression seemed to be present to some extent across all TECs in our data (Figure 3B). However, low and scattered transcript levels appeared across all thymic cell types (data not shown), indicating that background noise was too high to draw conclusions about stochastic TRA expression. To overcome this issue, future studies should include more sensitive approaches. Recently, Matsumoto et al.^21^ applied RamDA-seq^135^ on individual murine TECs. Their analysis of Aire-dependent gene expression revealed a clearer separation between stochastic and non-stochastic expression than what we observed in our heatmap (Figure 3B). Of note, their study supported the view that stochastic expression is present in Aire^+^ mTECs, while coordinated expression occurs in mimetic TECs.

### A joint model for TEC differentiation and TRA expression

**Figure 8:**
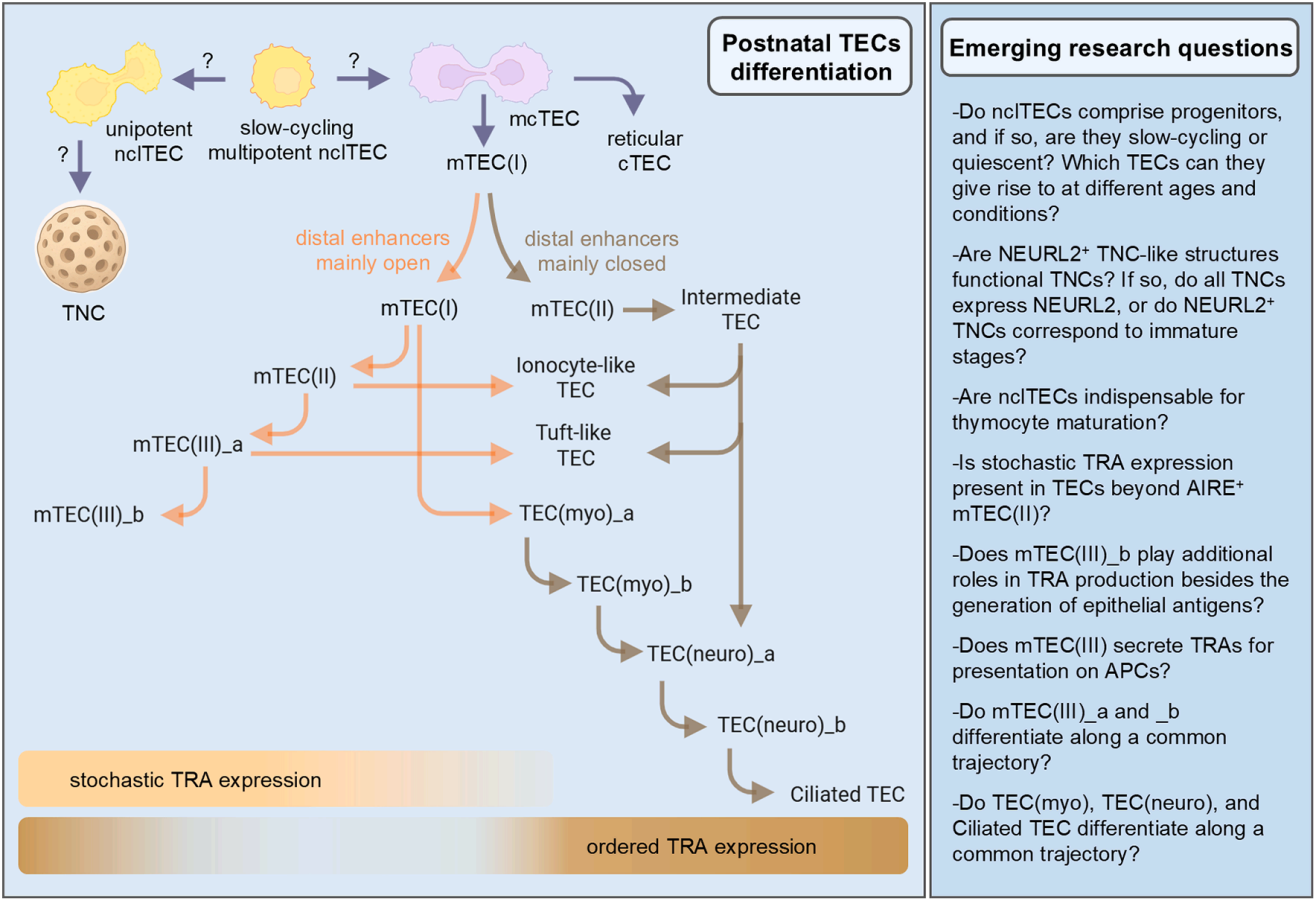
Emerging research questions and a joint model for postnatal TEC differentiation and TRA expression In the postnatal/pediatric thymus, we propose that unipotent nclTECs give rise to TNCs and that the unipotent nclTEC population is maintained by scarce slow-cycling multipotent nclTECs. These slow-cycling cells might also sustain the mcTEC population. Multipotent mcTECs give rise to reticular cTECs, mTECs, and mimetic mTECs. Differentiation toward mTEC(III)_b is characterized by chromatin accessibility over distal enhancers, including SEs, and we relate this accessibility to stochastic TRA expression. In most mimetic mTECs, distal enhancer accessibility is restricted to cell-type-specific enhancers, in line with coordinated TRA expression driven by master TFs. TF-driven TRA expression is also seen in mTECs, especially in mTEC(III)_b. Some mimetic mTECs, particularly TEC(myo)_a, show an open conformation over distal enhancers, which we assume reflects alternative differentiation pathways toward mimetics. Figure created with BioRender.com. TNC illustration by ChatGPT.

Single-cell studies are inherently explorative and provide an ideal basis for hypothesis generation. Thus, we have highlighted key research questions emerging from the present work (Figure 8, right panel) and propose a joint model for postnatal TEC differentiation and TRA expression (Figure 8, left panel). We hypothesize that TEC differentiation separates into trajectories toward TNCs, reticular cTECs, mTEC(III)_b, or mimetic mTECs. In our model, TRA expression is associated with changes in chromatin accessibility as TECs differentiate, with stochastic expression in interplay with distal enhancers restricted to cells along the mTEC(III)_b trajectory. Coordinated expression initiated by master TFs is more widespread, but particularly evident in mTEC(III)_b and mimetic TECs.

As mimetics are reported to develop from both Aire^+^ and Aire^−^ TECs,^11^^,^^15^^,^^16^ we suggest alternative crossroads where the mimetic trajectory could diverge from the conventional TEC trajectory. We hypothesize that the minor proportion of mTEC(I) with relatively closed distal enhancers is destined for a mimetic fate via mTEC(II), in line with low accessibility over distal enhancers in most mimetics. A substantial portion of TEC(myo)_a had an open conformation over these elements, which could imply that TEC(myo)_a diverges from the main mimetic differentiation trajectory at an earlier stage, supported by the notion that muscle TECs seem to develop independently of Aire.^15^ A similar pattern was observed for the Ionocyte-like TEC cluster; hence, we propose that these cells might follow an alternative pre-AIRE trajectory in addition to their reported differentiation from Aire^+^ cells.^15^ Such a scenario, with two possible trajectories toward most mimetics, would explain their reported differentiation from both pre-*Aire* and post*-Aire* precursors.

### Limitations of the study

Ideally, our study would include more samples across different age spans, e.g., for further assessment of nclTECs. An optimal setup would also include scATAC-seq performed on freshly isolated samples in parallel with the initial scRNA-seq experiments. With conventional cTECs missing from our scATAC-seq dataset, our focus was on the mTECs in the chromatin-related analyses. As these analyses lacked mTEC(III)_a, we do not have DORC predictions and other informative chromatin measures from these cells. Hence, we call for future studies to cover these gaps.

Recovery of intact reticular TECs and HC-derived cells from dissociated pediatric thymus is challenging. This is indicated by the loss of mitochondrial transcripts by us and others, and one should be aware of the confounding effects from poor-quality cells. Although we cannot rule out effects from potential loss of cytoplasmic RNA from mTEC(III)_a and cTECs, our IF stainings confirmed that there are true biological differences in localization and cell morphology between mTEC(III)_a and mTEC(III)_b, and between cTECs and nclTECs. In the future, combining data from freshly isolated single nuclei with high-resolution spatial transcriptomics might uncover the *in vivo* proportions of the various TEC populations and provide an even more comprehensive understanding of their nature.

Another limitation of our study is the lack of a specific marker for human TNCs, leaving their identity to be inferred from morphology in the dual-marker staining procedure. The stainings performed to identify and localize nclTECs would benefit from a large panel of markers, including those inferred to convey signaling between nclTECs and thymocytes. Antibody staining of thymic sections is challenged by high autofluorescence and background levels (probably influenced by widespread TRA expression), and additionally, two-dimensional cell segmentation is difficult due to the inherent tissue structure with epithelial networks densely packed with thymocytes. To obtain high-quality images of nclTECs and their *in situ* interacting partners, manual optimization should be combined with multiplexed imaging techniques, preferably in parallel with high-resolution spatial transcriptomics. Further, functional validations are needed to settle whether nclTECs do play an essential role in thymopoiesis, if they are indeed progenitors, and if so, which cells they give rise to at different ages.

### Concluding remarks

Detailed knowledge of TECs and their interplay with developing thymocytes is crucial to improve or develop therapies related to T cell or thymic dysfunction. In this study, we identify increased cell type heterogeneity in the pediatric thymus, describe changes in chromatin conformation along inferred differentiation trajectories, and propose a model in which distinct chromatin accessibility profiles reflect underlying regulatory mechanisms controlling TRA expression. Further, we characterize a NEURL2^+^ TEC population, which might prove important in therapeutic settings, suggested to include progenitors and to be essential for thymopoiesis.

## Resource availability

### Lead contact

Requests for further information and resources should be directed to and will be fulfilled by the lead contact, Hanne S. Hjorthaug (uxhjhb@ous-hf.no).

### Materials availability

This study did not generate new material.

### Data and code availability

• Single-cell ATAC-seq and Multiome-seq data have been deposited at GEO: GSE318154 (ATAC) and GEO: GSE318155 (Multiome).
• This paper does not report original code.
• Any additional information required to reanalyze the data reported in this paper is available from the lead contact upon request.

## Acknowledgments

We are grateful to the donors and their parents for their participation in this study, and to Karl-Andreas Dumont and staff at the Department of Cardiothoracic Surgery at the Oslo University Hospital, who facilitated sample collection. Cryosectioning was performed by Don Trinh at the Department of Pathology, Oslo University Hospital. Sequencing and data preprocessing in Cell Ranger ATAC/ARC were performed by the Norwegian Sequencing Center, a national technology platform hosted by the University of Oslo and Oslo University Hospital, supported by the Research Council of Norway and the Southeastern Regional Health Authorities. We are also thankful to Pål Marius Bjørnstad (Department of Medical Genetics, Oslo University Hospital and University of Oslo) for help with setting up Conda and ChromaFold environments. Computational resources were provided by Tjenester for Sensitive Data (TSD) facilities (University of Oslo) and the Norwegian Research Infrastructure Services (NRIS). Funding was provided by the 10.13039/501100005416Norwegian Research Council (project number 274718) and 10.13039/501100006095South-Eastern Norway Regional Health Authority (project number 2023065).

## Author contributions

Conceptualization, H.S.H., M.H., S.T.F., and B.A.L.; methodology, H.S.H., M.H., S.T.F., and B.A.L.; formal analysis, H.S.H., M.H., and B.A.L.; investigation, H.S.H., M.H., and S.T.F.; data curation, H.S.H., M.H., S.T.F., and B.A.L.; writing – original draft, H.S.H. and M.H., writing – review and editing, H.S.H., M.H., S.T.F., and B.A.L., visualization, H.S.H. and M.H.; supervision, B.A.L.; project administration, B.A.L.; funding acquisition, B.A.L.

## Declaration of interests

The authors declare no competing interests.

## Declaration of generative AI and AI-assisted technologies in the writing process

During the preparation of this work, the authors used ChatGPT and CoPilot to improve readability in selected sentences and paragraphs. After using these tools, the authors reviewed and edited the content as needed and take full responsibility for the content of the publication.

## STAR★Methods

### Key resources table

**Table:** 

REAGENT or RESOURCE	SOURCE	IDENTIFIER
**Antibodies**		

AffiniPure Fab Fragment Goat Anti-Rabbit IgG (1.3 μg/L)	Jackson ImmunoResearch Labs	Cat# 111-007-003; RRID:AB_2337925
anti-IVL (rabbit polyclonal IgG, 0.08 μg/μl)	AtlasAntibodies	Cat# HPA055211; RRID: AB_268273
anti-KRT1 (rabbit polyclonal IgG, 0.05 μg/μl)	AtlasAntibodies	Cat# HPA017917; RRID: AB_1852192
anti-NEURL2 (rabbit polyclonal IgG, 0.1 μg/μl)	AtlasAntibodies	Cat# HPA059842; RRID: AB_2684139
anti-KRT8 (rabbit polyclonal IgG, 0.05 μg/μl)	AtlasAntibodies	Cat# HPA049866; RRID: AB_2680923
isotype control (anti-cellulase (Arabidopsis thaliana)) (rabbit polyclonal IgG, 1 μg/μl)	Bioss Antibodies	Cat# bs-2222R-BF647-20
anti-Rabbit, IgG Highly Cross-Adsorbed, Alexa Fluor™ Plus 647 (goat polyclonal, 2 μg/μl)	Thermo Fisher Scientific	Cat# A32733; RRID:AB_2633282
anti-Rabbit IgG, Highly Cross-Adsorbed, Alexa Fluor™ Plus 555 (goat polyclonal, 2 μg/μl)	Thermo Fisher Scientific	Cat# A32732; RRID:AB_2633281

**Biological samples**		

Human postnatal thymic tissue	Department of Cardiothoracic Surgery, Oslo University Hospital	NA

**Chemicals, peptides, and recombinant proteins**		

Optimal Cutting Temperature compound	CellPath	Cat# KMA-0100-004
PBS	ThermoFisher Scientific	Cat# 14190094
Formaldehyde 16%	ThermoFisher Scientific	Cat# 28906
Triton X-100	Sigma-Aldrich	Cat# T8787
Goat serum	Jackson ImmunoResearch Labs	Cat# 005-000-121
Rabbit serum	Jackson ImmunoResearch Labs	Cat# 011-000-120
FcR Blocking Reagent	Miltenyi	Cat# 130-059-901
DAPI	Sigma-Aldrich	Cat# D9542
TrueVIEW Autofluorescence Quenching Kit, including VECTASHIELD Vibrance Antifade Mounting Medium	Vector Laboratories	Cat# SP-8400
RPMI	Sigma-Aldrich	Cat# R7509
FBS	Biowest	Cat# S181H-500
Tween 20	Sigma-Aldrich	Cat# P1379
Nonidet P40	Sigma-Aldrich	Cat# 127087-87-0
Digitonin 5%	ThermoFisher Scientific	Cat# BN2006
BSA	ThermoFisher Scientific	Cat# AM2616
RNase inhibitor	ThermoFisher Scientific	Cat# 18091050
DTT 1M	Qiagen	Cat# 1032395
DNaseI	Sigma-Aldrich	Cat# 11284932001

**Critical commercial assays**		

Chromium Single Cell ATAC Library & Gel Bead Kit, 4 rxns	10*x* Genomics	PN-1000111
Chromium Chip E Single Cell ATAC Kit, 16 rxns	10*x* Genomics	PN-1000086
Chromium i7 Multiplex Kit N, Set A, 96 rxns	10*x* Genomics	PN-1000084
Chromium Next GEM Single Cell ATAC Library & Gel Bead Kit, 4 rxns	10*x* Genomics	PN-1000176
Chromium Next GEM Chip H Single Cell Kit, 16 rxns	10*x* Genomics	PN-1000162
Single Index Kit N, Set A, 96 rxns	10*x* Genomics	PN-1000212
Chromium Next GEM Single Cell Multiome ATAC + Gene Expression Reagent Bundle, 4 rxns	10*x* Genomics	PN-1000285
Chromium Next GEM Chip J Single Cell Kit, 16 rxns	10*x* Genomics	PN-1000230
Single Index Kit N Set A, 96 rxns	10*x* Genomics	PN-1000212
Dual Index Kit TT Set A, 96 rxns	10*x* Genomics	PN-1000215

**Deposited data**		

scRNA-seq on thymic cells	Heimli et al.^57^	GEO: GSE207204
scATAC-seq on stromal-enriched thymic cells	Present study	GEO: GSE318154
scMultiome-seq on stromal-enriched thymic cells	Present study	GEO: GSE318155
HTA08.v01.A05.Science_human_epi.h5ad (scRNA-seq on thymic epithelial cells)	Park et al.^13^	https://zenodo.org/records/3572422
GSE147520_epithelial_cells.h5ad (scRNA-seq on thymic epithelial cells)	Bautista et al.^14^	GEO: GSE147520

**Software and algorithms**		

R v.4.1.2/4.1.3/4.3.2	R Core Team	https://www.r-project.org/
RStudio 2022.02.0/2021.09.1/2023.12.1	R Core Team	https://posit.co/products/open-source/rstudio
Anaconda3 2022.10	Anaconda Software Distribution	https://www.anaconda.com/
Python 3.8.12	Python Software Foundation	https://www.python.org/
Seurat v.4.1.1/4.0.4	Hao et al.^136^	https://github.com/satijalab/seurat/releases
Signac v.1.7	Stuart et al.^137^	https://github.com/stuartlab/signac/releases
ggplot2	Wickham^138^	https://ggplot2.tidyverse.org/
ComplexHeatmap	Gu^139^	https://github.com/jokergoo/ComplexHeatmap
g:Profiler version e113_e.g.,59_p19_f6a03c19	Kolberg et al.^140^	https://biit.cs.ut.ee/gprofiler
CellPhoneDB v.5.0.1	Troulé et al.^141^	https://github.com/ventolab/CellphoneDB
CytoTRACE 2	Kang et al.^142^	https://github.com/digitalcytometry/cytotrace2
Monocle3 v.1.0.0	Cao et al.^143^	https://github.com/cole-trapnell-lab/monocle3
SCENIC v.1.3.1	Van de Sande et al.^144^	https://github.com/aertslab/SCENIC
MACS2 v.2.2.7.1	Gaspar^145^	https://pypi.org/project/MACS2/2.2.7.1/
SoupX v.1.6.1	Young, Behjati^146^	https://github.com/constantAmateur/SoupX
Genomic Regions Enrichment of Annotations Tool (GREAT) v.4.0.4	Tanigawa et al.^147^	https://great.stanford.edu
chromVAR v.1.16.0	Schep et al.^148^	https://github.com/GreenleafLab/chromVAR
GenomicRanges v.1.42.0	Lawrence et al.^149^	https://bioconductor.org/
ChromaFold	Gao et al.^150^	https://github.com/viannegao/ChromaFold
ArchR v.1.0.2	Granja et al.^151^	https://www.archrproject.com/
Jupyter Notebook v.7.2.2	Kluyver et al.^152^	https://jupyter.org/
matplotlib v.3.7.5	Hunter^153^	https://matplotlib.org/
CoolBox v.0.3.7	Xu t al.^154^	https://gangcaolab.github.io/CoolBox/
FigR	Kartha et al.^102^	https://buenrostrolab.github.io/FigR/
3D Genome Browser 2.0	Yu et al.^155^	https://3dgenome.fsm.northwestern.edu

**Other**		

Demonstrated Protocol - Nuclei Isolation for Single Cell ATAC Sequencing CG000169 Rev D	10*x* Genomics	https://www.10xgenomics.com/
Chromium Single Cell ATAC Reagent Kits User Guide CG000168 Rev B	10*x* Genomics	https://www.10xgenomics.com/
Chromium Next GEM Single Cell ATAC Reagent Kits v1.1 User Guide CG000209 Rev D	10*x* Genomics	https://www.10xgenomics.com/
Chromium Next GEM Single Cell ATAC + Gene Expression User Guide, CG000338 Rev E	10*x* Genomics	https://www.10xgenomics.com/
The Human Protein Atlas v.22.0	Uhlén et al.^156^	https://www.proteinatlas.org/
ENCODE Registry of cCREs	Abascal et al.^157^	https://screen.encodeproject.org/index/cversions
refTSS (reference dataset for transcriptional start sites)	Abugessaisa et al.^158^	https://reftss.riken.jp/datafiles/current/human/
SEdb (Super-enhancer database)	Song et al.^159^	http://www.licpathway.net/sedb
*Cis*-BP database	Weirauch et al.^160^	https://cisbp.ccbr.utoronto.ca/

### Experimental model and study participant details

Thymic tissues were collected from young pediatric donors undergoing corrective heart surgery at the Department of Cardiothoracic Surgery, Oslo University Hospital (Table S1).

### Ethical statement

The project was approved by the Regional Ethics Committee of South East Norway (REC 31516) and conducted in accordance with Declaration of Helsinki and applicable institutional and national guidelines. Written informed consent was obtained from donor parents.

### Method details

#### Sample processing for single-cell sequencing

Thymic tissue was cut into pieces, and either immediately processed for single-cell sequencing or snap-frozen and embedded in Optimal Cutting Temperature compound (CellPath Cat# KMA-0100-004) for storage at −80°C. Tissue dissociation and cell enrichment for single-cell sequencing were performed as part of our previous study.^57^ This processing resulted in three different thymic single-cell samples per donor, namely an unenriched sample, a sample enriched for antigen-presenting cells, and a CD45-depleted sample (termed the “stromal-enriched” sample in the present work). Cells in surplus were resuspended in fetal bovine serum supplemented with 10% dimethyl sulfoxide, and stored at −196°C.

#### Single-cell library preparations

##### scRNA-seq

scRNA-seq libraries were prepared in previous experiments (as part of Cellular Indexing of Transcriptomes and Epitopes by sequencing (CITE-seq)).^57^

##### scATAC-seq

For each sample, 200,000–400,000 cryopreserved cells were thawed, and nuclei isolated according to the procedure for primary/fragile cells in 10*x* Genomics’ Demonstrated Protocol - Nuclei Isolation for Single Cell ATAC Sequencing CG000169 revision D, with the following adjustments: All centrifugations at 340 rcf; Before the second centrifugation, 9 mL RPMI supplemented with 10% FBS and 5 μg/mL DNaseI (Sigma-Aldrich Cat# 11284932001) was added for a 5 min DNaseI treatment at room temperature (except for donor 2); After the second centrifugation and resuspension, a 30 μm cell strainer (Miltenyi Biotec Cat# 130-098-458) was placed on top of a 15-mL polystyrene conical tube, and cell suspension was passed through; An aliquot of the filtered cell suspension was used for counting on Nuclecounter NC-100 (ChemoMetec), before the filter was placed back on top of the 15 mL tube and rinsed with 3 mL PBS supplemented with 0.04% BSA. From this step onward, the instructions for “Low Cell Input Nuclei Isolation” were followed, except that the first centrifugation was performed at 340 rcf, and counting was carried out on Nucleocounter.

For donor 1, 2, and 5, sequencing libraries were prepared according to 10*x* Genomics Chromium Single Cell ATAC Reagent Kits User Guide revision B, while donor 3 and 4 were prepared as described in the 10*x* Genomics Chromium Next GEM Single Cell ATAC Reagent Kits v1.1 User Guide revision D. Donor 2 was the first sample to be prepared and was subject to a pilot 71 bp paired end (PE) sequencing on Illumina NextSeq. As downstream analyses indicated substantial amounts of free-floating DNA in the sample, the remaining samples (donor 1, 3–5) were treated with DNaseI at the beginning of the nuclei isolation procedure. Free-floating DNA was filtered out in downstream quality control (QC) analysis, and Donor 2 did not deviate from the remaining donors in post-filtering QC plots. For all donors, it was aimed for a recovery of 10,000 cells for sequencing. Libraries were sequenced 151 bp PE on NovaSeq6000, either on a ½ SP flowcell each (donors 1 and 2), or as a pool on a ¼ S4 flowcell (donors 3–5).

##### scMultiome-seq

Cells from each donor were combined for simultaneous single-cell RNA and ATAC sequencing using the 10*x* Genomics Multiome approach. Cells were thawed, counted, and pooled (25,000 cells per sample) before nuclei isolation as described for scATAC-seq, using the standard nuclei isolation procedure. Libraries were prepared according to the 10*x* Genomics Chromium Next GEM Single Cell ATAC + Gene Expression User Guide, revision E, aiming at 10,000 recovered cells. For the scRNA-seq library, 14 cycles were used in the index PCR. Each library was sequenced on a ½ SP NovaSeq6000 flowcell, 100 bp PE.

#### Immunofluorescence microscopy

3 μm cryosections of freshly frozen thymic tissues were mounted on glass slides, with three successively cut small sections, or one large section, per slide. The slides were stored at −80°C prior to IF staining. Solutions are listed in Table S22. Antibodies against human NEURL2 (Cat# HPA059842, RRID: AB_2684139), KRT8 (Cat# HPA049866, RRID: AB_2680923), KRT1 (Cat# HPA017917, RRID: AB_1852192), and IVL (Cat# HPA055211, RRID: AB_268273) were purchased from Atlas Antibodies, with specificity thoroughly validated by the manufacturer. As these were all produced in rabbit, additional blocking steps were applied to ensure target-specific signals in our double-staining experiments, following the recommendations of Jackson ImmunoResearch (https://www.jacksonimmuno.com/technical/products/protocols/double-labeling-same-species-primary/example-c). The sequence of the antibody incubations was as follows: 1) For double-staining of NEURL2 and KRT8: anti-NEURL2, Alexa Fluor (AF) Plus 555-conjugated secondary antibody (Thermo Fisher Scientific Cat# A32732, RRID:AB_2633281), anti-KRT8, Alexa Fluor Plus 647-conjugated secondary antibody (Thermo Fisher Scientific Cat# A32733, RRID:AB_2633282). 2) For double-staining of IVL and KRT1: anti-IVL, Alexa Fluor Plus 647-conjugated secondary antibody, anti-KRT1, Alexa Fluor Plus 555-conjugated secondary antibody. For all donors and for the double-staining procedures, control stainings were performed by substituting either the first or the second primary antibody with an isotype control (anti-cellulase (Bioss Antibodies Cat# bs-2222R-BF647-20)). The double-staining was applied to the middle section on each slide, with the control stainings at either side, except for the double-staining of NEURL2 and KRT8 for donor 6. In this case, two successively cut sections were used: one for the double-staining and one for both control stainings, with the latter separated into two areas using a PAP pen.

The stainings were performed as follows: Slides were equilibrated to 4°C (−20°C at 60 min, 4°C at 10 min), fixed in Fixation solution for 15 min on ice, and washed in cold PBS (2 × 5 min), with this and subsequent washes performed with gentle shaking (200 rpm). For double-stainings with anti-NEURL2 and anti-KRT8, slides were incubated in Permeabilization solution at room temperature (RT) for 2 × 5 min and rinsed in PBS (no permeabilization for anti-IVL and anti-KRT1 double-staining). Next, slides were incubated in Blocking solution 1 at RT for 45 min, with this and subsequent incubations performed in a hydration chamber. The blocking solution was removed, and the slides were incubated with the first primary antibody (or isotype control) at 4°C overnight. After this incubation, and all subsequent antibody/blocking steps, slides were washed 3 × 5 min in Washing solution, followed by 3 × 5 min in PBS. Further incubations (at RT in the dark) were: The first secondary antibody (45 min), Blocking solution 2 (60 min), Blocking solution 3 (60 min), the last primary antibody (or isotype control) (60 min), the last secondary antibody (45 min), and DAPI (Sigma-Aldrich Cat# D9542) (15 min). After incubation with DAPI, slides were washed 2 times for 5 min each in PBS, and excess buffer was removed. Quenching and mounting of coverslips were performed according to instructions in the TrueVIEW Autofluorescence Quenching Kit (Vector Laboratories Cat# SP-8400).

Multichannel IF was assessed with a Nikon Eclipse E600 microscope equipped with a D-LEDI light source and a DS-Fi3 camera, using the Cy5 and Muli-LED (DAPI/FITC/TRITC) filter blocks and the NIS-Elements BR 5.42.04 Software (Nikon). For imaging of control stainings, acquisition settings were equal to those used for the corresponding double-staining image. The following objectives were used: Plan Fluor 20× (numerical aperture (NA) 0.5; Figures 5A, 5B, and 5D_left), Plan Fluor ELWD 40*x* (NA 0.6; Figures 5K_left, S9A, S9C, and S9D), Plan Apo 60*x* oil (NA 1.4; Figure S9E), and Plan Apo VC 100*x* oil (NA 1.4; remaining images in Figures 5 and S9). Adjustments of images presented in Figures 5 and S9 were done in NIS-Elements Viewer (available from https://www.nisoftware.net/NikonSaleApplication/Default.aspx) using linear lookup tables (LUTs). For control staining images, TRITC and Cy5 channel LUTs were the same as the LUTs applied to the corresponding double-staining image. Some images were out of focus in the DAPI channel, and these were sharpened in GIMP^161^ 3.0.6–1 with the Unmask Sharp filter (Figures 5D_left, 5K, and S9C). Uncropped, grayscale images for each color channel are provided in Document S1. Raw image files and the LUTs applied to these are available as supplemental material (Data S2).

Before settling on the above protocol, antibody concentrations and blocking conditions were optimized. Initially, we tried to stain for NEURL2 together with a combined nclTEC and cTEC marker, i.e., PRSS16 or PSMB11. We obtained signals from both anti-PRSS16 and anti-PSMB11 in single-stain experiments, but the signals were too dim to be recapitulated in the double-staining protocol.

### Quantification and statistical analysis

Most analyses were performed in RStudio 2022.02.0/2021.09.1/2023.12.1 using R v.4.1.2/4.1.3/4.3.2. For analyses requiring high memory, computations were performed on a high-performance computing cluster (Norwegian Research Infrastructure Services (NRIS)) using R v.4.1.2 (except for R v.4.0.3 used for peak calling) or a Conda environment (Anaconda3 2022.10) configured with Python 3.8.12. R packages Seurat v.4.1.1/4.0.4^136^ and Signac v.1.7^137^ were used for quality control (QC) and several downstream analyses. Unless described, arguments in Seurat/Signac functions were kept at their default settings. Plots were generated using the relevant analysis package and/or ggplot2,^138^ or the ComplexHeatmap package.^139^^,^^162^

#### scRNA-seq data processing

The processing and analysis of thymic scRNA-seq data are described in Heimli et al.^57^ (data available at GEO: GSE207204), with the following datasets used in the present study: 1) The full thymic dataset, integrated from unenriched, antigen-presenting-cells-enriched, and stromal-enriched samples. 2) The stromal-enriched dataset, used in cell type annotation of the scATAC-seq stromal-enriched dataset. 3) The TEC dataset, in-silico subsetted from the stromal-enriched dataset. For the present study, clusters in the TEC dataset were manually annotated using a cluster resolution of 1.2 (increased from 0.8 in Heimli et al.), based on marker genes from the literature and from differential expression analysis.

#### Analyses of scRNA-seq data

##### Differentially expressed genes

Differential gene expression analysis between one cell type versus the remaining cell types in the scRNA-seq TEC dataset was performed using the Seurat FindMarkers() function, with min.pct set to 0.25 and only.pos set to TRUE. Similarly, analysis was performed between two cell clusters (TEC(neuro)_a vs. TEC(neuro)_b, TEC(neuro)_b vs. TEC(neuro)_a), and between nclTECs inferred with different developmental potential.

##### Gene set enrichment analysis

Gene set enrichment analysis was performed using g:Profiler^140^ (version e113_e.g.,59_p19_f6a03c19) with g:SCS multiple testing correction method, applying a significance threshold of 0.05. For analysis of one cell type vs. the remaining cell types, all DE genes with an adjusted *p*-value <0.05 for the given cell type were used as input. For analysis of one cell type vs. another cell type, the 100 genes with the highest fold change were used as input. These genes had an adjusted *p*-value <0.05 from the differential expression analysis, except for the 11 least significant genes among the top 100 TEC(neuro)_a vs. TEC(neuro)_b DE genes.

##### Cell signaling by CellPhoneDB

To infer receptor-ligand interactions, CellPhoneDB v.5.0.1^141^ analysis was applied to the full thymic scRNA-seq dataset, using the “statistical method”/“Method 2” (https://cellphonedb.readthedocs.io). Notably, for populations that received additional annotations and filtering of doublet/contaminating clusters after subsetting, including TECs (this work), dendritic cells,^57^ and B cells,^57^ the more detailed subset annotations were used. The significance level was kept at the default value (0.05).

##### Developmental potential by CytoTRACE 2

Developmental potential of TECs was inferred using CytoTRACE 2 v1.10,^142^ as detailed at https://github.com/digitalcytometry/cytotrace2?tab=readme-ov-file#readme, using the counts table of the scRNA-seq TEC dataset as input.

##### Pseudotime analysis by Monocle3

Single-cell trajectories were built using Monocle3 v.1.0.0,^143^ as outlined in the vignette (https://cole-trapnell-lab.github.io/monocle3/docs/trajectories/). First, the scRNA-seq TEC dataset was converted using cell_data_set(), followed by cell clustering. Uniform Manifold Approximation and Projection (UMAP) was used as reduction method. Graphs were learned with use_partition set to TRUE. For cell ordering, the starting node was set to the first node in the mcTEC cluster (for the graph encompassing conventional TECs), and to the node on the border between TEC(myo)_a and TEC(myo)_b (for the mimetic mTECs graph).

##### RNA velocity

RNA velocity leverages the relationship between unspliced and spliced reads to model gene expression dynamics, implemented in scVelo as either a “steady-state (stochastic)” or “dynamical” model.^163^ For these analyses, raw counts tables, separated into spliced, unspliced, or ambiguous reads, were prepared from the Cell Ranger count matrices for the scRNA-seq stromal-enriched samples using velocyto^164^ v.0.17.17 in Python 3.10.8. These counts were subsequently loaded as an AnnData object using Scanpy^165^ v.10.10.4 and scVelo v.0.3.3 in Python 3.12.4. Analyses were guided by instructions at https://github.com/hamidghaedi/scRNA_velocity_analysis, https://scvelo.readthedocs.io/en/stable/VelocityBasics.html, and https://scvelo.readthedocs.io/en/stable/DynamicalModeling.html. Relevant information from previous analyses, including UMAP coordinates, cluster identities, and annotations for each cell barcode, was transferred from the TEC Seurat object, enabling cell filtering of the AnnData object to only retain cells included in the TEC dataset, as well as downstream visualizations of velocities using the previously defined UMAP coordinates. Filtering and normalization of the AnnData gene counts were performed as implemented in the scVelo pp.filter_and_normalize() function, requiring a shared count value of 30 for a gene to be kept.

Principal component analysis followed by neighborhood graph construction was run as implemented in the Scanpy pp.pca() and pp.neighbor() functions, with 30 principal components retained and 30 neighbors for graph construction. Next, moments were computed using the scVelo pp.moments() function. Velocities and velocity graphs were calculated using either 1) the steady state (stochastic) model by applying the scVelo functions tl.velocity() and tl.velocity graph() with default parameters, or 2) the dynamical model by running the tl.recover_dynamics() function followed by tl.velocity() (setting mode = ‘dynamical’) and tl.velocity graph(). Visualization was performed by the scVelo pl.velocity_emedding_stream() function, setting basis = ‘umap’.

##### Tissue-restricted antigen expression

A list of TRAs was obtained from the Human Protein Atlas (HPA)^156^ v.22.0 (www.proteinatlas.org), with each gene belonging to one of either categories: 1) tissue-enriched genes (*n* = 3,106) having mRNA levels > 4-fold higher in one tissue compared to any other tissue, 2) group-enriched genes (*n* = 1,628) having mRNA levels > 4-fold higher in a restricted number of 2–5 tissues compared to any other tissue, 3) tissue-enhanced genes (*n* = 6,252) having at least 4-fold higher expression in a particular tissue compared to the average mRNA level in all other tissues. In subsequent analyses, a TRA was defined as expressed if the transcript was detected in 3 or more TECs, as given by the Seurat counts table. TRA expression was explored in two ways: Calculation of TRA-enrichment in SCENIC regulons (based on all three HPA categories) and visualization of tissue-specific TRA expression across cell types (based on HPA tissue-enriched genes).

##### Gene regulation by SCENIC

SCENIC^144^^,^^166^ v.1.3.1 predicts TF regulators and their target genes (regulons), based on co-expression with the TF and enrichment of the TF motif within a 10 kb region around the genes’ TSS. For each regulon, each cell is assigned an individual activity score based on expression of the inferred target genes. Analyses were run according to the vignette (http://htmlpreview.github.io/?https://github.com/aertslab/SCENIC/blob/master/inst/doc/SCENIC_Running.html, built on Oct 13, 2020) with the counts table of the scRNA-seq TEC dataset as input. Genes were kept if 1) they were expressed with a UMI count corresponding to a value of 3 in 1% of the cells, 2) they were expressed in at least 3 cells within the TEC object, and 3) they were available in the RcisTarget databases described for subsequent steps, resulting in 10,324 retained genes. To identify putative gene regulatory networks based on co-expression patterns, GENIE3 v.1.24.0 was run on the filtered gene matrix, which outputs a matrix of weights for putative regulatory TF-target gene links. Candidate regulons were then identified using multiple distinct selection criteria implemented in the *runSCENIC_1_coexNetwork2modules()* function, including selection of the best target genes for each TF based on the target weights, and conversely, selection of the best TF regulators for each gene by selecting targets for which the TF was within the top regulators. Only positively correlated TF-target gene pairs, as determined by a Spearman correlation implemented in the *runCorrelation()* function, were retained.

As they were inferred from co-expression patterns, the candidate regulons could contain both direct and indirect targets of the regulatory TF. Hence, as implemented in the *runSCENIC2_createRegulons()* function, RcisTarget v.1.19.2 was used to assess whether target genes were enriched for the regulating TF’s binding motifs within their *cis*-regulatory regions. For motif enrichment, RcisTarget confers two built-in databases containing gene-motif rankings. The databases differ in the boundaries of the gene regulatory regions, defined as either 500 bp upstream of the TSS or 10 kb centered around the TSS. Next, the motifs from the enrichment analysis were annotated to TFs, resulting in two classes of regulons: 1) High confidence, exhibiting enrichment of binding motifs for the regulating TF within the regulatory regions of the co-expressed genes as identified in *Homo sapiens* or inferred from the orthologous species, or 2) Low confidence, having such enrichment inferred by motif similarity. Finally, AUCell v.1.24.0 was used to predict activity at the cellular level, as implemented in the *runSCENIC_3_scoreCells()* function, yielding an area under the curve value that describes the extent to which the genes in each regulon are expressed in a given cell.

We only considered the high-confidence regulons (*n* = 131) for downstream investigations. To increase confidence and align with other analyses, we restricted the regulons to those with their TF motif in the cisBP_human_pfms_2021 database^160^ (downloaded from https://github.com/buenrostrolab/FigR/tree/master/data), resulting in a set of 117 regulons. To focus on cell type specificity, we identified, for each cell type, the 40 regulons with the highest mean activity and used their union (*n* = 88) for further exploration.

TRA-enrichment in SCENIC regulons was tested for statistical significance using Pearson’s *χ*2 or Fisher’s exact test, and the *p*-values were Bonferroni corrected. Regulons with adjusted *p*-values <0.05 were considered significantly enriched in TRAs, or deprived of TRAs.

#### scMultiome-seq data processing

Raw sequencing data from the scMultiome-seq stromal-enriched sample were processed by the 10*x* Genomics Cell Ranger ARC v.2.0.2, using the GRCh38-2020-A-2.0.0 reference path.

##### Peak calling in the scMultiome-seq stromal-enriched dataset

Before peak calling, the Cell Ranger ARC ATAC fragment file was filtered to remove barcodes unlikely to represent intact nuclei, based on low read depth. First, fragments were counted by the Signac CountFragments() function, followed by visualization of fragment frequency counts in a histogram. Based on the histogram, a read depth cutoff was set to 10^2.1^, and fragments above that cutoff were kept by applying the Signac FilterCells() function on the fragment files. MACS2^145^ v.2.2.7.1 was used for initial peak calling on the filtered fragment file using the CallPeaks() function in Signac.

##### QC and preprocessing of the scMultiome-seq stromal-enriched dataset

Ambient RNA was removed using SoupX^146^ v.1.6.1, following standard procedure (https://github.com/cran/SoupX/blob/master/README.md). Next, a Seurat object was created to hold cells expressing at least 100 genes, and genes being expressed in at least 3 cells. The ATAC data were added to the object, with the peak matrix filtered to remove the following: Peaks in nonstandard chromosomes and genomic blacklisted regions, barcodes not present in the RNA assay, and barcodes having less than 500 peaks. The ATAC assay was normalized (RunTFIDF()) and subject to linear dimensional reduction (FindTopFeatures() with min.cutoff = 20, RunSVD()), before applying nonlinear dimensional reduction (RunUMAP() with reduction = ‘lsi' & dims = 2:50) and clustering (FindNeighbors() with reduction = ‘lsi’, dims = 2:50, and FindClusters() with algorithm = 3 & resolution = 1.2 & group.singletons = FALSE). Nucleosome signal and TSS enrichment were calculated according to the standard Signac workflow. The Seurat object was filtered to retain barcodes that met all of the following criteria: 600–7,500 expressed genes, >20% of scATAC-seq reads in peaks, nucleosome signal 0.5–2, and TSS enrichment >2.5. The preprocessed scMultiome-seq stromal-enriched dataset served as a bridge for annotating the scATAC-seq datasets.

To identify TECs among the stromal cells, the dataset was analyzed further. The RNA assay was log-normalized, variable features (*n* = 2,000) were identified using the “vst” method, and scaling was performed, regressing out the number of reads and the number of genes. Next, principal component analysis was performed, followed by UMAP embedding using the first 40 principal components. Neighbors were identified using the first 40 principal components, and clusters were found at resolution 1.2. To annotate the Multiome sample, cell type labels were transferred from the scRNA-seq stromal-enriched dataset, as implemented in the Seurat workflow (FindTransferAnchors(), TransferData(), AddMetadata()).

##### Peak calling, QC, and annotation of the scMultiome-seq TEC dataset

Clusters annotated as TECs were subsetted from the scMultiome-seq stromal-enriched dataset. The ATAC assay was re-clustered at a resolution of 1, and peaks were called for each cluster. The resulting peaks were merged with the initial peak set. A new count matrix and ATAC assay were generated based on the merged peaks, replacing the previous ATAC assay. The new assay was preprocessed as described above for the ATAC assay in the scMultiome-seq stromal-enriched dataset, except that min.cutoff was set to 5 when finding top features. Cell type labels were transferred from the scRNA-seq TECs dataset, expression of established TEC and non-TEC markers was explored, and clusters standing out as non-TECs, or a mix of TECs and non-TECs (putative doublets), were filtered out. In addition, some clusters expressing considerably fewer genes than other clusters, and containing a higher proportion of poor-quality events according to ATAC QC measures, were discarded from the object. Next, the fragment file was updated to include only barcodes present in the Seurat object, using the FilterCells() function. Assays were normalized, scaled (RNA assay only), and dimensionally reduced as described above, except min.cutoff being set to 1 when finding top features in the ATAC assay. Finally, a joint UMAP was constructed (FindMultiModalNeighbors() with reduction.list = list(“pca”, “lsi”), dims.list = list(1:40, 2:50), and modality.weight.name = “RNA.weight”). The annotated scMultiome-seq TEC dataset served as support for the final filtering of the bridge-annotated scATAC-seq TEC dataset.

#### scATAC-seq data processing

Raw sequencing data were processed by the 10*x* Genomics Cell Ranger ATAC pipeline v.1.2.0, with alignment to GRCh38. For donor 2, NovaSeq6000 reads were trimmed to 71 bp and combined with the 71 bp NextSeq reads before running the Cell Ranger ATAC Count pipeline.

##### Peak calling, QC, integration, and annotation of the stromal-enriched scATAC-seq dataset

Fragment files were filtered before MACS2 peak calling, as described for the stromal-enriched scMultiome-seq sample. Read depth cutoff was set for each donor, ranging from 10^2.6^ to 10^2.8^, and peaks were called per donor. Filtered fragment files and the corresponding peak sets were used to create peak count matrices and Seurat objects. The resulting Seurat objects were merged into a single object, which was normalized, dimensionally reduced, and clustered as described for the scMultiome-seq stromal-enriched ATAC data. The resulting UMAP indicated batch effects from donors; thus, data from the five donors were integrated. Before integration, QC and filtering of poor-quality barcodes were performed on the merged object, with QC measures calculated according to the Signac workflow. Resulting measures were visualized both per donor and per cluster, leading to an initial filtering to keep events meeting the following criteria: Number of peaks >2,000, reads in peaks >20%, blacklisted reads <0.5%, TSS enrichment >2, and nucleosome signal 0.3–2.5. For each donor, the Seurat object was filtered to contain only barcodes present in the filtered merged object, followed by normalization and linear dimensional reduction as described above. Seurat objects were integrated according to the Signac workflow, with reduction = “rlsi” and dims = 2:50 in the FindIntegrationAnchors() function, and dimensions 1–50 used for integrating embeddings. A UMAP was created for the resulting object, with settings as for the merged object. This UMAP contained a cluster mainly composed of events with poorer QC metrics and fewer peaks, compared to other clusters. Events in this cluster were removed based on their peak count, as follows: donor 1: <3,000, donor 2: <4,000, donor 3: <6,500, donor 4: <3,500; donor 5: <3,000.

At this point in the analyses, doublets were predicted by two methods, scDblFinder^167^ and Amulet.^168^ The scDblFinder aims to find heterotypic doublets (consisting of two different cell types), while the Amulet method is made to find both heterotypic and homotypic doublets. For these calculations, we used the R package scDblFinder v.1.11.4, which includes an implementation of the Amulet method (https://bioconductor.org/packages/release/bioc/vignettes/scDblFinder/inst/doc/scATAC.html). We did not filter on the doublet scores due to poor method agreement and the risk of removing biologically relevant events. We did, however, filter out a few events with an extreme number of peaks, using the following cut-offs: donor 1: >30,000; donor 2: >28,000; donor 3: >30,000; donor 4: >35,000; and donor 5: >45,000.

Finally, the scATAC-seq stromal-enriched dataset was annotated with cell types using the bridge integration approach^58^ and Seurat v.4.1.1.9001. The scRNA-seq stromal-enriched dataset was used as the reference, and the preprocessed scMultiome-seq stromal-enriched dataset served as the bridge. The procedure was performed as outlined in the bridge integration vignette (https://satijalab.org/seurat/articles/bridge_integration_vignette.html), following the instructions for custom reference data.

##### Peak calling, QC, integration, and annotation of the scATAC-seq TEC dataset

TEC clusters were extracted from the scATAC-seq stromal-enriched dataset and re-clustered at resolution 1. Peaks were called on each cluster, and the resulting peaks were merged with the peak set from the scATAC-seq stromal-enriched dataset. For each donor, a peak count matrix and a Seurat object were constructed. The five objects were processed and integrated as described for the scATAC-seq stromal-enriched dataset, except for k.weight set to 98 in the IntegrateEmbeddings() function. The resulting object was annotated via bridge integration, mainly as described above for the scATAC-seq stromal-enriched dataset, using the scRNA-seq TEC dataset as the reference. Again, the scMultiome-seq stromal-enriched dataset served as the bridge, with its ATAC assay requantified to contain the peak set of the scATAC-seq TEC object. After annotation, the scATAC-seq TEC object was subject to a thorough filtering process to remove any remaining non-TECs. This process involved assessing peaks in cell type marker regions, performing differential peak accessibility analysis across clusters, and comparing the results with the scMultiome-seq TEC dataset. The FilterCells() function was applied to the fragment files to keep only fragments present in the final object. The donor integration procedure was repeated (as for the scATAC-seq stromal-enriched dataset, except for k.weight set to 84 in IntegrateEmbeddings()), and a UMAP was constructed. The bridge integration was also repeated after the final filtering. The cells were re-clustered at resolution 3.5, and final annotations were set according to the transferred cell type labels, with these exceptions: 1) One cluster that was predicted to consist of several cell types was named Intermediate TECs. 2) A few cells predicted as cTECs with low prediction scores were further validated and annotated as nclTECs. 3) The mcTECs formed three distinct clusters in the scATAC-seq data, and these clusters were named mcTEC_a, mcTEC_b, and mcTEC_c.

#### Analyses of scATAC-seq data

##### Differentially accessible peaks

For Intermediate TECs, differential peak accessibility analysis against remaining TEC clusters was performed using the Seurat FindMarkers() function, with min.pct = 0.25, only.pos = FALSE, and test.use = ‘LR'.

##### Assignment of GO terms based on chromatin accessibility

Genomic Regions Enrichment of Annotations Tool (GREAT)^147^^,^^169^ v.4.0.4 was applied to Intermediate TECs DA peaks with negative average log2 fold change and adjusted *p*-values <0.05. Default settings were used (association rule Basal+extension: 5,000 upstream; 1,000 bp downstream; 1,000,000 bp max extension; curated regulatory domains included).

##### Chromatin accessibility over motifs and segments by chromVAR

Chromatin accessibility over TF motifs and genomic segments was explored using the R package chromVAR^148^ v.1.16.0, according to vignette instructions (https://greenleaflab.github.io/chromVAR/index.html). A chromVAR deviation value tells if a particular cell, or group of cells, is more (positive deviation) or less (negative deviation) accessible in peaks overlapping a given annotation as compared to other cells. A RangedSummarizedExperiment object was made using the peak count matrix of the scATAC-seq TEC dataset, with cell type annotations in colData. For GC content computation, genome was set to BSgenome.Hsapiens.UCSC.hg38 (UCSC version hg38).

For TF motif analyses, the *Cis*-BP database (cisBP_human_pfms_2021 downloaded from https://github.com/buenrostrolab/FigR/tree/master/data) was used for annotation of peaks overlapping with motifs. chromVAR deviations were computed, and the mean deviation *Z* score of each motif was calculated per cluster. To compute differential motif accessibility, an annotation column was added to the deviations object for each cluster, grouping the cell barcodes as belonging to that particular cluster or not, and differential deviations were identified for each cluster versus the remaining TECs, using a one-sided (“greater”) parametric test (implemented in the differentialDeviations() function).

Accessibility was also assessed over additional genomic segments. The R package TxDb.Hsapiens.UCSC.hg38.refGene v.3.13.0 was applied to segment into promoters and genes. Distal enhancers (>2 kb from TSS), proximal enhancers (<2 kb from TSS), and CTCF elements were downloaded from the ENCODE Registry of cCREs^157^ (https://screen.encodeproject.org/index/cversions, at 14/02/2024), TSS annotation from refTSS^158^ (https://reftss.riken.jp/datafiles/current/human/, at 14/02/2024), and super-enhancers from SEdb^159^^,^^170^ (details in Table S5). For genome-wide accessibility, genomic ranges were extracted per chromosome (seqinfo(BSgenome.Hsapiens.UCSC.hg38)). Each segment group (e.g., promoters or distal enhancers) was converted to a genomic ranges list (R package GenomicRanges^149^ v.1.42.0), and used to annotate the peaks in the RangedSummarizedExperiment object by chromVAR’s getAnnotations() function (one annotation per segment group). chromVAR deviations were computed per segment group, and deviation z-scores were visualized in clustered heatmaps.

##### Footprinting

Peaks overlapping distal and proximal enhancers were identified as described in the previous section, and the Signac TECs object was subsetted on each peak set. TF footprinting was performed using the Signac Footprint() function, either on the complete peak set or on peaks overlapping distal or proximal enhancers.

##### Prediction of chromatin contact maps by ChromaFold

ChromaFold^150^ calculates pseudobulk chromatin accessibility and co-accessibility from scATAC-seq data, and uses these calculations, together with predicted CTCF motif tracks, as input to model chromatin contact maps, similar to those obtained from chromosome conformation capture experiments. The method predicts contacts over genomic distances up to 2 Mb, at 10 Kb resolution. The output score for any two interacting loci reflects their contact frequency in the studied cell population, with a high score corresponding to frequent contact.

Before ChromaFold analyses, one duplicated barcode was removed from the scATAC-seq TECs dataset, fragment files were extracted per donor using Signac FilterCells(), and a joint.csv file containing all barcodes and their corresponding cell types was made (quote = FALSE). ChromaFold computations were run on a high-performance computing cluster, using the Conda environment provided by the ChromaFold developers (with Python 3.8.12). For computations with ArchR^151^ (v.1.0.2), R v.4.1.2 was used. Jupyter Notebook v.7.2.2^152^ was used for visualization, with the same Conda environment except for the following updates: pandas v.1.5, matplotlib^153^ v.3.7.5, and CoolBox^154^ v.0.3.7. Briefly, the ChromaFold workflow includes these steps.

1) Merging fragments into separate files for each cell type, and converting files to sorted bgzip format
2) Preprocess each file using ArchR to get reduced dimensions (LSI file)
3) Calculate tile files and metacells, per cell type
4) Predict chromatin interactions per cell type, per chromosome
5) Visualize the predicted interactions in contact maps (matplotlib), annotate maps with genes and interaction arcs (DnaFeaturesViewer,^171^ CoolBox).

Computations and visualizations were performed according to scripts and instructions at https://github.com/viannegao/ChromaFold, using hg38, inference on chromosomes 1–22 without offset, and with the following changes applied: (I) In script fragment_celltype_merge.py (step 1) argument nrows_skip was set to 0 (line 52), “cell_barcode” in line 66 was changed to “cell_id”, and line 64 was deleted. (II) In scATAC_preparation.py (step 3) n_neighbours was set to 30 in metacell_computing(). To generate.bedpe files for visualizing interactions as arcs, additional code was obtained from the ChromaFold developers.

The Ionocyte-like TEC and mcTEC_c clusters contained too few cells (45 and 88, respectively) to produce meaningful contact maps, inferred to have fewer and weaker contacts than the remaining cell types.

##### Pseudotime analysis by Monocle3

Single-cell trajectories were built for the scATAC-seq TEC dataset as described for the scRNA-seq dataset, with starting nodes in the mcTEC_a and TEC(myo)_a clusters for the graphs encompassing conventional mTECs and mimetic mTECs, respectively.

#### Computationally paired scRNA-seq and scATAC-seq data

To obtain multimodal data at the single-cell resolution, each cell in the scATAC-seq TEC dataset was matched with a cell in the scRNA-seq TEC dataset. First, both datasets were filtered to include only shared cell types, leaving 2,981 and 2,356 cells in the scATAC-seq and scRNA-seq Seurat objects, respectively. Next, normalized per-donor gene activity matrices were computed for the ATAC object, according to standard Signac procedures, followed by integration of the matrices (SelectIntegrationFeatures() with nfeatures = 10,000, FindIntegrationAnchors(), and IntegrateData() with 40 dimensions), identification of variable features, and scaling. The resulting integrated gene activity matrix was used together with the scaled integrated gene expression matrix from the scRNA-seq Seurat object to compute canonical correlation analysis components based on the union of top variable features (Seurat RunCCA() with num.cc = 40), and the components were normalized using Seurat L2CCA(). Finally, these components were used as input to FigR^102^ pairCells(), allowing multiple matches for cells in the smaller dataset. For evaluation, RNA cell type labels were transferred to the ATAC object according to the pairing and visualized on the ATAC Seurat object’s UMAP. This revealed that many mTEC(II) cells from the RNA object were paired with non-mTEC(II) cells from the ATAC object, likely due to the underrepresentation of mTEC(II) in the ATAC object. Hence, these mispaired cells were discarded from the RNA object, and the pairing procedure was repeated, showing good agreement between RNA and ATAC cell type labels upon evaluation.

#### Gene regulatory networks by FigR

A gene regulatory network analysis was performed on the computationally paired scRNA-seq and scATAC-seq TEC datasets (previous section) using FigR,^102^ following the guidelines at https://buenrostrolab.github.io/FigR/index.html. FigR identifies domains of regulatory chromatin (DORCs) and the TFs inferred to regulate these domains. Each DORC is assigned a score for each cell, reflecting the DORC’s accessibility. For the present work, a DORC was defined as a region up to 100 kb centered around a particular gene and containing ≥7 ATAC peaks positively correlated with the gene’s expression, yielding 443 DORCs.

Two-sample Wilcoxon rank-sum tests were performed to test for differences in smoothed DORC accessibility scores between one cell type and the remaining cell types, for each DORC and each cell type. GO analysis of all DORC genes, and of the DORC genes corresponding to the 100 most significantly DA DORCs of mTEC(III)_b (adjusted *p*-values from differential DORC accessibility analysis ≤1.4∗10^−288^), was obtained by g:Profiler.^140^ Classification of DORC genes as TRAs, or non-TRAs, was done by comparison to the list of TRAs from HPA (referred to in section “Tissue restricted antigen expression”).

For identification of DORCs with high residuals (i.e., DNA accessibility minus RNA expression), the following strategy was applied: 1) Residuals for each DORC were calculated for each cell. 2) Per DORC, the mean residual for the cells with the 15 largest residuals was calculated. 3) Per DORC, the mean residual for the cells with the 30 largest residuals was calculated. 4) Per DORC, the mean residual for the cells with the 60 largest residuals was calculated. 5) A list of DORCs with the largest residuals, using the union of the top 100 means from 2), 3), and 4), was obtained. For each of these DORCs, plots of chromatin accessibility and gene expression were inspected to identify DORCs for which chromatin accessibility preceded DORC gene expression along inferred differentiation trajectories.

## Supplemental information

Document S1. Figures S1–S16; Tables S1, S2, S4, S8, S13–S17, S20, and S22; and Data S1

Table S3. DA peaks in the Intermediate TECs cluster, related to Figure 1 The analysis was performed to identify (1) peaks showing increased accessibility (positive avg_log2FC values) and (2) peaks showing decreased accessibility (negative avg_log2FC values) relative to the remaining TEC clusters. No peaks in category (1) were identified.

Table S5. Selected super-enhancers from SEdb, related to Figures 1 and S4

Table S6. DA motif analysis in the mcTEC clusters, related to Figure 1 For each cluster, the analysis was performed to identify motifs showing increased accessibility relative to the remaining TEC clusters. *p* values, motif mean deviation *Z* scores, and measures describing the spread of the deviation *Z* scores across single cells are listed.

Table S7. DE genes between annotated clusters in the scRNA-seq TEC dataset, related to Figure 1 For each cluster, the analysis was performed to identify genes showing increased expression relative to the remaining TEC clusters. For TEC(neuro)_a and TEC(neuro)_b, pairwise comparisons were also performed.

Table S9. Assignment of Biological Process terms for selected clusters in the scRNA-seq TEC dataset, related to Figure 1 For each cluster, all DE genes with an adjusted *p* value <0.05 were used as input to the analysis. For pairwise comparisons between TEC(neuro)_a and TEC(neuro)_b, the 100 most significant DE genes were used.

Table S10. Assignment of Cellular Component terms for selected clusters in the scRNA-seq TEC dataset, related to Figure 1 For each cluster, all DE genes with an adjusted *p* value <0.05 were used as input to the analysis.

Table S11. Assignment of Human Protein Atlas tissue terms for selected clusters in the scRNA-seq TEC dataset, related to Figure 1 For each cluster, all DE genes with an adjusted *p* value <0.05 were used as input to the analysis.

Table S12. SCENIC regulons and mean regulon activities per cell type, related to Figure 4

Table S18. Differential DORC accessibility analysis for annotated clusters in the scATAC-seq TEC dataset, related to Figure 7 *p* values for differential DORC accessibility analysis between TEC clusters, supplemented with mean DORC scores and measures describing the spread of the DORC scores across single cells. For each cell type, the analysis was performed to identify DORCs with increased accessibility relative to the remaining cell types.

Table S19. Assignment of Biological Process terms from all DORC genes, related to Figure 7

Table S21. DORC regulators, related to Figure 7 DORC regulators at an absolute regulation score cutoff of 1.25. DORC names are given in column A and regulator names in row 1. A positive regulation score indicates activation of the DORC, while a negative score indicates repression.

Data S2. This folder contains all raw microscopy image files and their corresponding lookup tables used for visualization in Figures 5 and S9
