# Supplementary material for: Multimodal single-cell profiling identifies nclTECs and links chromatin remodeling to TEC differentiation and self-antigen expression modes
Source: iScience. 2026 Jul 28;29(8):116916. doi: 10.1016/j.isci.2026.116916 (PMC13450229; doi:10.1016/j.isci.2026.116916)
Supplement: Supplementary file 1 [file mmc1.pdf]

iScience, Volume *29*

Supplemental information

Multimodal single-cell profiling identifies ncITECs and links chromatin remodeling to TEC differentiation and self-antigen expression modes

Hanne Sagsveen Hjorthaug, Marte Heimli, Siri Tennebø Flåm, and Benedicte Alexandra Lie

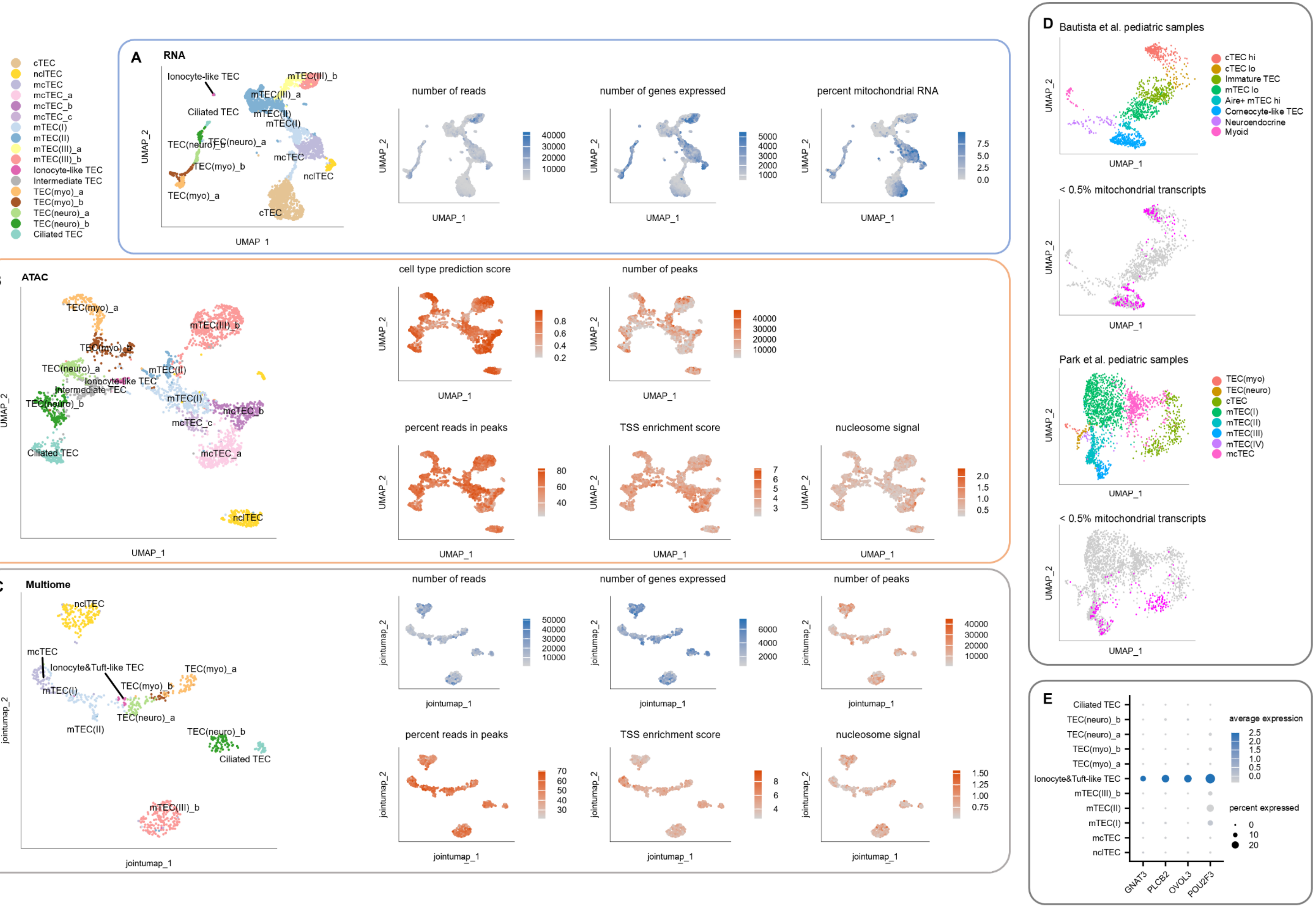
cTEC
nclTEC
mcTEC
mcTEC_a
mcTEC_b
mcTEC_c
mTEC(I)
mTEC(II)
mTEC(III)_a
mTEC(III)_b
Ionocyte-like TEC
Intermediate TEC
TEC(myo)_a
TEC(myo)_b
TEC(neuro)_a
TEC(neuro)_b
Ciliated TEC
A
RNA
Ionocyte-like TEC
mTEC(III)_b
mTEC(III)_a
Ciliated TEC
mTEC(II)
mTEC(I)
TEC(neuro)_b
TEC(neuro)_a
mcTEC
TEC(myo)_b
nclTEC
TEC(myo)_a
cTEC
UMAP_2
UMAP_1
number of reads
40000
30000
20000
10000
number of genes expressed
5000
4000
3000
2000
1000
percent mitochondrial RNA
7.5
5.0
2.5
0.0
B
ATAC
TEC(myo)_a
mTEC(III)_b
TEC(myo)_b
TEC(neuro)_a
mTEC(II)
Ionocyte-like TEC
Intermediate TEC
TEC(neuro)_b
mTEC(I)
mcTEC_b
mcTEC_c
Ciliated TEC
mcTEC_a
nclTEC
cell type prediction score
0.8
0.6
0.4
0.2
number of peaks
40000
30000
20000
10000
percent reads in peaks
80
60
40
TSS enrichment score
7
6
5
4
3
nucleosome signal
2.0
1.5
1.0
0.5
C
Multiome
nclTEC
mcTEC
Ionocyte&Tuft-like TEC
TEC(myo)_a
mTEC(I)
TEC(myo)_b
mTEC(II)
TEC(neuro)_a
TEC(neuro)_b
Ciliated TEC
mTEC(III)_b
jointumap_2
jointumap_1
number of reads
50000
40000
30000
20000
10000
number of genes expressed
6000
4000
2000
number of peaks
40000
30000
20000
10000
percent reads in peaks
70
60
50
40
30
TSS enrichment score
8
6
4
nucleosome signal
1.50
1.25
1.00
0.75
D
Bautista et al. pediatric samples
cTEC hi
cTEC lo
Immature TEC
mTEC lo
Aire+ mTEC hi
Corneocyte-like TEC
Neuroendocrine
Myoid
< 0.5% mitochondrial transcripts
Park et al. pediatric samples
TEC(myo)
TEC(neuro)
cTEC
mTEC(I)
mTEC(II)
mTEC(III)
mTEC(IV)
mcTEC
< 0.5% mitochondrial transcripts
E
Ciliated TEC
TEC(neuro)_b
TEC(neuro)_a
TEC(myo)_b
TEC(myo)_a
Ionocyte&Tuft-like TEC
mTEC(III)_b
mTEC(II)
mTEC(I)
mcTEC
nclTEC
GNAT3
PLCB2
OVOL3
POU2F3
average expression
2.5
2.0
1.5
1.0
0.5
0.0
percent expressed
0
10
20

Figure S1. Quality measures and donor contributions. Related to Figure 1.
(A) scRNA-seq TEC dataset, prepared from freshly isolated cells. Cells with close to zero mitochondrial content correspond to cell types lost/reduced after cryopreservation (B, C), indicating leaky/missing cell membranes upon tissue dissociation.
(B) scATAC-seq TEC dataset, prepared from cryopreserved cells.
(C) scMultiome-seq TEC dataset, prepared from cryopreserved cells.
(D) Pediatric TECs from studies by Bautista et al.[1] and Park et al.[2]. Cells depleted of mitochondrial transcripts (pink) are mainly seen in clusters corresponding to cTEC, mTEC(II), and mTEC(III). Bautista et al. donors: 6 days, 10 months; Park et al. donor: 3 months. Plots were generated using Seurat plotting functions, after converting the datasets into Seurat objects.
(E) In the scMultiome-seq TEC dataset, markers of Tuft-like TECs[3,4] were expressed in the cluster annotated as Ionocyte-like TECs (annotation by label transfer from the scRNA-seq TEC dataset). Accordingly, similarities between Tuft-like and Ionocyte-like TECs have previously been reported[1].

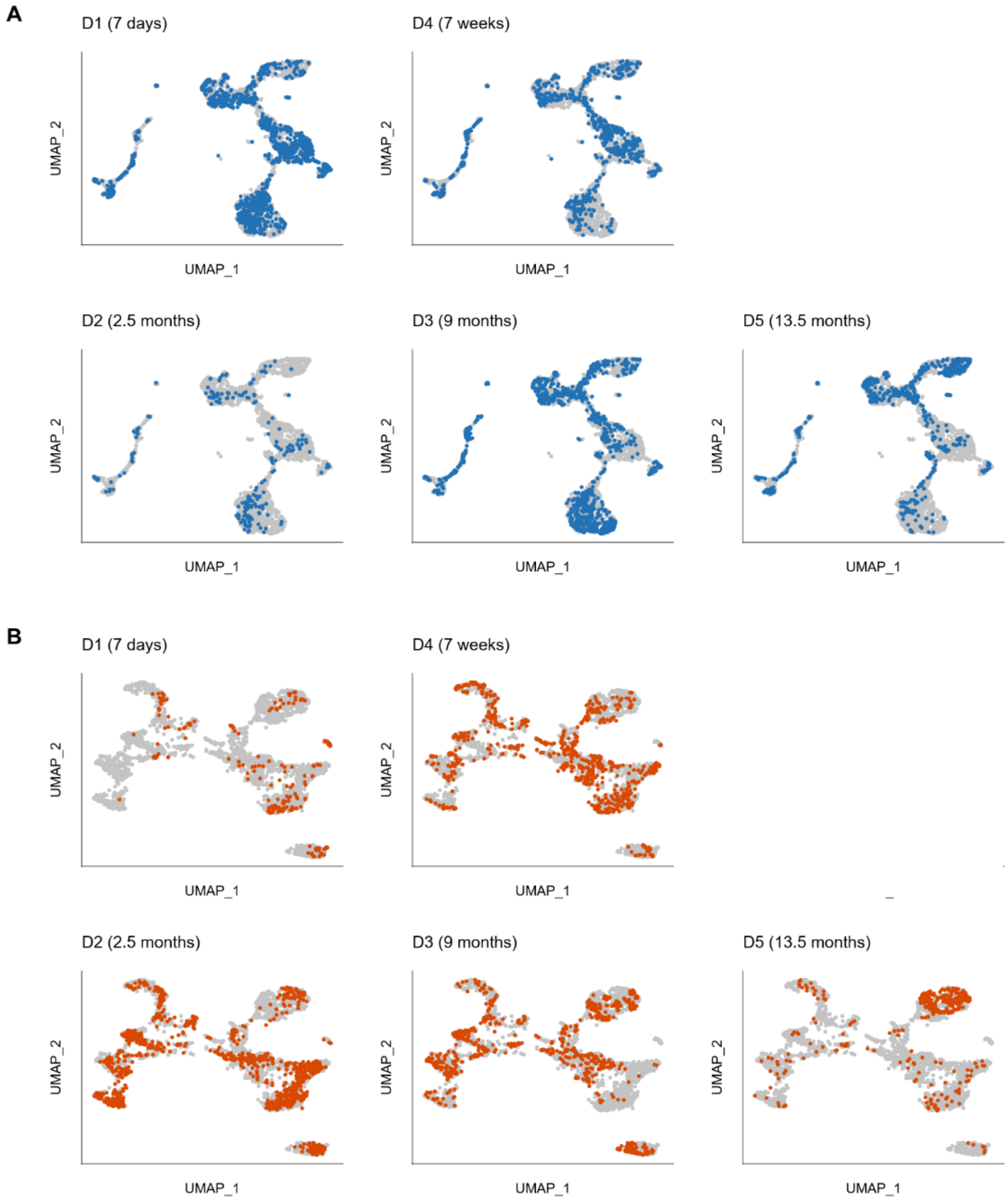

Figure S2. Donor contributions to the single-cell datasets. Related to Figure 1.
(A) Donor contribution to the scRNA-seq TEC dataset.
(B) Donor contribution to the scATAC-seq TEC dataset.

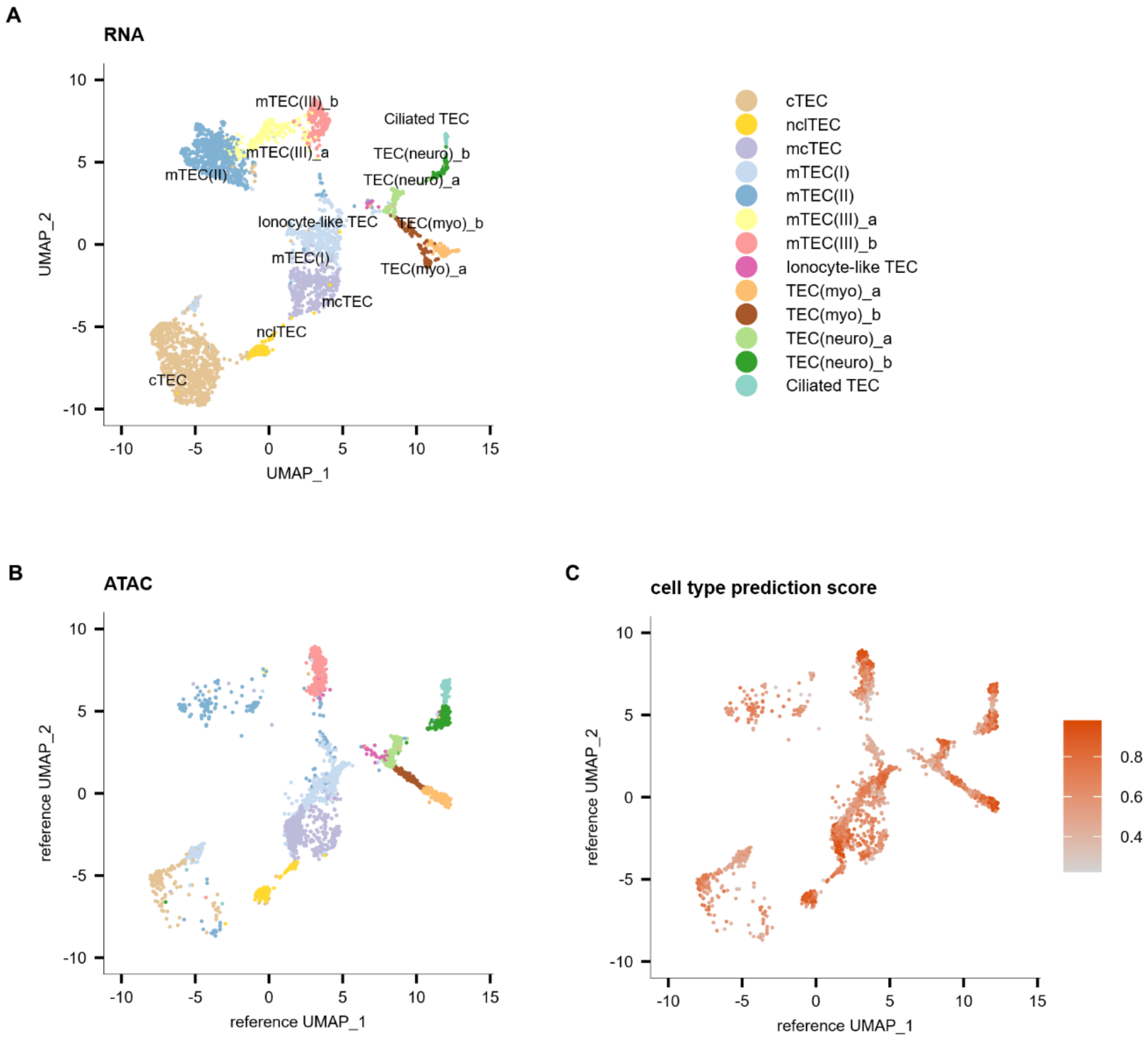

Figure S3. Labelling of cell types in the scATAC-seq TEC dataset by bridge integration. Related to Figure 1.
(A) UMAP embedding generated after applying sctransform on the scRNA-seq TEC dataset. Upon integration, this transformed dataset served as the reference, and the scMultiome-seq stromal-enriched dataset served as the bridge.
(B) Cell type labels in the scATAC-seq TEC dataset after bridge integration, before manual curation. Cells are mapped onto the RNA reference UMAP from (A).
(C) Prediction scores for the cell type labels in (B).

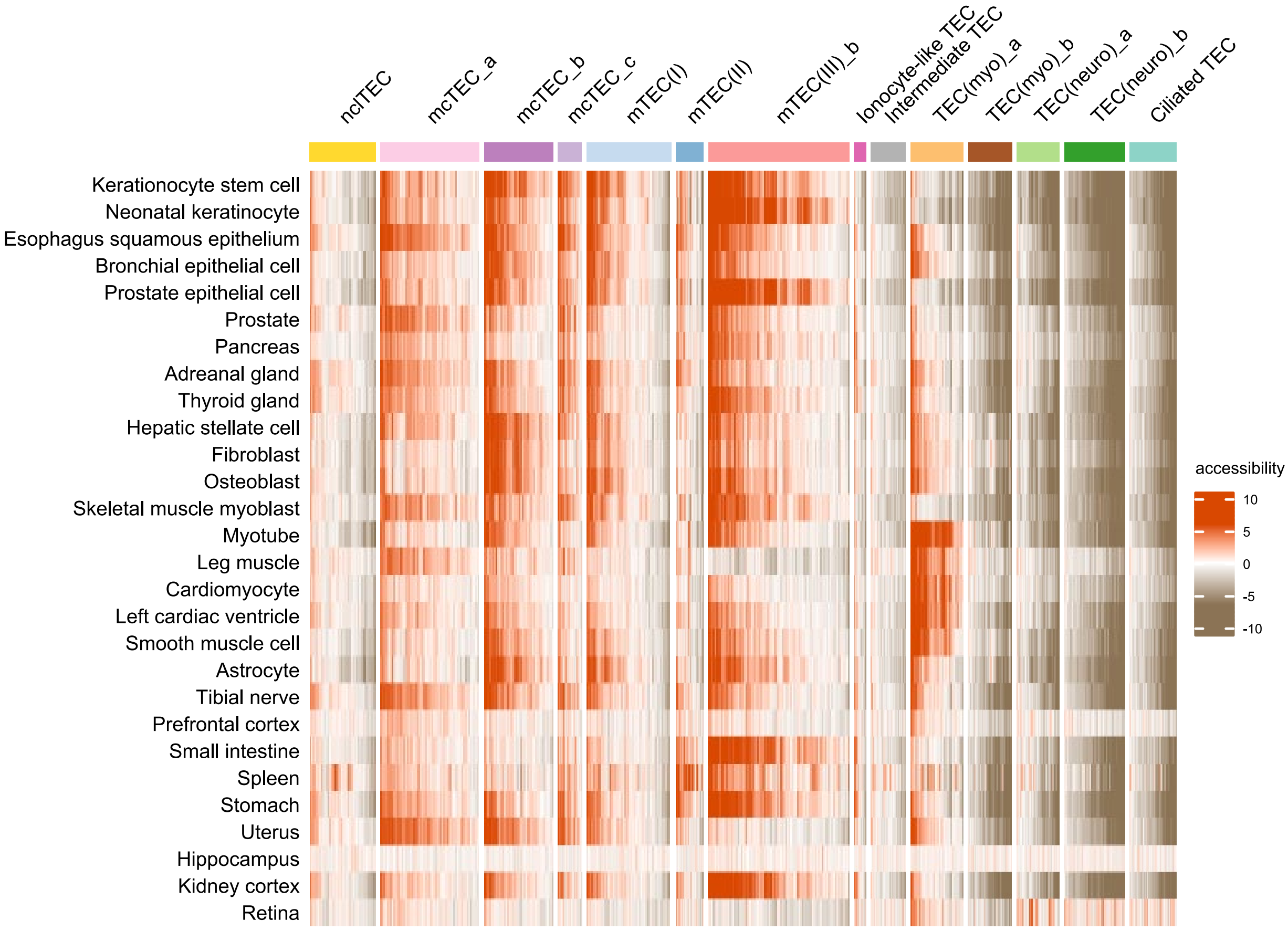

Figure S4. Genome-wide chromatin accessibility across TEC populations over selected peripheral tissue SEs. Related to Figure 1. See Table S5 for details regarding the SEs.

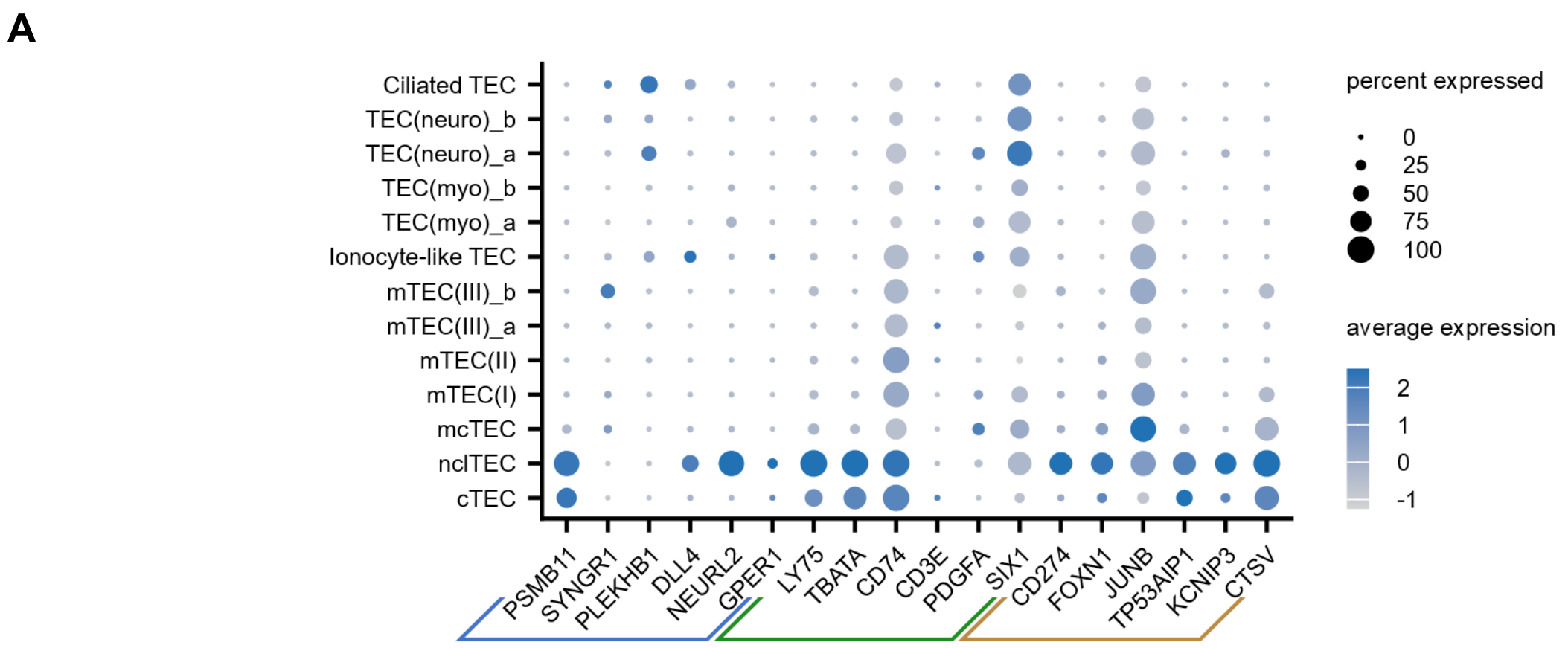

Marker genes from murine perinatal cTECs[5], partly overlapping with our nclTECs.	Markers distinguishing TNCs from other cTECs in newborn and 5-week-old mice[6]. *LY75*, *TBATA*, and *CD74* were among the genes with higher expression in TNCs (as seen for our nclTECs), while *PDGFA* and *SIX1* showed lower expression in TNCs (not seen for our nclTECs). The TNCs reported by Gao et al. expressed *CD3E* and other T cell markers, suggesting that TNCs contained thymocytes in these experiments (not observed for our nclTECs).	cTEC IEGs[7] are reported to express higher levels of *CD274*, *FOXN1* and *JUNB* compared to conventional cTECs (in line with our nclTECs), and lower levels of *TP53AIP1*, *KCNIP3*, and *CTSV* (in contrast to our nclTECs).

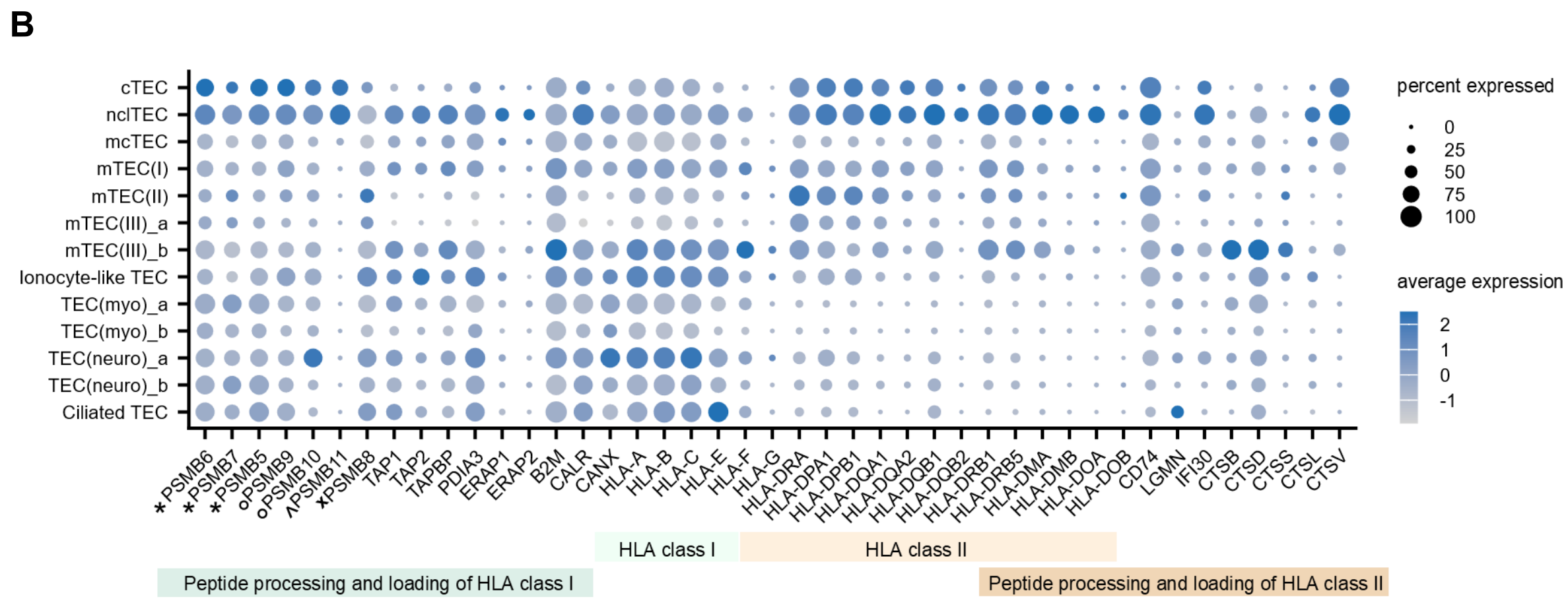

Figure S5. Expression of selected markers across the scRNA-seq TEC dataset. Related to Figure 1.

(A) Markers of previously defined single-cell TEC clusters that partly resemble our nclTECs.

(B) Expression of genes involved in antigen presentation.

* Encoding standard proteasome catalytic subunits β1 (*PSMB6*), β2 (*PSMB7*), β5 (*PSMB5*)

° Encoding immuno- and thymoproteasome catalytic subunits β1i (*PSMB9*), β2i (*PSMB10*)

^ Encoding thymoproteasome catalytic subunit β5t (*PSMB11*)

X Encoding immunoproteasome catalytic subunit β5i (*PSMB8*)

HLA-DRB3 and *HLA-DRB4* were not expressed within the scRNA-seq TEC dataset.

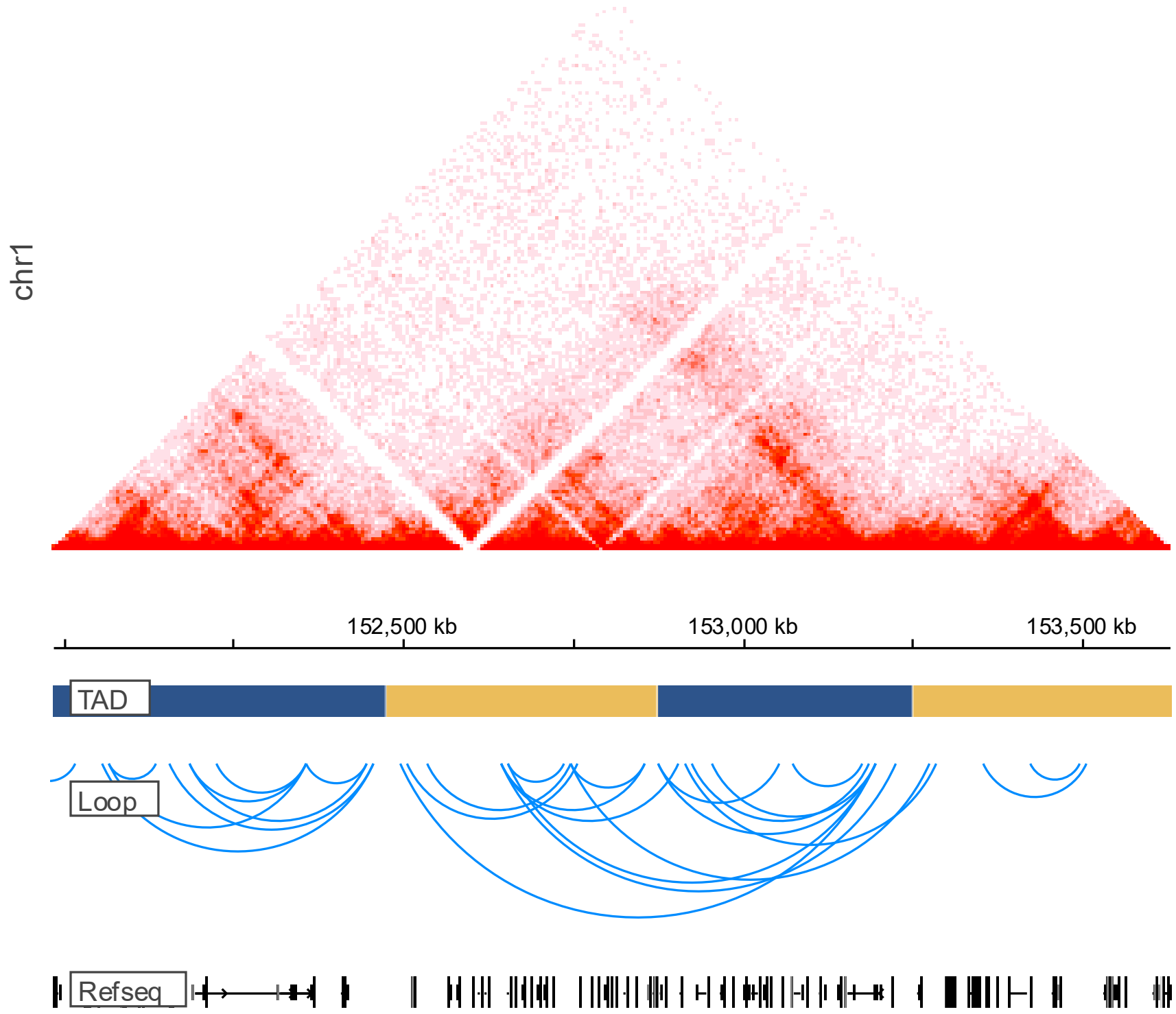

Figure S6. Chromatin contact map of the EDC locus in epidermal keratinocytes. Related to Figure 2. Map, loops, and predicted TADs for chr1:151 982 915-153 632 039, visualized by the 3D Genome Browser 2.0[8], with data from human epidermal keratinocytes[9].

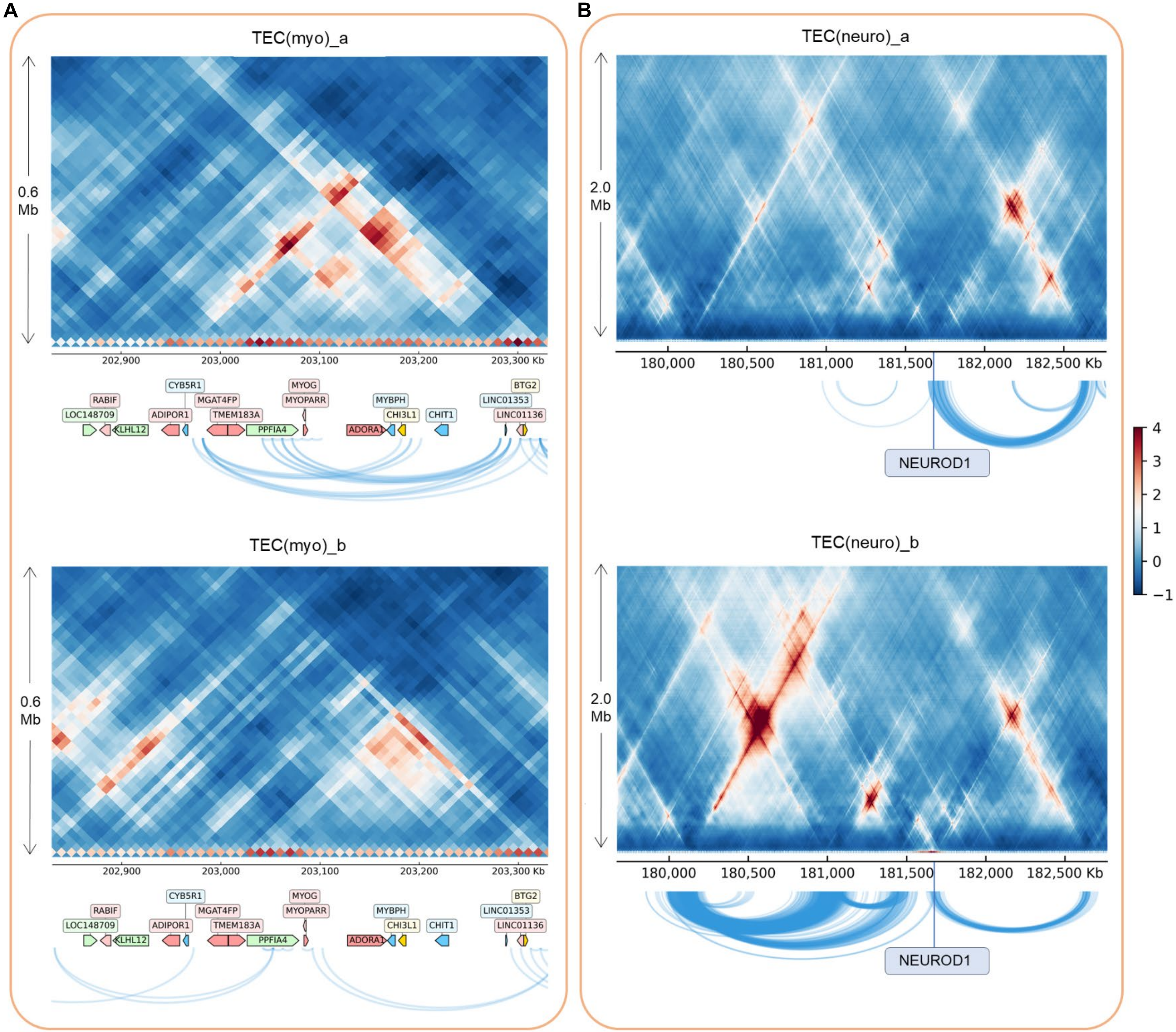

A
TEC(myo)_a
0.6 Mb
202,900
203,000
203,100
203,200
203,300 Kb
CYB5R1
MYOG
BTG2
RABIF
MGAT4FP
MYOPARR
MYBPH
LINC01353
LOC148709
ADIPOR1
TMEM183A
CHI3L1
CHIT1
LINC01136
KLHL12
PPFIA4
ADORA1
TEC(myo)_b
0.6 Mb
B
TEC(neuro)_a
2.0 Mb
180,000
180,500
181,000
181,500
182,000
182,500 Kb
NEUROD1
TEC(neuro)_b
2.0 Mb
4
3
2
1
0
−1

Figure S7. Predicted chromatin contact maps. Related to Figure 2. Predicted interactions are visualized by a blue-white-red color scale where dark blue corresponds to low contact frequencies, and dark red to high contact frequencies. Contacts with scores above 3 are visualized as arcs. Genomic coordinates for the chromosome in question are shown below each map.

(A) Inferred interactions for TEC(myo)_a and TEC(myo)_b in a region on chromosome 1, with *MYOG* in the center.

(B) Inferred interactions for TEC(neuro)_a and TEC(neuro)_b in a region on chromosome 2, encompassing *NEUROD1*.

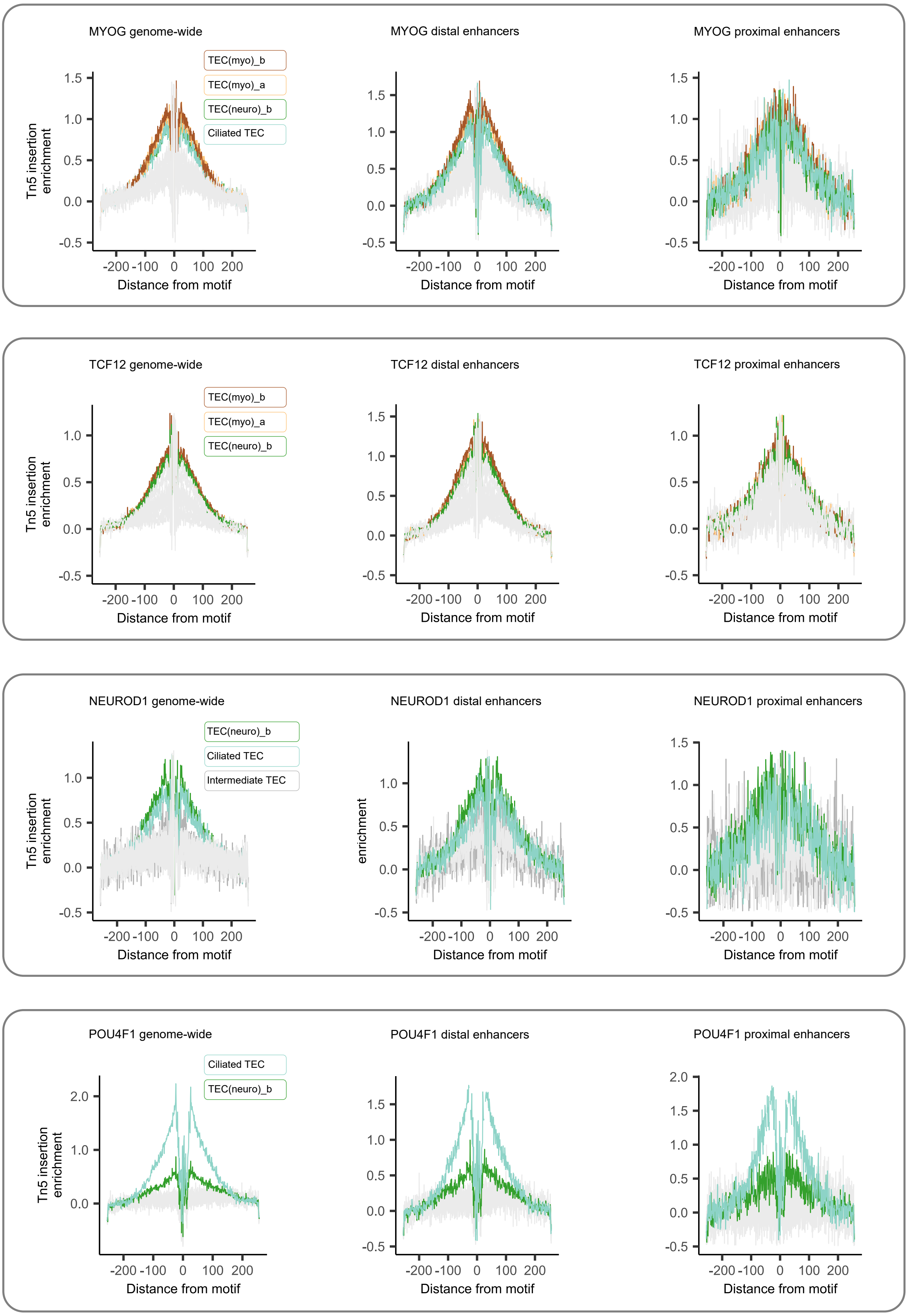

Figure S8. Footprints for selected activators of TRA-enriched SCENIC regulons. Related to Figure 4. Footprints were calculated genome-wide, for distal enhancers, and for proximal enhancers. Cell types with the highest scores in colors, and remaining cell types in gray.

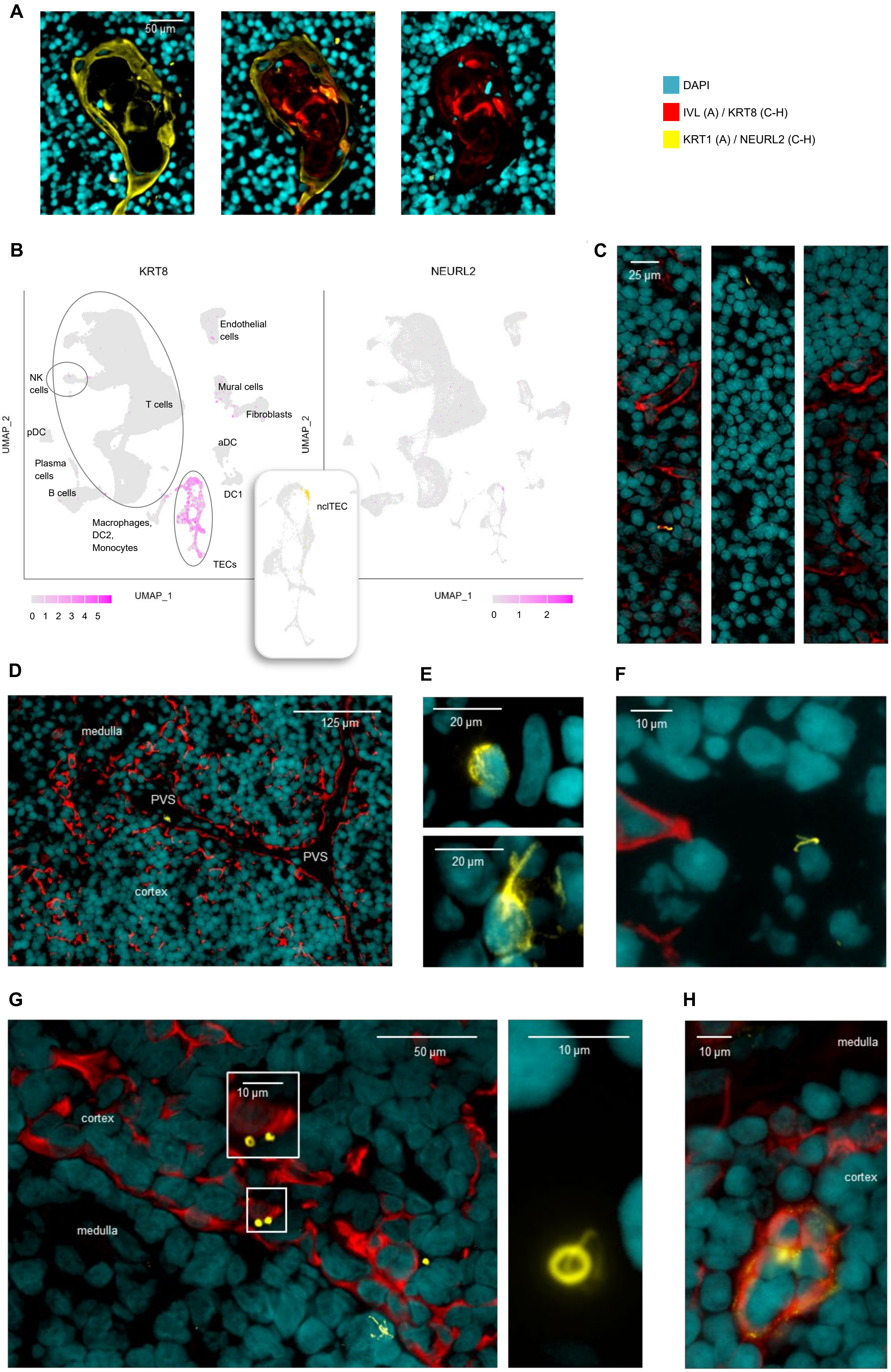

A
50 µm
DAPI
IVL (A) / KRT8 (C-H)
KRT1 (A) / NEURL2 (C-H)
B
KRT8
NEURL2
Endothelial cells
NK cells
T cells
Mural cells
Fibroblasts
pDC
aDC
Plasma cells
B cells
DC1
Macrophages, DC2, Monocytes
TECs
ncITEC
UMAP_2
UMAP_1
0 1 2 3 4 5
0 1 2
C
25 µm
D
medulla
125 µm
PVS
PVS
cortex
E
20 µm
20 µm
F
10 µm
G
50 µm
10 µm
cortex
medulla
10 µm
H
10 µm
medulla
cortex

Figure S9. IF staining controls and additional images related to Figure 5.

(A) Evaluation of antibody specificity and blocking efficiency, showing the same HC in successively cut sections (donor 8, representative results for all donors). Left: Control staining substituting anti-IVL with isotype control. Middle: Double-staining (anti-KRT1 and anti-IVL). Right: Control staining substituting anti-KRT1 with isotype control.

(B) Co-expression of *NEURL2* and *KRT8* was restricted to nclTECs in the full thymic scRNA-seq dataset, and antibodies against their encoded proteins were chosen to identify nclTECs in IF experiments.

(C) Evaluation of antibody specificity and blocking efficiency, donor 6 (representative for all donors). Left: Double-staining (anti-NEURL2 and anti-KRT8). Middle: Control staining, substituting anti-KRT8 with isotype control. No reticular network or TNC-like structures were present. Right: Control staining, substituting anti-NEURL2 with isotype control. The characteristic staining patterns of NEURL2, described in Figure 5, were not seen anywhere in this section.

(D) A NEURL2^{+} cell in the PVS (donor 6).

(E) NEURL2 locating to filament-like structures, presumably extending from, or towards, one side of the cell/nucleus. NEURL2 single-stainings (CMJ, donor 6).

(F) NEURL2 localizing to a lasso-like structure (CMJ, donor 6).

(G) NEURL2 signal appearing as small ring-shaped structures and dots. Left: At the cortical side of the epithelial layer separating cortex and medulla (donor 6). Also, notice the NEURL2-positive filament-like structures surrounding a nucleus on the medullary side. Right: In the CMJ (donor 6).

(H) TNC-like structure close to CMJ (donor 8).

DAPI was used as DNA counterstain for visualization of nuclei. Images are displayed using linear lookup tables. Per-channel grayscale images are available in Document S1.

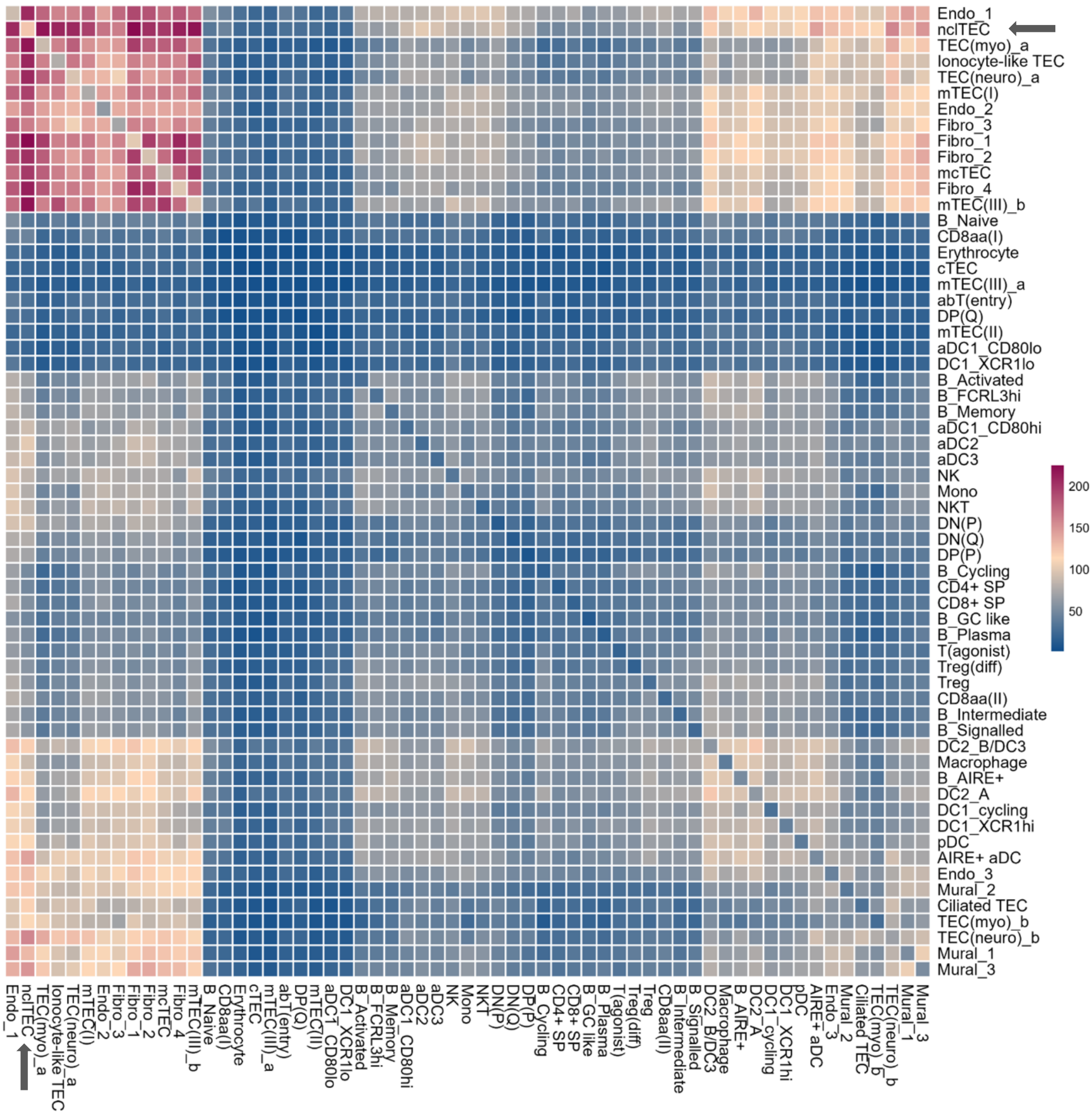

Figure S10. Predicted cell interaction partners in pediatric thymus. Related to Figure 6. For each cell pair, the color represents the number of different significant interactions predicted by CellPhoneDB. Arrows point to nclTEC.

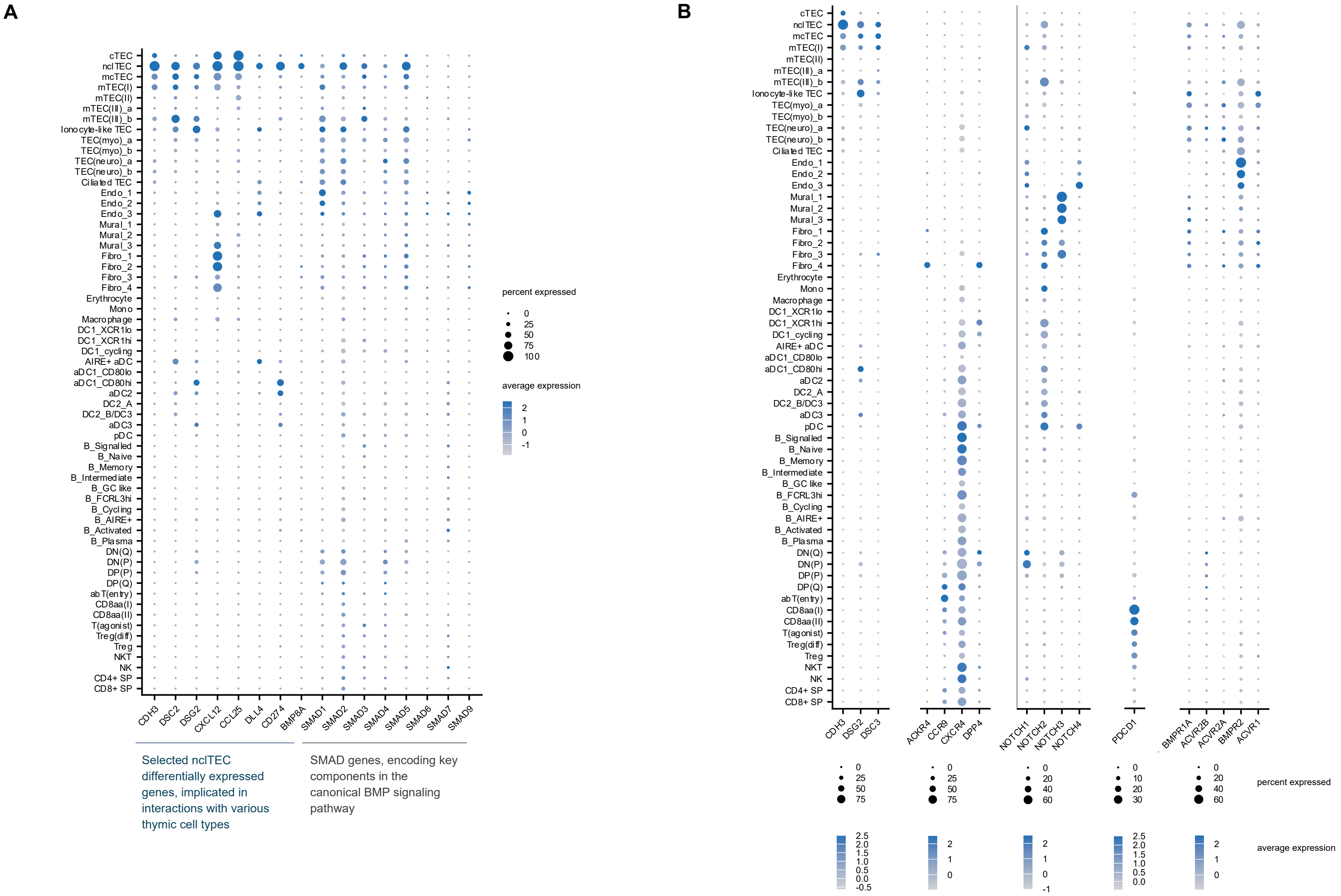

Figure S11. Expression of genes implicated in ncITEC signaling/adhesion. Related to Figure 6.

(A) Expression of genes encoding SMADs and selected ncITEC ligands/adhesion molecules across the full thymic scRNA-seq dataset.

(B) Expression of genes encoding signaling/adhesion partners of ncITEC ligands, assessed across the full thymic scRNA-seq dataset.

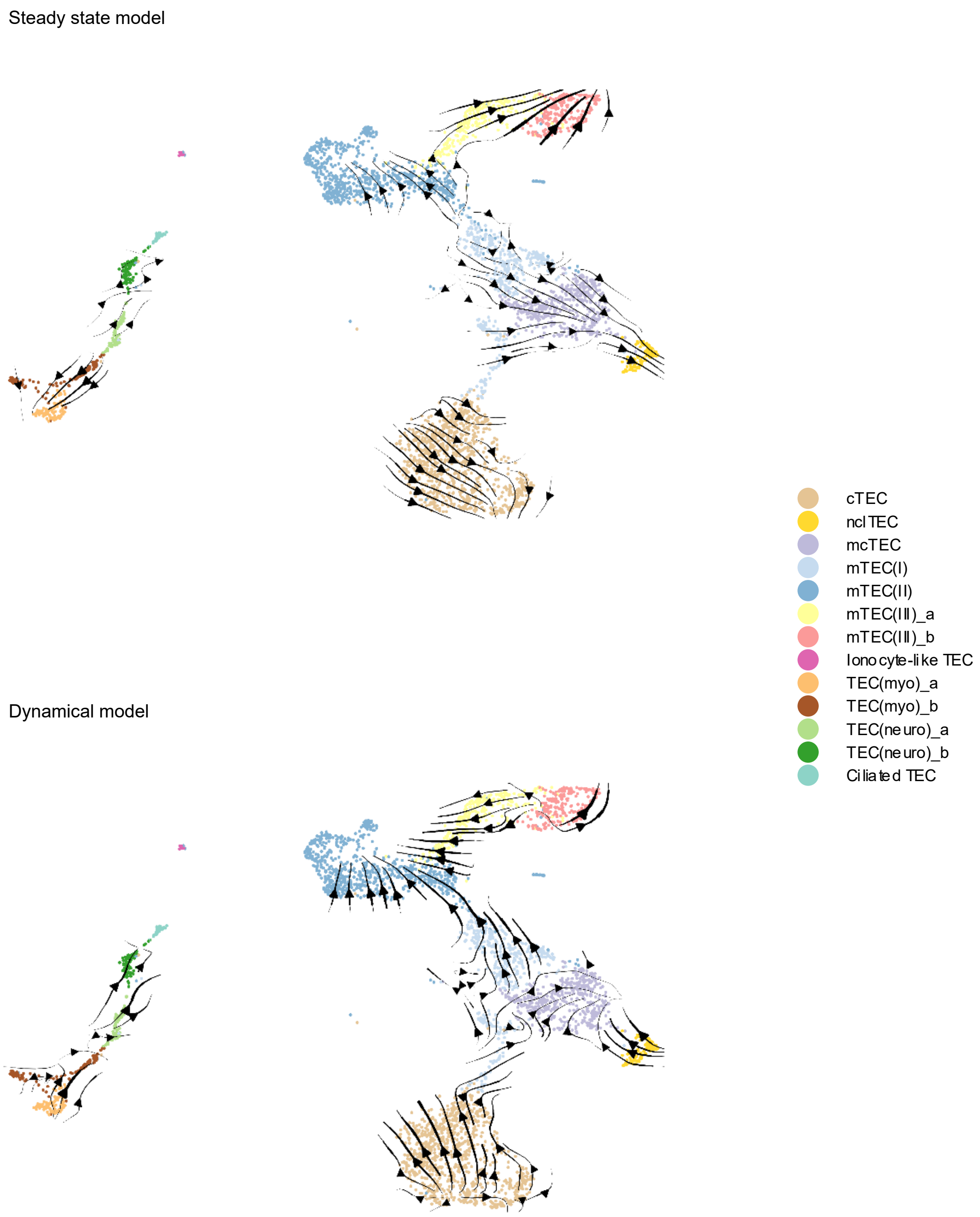

Figure S12. Conflicting RNA velocities in the scRNA-seq TEC dataset. Related to Figure 7. Velocities were calculated using scVelo[10], and both the "steady-state (stochastic)" and "dynamical" models were applied. The resulting velocities were partly conflicting with established routes (e.g., velocities from mTEC(I) to mcTEC in the steady-state model), and the two distinct models yielded considerable differences, e.g., opposite velocity directions between the mcTEC and nclTEC populations.

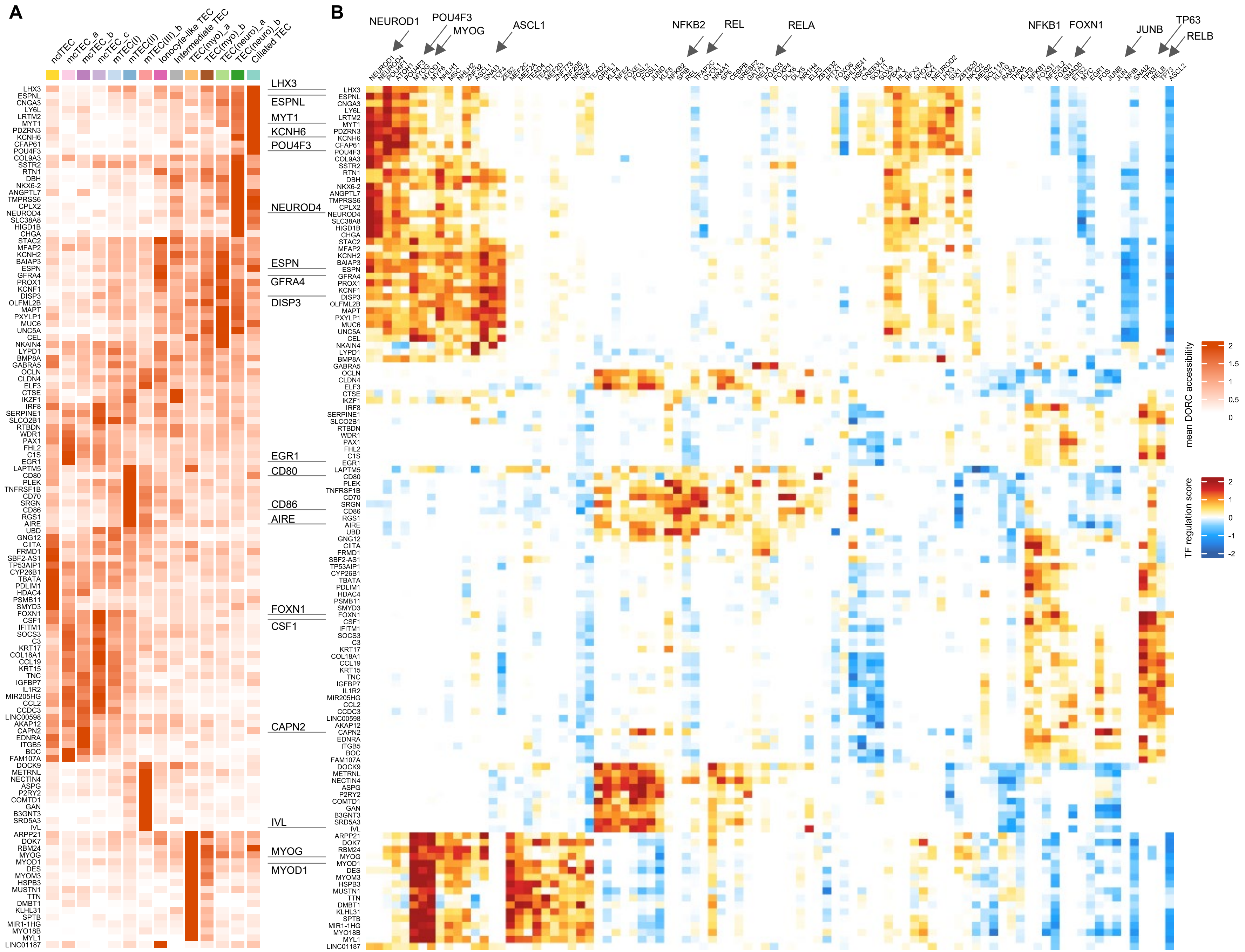

A
B
NEUROD1
POU4F3
MYOG
ASCL1
NFKB2
REL
RELA
NFKB1
FOXN1
JUNB
TP63
RELB
LHX3
ESPNL
MYT1
KCNH6
POU4F3
NEUROD4
ESPN
GFRA4
DISP3
EGR1
CD80
CD86
AIRE
FOXN1
CSF1
CAPN2
IVL
MYOG
MYOD1
mean DORC accessibility
TF regulation score

Figure S13. Regulatory chromatin domains and their regulators across TEC populations. Related to Figure 7.

(A) Top DA DORCs, defined as the union of the top 10 DA DORCs for each cell type. The mcTEC clusters showed differences over several DORCs, including *EGR1* (most accessible in mcTEC_a; EGR1 has reported roles in cell proliferation and differentiation[11,12]), *CAPN2* (most accessible in mcTEC_b; CAPN2 is implicated in e.g. cytoskeletal remodeling[13] and cell proliferation[14]), and genes related to immune cell differentiation and function (e.g., *CSF1*[15], most accessible in mcTEC_c). The DORCs holding *AIRE*, *CD80*, and *CD86* were among the most accessible in mTEC(II), agreeing with expression of established markers[16–18]. In TEC(myo)s, open chromatin was apparent around genes related to muscle development and functions (e.g. *MYOD1*, *MYOG*[19]), particularly in TEC(myo)_a. Ionocyte-like TECs were accessible in e.g. the *ESPN* DORC, encoding a protein with functions in mechanosensory and chemosensory cells[20,21]. *DISP3*, related to neuronal precursors[22,23], was associated with high DORC accessibility in TEC(neuro)_a, while the DORC holding neuronal differentiation factor *NEUROD4* was most accessible in TEC(neuro)_b. Ciliated TECs had an open conformation around genes reported in differentiation and function of ciliated cells (*POU4F3*[24], *ESPNL*[21]), as well as around genes involved in differentiation of oligodendrocytes and neurons (*MYT1*[25,26], *LHX3*[27]).

(B) TFs inferred to regulate the top DA DORCs, with TFs on the x-axis and DORCs on the y-axis. A positive regulation score indicates that the TF activates the DORC gene, while the opposite is true for a negative regulation score. Only TFs with an absolute regulation score above 1.25 are shown in this plot.

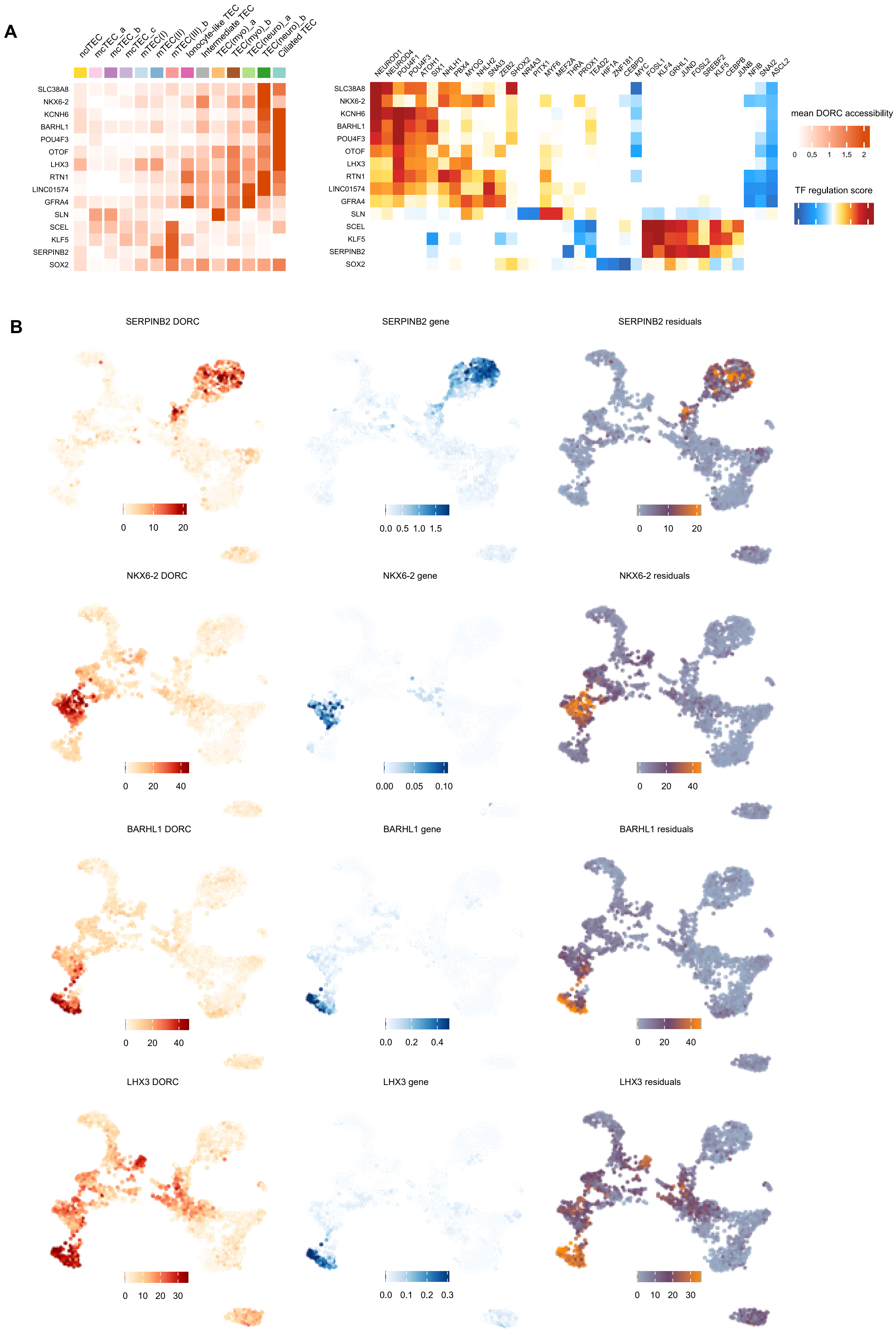
A
ncITEC
mcTEC_a
mcTEC_b
mcTEC_c
mTEC(I)
mTEC(II)
mTEC(III)_b
Ionocyte-like TEC
Intermediate TEC
TEC(myo)_a
TEC(myo)_b
TEC(neuro)_a
TEC(neuro)_b
Ciliated TEC
SLC38A8
NKX6-2
KCNH6
BARHL1
POU4F3
OTOF
LHX3
RTN1
LINC01574
GFRA4
SLN
SCEL
KLF5
SERPINB2
SOX2
NEUROD1
NEUROD4
POU4F1
POU4F3
ATOH1
SIX1
NHLH1
PBX4
MYOG
NHLH2
SNAI3
ZEB2
SHOX2
NR4A3
PITX1
MYF6
MEF2A
THRA
PROX1
TEAD2
HIF1A
ZNF181
CEBPD
MYC
FOSL1
KLF4
GRHL1
JUND
FOSL2
SREBF2
KLF5
CEBPB
JUNB
NFIB
SNAI2
ASCL2
mean DORC accessibility
0
0.5
1
1.5
2
TF regulation score
B
SERPINB2 DORC
SERPINB2 gene
SERPINB2 residuals
NKX6-2 DORC
NKX6-2 gene
NKX6-2 residuals
BARHL1 DORC
BARHL1 gene
BARHL1 residuals
LHX3 DORC
LHX3 gene
LHX3 residuals

Figure S14. Putative lineage-priming in TECs. Related to Figure 7.
(A) Accessibility of putative lineage-primed DORCs across TECs (left), and their inferred TF regulators at regulation score cut-off ±1.25 (right). The *LINC01187* and *CADM1* DORCs were also deduced as primed but did not have inferred regulators at this cut-off.
(B) Accessibility of a selection of DORCs from (A) (left), side-by-side with expression of the corresponding DORC genes in computationally paired cells from the scRNA-seq data (middle) and accessibility minus expression residuals (right). Primed chromatin is indicated by high residuals in cells preceding the gene-expressing cells during TECs differentiation.

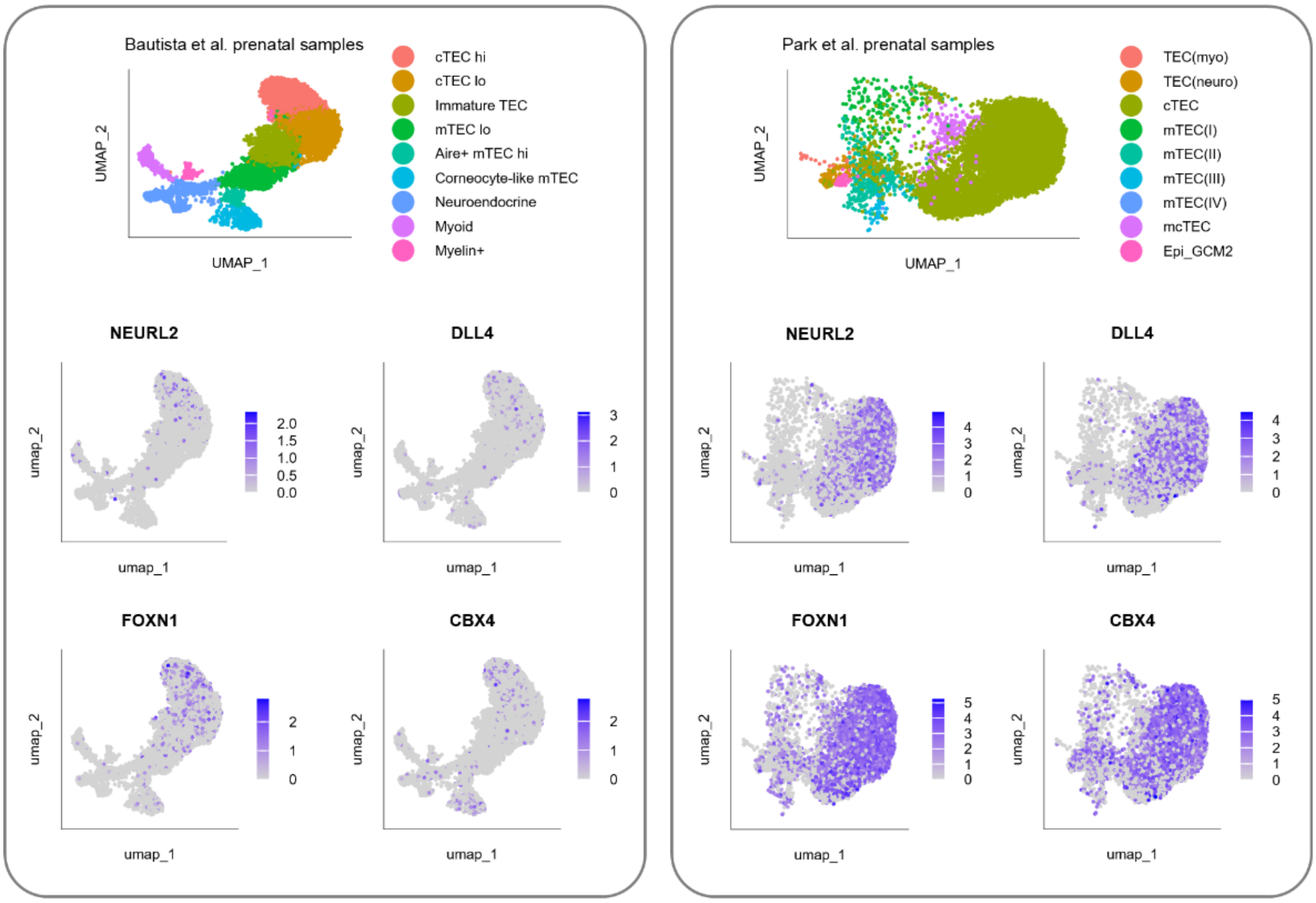

Figure S15. **Expression of ncITEC marker genes in prenatal samples. Related to Figure 8.** Data from studies by Bautista et al.[1] and Park et al.[2]. Bautista et al. donors: 19 post-conception weeks (pcw), 23 pcw; Park et al. donors: 10 donors ranging from 7 to 17 pcw.

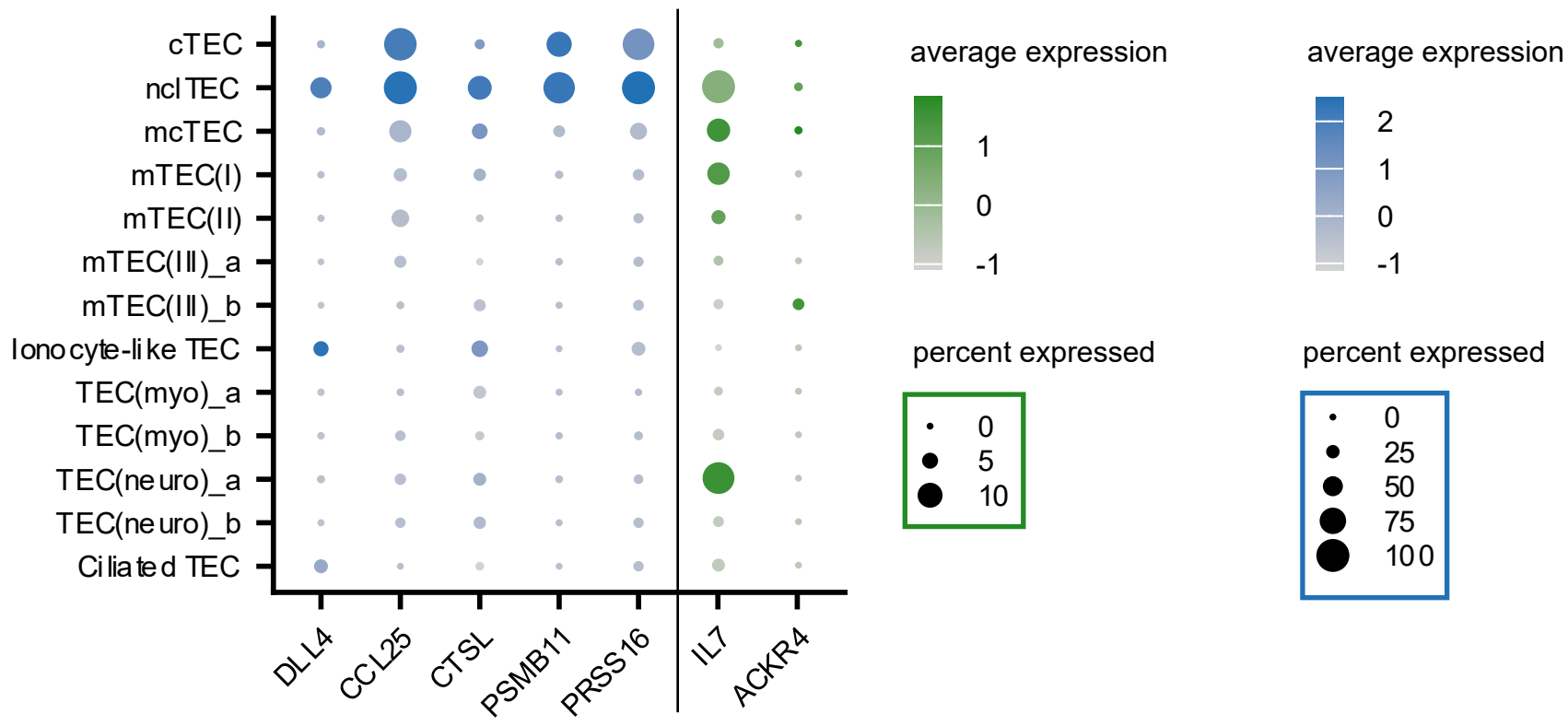

Figure S16. Markers of a previously reported cTEC population giving rise to mTECs in murine embryonic RTOCs[28]. Related to Figure 8. All reported markers, except *ACKR4* (*CCRL1*), were clearly expressed within our nclTEC population.

Table S1. Donor metadata. Related to Figure 1.

Donor	Sex	Age	Application
D1	Male	7 days	Single-cell sequencing
D2	Male	2.5 months	Single-cell sequencing
D3	Female	9 months	Single-cell sequencing
D4	Female	7 weeks	Single-cell sequencing
D5	Male	13.5 months	Single-cell sequencing
D6	Male	10 days	Immunofluorescence
D7	Male	4 months	Immunofluorescence
D8	Female	12 months	Immunofluorescence

Table S2. Datasets used in the present study. Related to Figure 1.

Data type	scRNA-seq			scATAC-seq		scMultiome-seq	
**Dataset**	Full thymic dataset*	scRNA-seq stromal-enriched dataset**	scRNA-seq TEC dataset	scATAC-seq stromal-enriched dataset	scATAC-seq TEC dataset	scMultiome-seq stromal-enriched dataset	scMultiome-seq TEC dataset
**Donors and sequencing libraries**	Five donors, three libraries each	Five donors, one library each		Five donors, one library each		Five donors combined into one library	
**In-silico subsetting**			TECs subsetted from the scRNA-seq stromal-enriched dataset		TECs subsetted from the scATAC-seq stromal-enriched dataset		TECs subsetted from the scMultiome-seq stromal-enriched dataset
**Number of cells**	82,168	20,097	3,508	30,276	2,981	6,704	755

* This dataset equals the scRNA-seq modality of our previously integrated CITE-seq data from unenriched, antigen-presenting-cells-enriched, and CD45-depleted thymic cell samples[29]
** This dataset equals the scRNA-seq modality of our previously published CITE-seq data from CD45-depleted thymic cell samples[29]

Table S4. Assignment of Biological Process terms from peaks underrepresented in the Intermediate TEC cluster. Related to Figure 1. Differentially accessible peaks with negative average log2 fold change and adjusted p-value < 0.05 were used as input to the analysis. The test set of 107 genomic regions picked 181 genes (1%) of all 18,777 genes.

GO-term name	Binom Rank	Binom Raw P-Value	Binom FDR Q-Val	Binom Fold Enrichment	Binom Observed Region Hits	Binom Region Set Coverage	Hyper Rank	Hyper FDR Q-Val	Hyper Fold Enrichment	Hyper Observed Gene Hits	Hyper Total Genes	Hyper Gene Set Coverage
epidermis development	3	2.31E-09	1.02E-05	5.76	18	0.17	33	0.014	3.62	14	401	0.077
hemidesmosome assembly	4	1.37E-08	4.52E-05	69.56	5	0.05	14	0.002	37.72	4	11	0.022
epithelial cell differentiation	9	5.21E-07	7.61E-04	3.34	22	0.21	38	0.020	2.88	18	648	0.099
cell junction organization	12	1.47E-06	1.61E-03	4.78	14	0.13	10	0.001	6.38	12	195	0.066
tissue development	13	1.53E-06	1.55E-03	2.09	40	0.37	7	0.000	2.43	38	1625	0.210
cell-substrate junction assembly	15	3.09E-06	2.71E-03	15.39	6	0.06	36	0.016	12.65	5	41	0.028
epithelium development	18	7.78E-06	5.68E-03	2.35	29	0.27	15	0.003	2.67	27	1050	0.149

Table S8. ncITEC differentially expressed genes with reported roles in stem cells or progenitors. Related to Figure 1.

Gene	Differential expression in the ncITEC cluster (Table S7)				Function of the encoded protein, or of a protein homolog, in relation to progenitors/stem cells	Reported interaction with members of the Neuralized Homology Repeats family
	avg log2 FC	pct.1	pct.2	p_val_adj		
CBX4	0.86	0.71	0.11	1E-67	Part of the p63-regulated program that establishes and maintains epithelial cell fate in epidermal progenitors[30]	
					Maintains human epidermal stem cells as slow-cycling and undifferentiated[31]	
					Cbx4-deficient TECs exhibit impaired proliferative capacity[32]	
CDH3	1.92	0.98	0.26	2E-67	Role in hair follicle growth[33]	
					Colocalize with proliferation marker Ki-67 in human adult epithelia[34]	
DIAPH2	0.86	0.78	0.08	2E-104	Required for cytokinesis, localizes to the midbody[35]	
					Increased expression during mitosis[36]	
					Implicated in actin and microtubule dynamics during cell division[37]	
DLL4	1.00	0.55	0.04	5E-109	Drosophila melanogaster homolog Delta is involved in asymmetric epithelial cell division through Notch-signaling[38]	Neur (NEURL2 homolog) mediates ubiquitinylation of Delta (DLL4 homolog) and subsequent Notch activation during asymmetric cell division in Drosophila melanogaster sensory organ precursor cells[38]
EGFR	0.75	0.88	0.21	2E-42	Role in epidermal stem cell proliferation[39]	

ETNK1	2.52	1.00	0.27	8E-74	Kinase specific and required for the synthesis of phosphatidylethanolamine, a compound with emerging roles in stem cell pluripotency and protection from senescence[40-43]	
FLCN	0.62	0.52	0.06	1E-57	Regulates exit from pluripotency in embryonic stem cells[44]	
FOXN1	0.70	0.78	0.18	4E-38	Essential for pre- and postnatal TECs development and differentiatiation[45–48]	
HDAC4	1.08	0.79	0.06	1E-135	Expressed in the proliferative, undifferentiated cell compartment in the normal intestinal epithelium[49]	
					Repress differentiation in muscle and bone[50,51]	
MPP5 (PALS1)	0.68	0.74	0.15	1E-44	Regulates neuronal progenitor cell fate and cell survival[52]	Neur (NEURL2 homolog) promotes endocytosis of the apical polarity complex Crumbs via its member Stardust (MPP5 homolog) during epithelial remodeling in Drosophila melanogaster embryo[53]
NEURL2	2.00	0.95	0.04	7E-300	Ortholog Neur is involved in asymmetric cell division in Drosophila melanogaster sensory organ precursor cells[38]	
OXCT1	1.59	0.96	0.26	3.9E-55	Synthesis of extrahepatic ketones, with emerging effects on SCs[54–57]	
OXCT2	0.97	0.72	0.09	8.9E-73		
STARD9	1.79	0.88	0.04	9E-258	Regulator of mitotic spindle pole assembly, essential for mitosis[58,59]	
TP63	0.63	0.74	0.18	1.3E-29	Regulates Cbx4 to establish and maintain epithelial cell fate in epidermal progenitors[30]	
					Essential for the proliferative potential of stem cells in stratified epithelia[60]	

Table S13. Results from TRA-enrichment test per SCENIC regulon. Related to Figure 4.
Regulons enriched in TRAs in green and regulons deprived of TRAs in blue.

Regulon	Regulon length	Total TRA count	Total TRA pct	p-value	p-value adjusted
NFE2L1 (12g)	12	12	100.0	1.00E-06	1,17E-04
SNAI3 (22g)	22	20	90.9	1.00E-06	1,17E-04
MYF6 (207g)	207	182	87.9	1.50E-33	1,76E-31
NEUROD1 (16g)	16	14	87.5	1.00E-04	1,17E-02
TFAP2C (16g)	16	14	87.5	1.00E-04	1,17E-02
RARG (39g)	39	34	87.2	8.00E-08	9,36E-06
SOX2 (14g)	14	12	85.7	9.00E-04	1,05E-01
GATA3 (99g)	99	84	84.9	2.50E-16	2,93E-14
MEF2C (118g)	118	99	83.9	8.00E-19	9,36E-17
HSF4 (12g)	12	10	83.3	2.00E-03	2,34E-01
RUNX2 (12g)	12	10	83.3	2.00E-03	2,34E-01
TEAD4 (165g)	165	137	83.0	3.00E-25	3,51E-23
KLF7 (23g)	23	19	82.6	7.00E-05	8,19E-03
ETS1 (11g)	11	9	81.8	3.00E-03	3,51E-01
HOXB13 (59g)	59	48	81.4	9.00E-10	1,05E-07
NR2F2 (21g)	21	17	81.0	2.00E-04	2,34E-02
TCF3 (14g)	14	11	78.6	2.00E-03	2,34E-01
TFAP2A (89g)	89	69	77.5	2.00E-12	2,34E-10
SRF (61g)	61	46	75.4	2.00E-08	2,34E-06
NR1H3 (12g)	12	9	75.0	1.00E-02	1,00E+00
POU4F1 (52g)	52	39	75.0	3.00E-07	3,51E-05
RARA (16g)	16	12	75.0	3.00E-03	3,51E-01
SOX15 (24g)	24	18	75.0	4.00E-04	4,68E-02
POU4F3 (46g)	46	34	73.9	2.00E-06	2,34E-04
SPIC (46g)	46	34	73.9	2.00E-06	2,34E-04
KLF2 (29g)	29	21	72.4	2.00E-04	2,34E-02
ZBTB7A (17g)	17	12	70.6	7.00E-03	8,19E-01
SPIB (181g)	181	127	70.2	7.00E-18	8,19E-16
RXRA (20g)	20	14	70.0	4.00E-03	4,68E-01
POU2F3 (33g)	33	23	69.7	3.00E-04	3,51E-02
MEF2A (293g)	293	203	69.3	4.00E-27	4,68E-25
NFATC2 (13g)	13	9	69.2	2.00E-02	1,00E+00
SOX4 (45g)	45	31	68.9	3.00E-05	3,51E-03
NFIB (32g)	32	22	68.8	5.00E-04	5,85E-02
ZNF524 (22g)	22	15	68.2	4.00E-03	4,68E-01
ATOH1 (12g)	12	8	66.7	4.00E-02	1,00E+00
PBX1 (18g)	18	12	66.7	1.00E-02	1,00E+00

THRA (43g)	43	28	65.1	3.00E-04	3,51E-02
CEBPB (63g)	63	41	65.1	1.00E-05	1,17E-03
MYOG (483g)	483	314	65.0	4.00E-35	4,68E-33
ZNF300 (14g)	14	9	64.3	4.00E-02	1,00E+00
BACH1 (36g)	36	23	63.9	1.00E-03	1,17E-01
ASCL1 (404g)	404	252	62.4	8.00E-25	9,36E-23
SOX11 (92g)	92	57	62.0	2.00E-06	2,34E-04
CREB3L1 (13g)	13	8	61.5	7.00E-02	1,00E+00
MYOD1 (566g)	566	343	60.6	1.00E-30	1,17E-28
FOSL1 (307g)	307	186	60.6	9.00E-17	1,05E-14
POU2F1 (15g)	15	9	60.0	7.00E-02	1,00E+00
TCF7L1 (44g)	44	26	59.1	3.00E-03	3,51E-01
MAX (12g)	12	7	58.3	2.00E-01	1,00E+00
TCF12 (74g)	74	43	58.1	3.00E-04	3,51E-02
GRHL1 (444g)	444	257	57.9	2.00E-19	2,34E-17
ELK3 (66g)	66	38	57.6	8.00E-04	9,36E-02
RFX5 (14g)	14	8	57.1	1,00E+00	1,00E+00
SOX9 (63g)	63	36	57.1	1.00E-03	1,17E-01
IRF8 (106g)	106	59	55.7	1.00E-04	1,17E-02
MEF2D (261g)	261	145	55.6	1.00E-09	1,17E-07
FOSL2 (368g)	368	201	54.6	5.00E-12	5,85E-10
ZBTB43 (13g)	13	7	53.9	1,00E+00	1,00E+00
RFX2 (193g)	193	102	52.9	1.00E-05	1,17E-03
IRF3 (21g)	21	11	52.4	1,00E+00	1,00E+00
REL (288g)	288	150	52.1	2.00E-07	2,34E-05
TGIF2 (33g)	33	17	51.5	1,00E+00	1,00E+00
SP6 (460g)	460	236	51.3	2.00E-10	2,34E-08
KLF3 (333g)	333	170	51.1	1.00E-07	1,17E-05
USF2 (14g)	14	7	50.0	1,00E+00	1,00E+00
IRF7 (131g)	131	64	48.9	6.00E-03	7,02E-01
STAT1 (31g)	31	15	48.4	1,00E+00	1,00E+00
HES1 (19g)	19	9	47.4	1,00E+00	1,00E+00
ZNF460 (13g)	13	6	46.2	1,00E+00	1,00E+00
SP1 (46g)	46	21	45.7	1,00E+00	1,00E+00
MTF1 (11g)	11	5	45.5	1,00E+00	1,00E+00
PKNOX1 (91g)	91	40	44.0	1,00E+00	1,00E+00
ELF5 (780g)	780	339	43.5	2.00E-04	2,34E-02
CUX1 (19g)	19	8	42.1	1,00E+00	1,00E+00
CREB3L2 (131g)	131	55	42.0	1,00E+00	1,00E+00
ZNF528 (55g)	55	23	41.8	1,00E+00	1,00E+00

CEBPG (48g)	48	20	41.7	1,00E+00	1,00E+00
ERF (36g)	36	15	41.7	1,00E+00	1,00E+00
RELB (1073g)	1073	416	38.8	1,00E+00	1,00E+00
ATF6 (13g)	13	5	38.5	1,00E+00	1,00E+00
CREB3 (13g)	13	5	38.5	1,00E+00	1,00E+00
SP2 (429g)	429	165	38.5	1,00E+00	1,00E+00
KLF13 (718g)	718	276	38.4	1,00E+00	1,00E+00
JUNB (84g)	84	32	38.1	1,00E+00	1,00E+00
ZNF224 (11g)	11	4	36.4	1,00E+00	1,00E+00
ZNF23 (14g)	14	5	35.7	1,00E+00	1,00E+00
NFKB2 (700g)	700	247	35.3	1,00E+00	1,00E+00
NR2C1 (23g)	23	8	34.8	1,00E+00	1,00E+00
SREBF2 (333g)	333	114	34.2	1,00E+00	1,00E+00
IRF1 (79g)	79	27	34.2	1,00E+00	1,00E+00
NFE2L2 (83g)	83	28	33.7	1,00E+00	1,00E+00
FOXO3 (15g)	15	5	33.3	1,00E+00	1,00E+00
JUND (287g)	287	90	31.4	4.00E-02	1,00E+00
CLOCK (261g)	261	79	30.3	2.00E-02	1,00E+00
SREBF1 (149g)	149	45	30.2	1,00E+00	1,00E+00
E2F3 (17g)	17	5	29.4	1,00E+00	1,00E+00
ATF3 (390g)	390	111	28.5	3.00E-04	3,51E-02
IRF9 (32g)	32	9	28.1	1,00E+00	1,00E+00
MEIS2 (25g)	25	7	28.0	1,00E+00	1,00E+00
ETV6 (1840g)	1840	514	27.9	8.39E-21	9,81E-19
ATF4 (86g)	86	24	27.9	7.00E-02	1,00E+00
BHLHE41 (239g)	239	65	27.2	1.00E-03	1,17E-01
ELF3 (1161g)	1161	314	27.1	1.20E-14	1,40E-12
NFKB1 (2661g)	2661	719	27.0	6.06E-40	7,09E-38
ELK1 (181g)	181	47	26.0	2.00E-03	2,34E-01
ELF1 (3551g)	3551	910	25.6	1.16E-79	1,35E-77
FOSB (24g)	24	6	25.0	1,00E+00	1,00E+00
ELF2 (4161g)	4161	1040	25.0	6.85E-118	8,02E-116
XBP1 (210g)	210	50	23.8	6.03E-05	7,05E-03
RELA (301g)	301	68	22.6	1.49E-07	1,74E-05
ETV7 (653g)	653	147	22.5	1.61E-15	1,88E-13
ETS2 (2562g)	2562	557	21.7	2.00E-82	2,34E-80
NR1D2 (12g)	12	2	16.7	1,00E+00	1,00E+00
BPTF (16g)	16	2	12.5	5.00E-02	1,00E+00
YY1 (20g)	20	2	10.0	2.00E-02	1,00E+00
JUN (21g)	21	2	9.5	1.00E-02	1,00E+00

Table S14. Putative mechanisms underlying NEURL2 staining patterns. Related to Figure 5.

Fig.	Observed NEURL2⁺ structure	Putative in vivo structure	Hypothetical link between NEURL2 and putative in-vivo structure	Supporting literature
5G, S9E	Filament-like structures extending from, or towards, one side of the cell/nucleus	Actin, myosin, or actomyosin filaments involved in cellular polarization or cytoskeletal remodeling	NEURL2-mediated ubiquitinylation directing filament assembly/disassembly	- Murine Neurl2 binds to embryonic myosin filaments, marking them for ubiquitinylation and degradation[61]<br>- Alix is a target of Neurl2 and is shown to modulate F-actin remodeling[62]<br>- Alix is required for assembly of the apical actomyosin-tight junction complex in choroid plexus epithelial cells, preserving epithelial polarity[63]
5I, S9F	Lasso-like structure	Actomyosin ring formed to aid in the thinning of the intercellular bridge prior to cytokinesis[64]	1) NEURL2 binds to myosin, serving as a tag for subsequent ubiquitin-dependent degradation of the actomyosin ring<br>2) NEURL2 associates with F-actin-bound ALIX at the actomyosin ring	1) Murine Neurl2 binds to embryonic myosin filaments, marking them for ubiquitinylation and degradation[61]<br>2) Alix is a target of Neurl2 and is shown to modulate F-actin remodeling[62]
5F	Narrow cytoplasmic connection between two nuclei	1) Microtubules in the cytokinetic bridge, or<br>2) endosomal trafficking along these, or<br>3) cytoplasmic accumulation of proteins in the midbody arms	1) NEURL2-mediated ubiquitinylation directing microtubule degradation upon cytokinesis<br>2) NEURL2-mediated ubiquitinylation directing endosomal transport of ESCRT-III and its associated proteins (e.g., ALIX) to the midbody<br>3) Ubiquitin-mediated proteasome degradation mediated by NEURL2	1)Two other E3-ubiquitin ligases (MID1, MID2) are reported to localize to microtubules in the cytokinetic bridge and promote cell division[65]<br>2) ESCRT-III and ALIX are shown to be transported in vesicles along microtubules in the cytokinetic bridge[66]<br>3) Midbody protein dynamics depend on ubiquitin-mediated proteasome degradation[67]
5J, 5K, S9G	Small ring-shaped structures and dots	Extracellular midbody remnants from completed cell divisions	NEURL2-dependent ubiquitinylation involved in the recruitment/assembly/degradation of ESCRT complexes and other midbody proteins, e.g., ALIX	- Midbody protein dynamics depend on ubiquitin-mediated proteasome degradation[67]<br>- ALIX is known to interact with the ESCRT machinery at the midbody[68]
5E	Tiny cytoplasmic spots	Vesicles	NEURL2-mediated ubiquitinylation directing protein degradation or recycling in the endolysosomal system	- Ubiquitinylation is known to direct protein degradation or recycling in the endolysosomal system[69]<br>- In Drosophila melanogaster Neur (NEURL2 homolog) has been observed in vesicles[53,70]<br>- NEURL2-signals could be linked to ALIX, which is involved in several aspects of endosome biology, including sorting of cargo[71]

Table S15. Proportions of cells in each CytoTRACE 2 potency category, per TEC cluster. Related to Figure 7. Absolute cell numbers are given in parentheses.

	Differentiated	Unipotent	Oligopotent	Multipotent
**cTEC**	0.947 (865)	0.044 (40)	0.009 (8)	0.000 (0)
**nclTEC**	0.639 (62)	0.247 (24)	0.113 (11)	0.000 (0)
**mcTEC**	0.012 (5)	0.028 (12)	0.309 (133)	0.652 (281)
**mTEC(I)**	0.141 (59)	0.275 (115)	0.443 (185)	0.141 (59)
**mTEC(II)**	0.976 (735)	0.019 (14)	0.005 (4)	0.000 (0)
**mTEC(III)_a**	0.996 (238)	0.000 (0)	0.004 (1)	0.000 (0)
**mTEC(III)_b**	1.000 (222)	0.000 (0)	0 (0)	0.000 (0)
**Ionocyte-like TEC**	0.737 (14)	0.263 (5)	0 (0)	0.000 (0)
**TEC(myo)_a**	0.044 (4)	0.857 (78)	0.099 (9)	0.000 (0)
**TEC(myo)_b**	0.385 (45)	0.556 (65)	0.060 (7)	0.000 (0)
**TEC(neuro)_a**	0.770 (67)	0.138 (12)	0.092 (8)	0.000 (0)
**TEC(neuro)_b**	0.989 (90)	0.011 (1)	0.000 (0)	0.000 (0)
**Ciliated TEC**	1.000 (30)	0.000 (0)	0.000 (0)	0.000 (0)

Table S16. Results from differential gene expression analysis of differentiated vs unipotent and oligopotent ncITECs (as inferred by CytoTRACE 2). Related to Figure 7.

Genes	p_val	avg_log2FC	pct.1	pct.2	p_val_adj
PRDX5	3.32E-07	0.82	0.97	0.94	8.83E-03
ALDOA	4.21E-07	0.90	0.97	0.94	1.12E-02
ATP5MD	4.37E-07	0.95	0.97	0.91	1.16E-02
PSMB11	1.64E-06	0.82	0.98	0.86	4.37E-02
RPS24	3.26E-06	0.84	1.00	0.97	8.69E-02
COX7A1	4.66E-06	0.84	0.98	0.83	1.24E-01
PSMB6	5.11E-06	0.74	0.98	0.86	1.36E-01
COX4I1	6.11E-06	0.79	0.98	0.86	1.63E-01
ETFB	7.18E-06	0.81	0.98	0.94	1.91E-01
COX5B	1.21E-05	0.65	0.97	0.94	3.22E-01
COX7B	1.37E-05	0.76	0.97	0.77	3.66E-01
PITHD1	1.39E-05	0.70	1.00	0.97	3.70E-01
EIF3K	1.58E-05	0.71	0.97	0.83	4.22E-01
ADRM1	1.88E-05	0.59	0.97	0.86	5.00E-01
PGAM1	2.40E-05	0.69	0.95	0.80	6.39E-01
ATP5MC1	2.41E-05	0.76	0.94	0.74	6.42E-01

Table S17. Results from differential gene expression analysis of oligopotent vs differentiated and unipotent ncITECs (as inferred by CytoTRACE 2). Related to Figure 7.

Genes	p_val	avg_log2FC	pct.1	pct.2	p_val_adj
HES6	1.48E-08	0.38	0.36	0.00	3.95E-04
IL1R2	1.07E-07	1.61	0.55	0.05	2.85E-03
HPF1	2.45E-07	0.79	0.91	0.23	6.52E-03
ECE1	8.27E-07	0.77	0.36	0.01	2.20E-02
LINC01127	9.55E-07	1.34	0.46	0.04	2.54E-02
HAPLN3	9.70E-07	0.71	0.55	0.06	2.58E-02
HRH1	1.09E-06	0.58	0.27	0.00	2.90E-02
AREG	1.09E-06	1.21	0.27	0.00	2.90E-02
CLDN4	1.09E-06	2.02	0.27	0.00	2.90E-02
SCARA3	1.09E-06	0.61	0.27	0.00	2.90E-02
AL589745.1	1.09E-06	0.61	0.27	0.00	2.90E-02
KRT17	1.30E-06	2.82	0.91	0.28	3.46E-02
HIVEP3	1.90E-06	0.63	0.55	0.07	5.05E-02
MIR222HG	2.00E-06	0.58	0.55	0.06	5.34E-02
SIN3B	2.09E-06	0.74	0.82	0.21	5.56E-02
SEPTIN10	2.45E-06	1.24	1.00	0.55	6.53E-02
CRISPLD1	2.72E-06	1.29	0.64	0.11	7.23E-02
STAT2	3.99E-06	1.19	0.82	0.26	1.06E-01
NRXN3	6.03E-06	0.85	0.55	0.07	1.61E-01
ST3GAL1	7.38E-06	0.79	0.36	0.02	1.97E-01
IFITM3	7.43E-06	1.93	0.82	0.24	1.98E-01
RALGAPA1	7.93E-06	0.79	0.82	0.24	2.11E-01
WNT5A	8.39E-06	0.47	0.36	0.02	2.23E-01
NECTIN2	8.39E-06	0.69	0.36	0.02	2.23E-01
SNTB2	9.72E-06	0.96	0.73	0.19	2.59E-01
UNC5C	9.91E-06	0.71	0.46	0.05	2.64E-01
ERRFI1	1.06E-05	1.00	0.64	0.12	2.83E-01
FARP1	1.22E-05	0.58	0.36	0.02	3.26E-01
BACH2	1.26E-05	0.85	0.73	0.16	3.35E-01
IQGAP1	1.28E-05	0.93	0.73	0.19	3.41E-01
BCAM	1.39E-05	0.87	0.36	0.02	3.69E-01
PALLD	2.91E-05	1.22	1.00	0.66	7.75E-01
LY6E	3.11E-05	1.14	0.91	0.36	8.29E-01

Table S20. Biological Process gene set enrichment analysis of the top 100 differentially accessible (DA) DORCs' genes in mTEC(III)_b. Related to Figure 7.

Term name	Term id	High-lighted	p-value adj.	Term size	Query size	Intersection size	Effective domain size	Intersections
Skin development	GO:0043588	true	3.87E-04	327	91	11	21026	IVL,EVPL,KRT78,EPHA2,GJB3,GRHL1,ASPRV1,IL18,SPINK5,TGM1,CLDN4
Regulation of antibacterial peptide production	GO:0002786	true	6.16E-03	7	91	3	21026	EVPL,NOD2,SPINK5
Protein glycosylation	GO:0006486	true	1.41E-02	226	91	8	21026	SRD5A3,B3GNT3,MGAT3,ST3GAL4,NPC1,FUT2,GCNT3,GALNT12
Macromolecule glycosylation	GO:0043413	false	1.41E-02	226	91	8	21026	SRD5A3,B3GNT3,MGAT3,ST3GAL4,NPC1,FUT2,GCNT3,GALNT12
Regulation of antimicrobial peptide production	GO:0002784	false	1.47E-02	9	91	3	21026	EVPL,NOD2,SPINK5
Peptide cross-linking	GO:0018149	true	1.63E-02	29	91	4	21026	IVL,PI3,EVPL,TGM1
Epidermis development	GO:0008544	true	1.97E-02	398	91	10	21026	IVL,EVPL,KRT78,EPHA2,GRHL1,CALML5,IL18,SPINK5,TGM1,CLDN4
Antibacterial peptide production	GO:0002778	false	2.09E-02	10	91	3	21026	EVPL,NOD2,SPINK5
Glycosylation	GO:0070085	false	2.55E-02	245	91	8	21026	SRD5A3,B3GNT3,MGAT3,ST3GAL4,NPC1,FUT2,GCNT3,GALNT12
Antibacterial humoral response	GO:0019731	false	2.99E-02	70	91	5	21026	PI3,EVPL,NOD2,SPINK5,DEFB1
Antimicrobial peptide production	GO:0002775	false	3.81E-02	12	91	3	21026	EVPL,NOD2,SPINK5
Regulation of antimicrobial humoral response	GO:0002759	false	3.81E-02	12	91	3	21026	EVPL,NOD2,SPINK5

Table S22. Solutions used in immunofluorescence (IF) staining. Related to Star Methods.

Fixation solution	4 % formaldehyde (16 % formaldehyde diluted in PBS)
Permeabilization solution	PBS, 0.4 % Triton-X, 1 % goat serum
Blocking solution 1	PBS, 20 % FcR Blocking Reagent, 10 % goat serum in PBS
Washing solution	PBS with 0.1 % Triton-X
Blocking solution 2	PBS, 2 % rabbit serum, 10 % goat serum
Blocking solution 3	0.5 ug/ul AffiniPure Fab Fragment Goat Anti-Rabbit IgG in PBS
anti-NEURL2 primary antibody solution	4 ng/ul in PBS with 10 % goat serum
anti-KRT8 primary antibody solution	0.5 ng/ul in PBS with 10 % goat serum
anti-IVL primary antibody solution	0.8 ng/ul in PBS with 10 % goat serum
anti-KRT1 primary antibody solution	0.25 ng/ul in PBS with 10 % goat serum
Isotype antibody solution	4, 0.5, 0.8, or 0.25 ng/ul in PBS with 10% goat serum, matching the primary antibody to be controlled for
Secondary antibody solution	2 ng/ul in PBS with 10 % goat serum
DAPI staining solution	2 ug/ml in PBS

Data S1: Full-size grayscale per-channel images corresponding to Figure 5 and S9

Contents

Images used in Figure 5A-5C

- KRT1 signal (Alexa Fluor Plus 555)
- IVL signal (Alexa Fluor Plus 647)
- DAPI signal

Images used in Figure 5D-5K

- NEURL2 signal (Alexa Fluor Plus 555)
- KRT8 signal (Alexa Fluor Plus 647)
- DAPI signal

Images used in Figure S9A

- KRT1 signal (Alexa Fluor Plus 555)
- IVL signal (Alexa Fluor Plus 647)
- DAPI signal

Images used in Figure S9C-S9H

- NEURL2 signal (Alexa Fluor Plus 555)
- KRT8 signal (Alexa Fluor Plus 647)
- DAPI signal

Images used in Figure 5A-5C

KRT1 signal (Alexa Fluor Plus 555)

5A KRT1

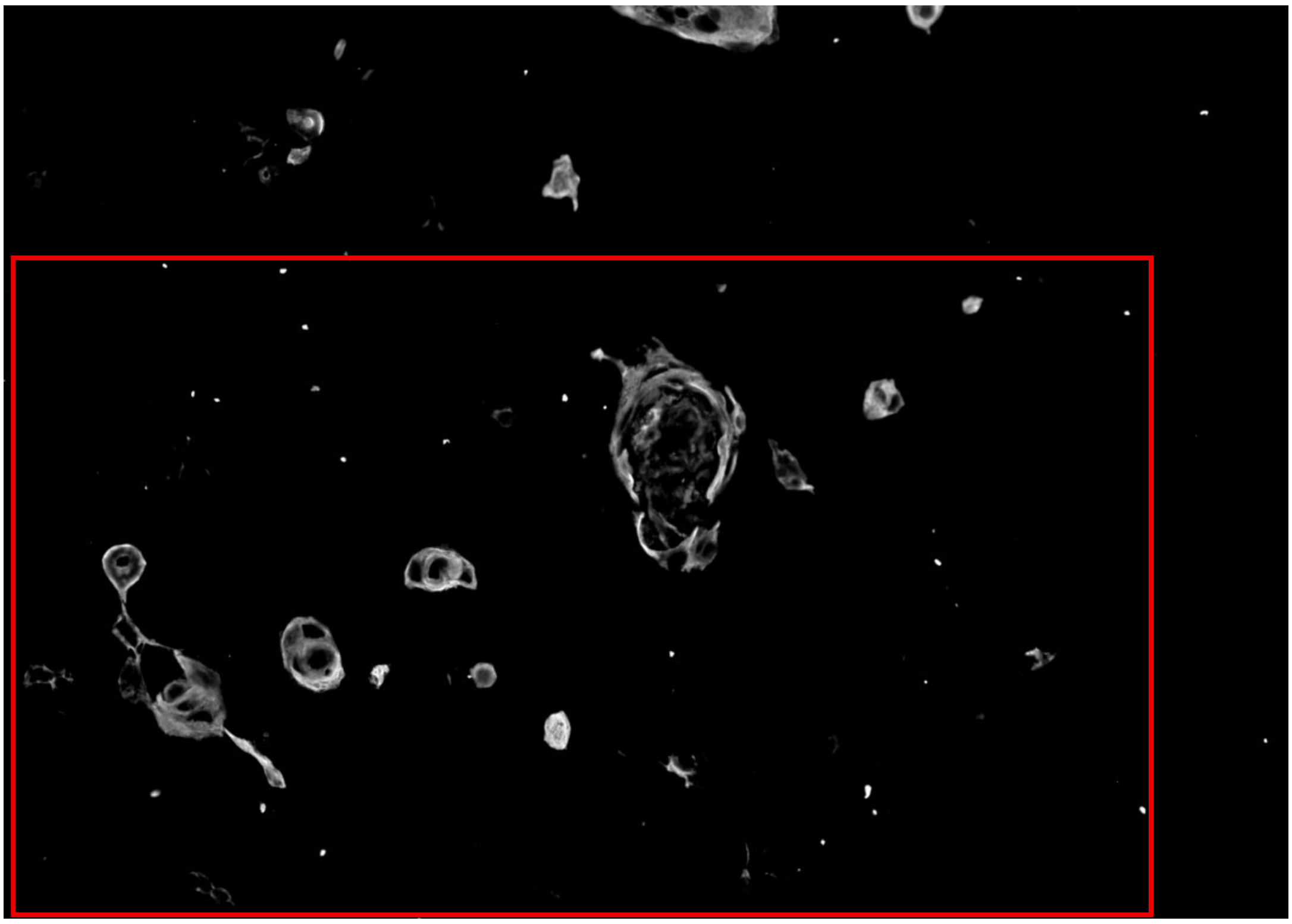

5B KRT1

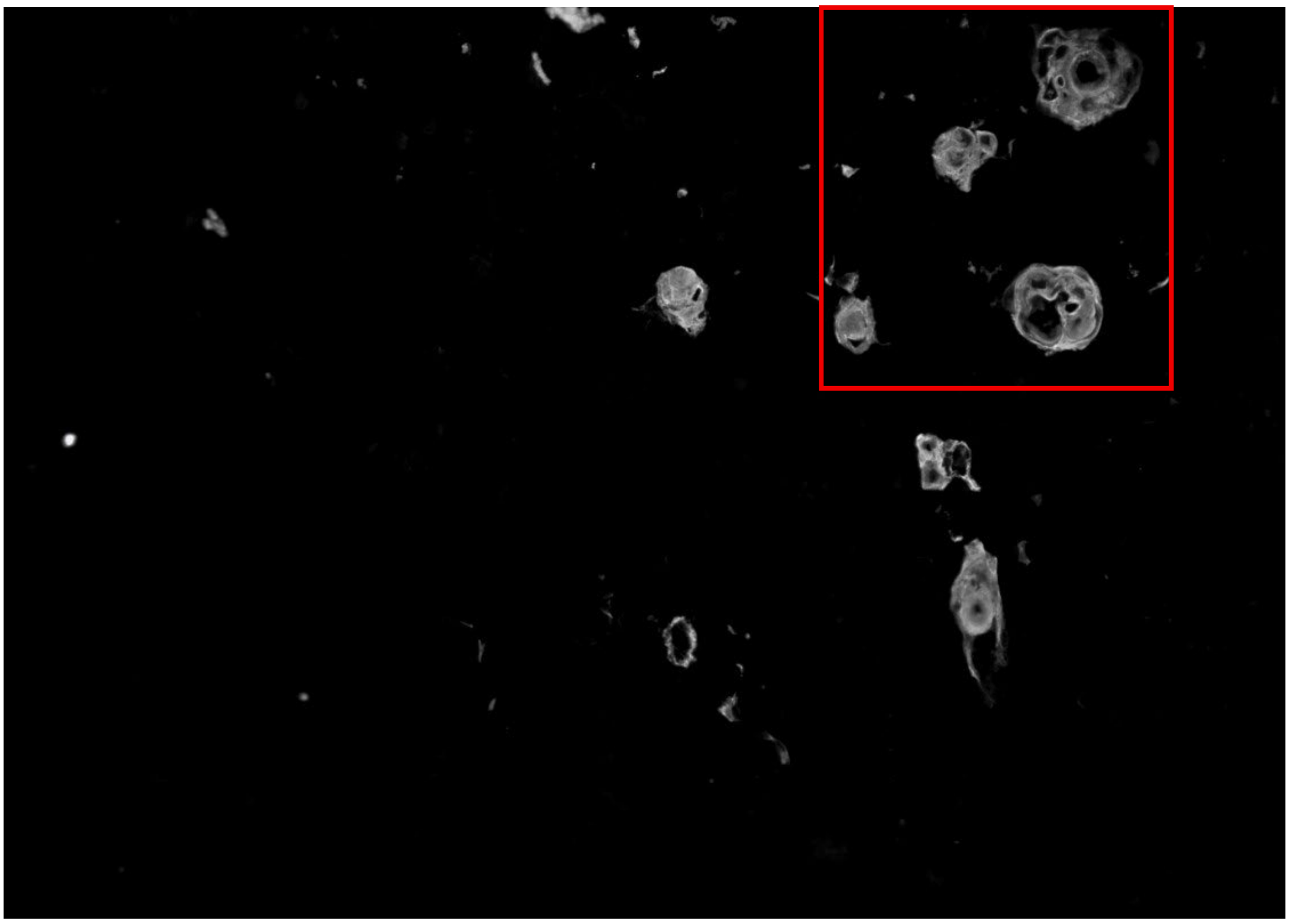

5C KRT1

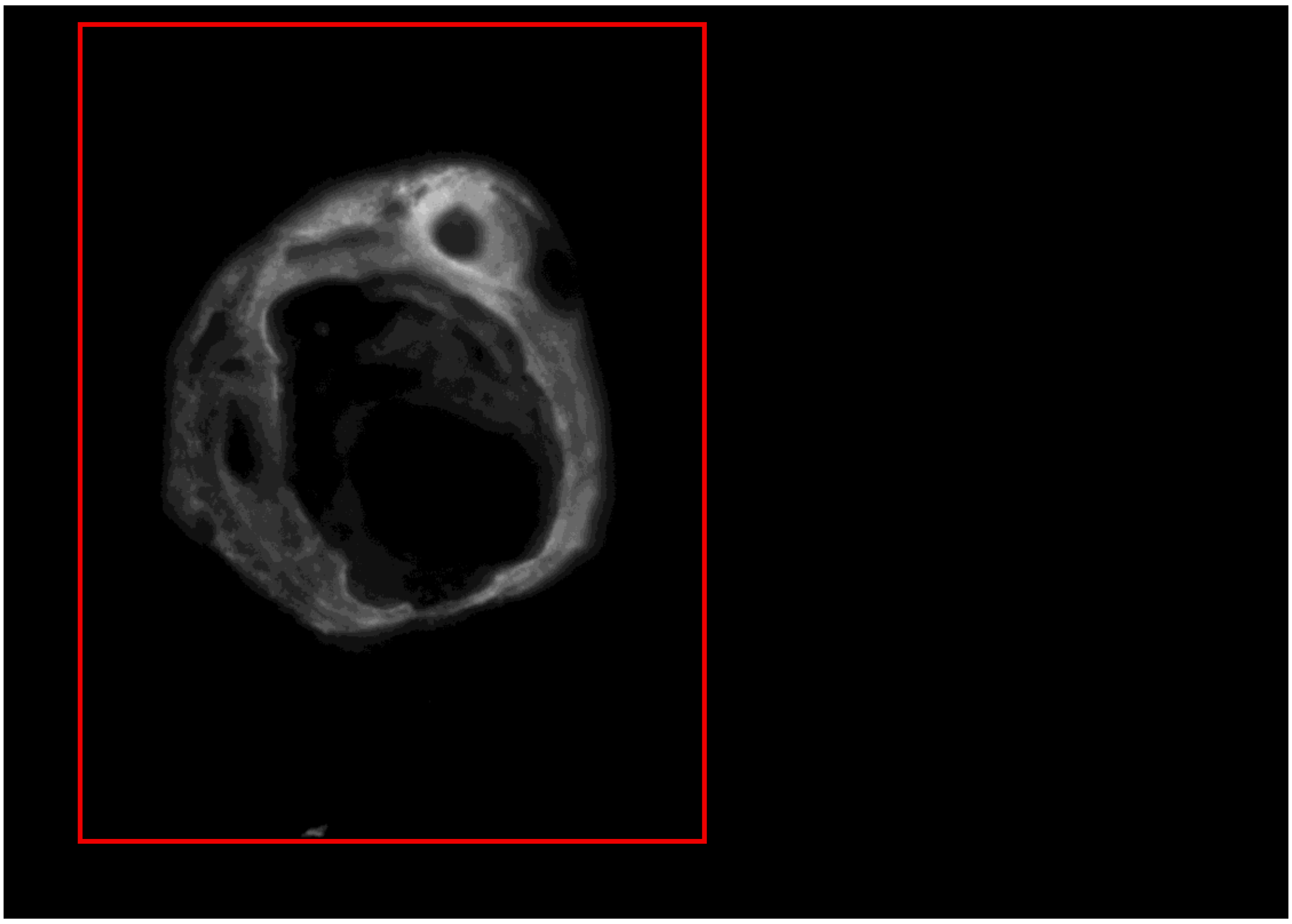

IVL signal (Alexa Fluor Plus 647)

5A IVL

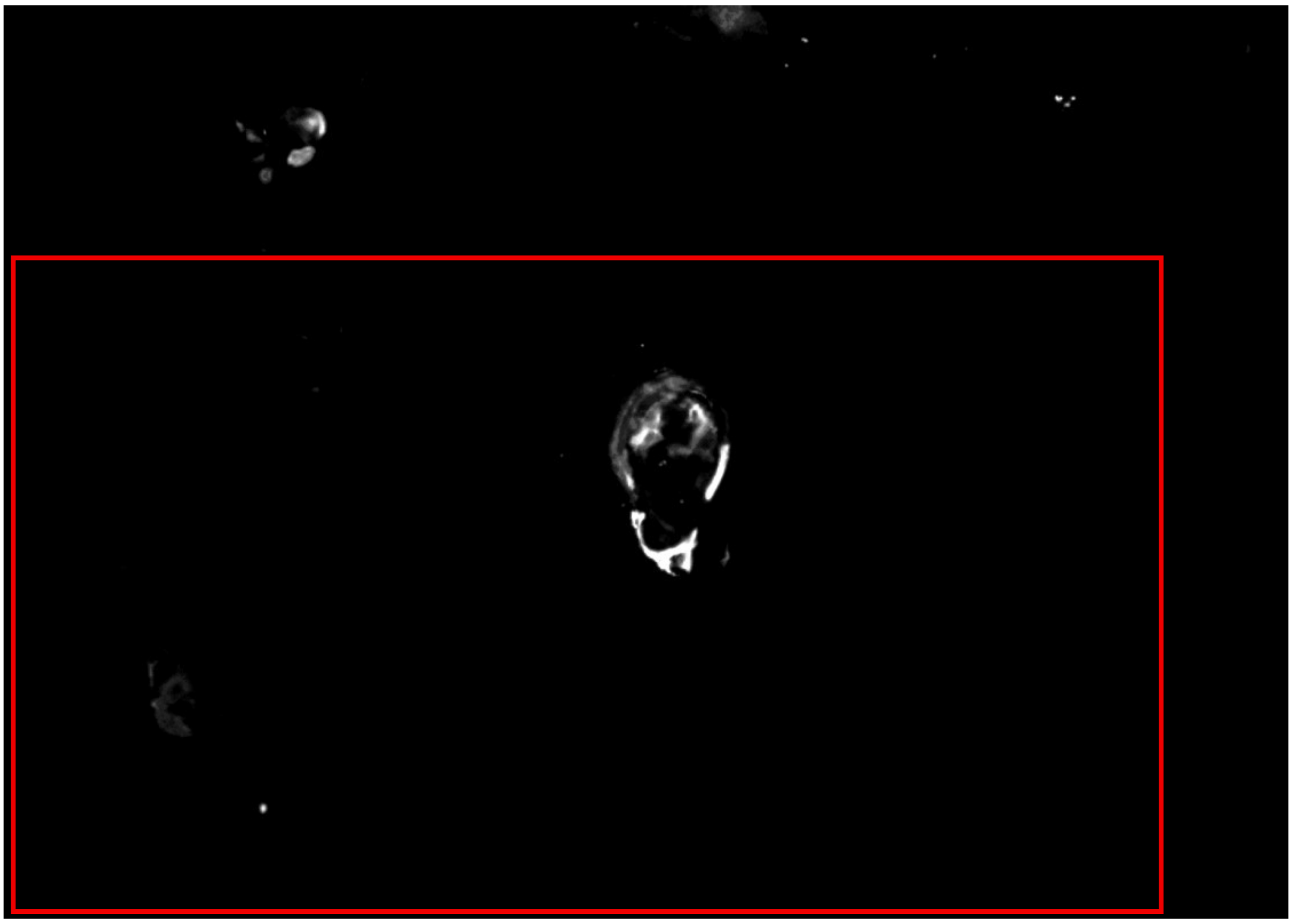

5B

IVL

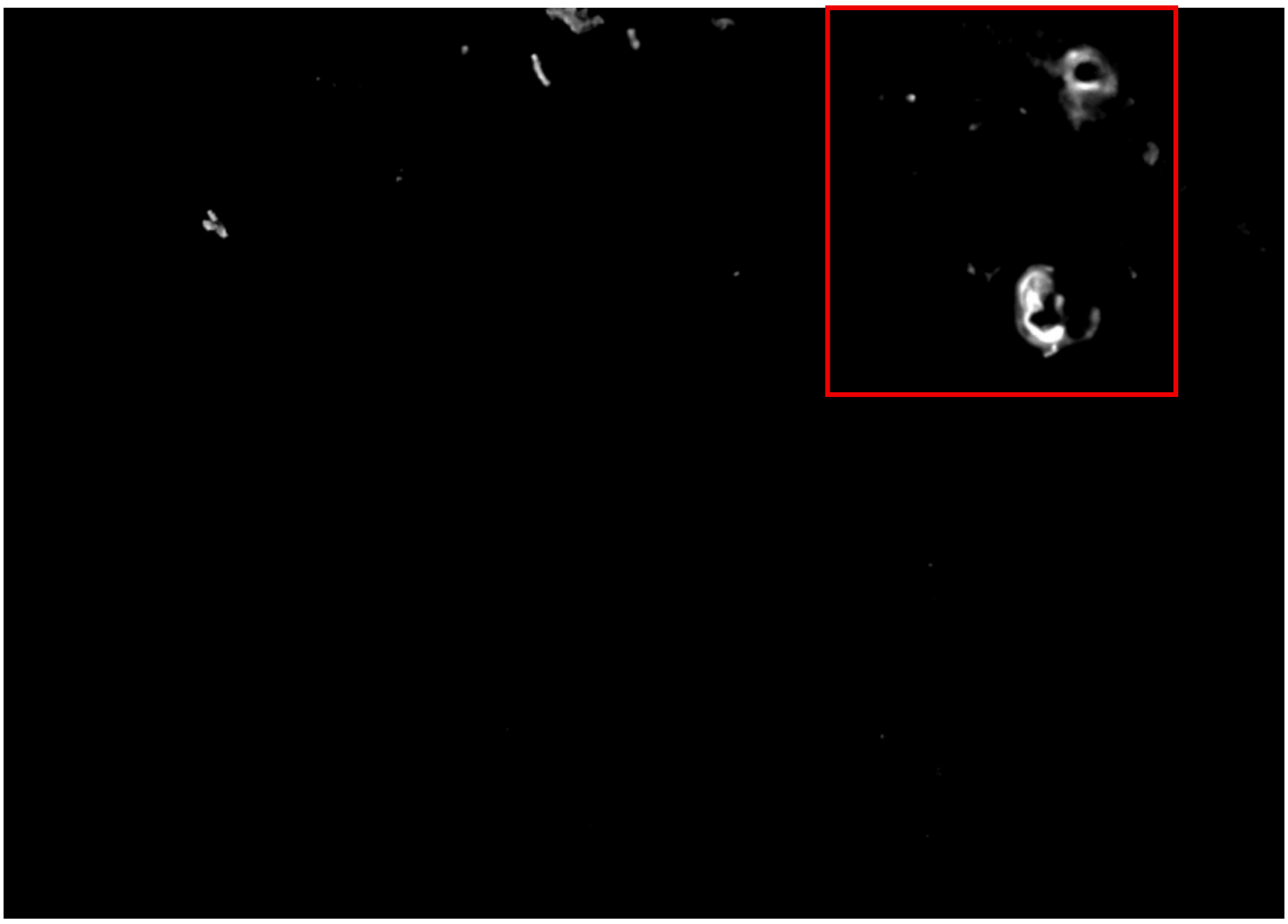

5C

IVL

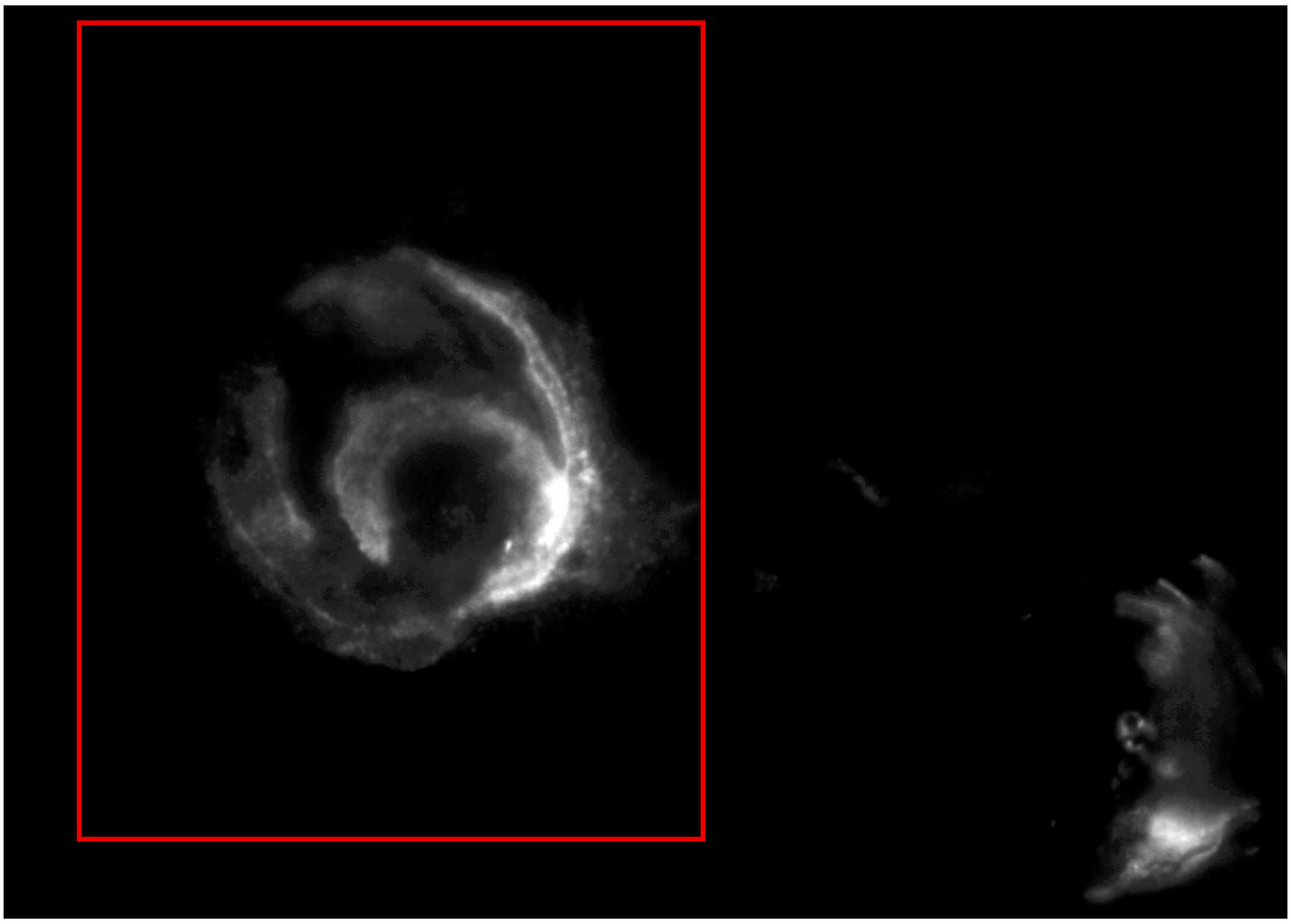

DAPI signal

5A DAPI

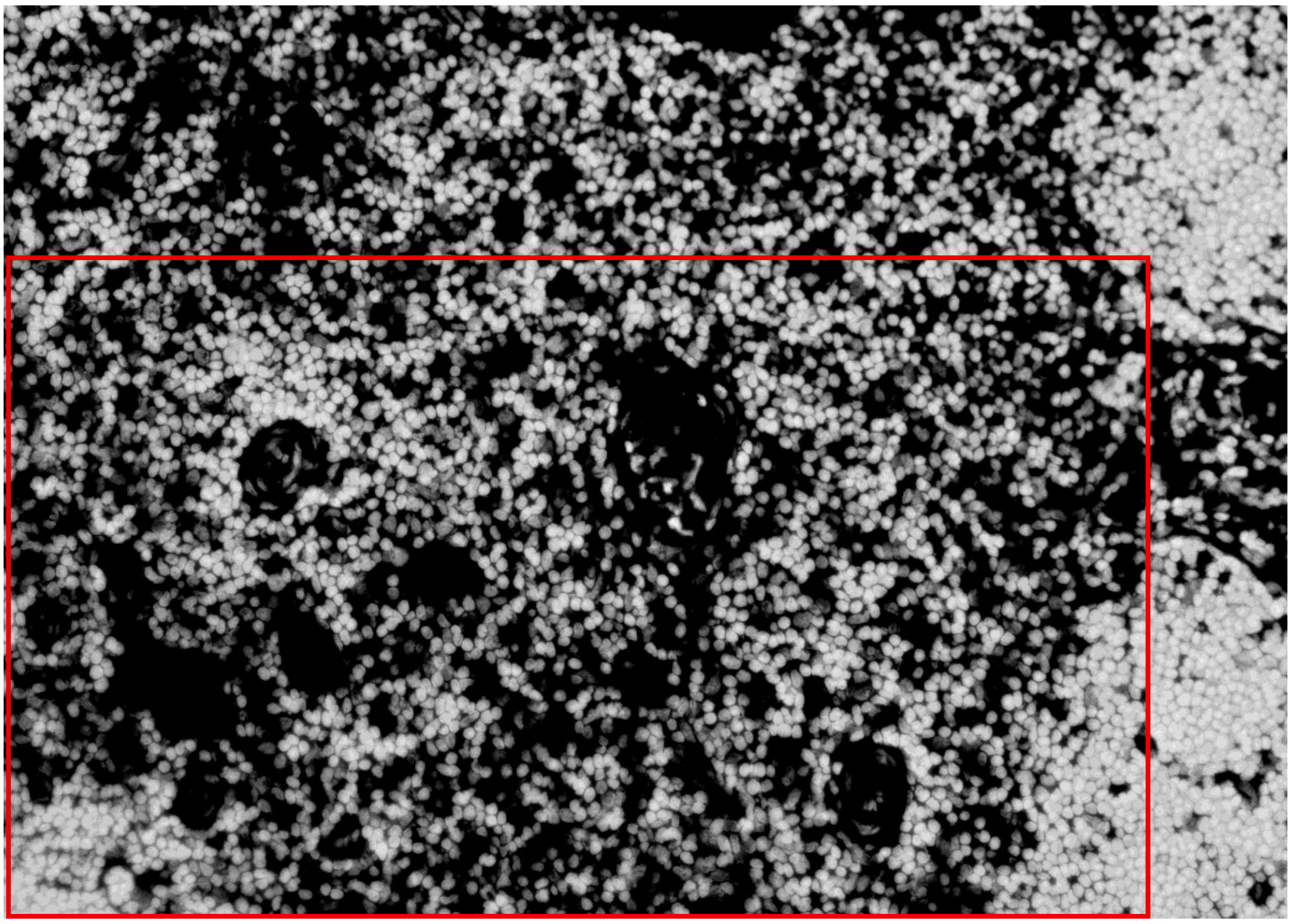

5B DAPI

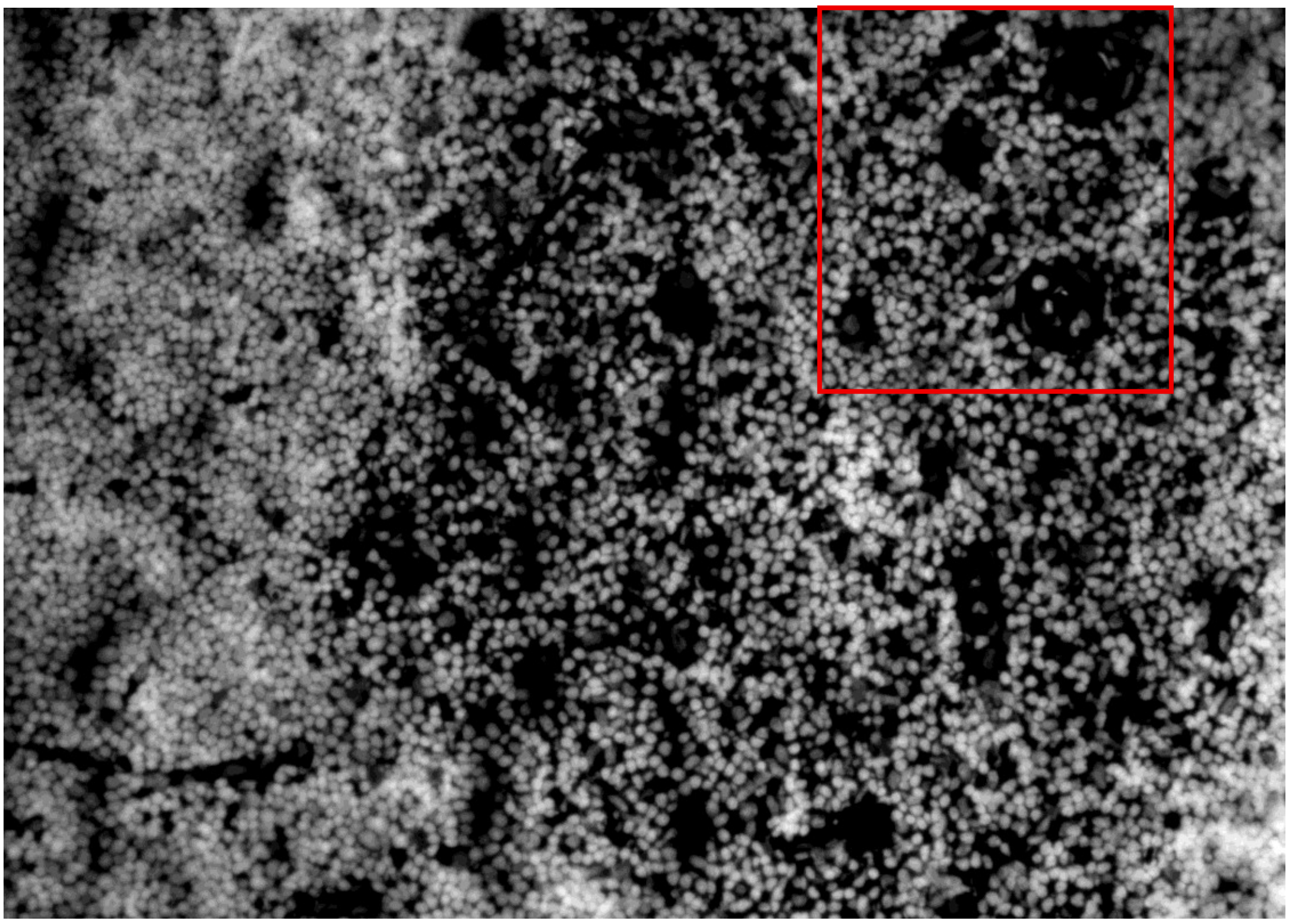

5C

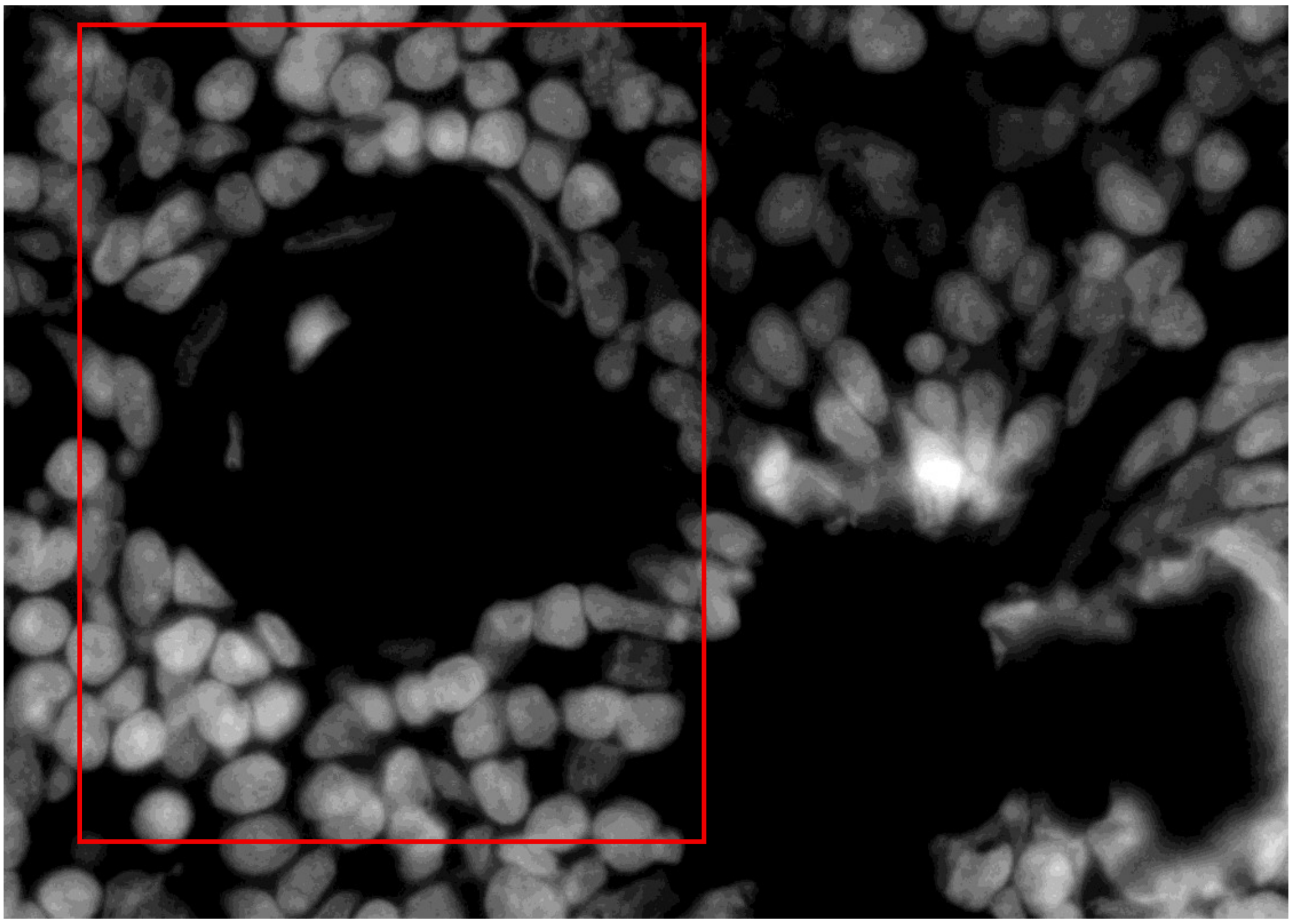

Images used in Figure 5D-5K

NEURL2 signal (Alexa Fluor Plus 555)

5D left NEURL2

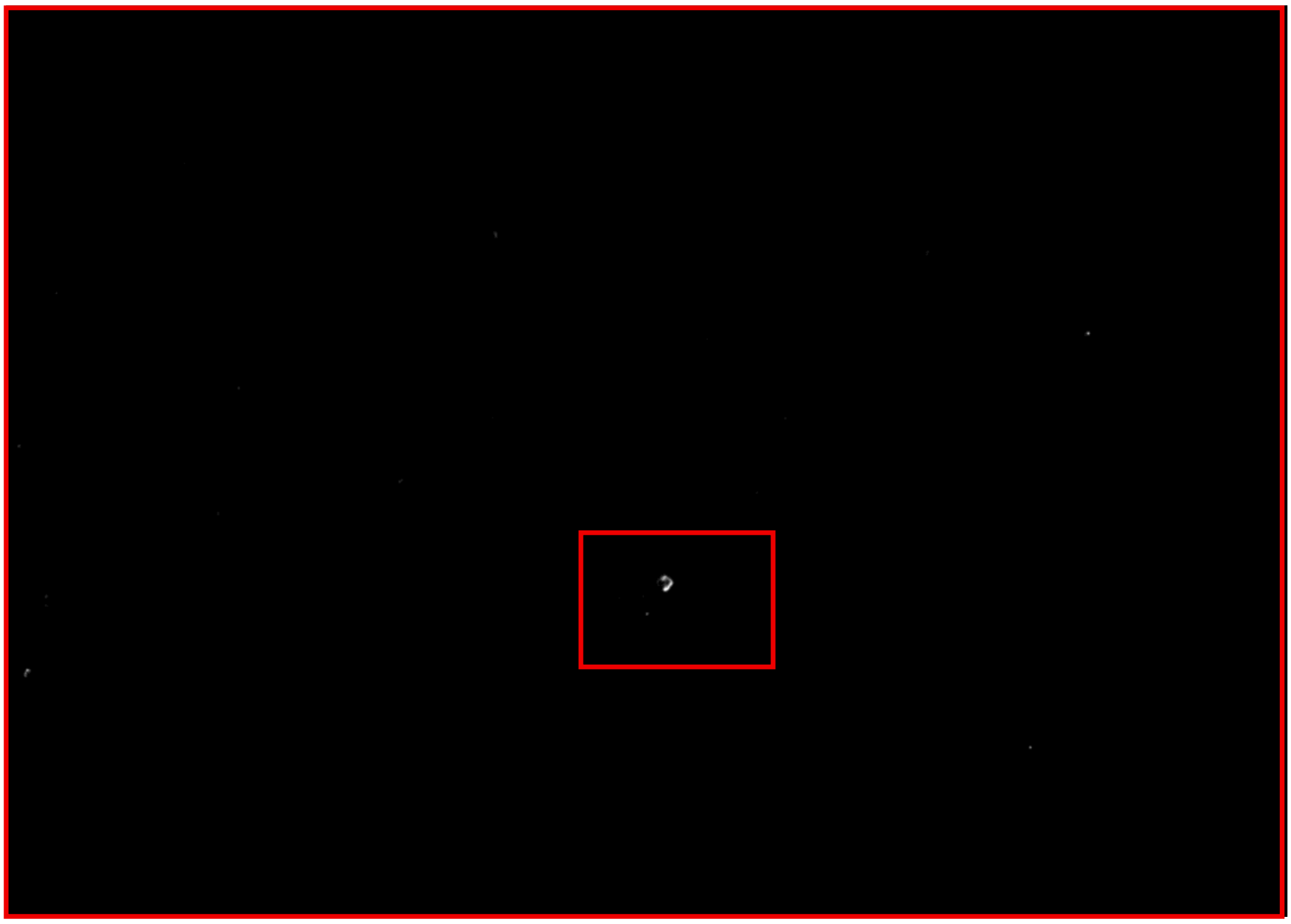

5D middle NEURL2

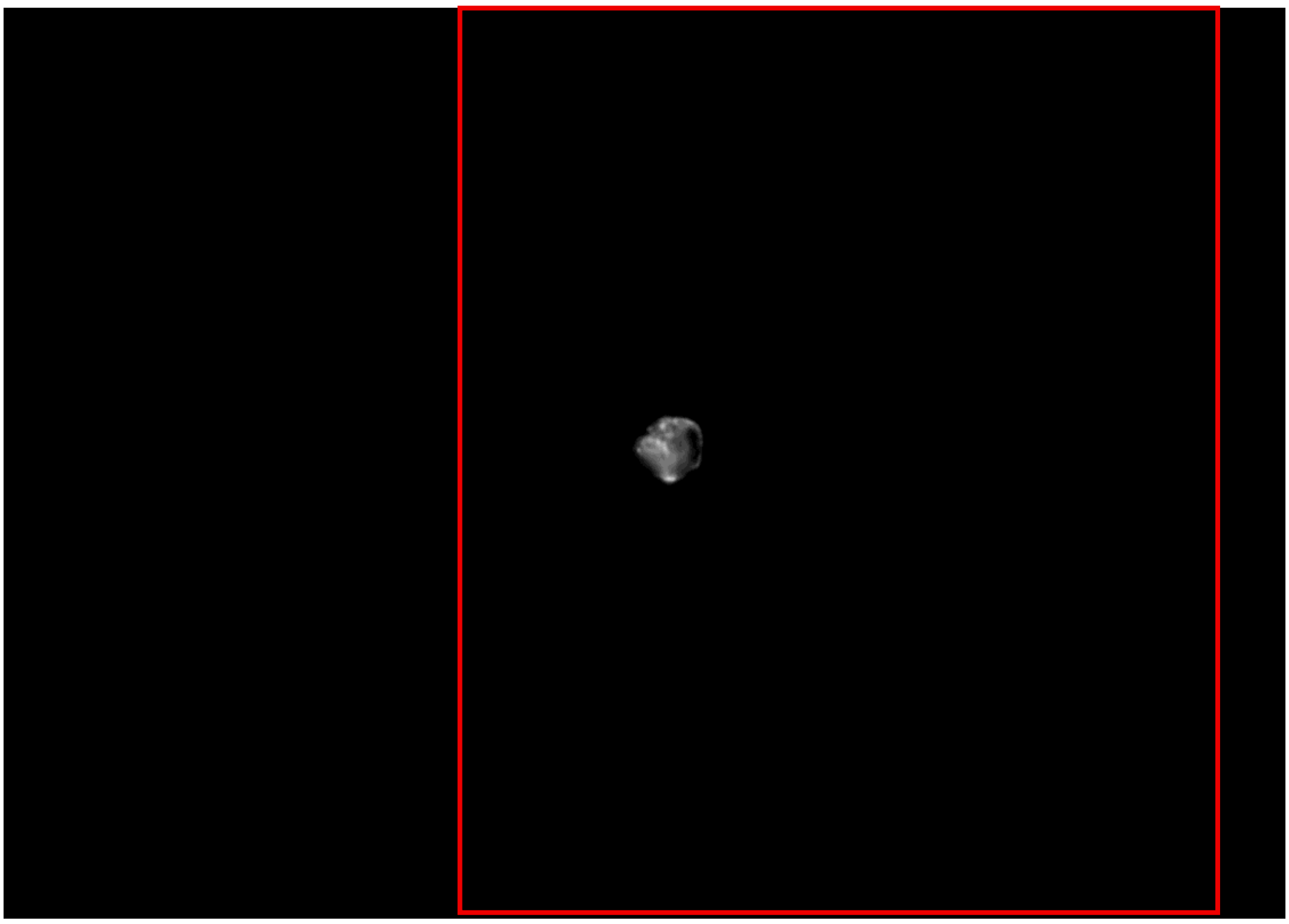

5D right NEURL2

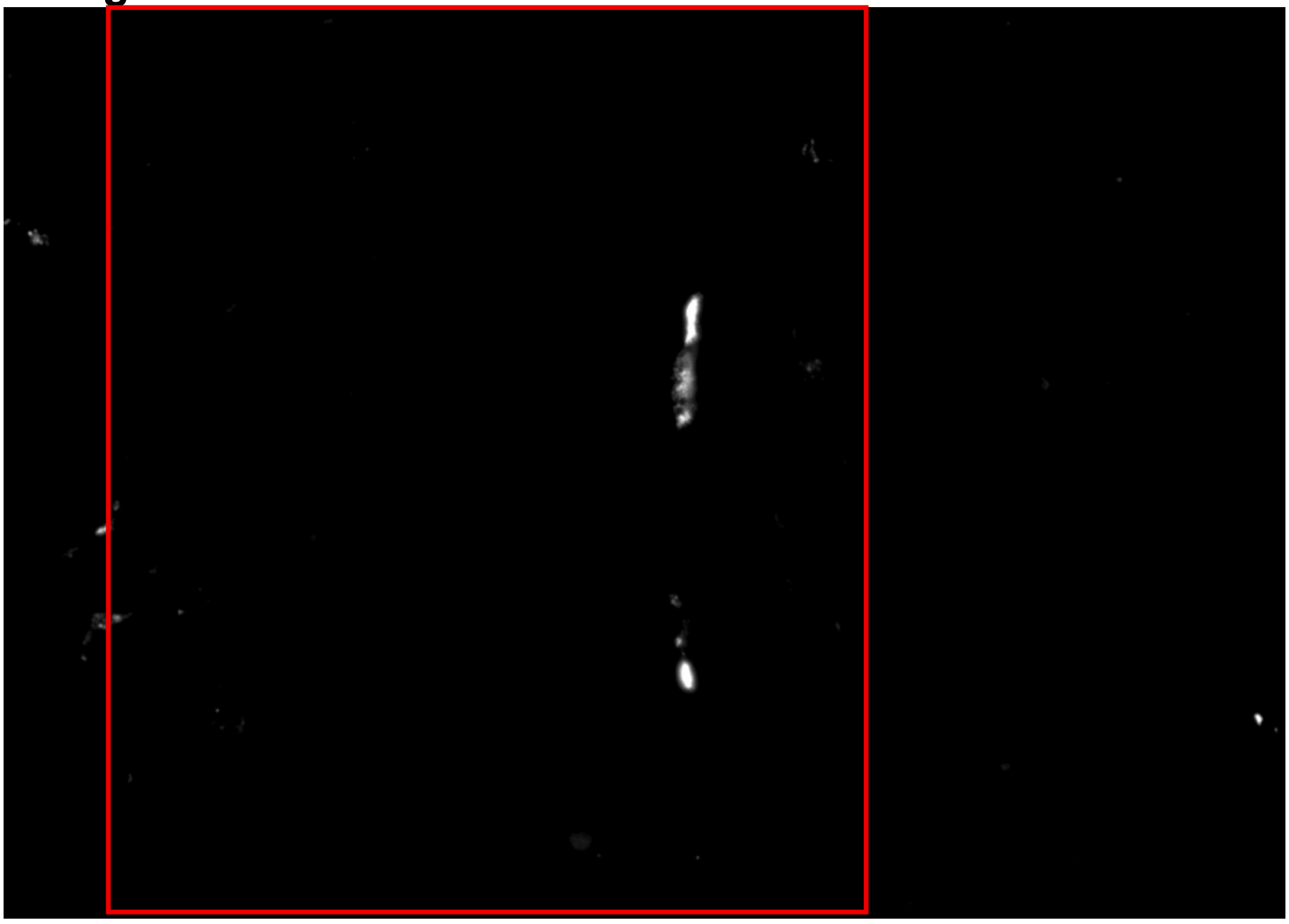

5E NEURL2

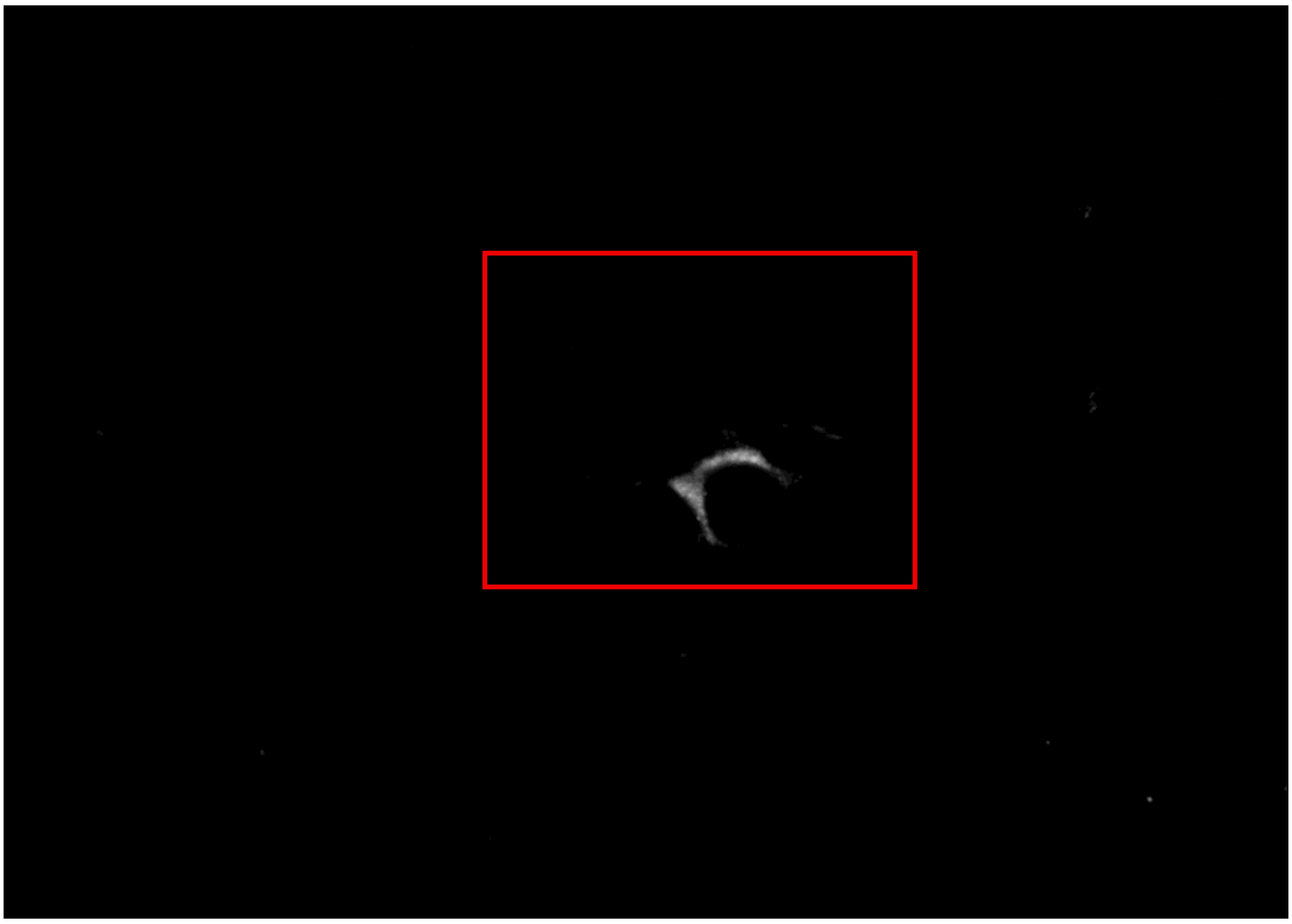

5F left NEURL2

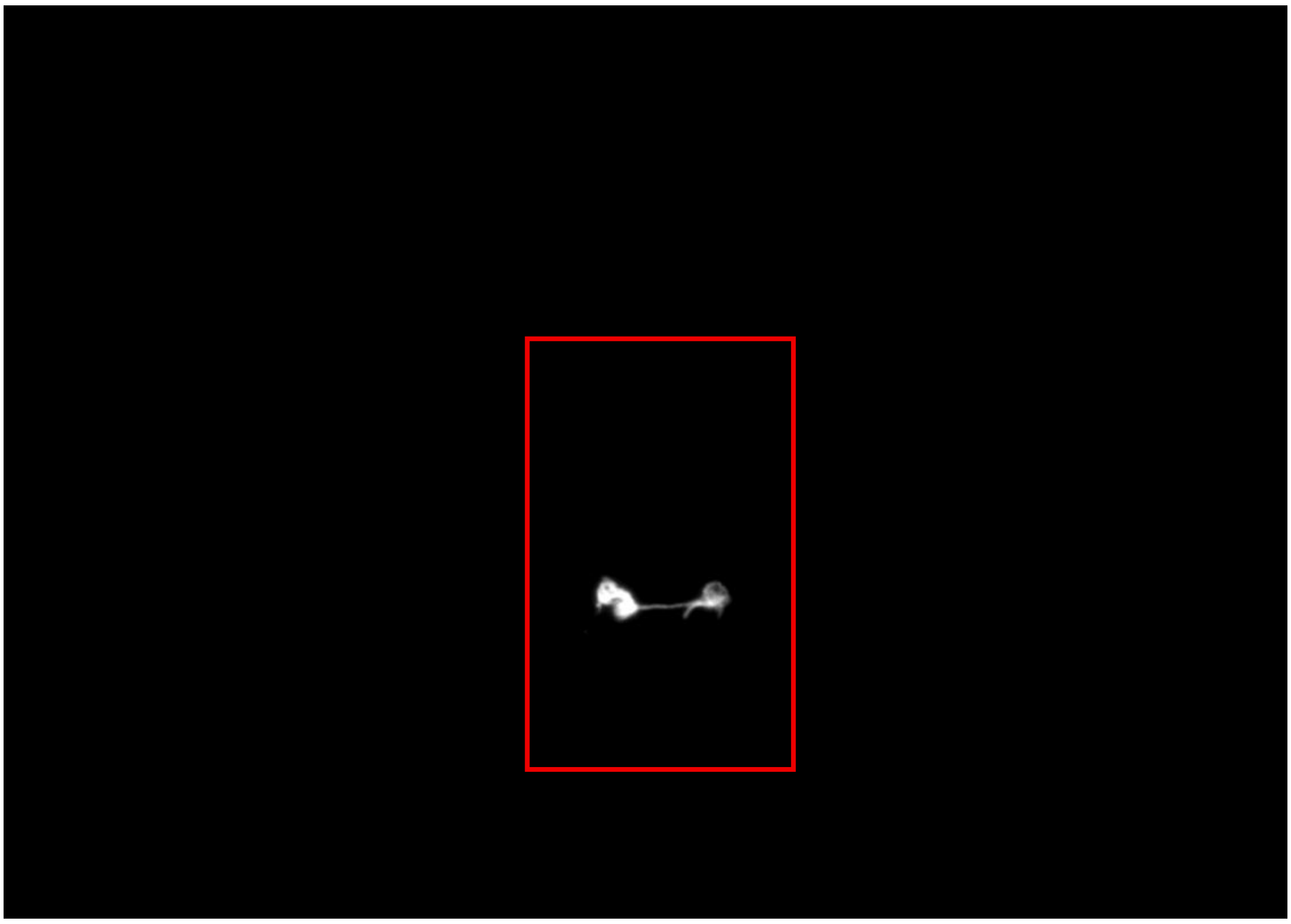

5F middle NEURL2

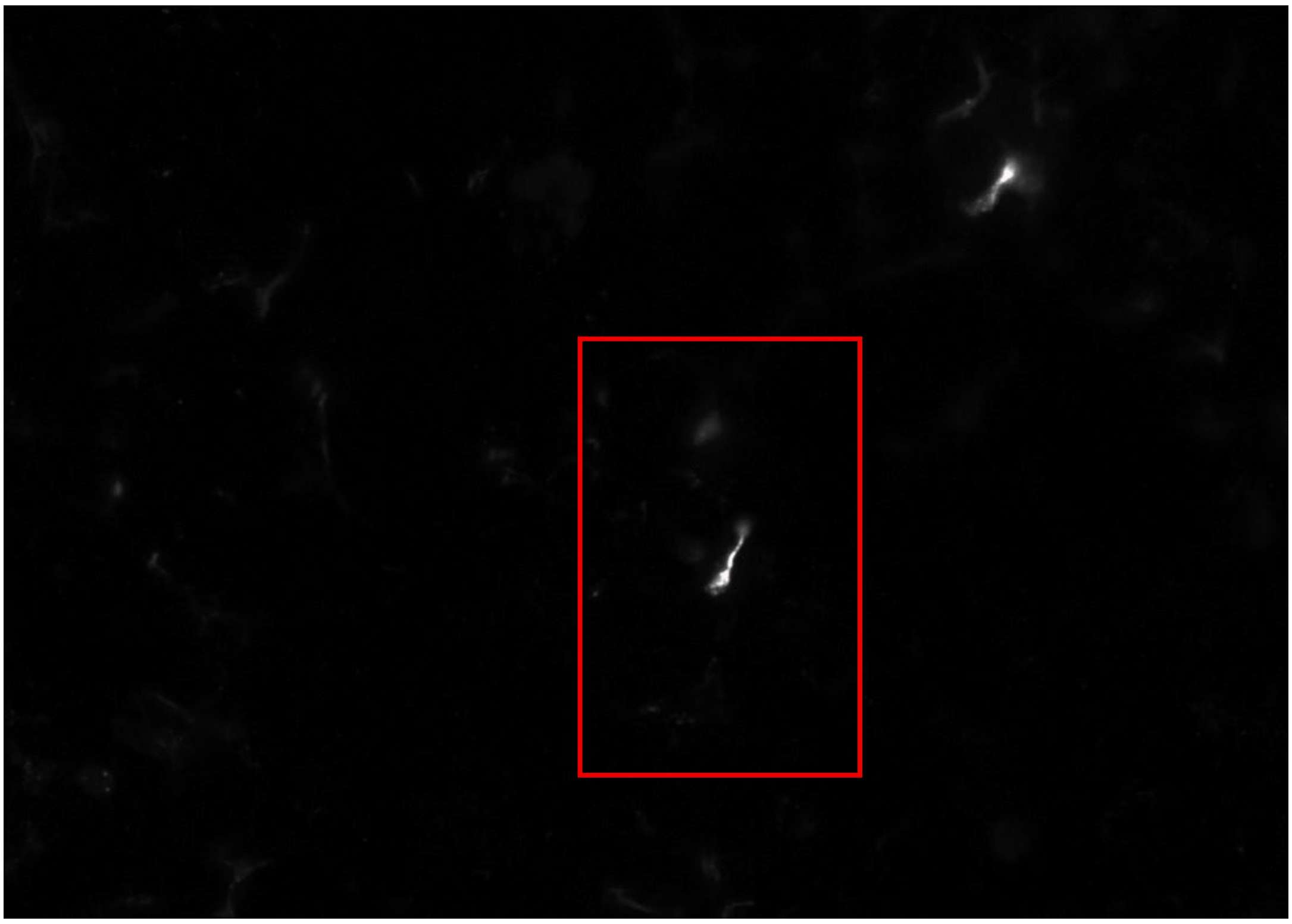

5F right NEURL2

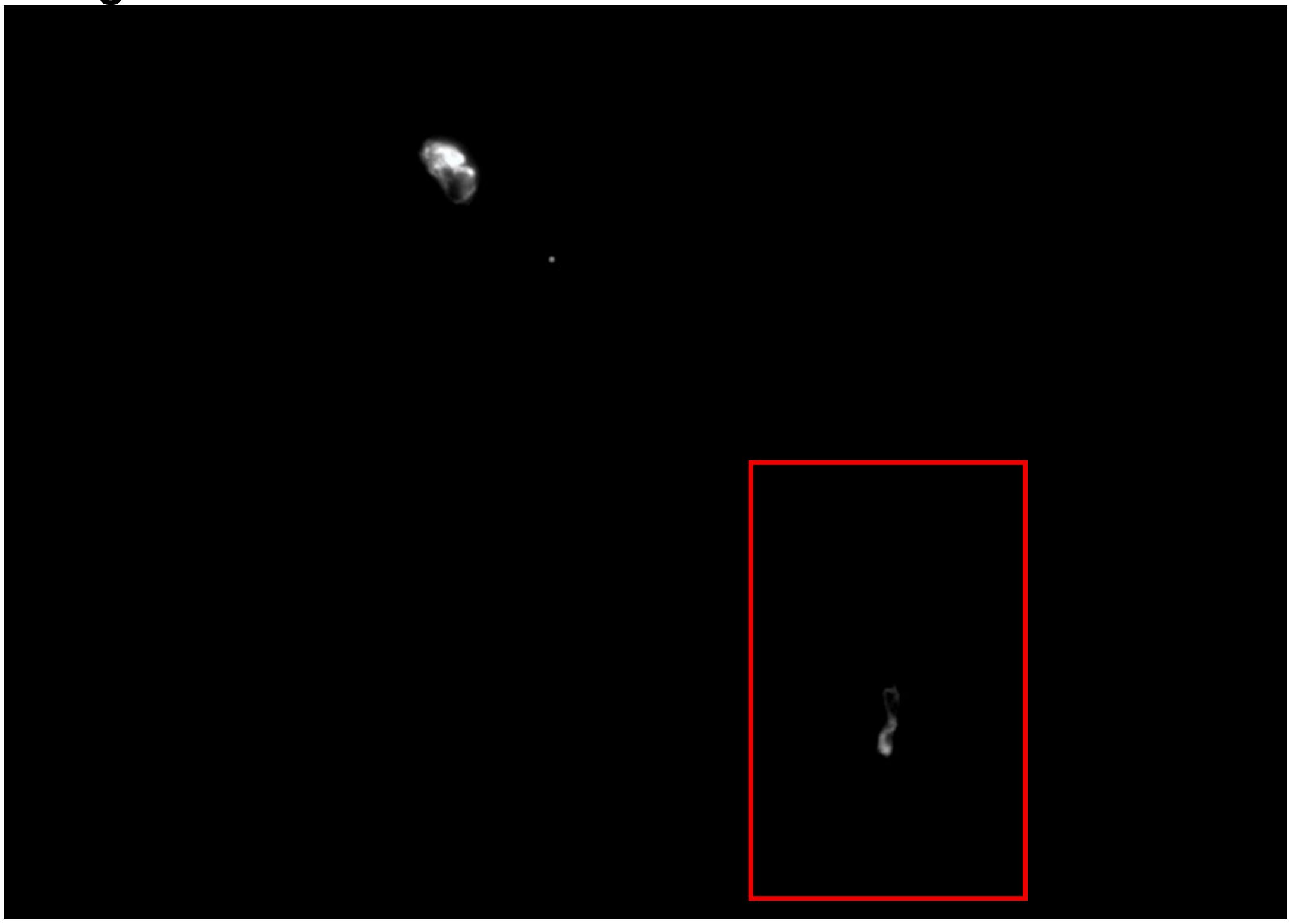

5G NEURL2

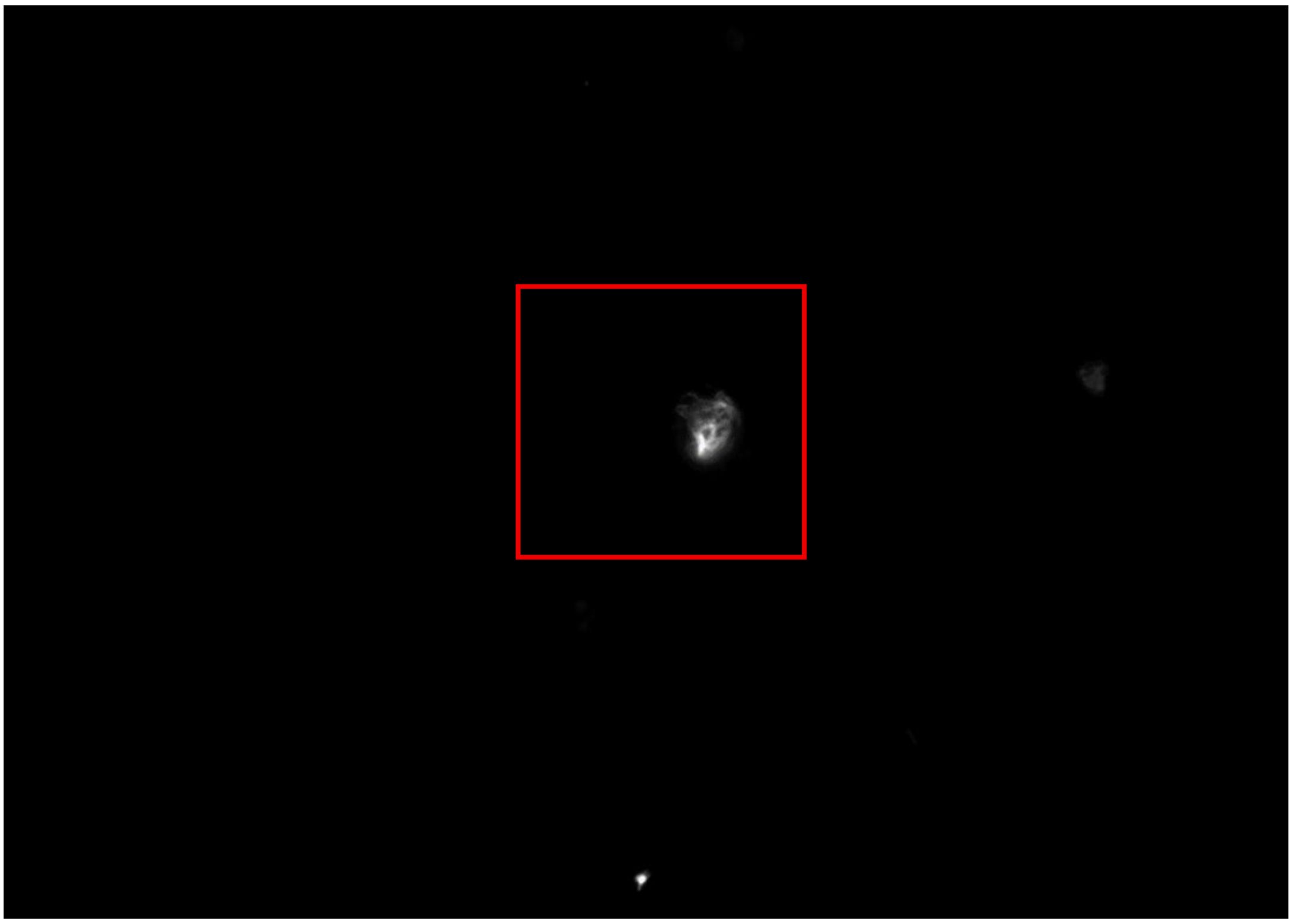

5H NEURL2

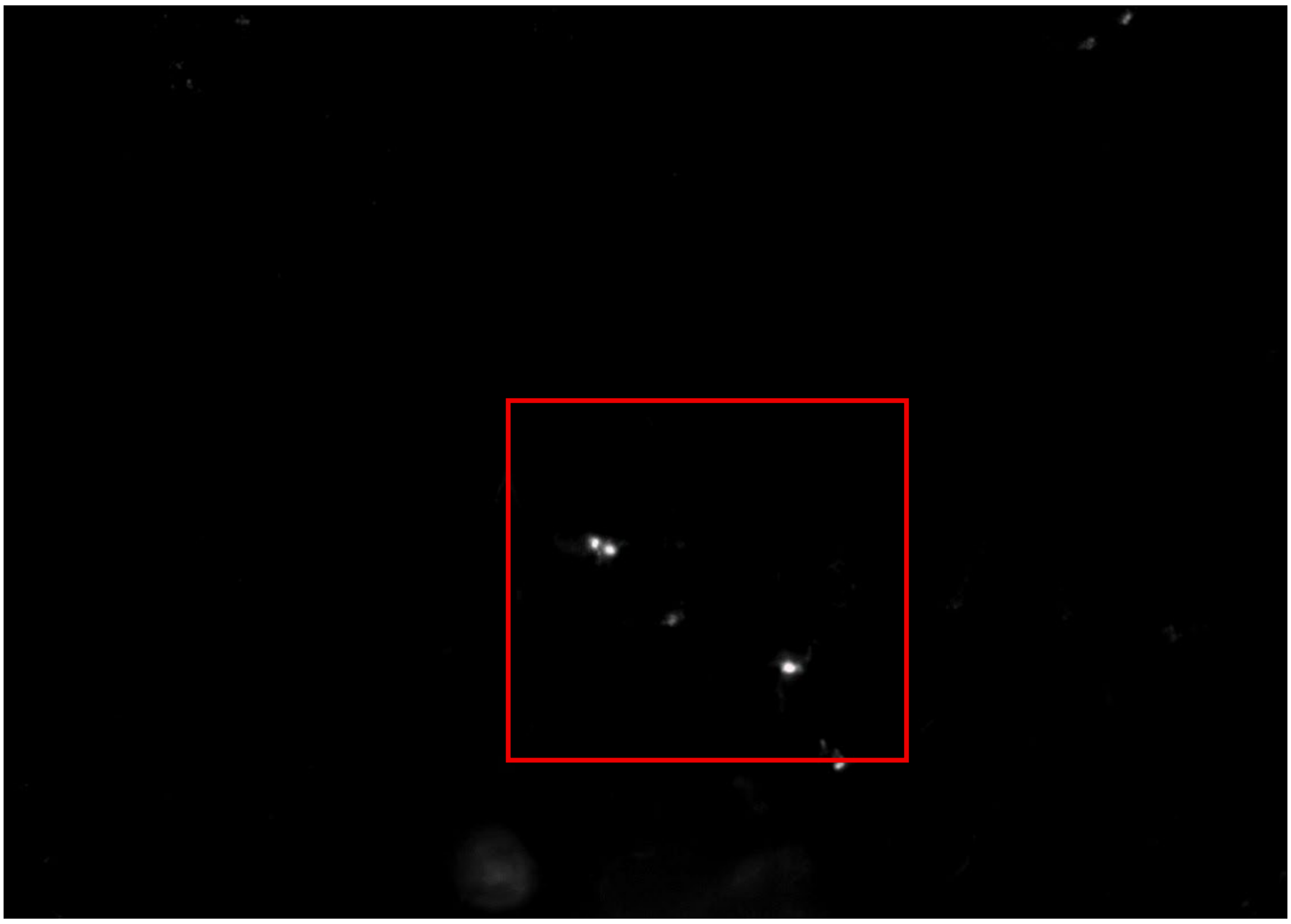

5I left NEURL2

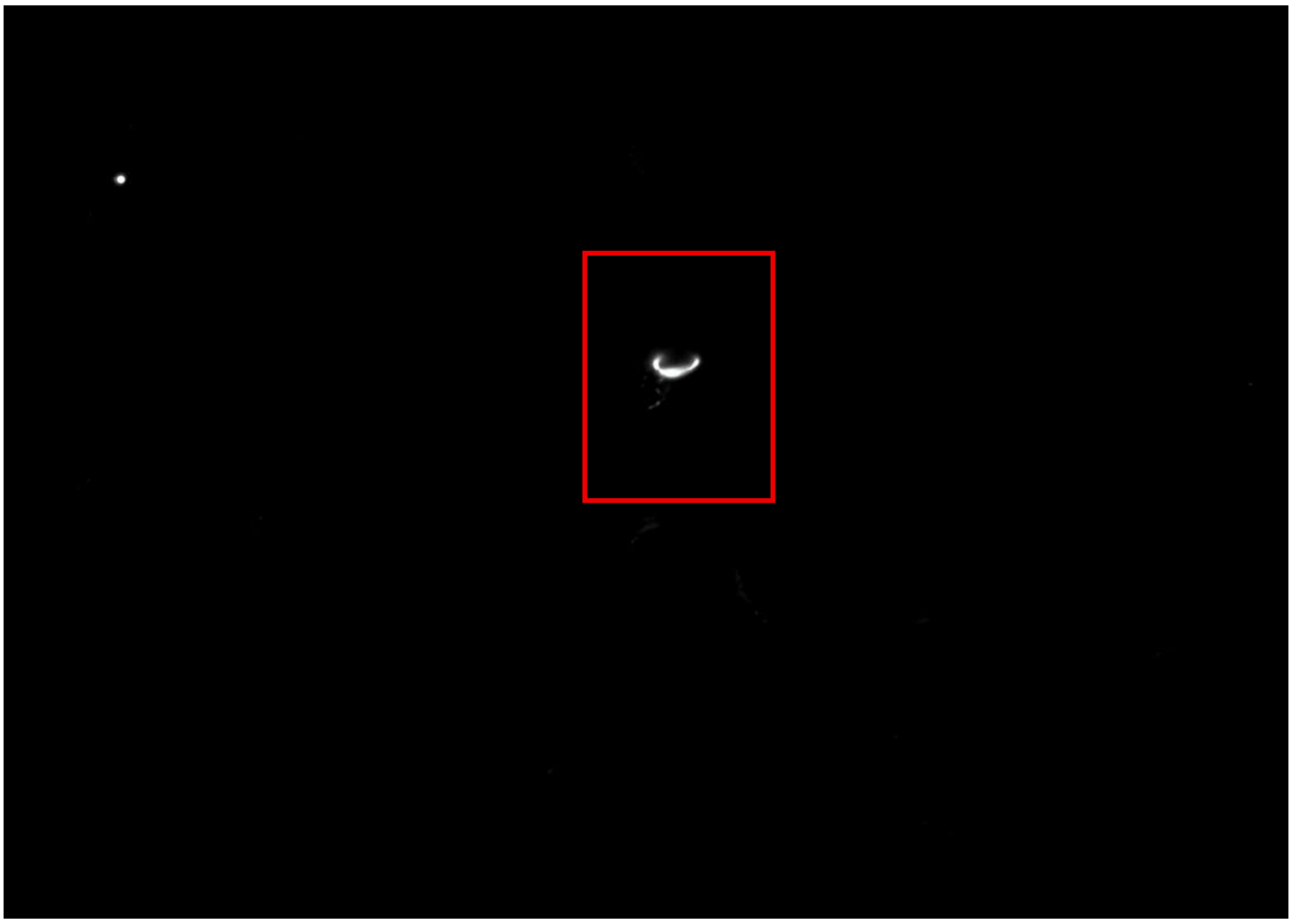

5I upper right NEURL2

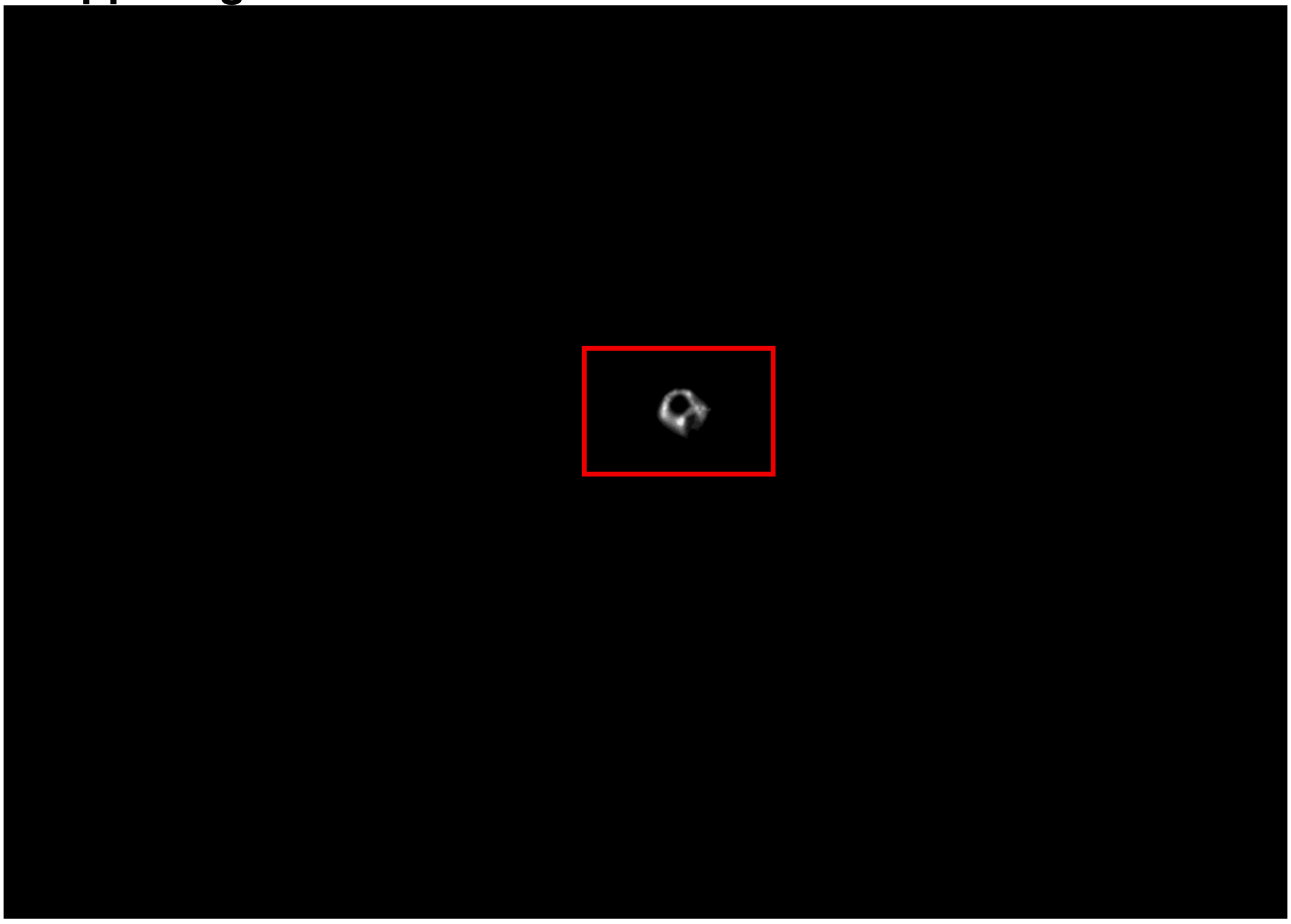

5I lower right NEURL2

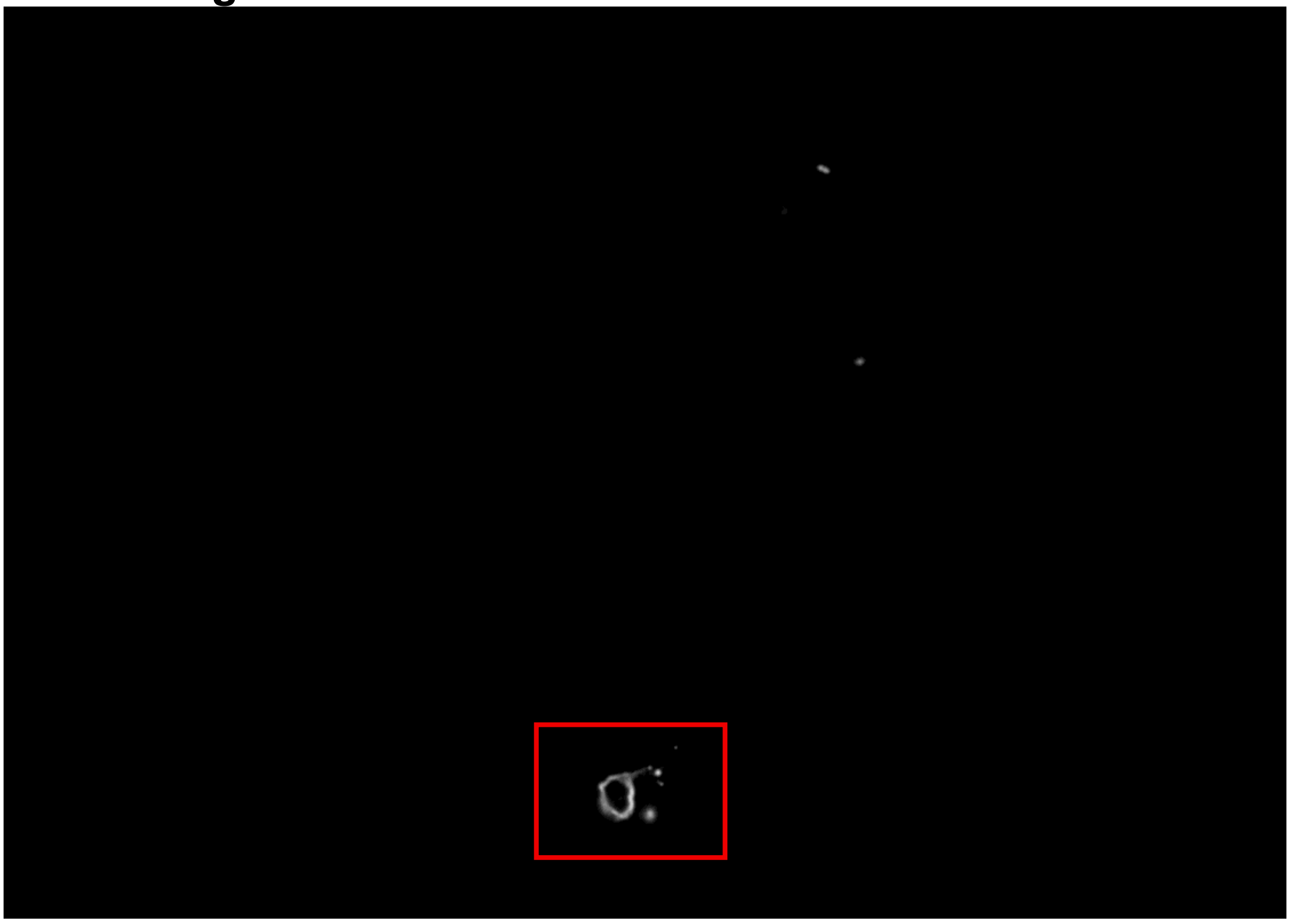

5J left NEURL2

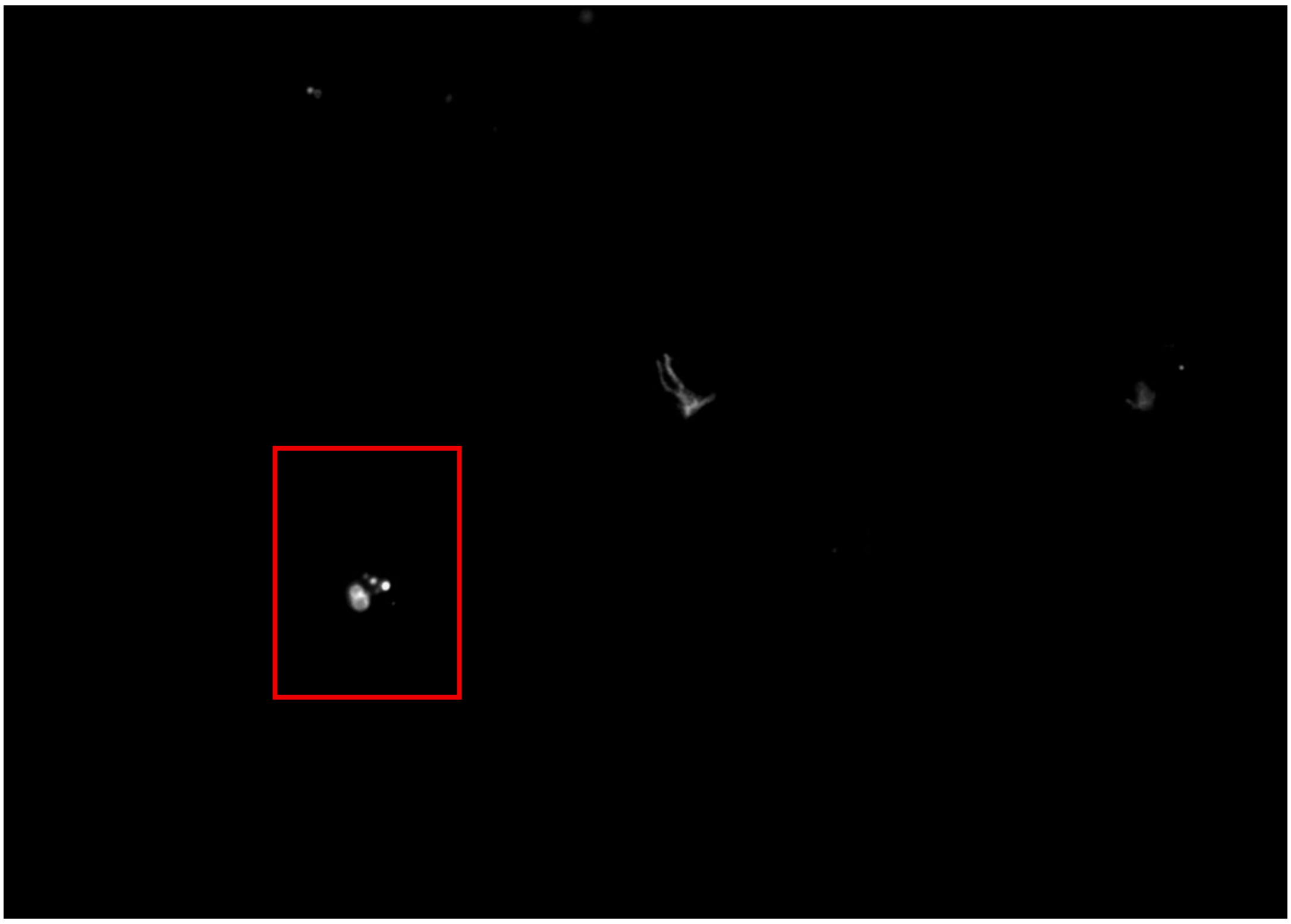

5J right NEURL2

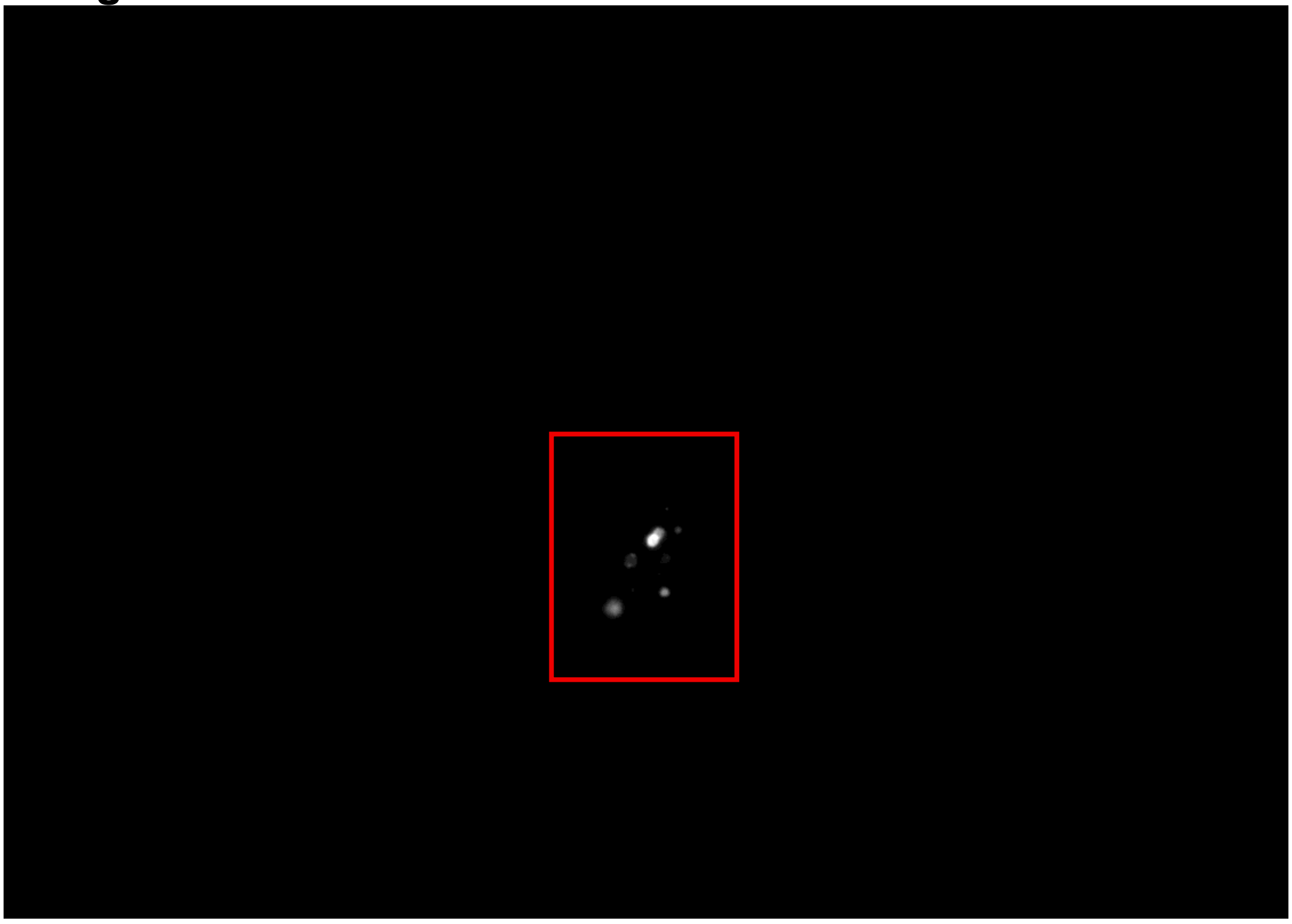

5K left NEURL2

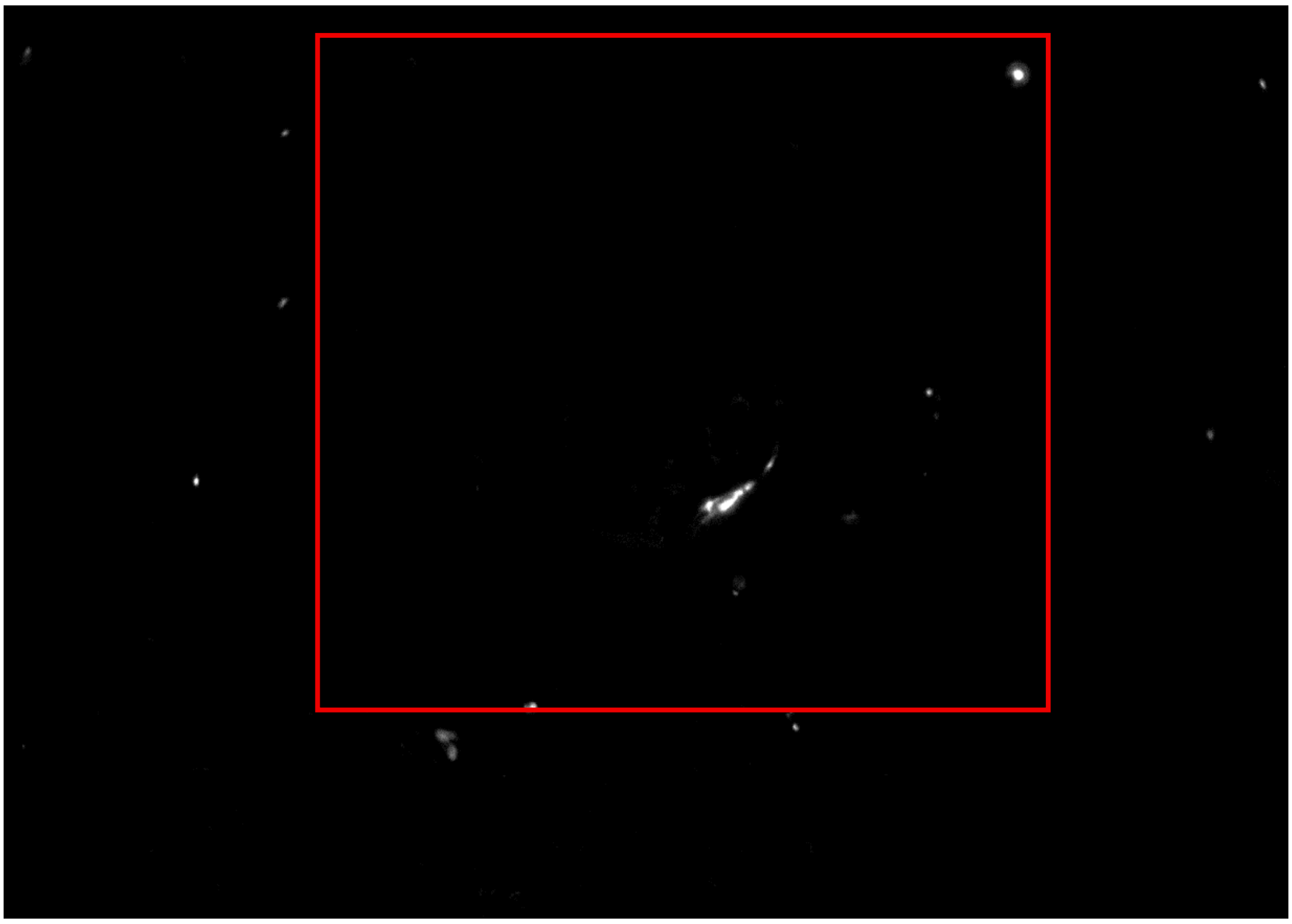

5K right NEURL2

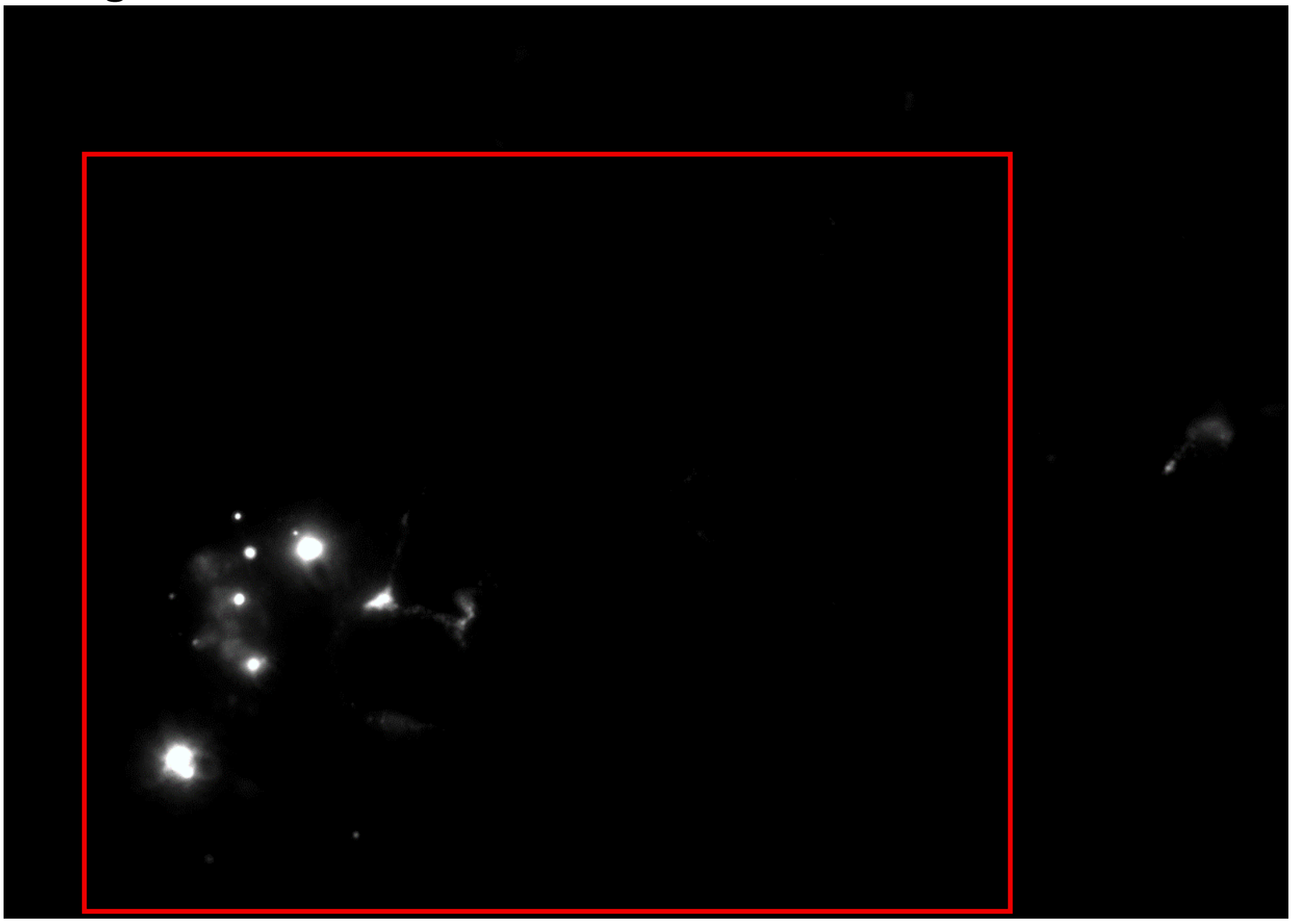

KRT8 signal (Alexa Fluor Plus 647)

5D left KRT8

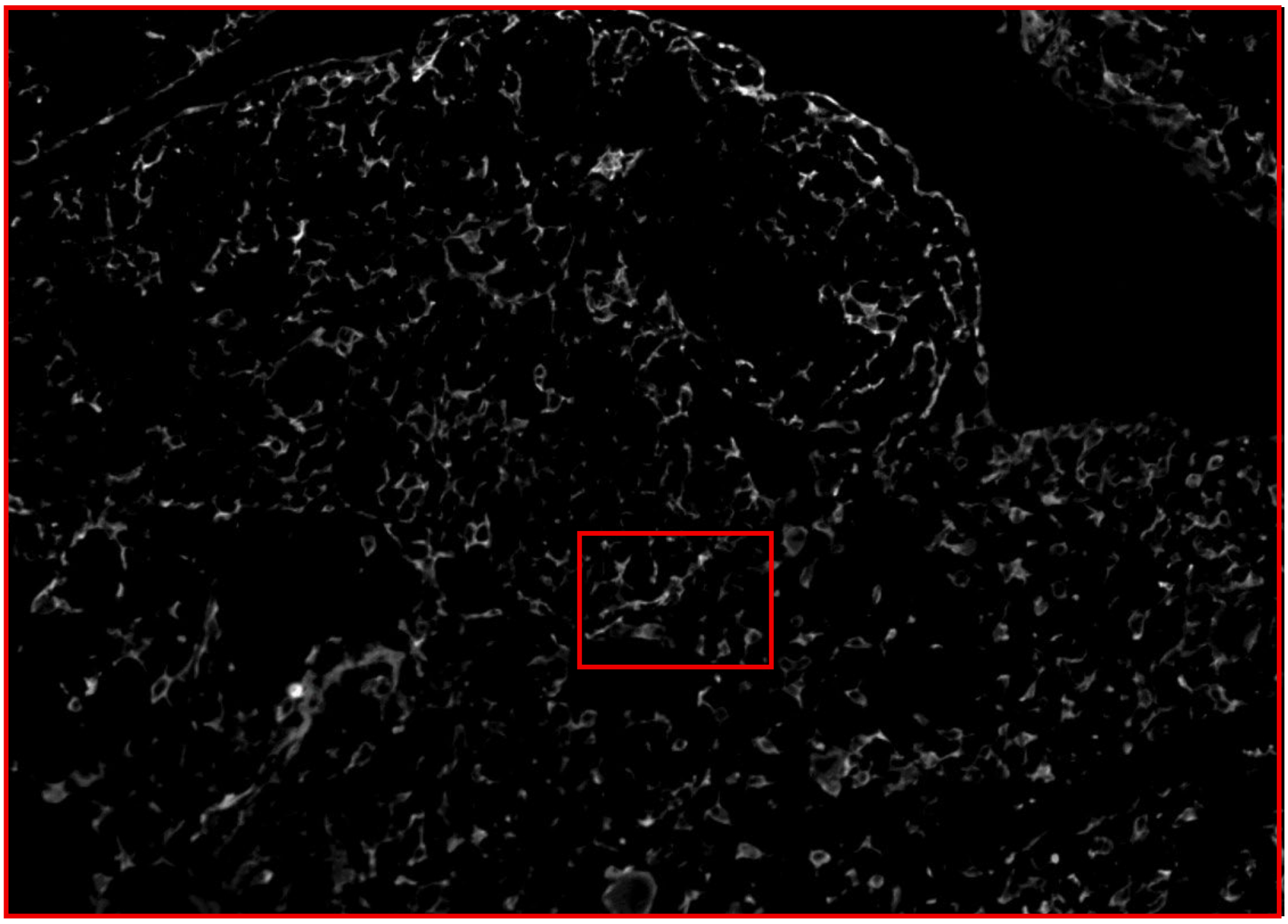

5D middle KRT8

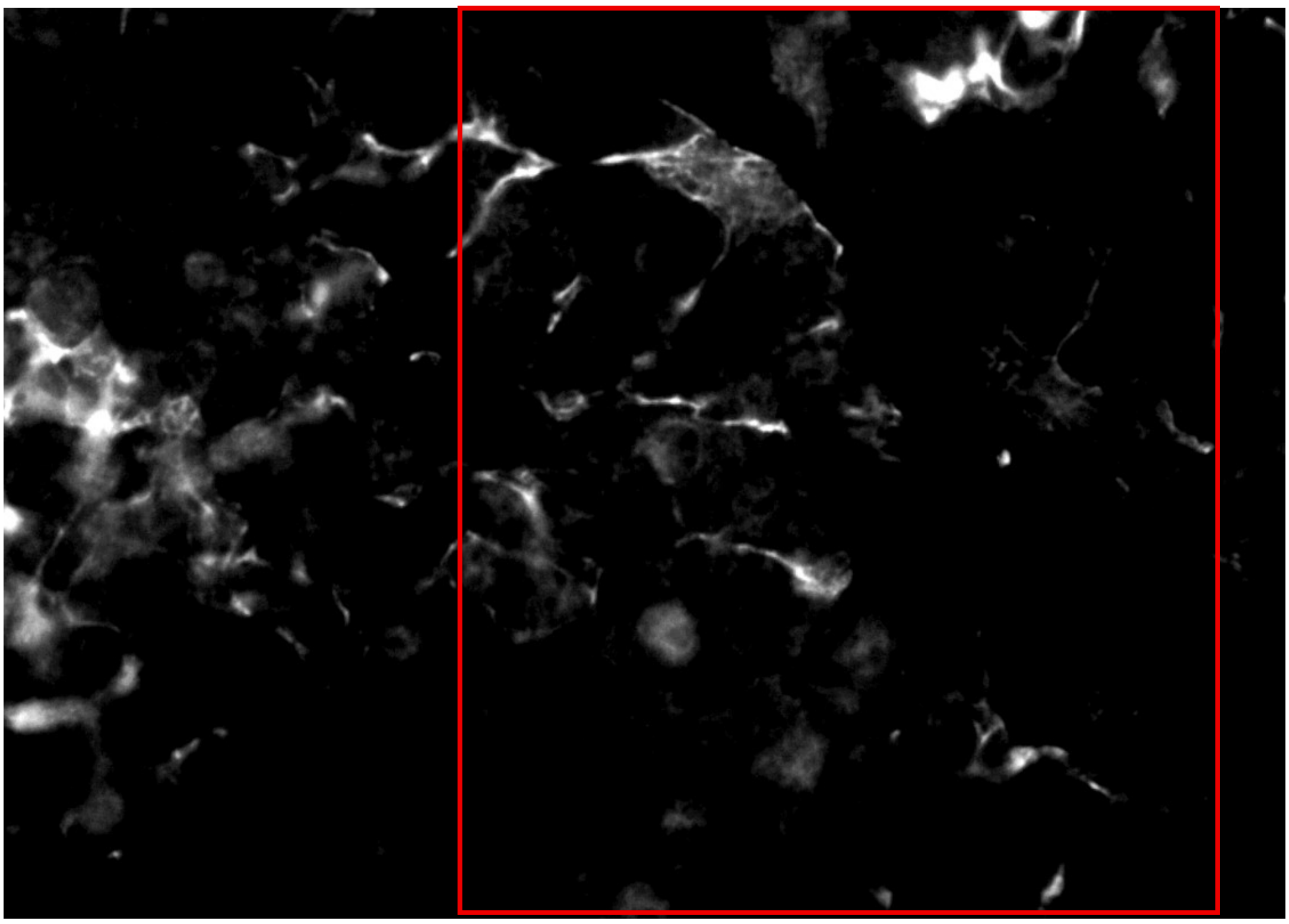

5D right KRT8

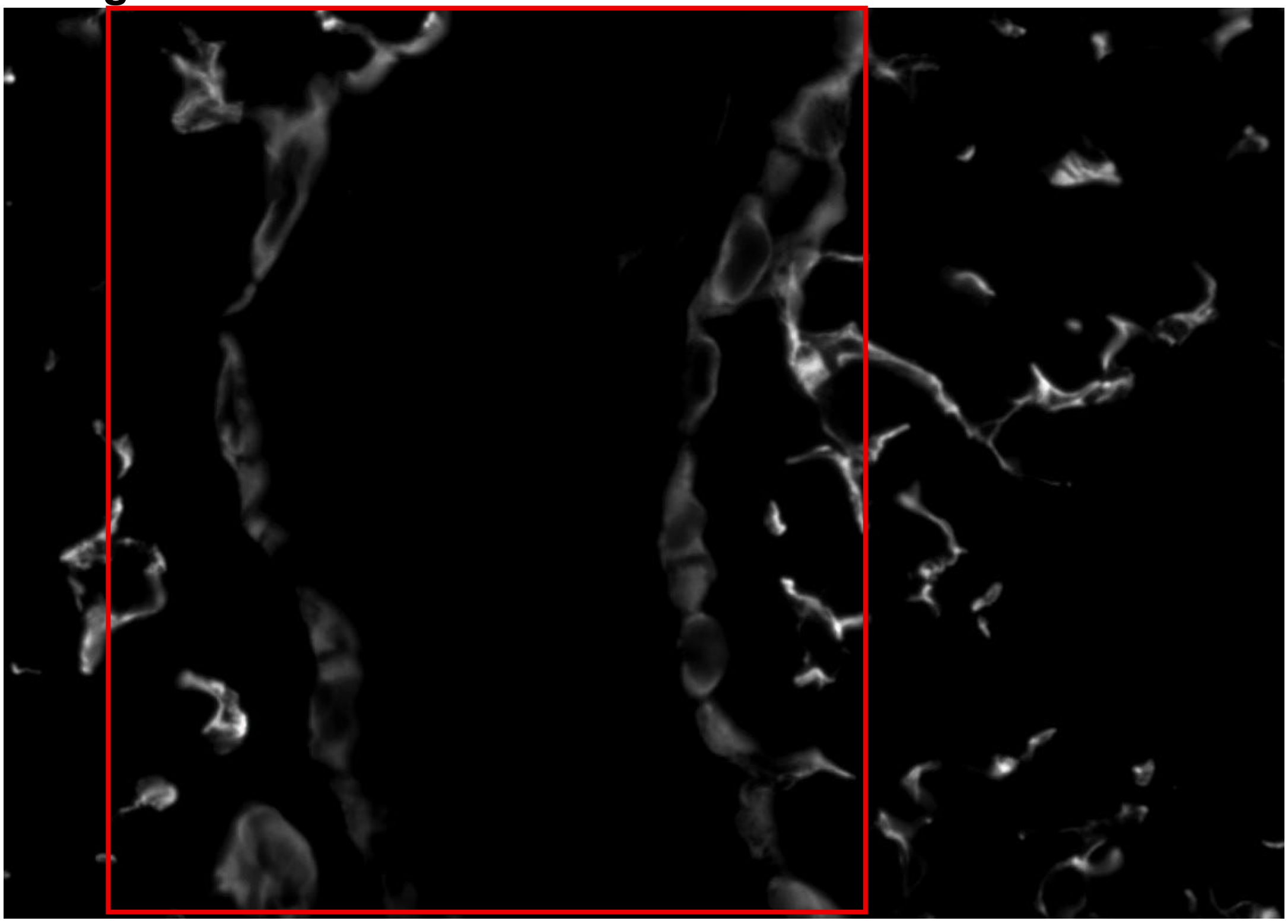

5E KRT8

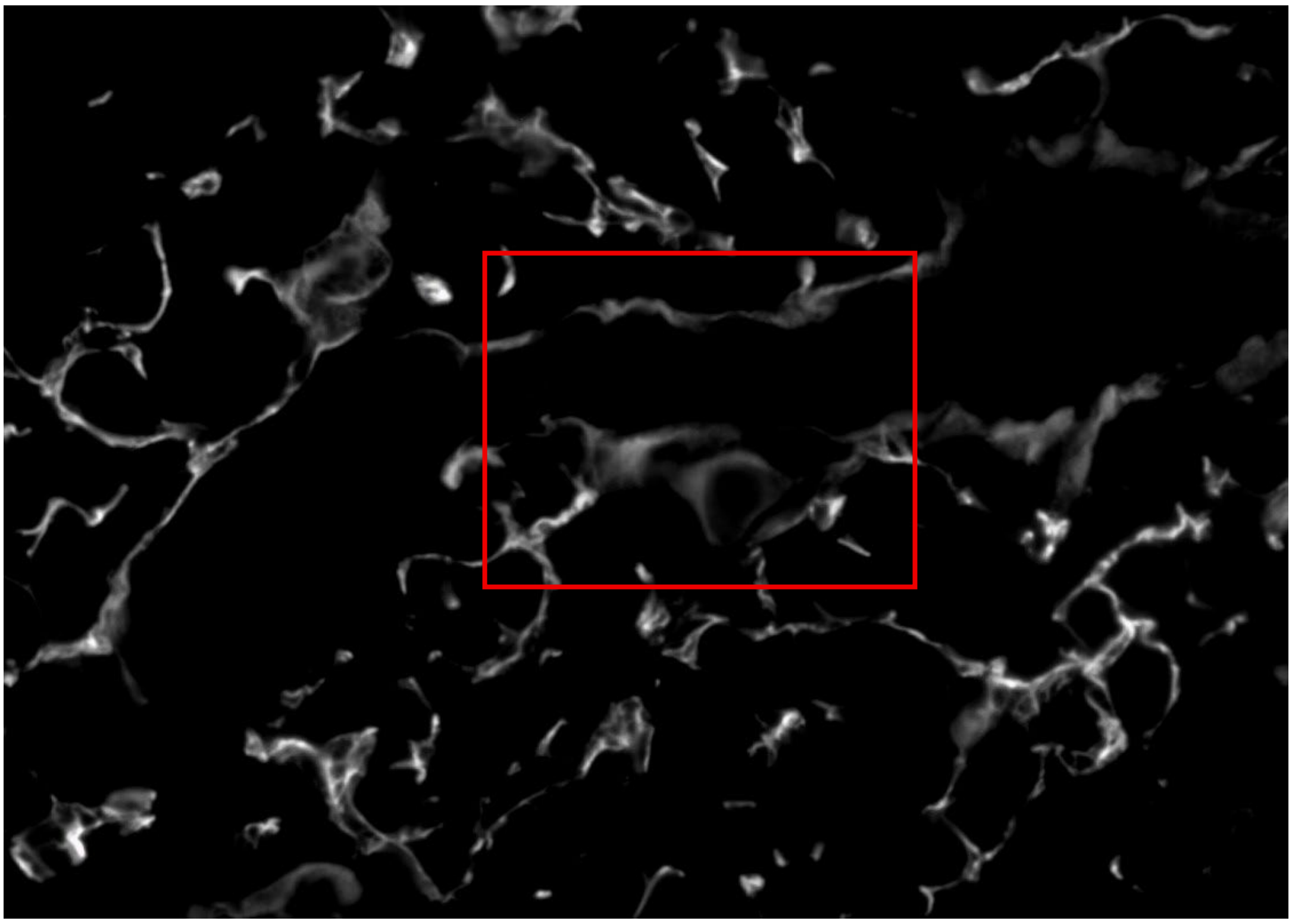

5F left KRT8

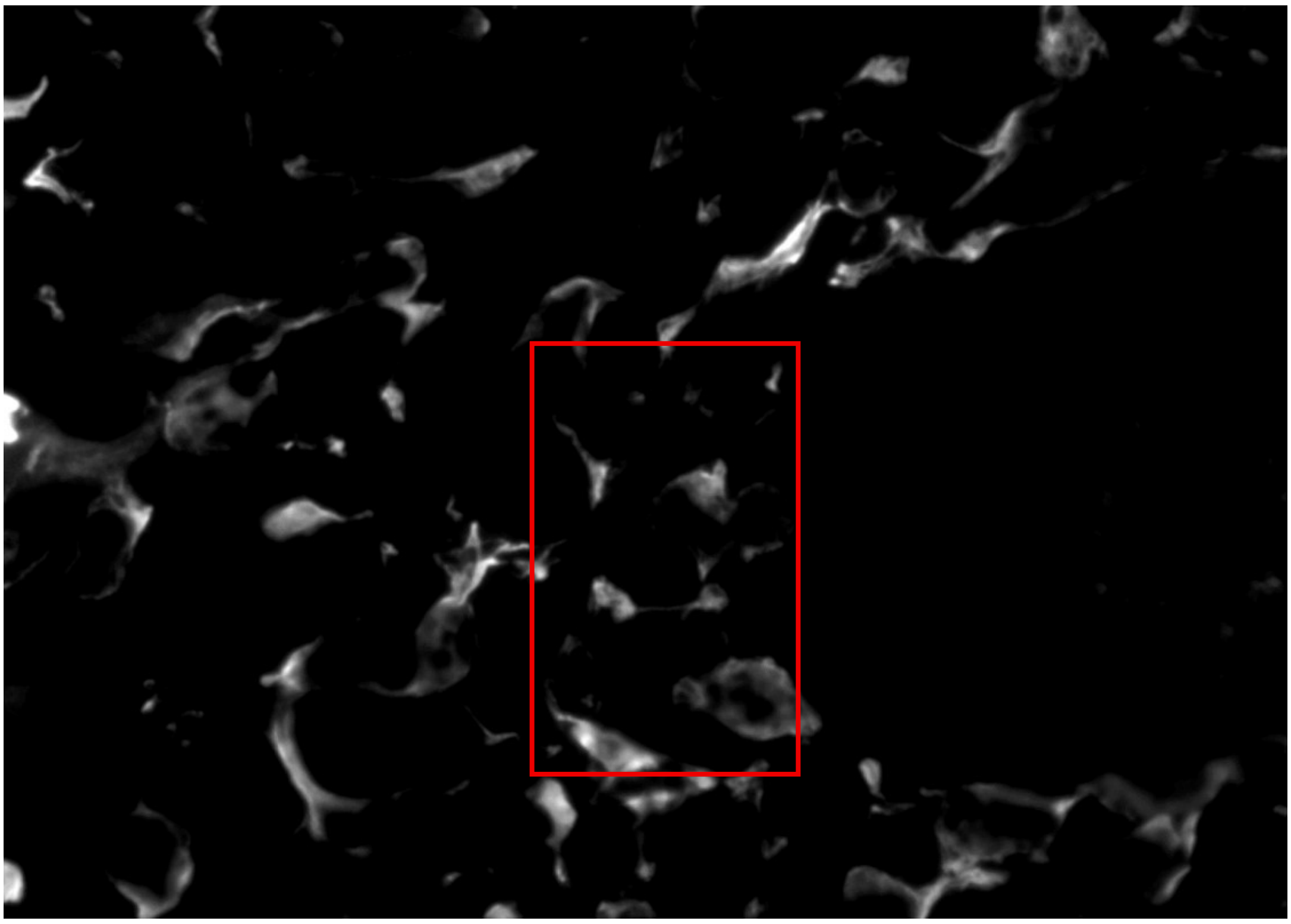

5F middle KRT8

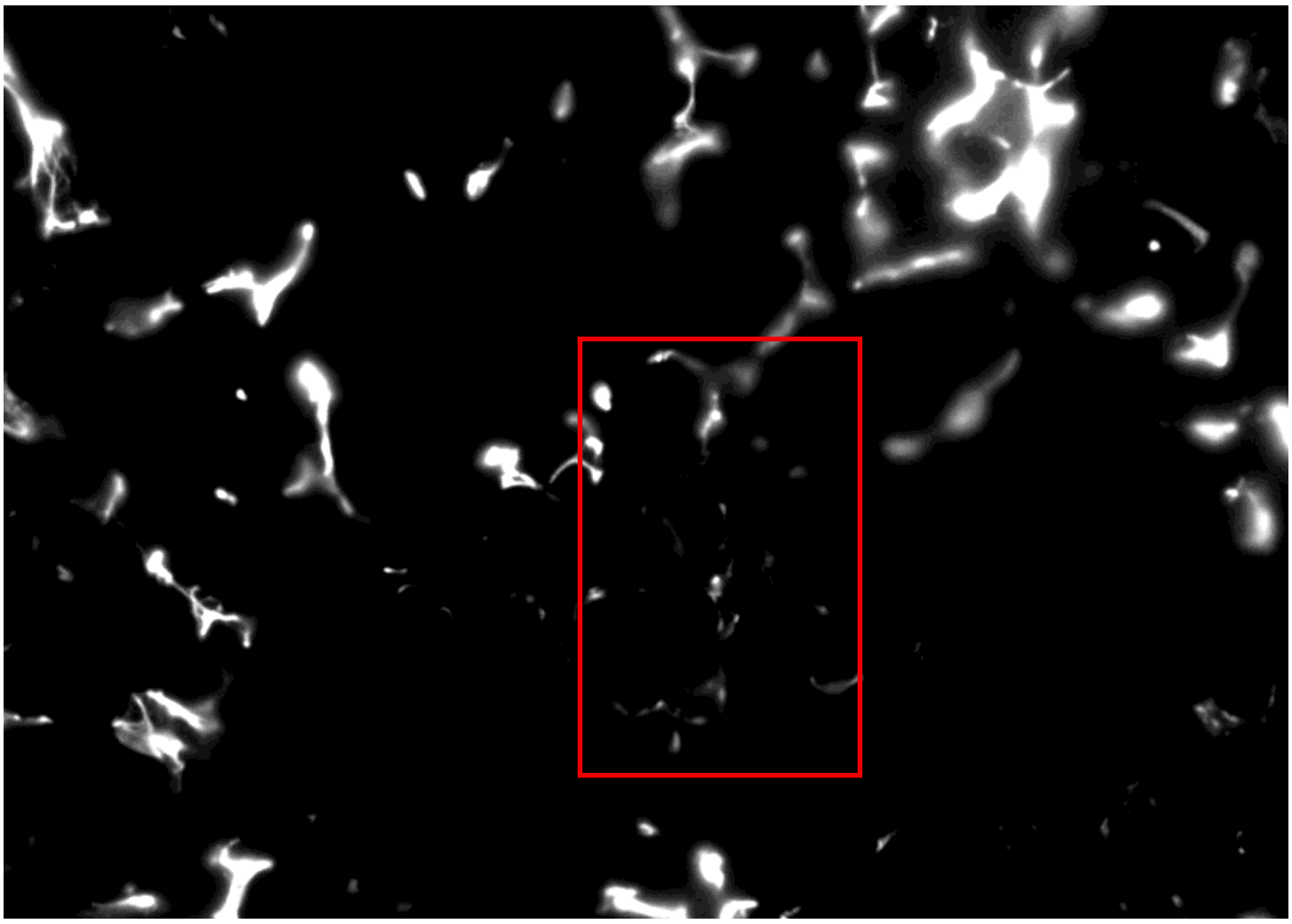

5F right KRT8

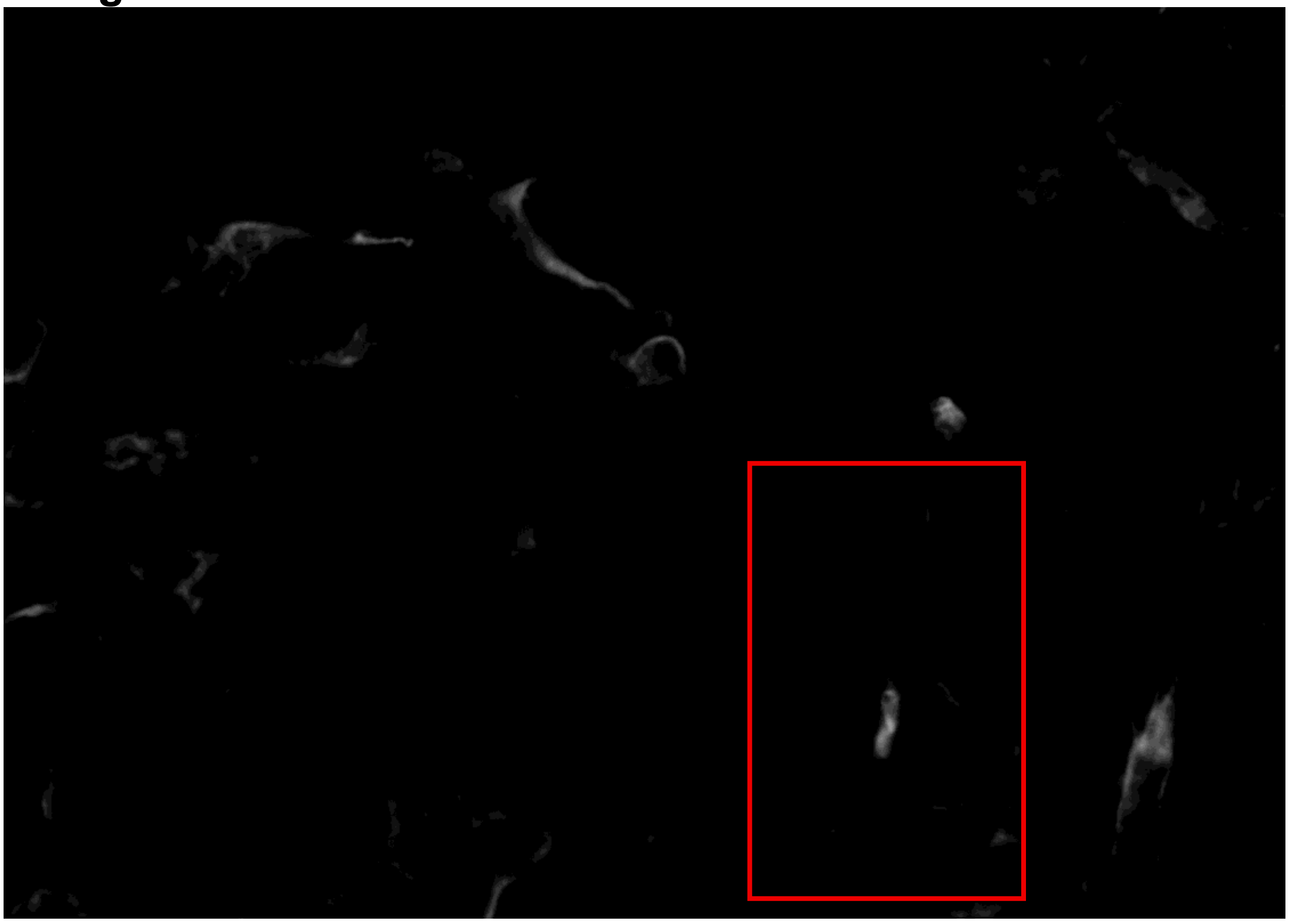

5G KRT8

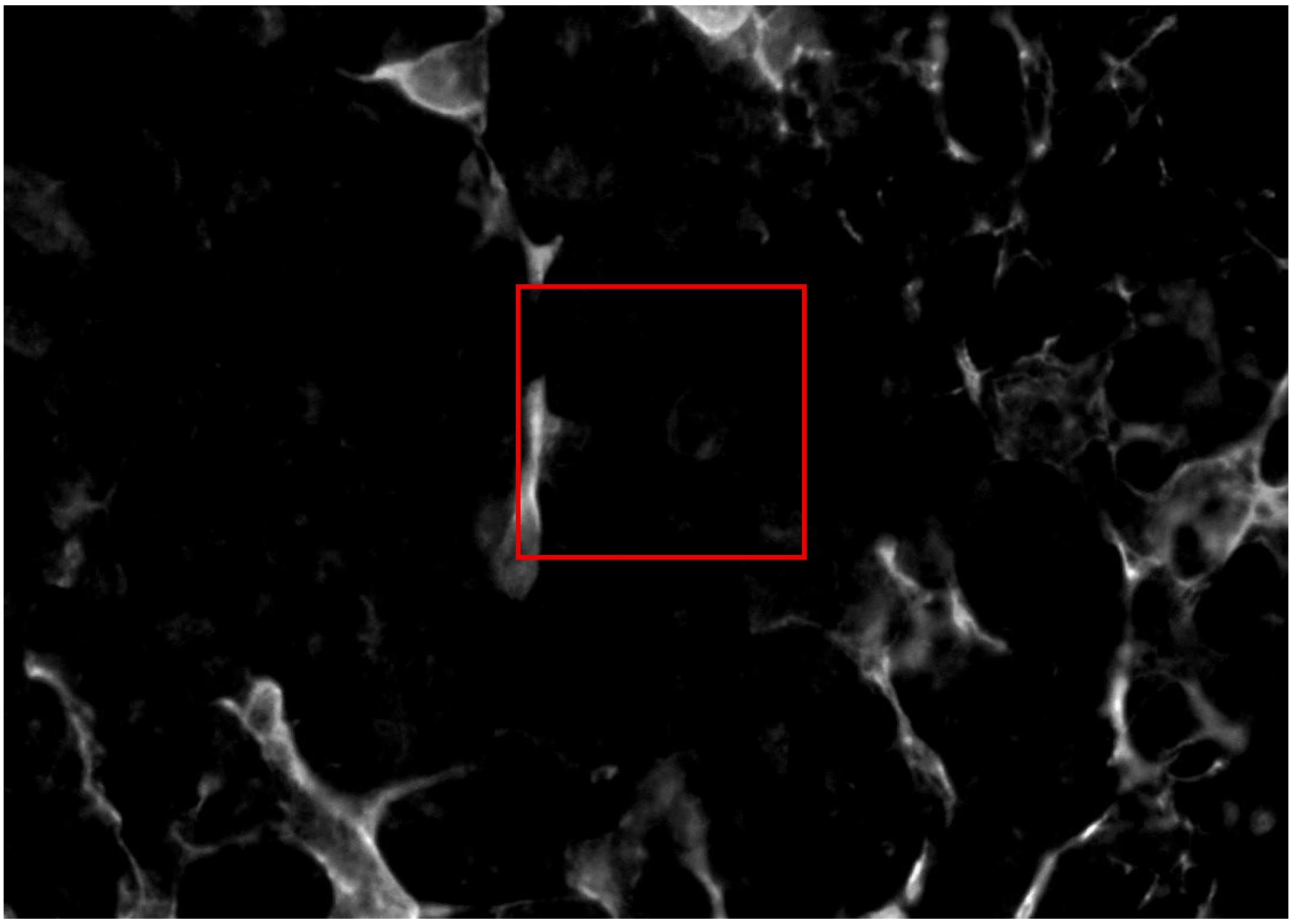

5H

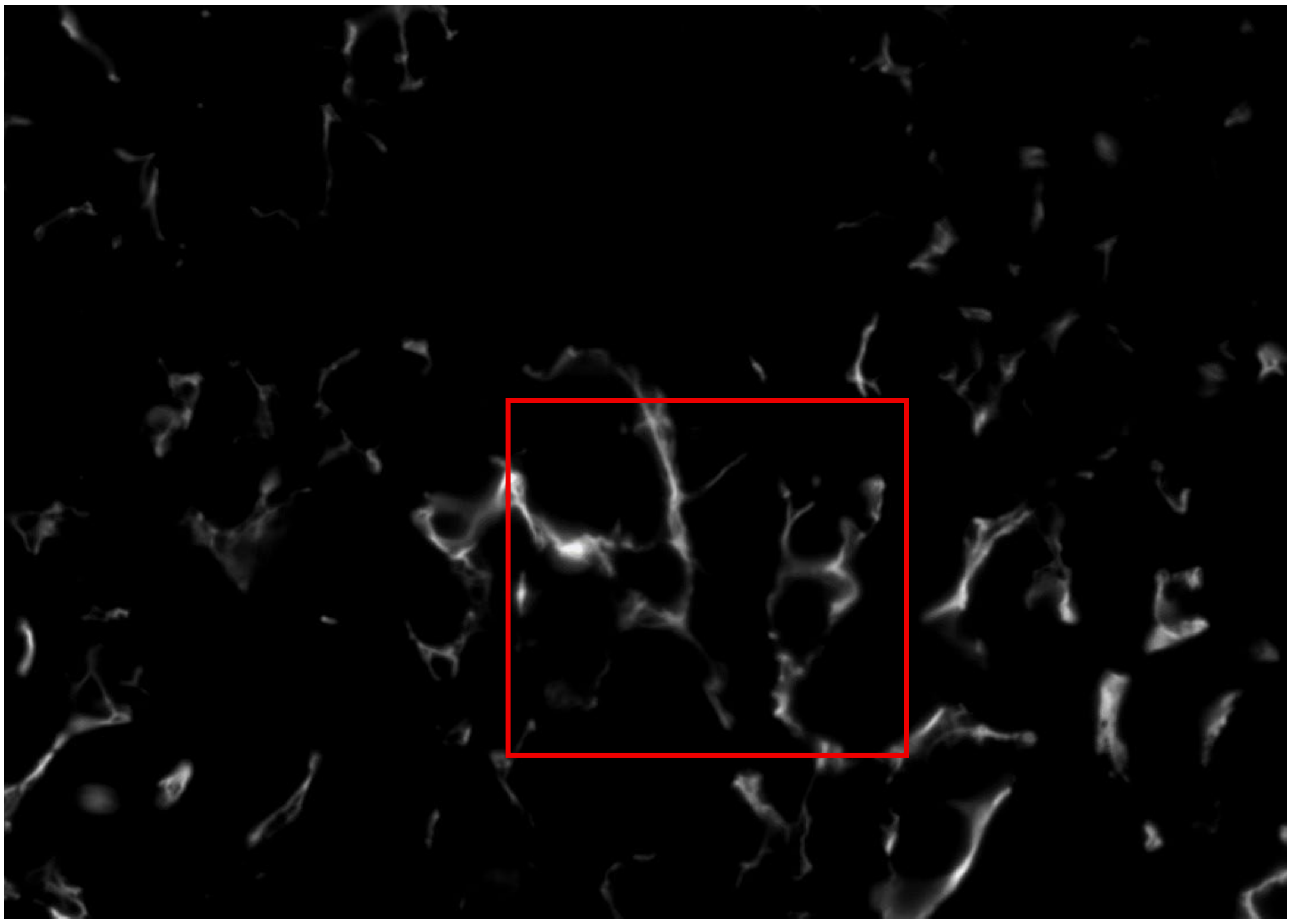

5I left

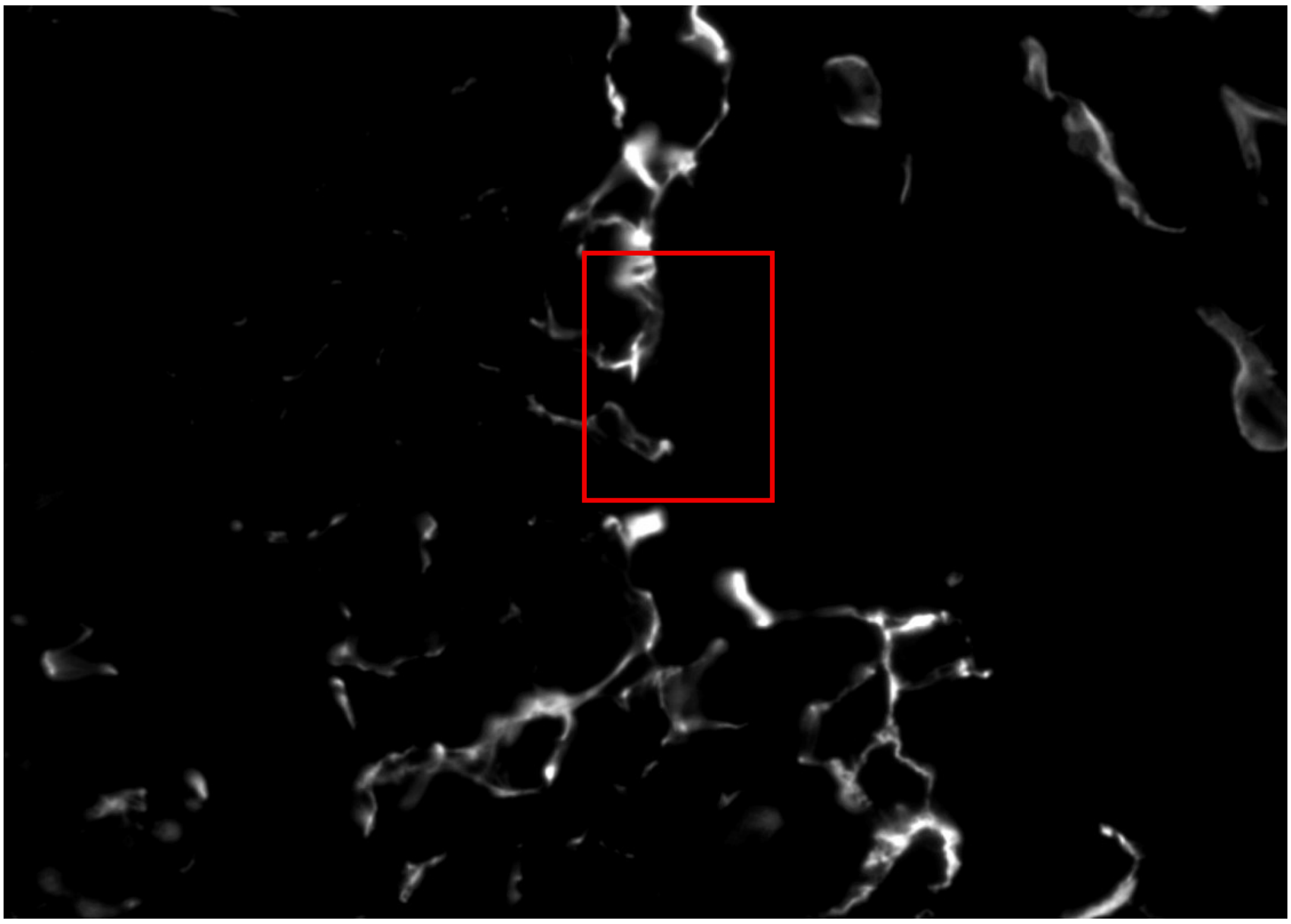

5I upper right KRT8

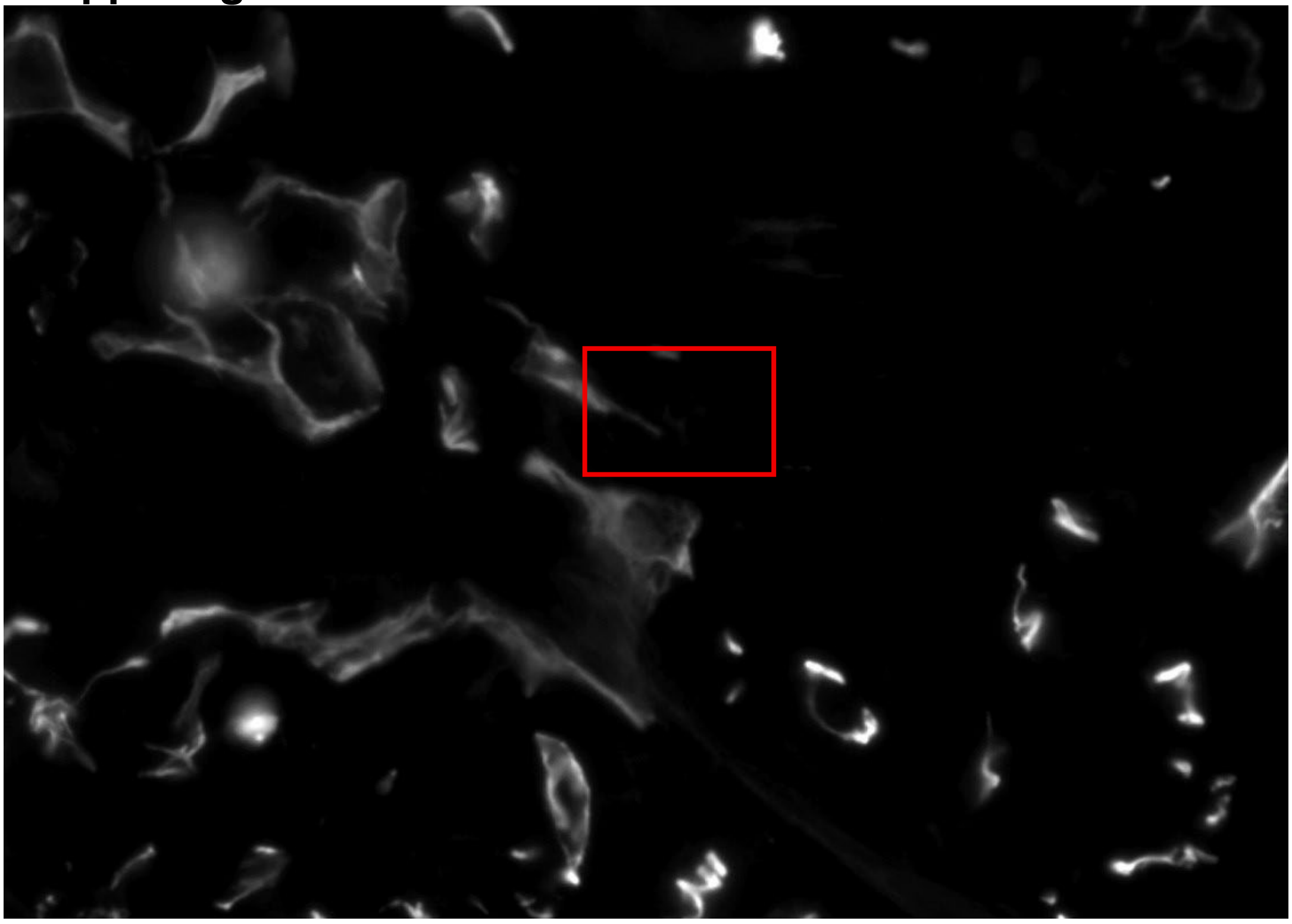

5I lower right KRT8

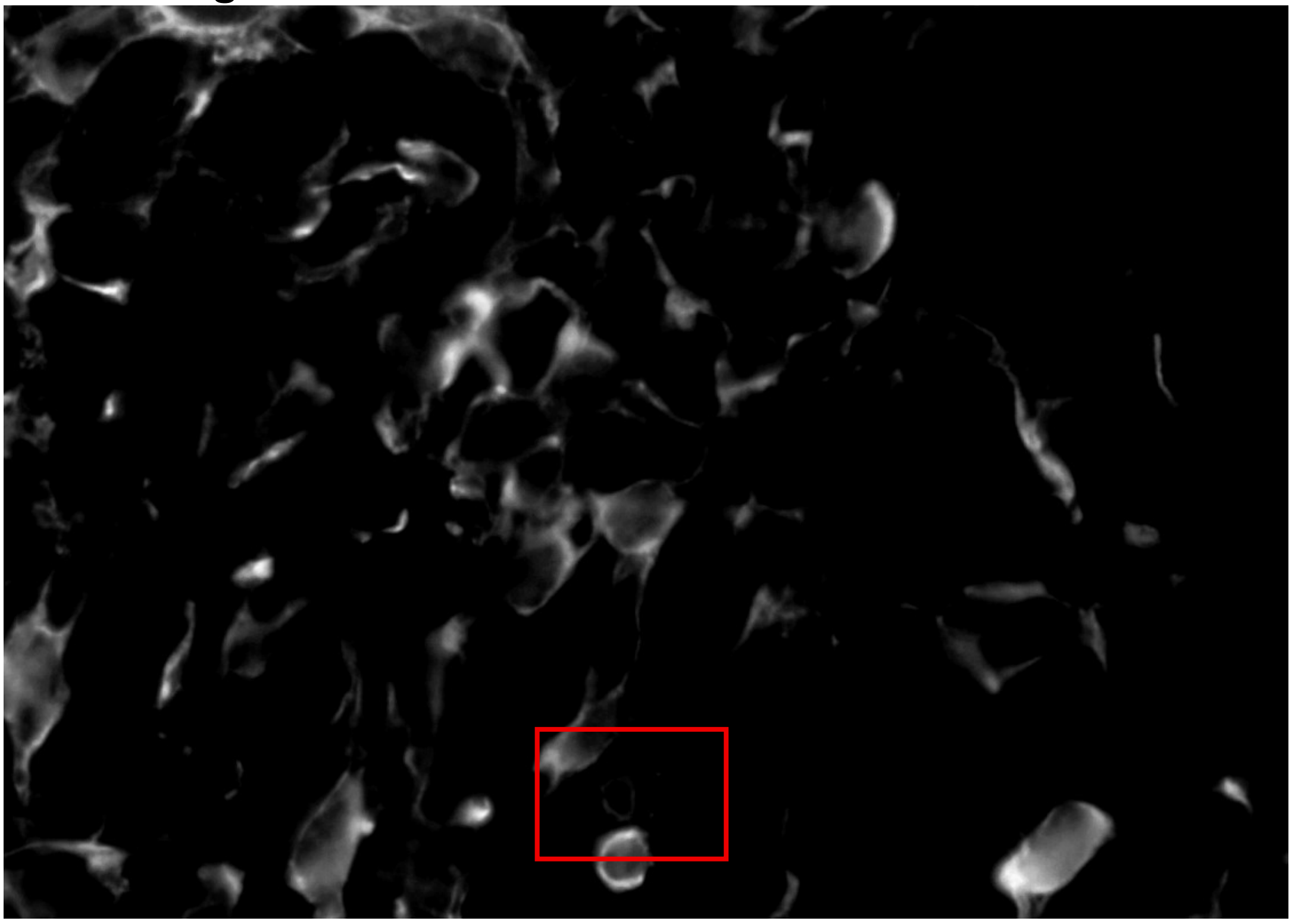

5J left KRT8

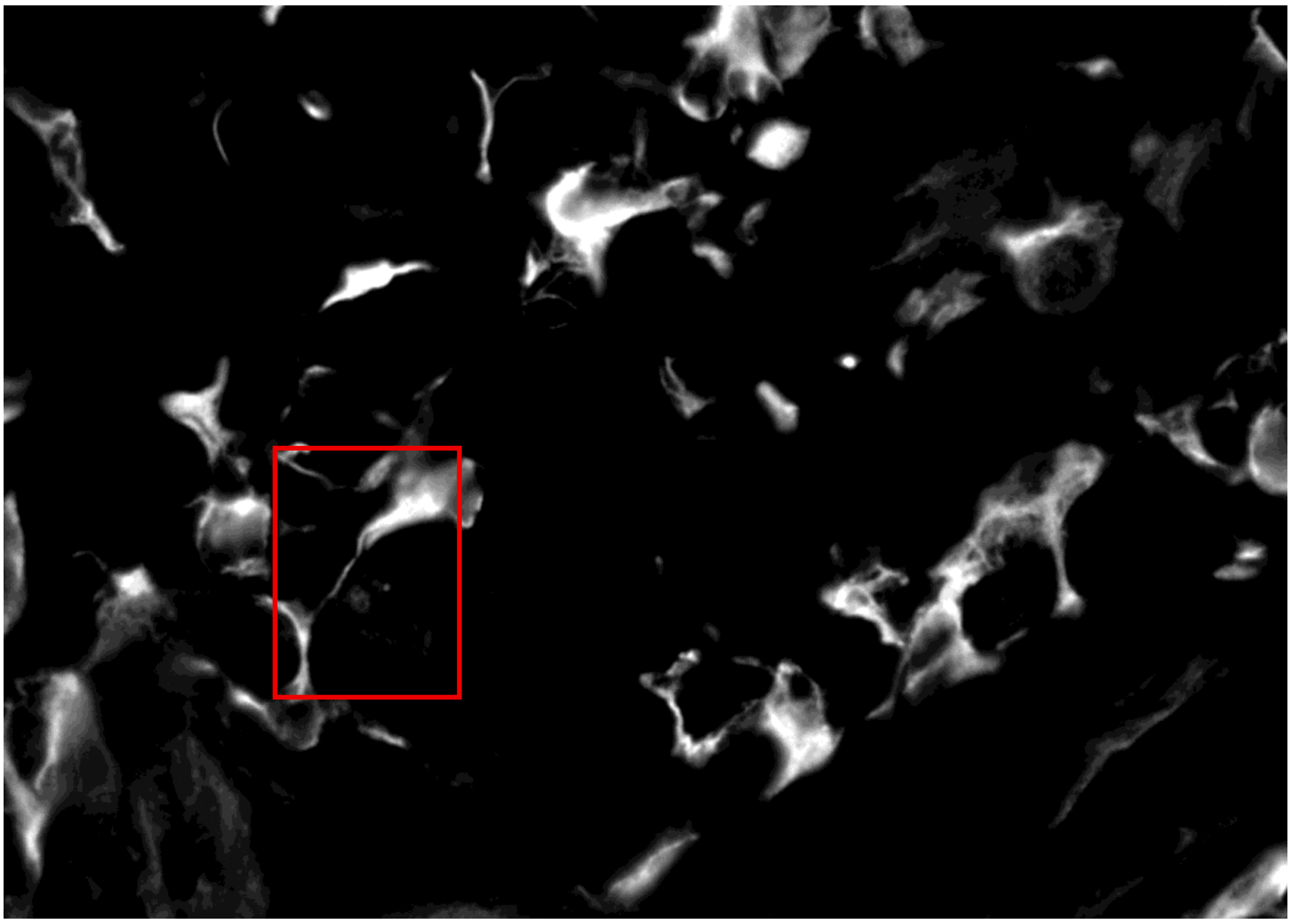

5J right KRT8

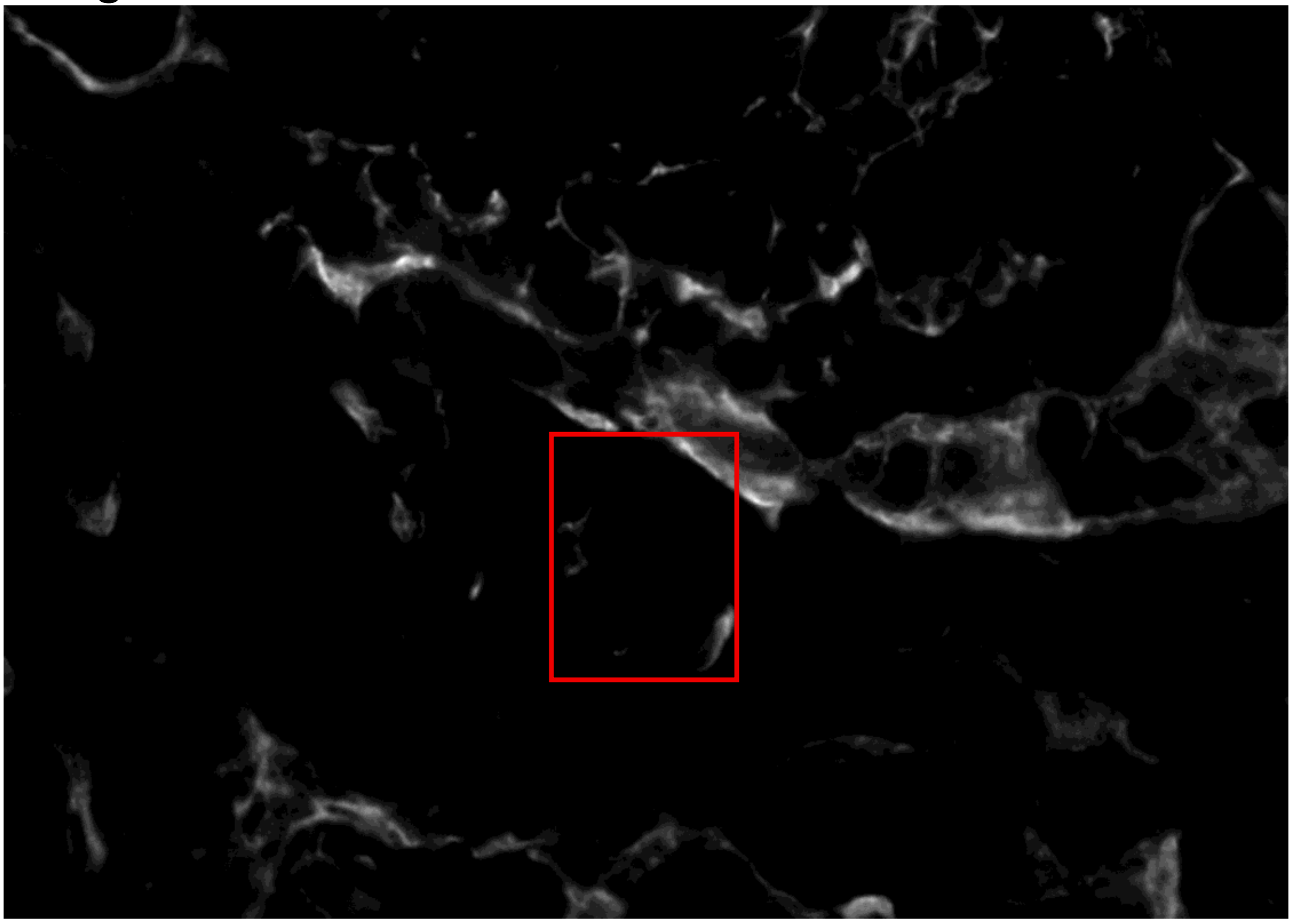

5K left KRT8

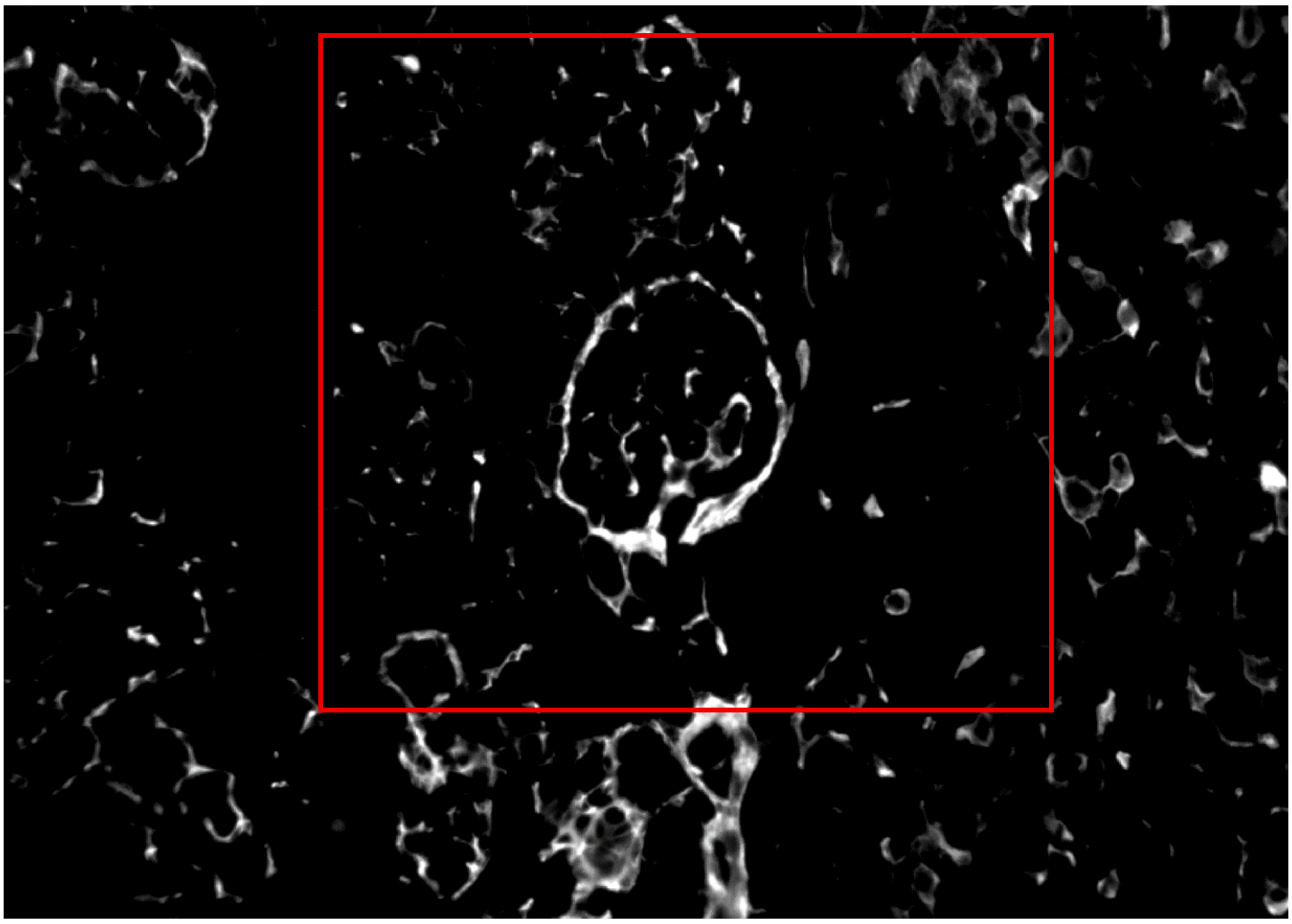

5K right KRT8

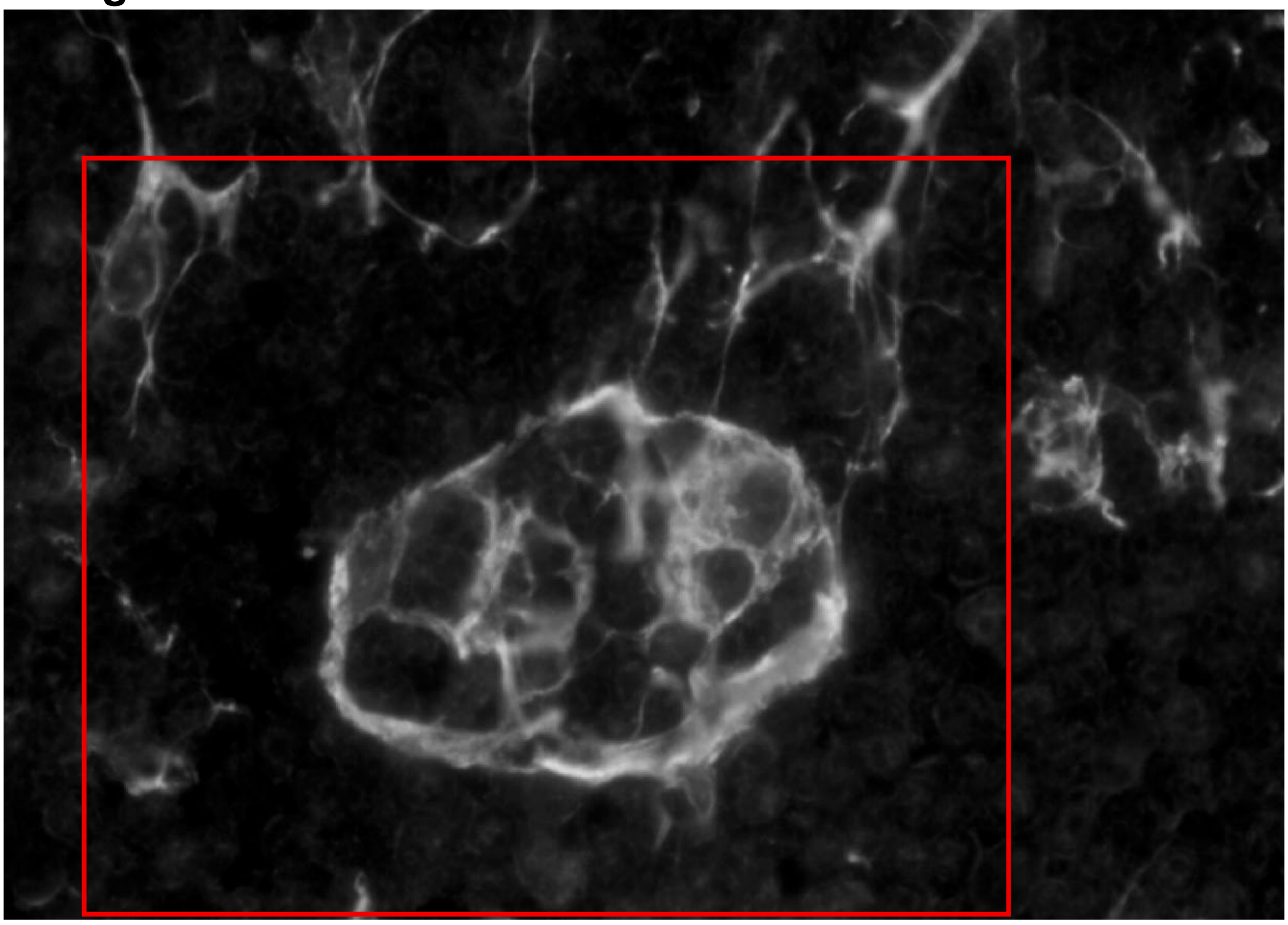

DAPI signal

5D left

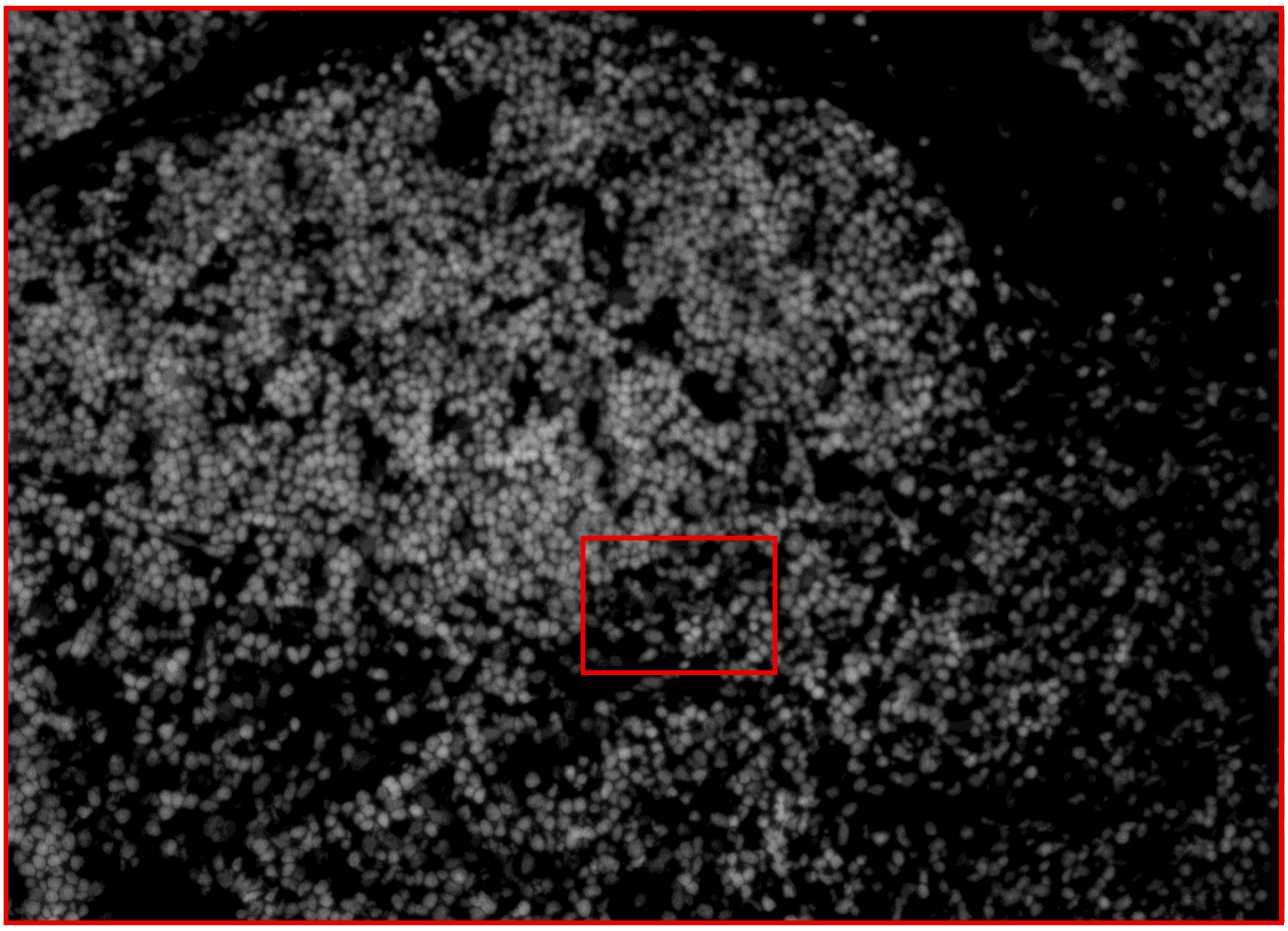

5D middle

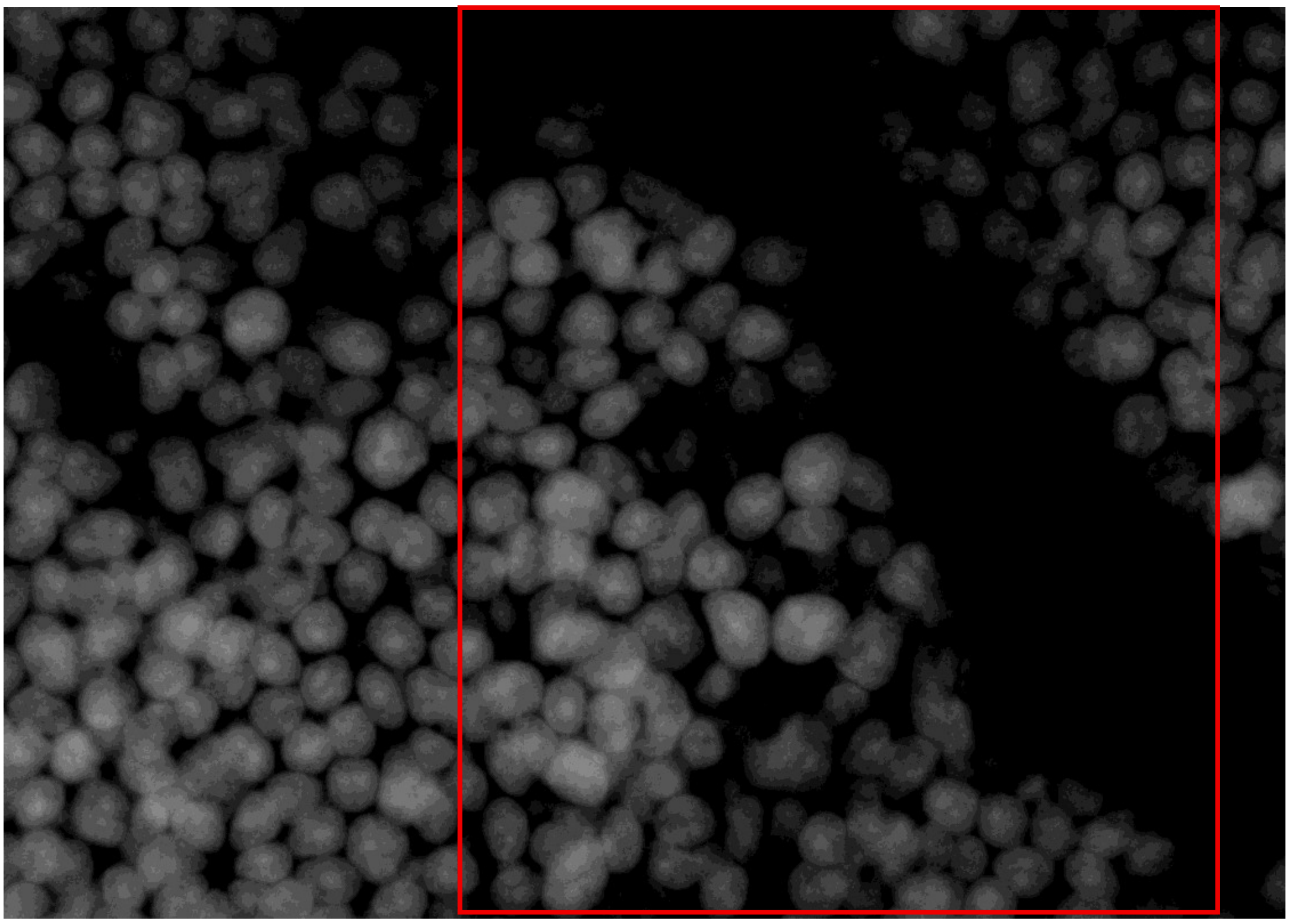

5D right DAPI

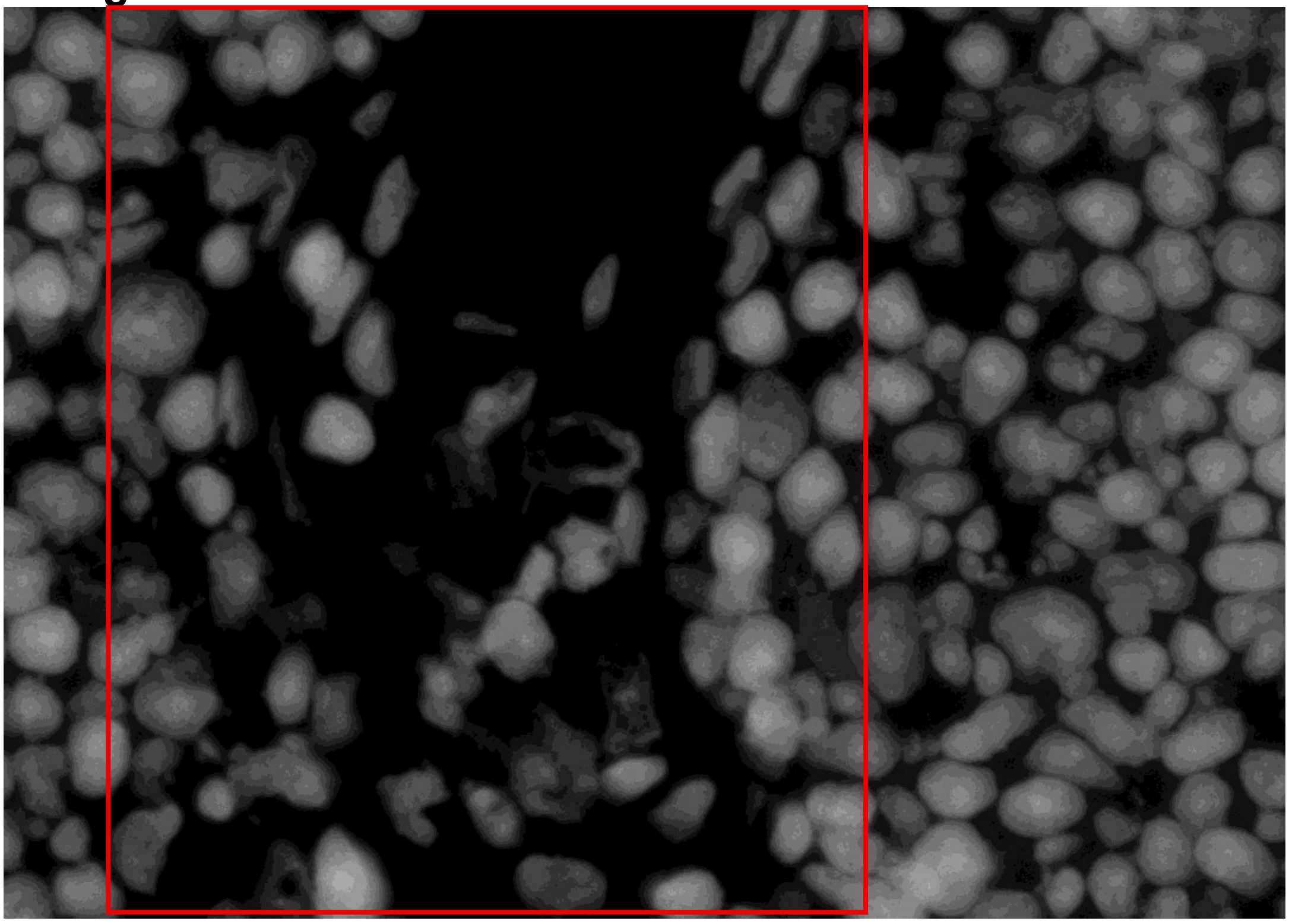

5E DAPI

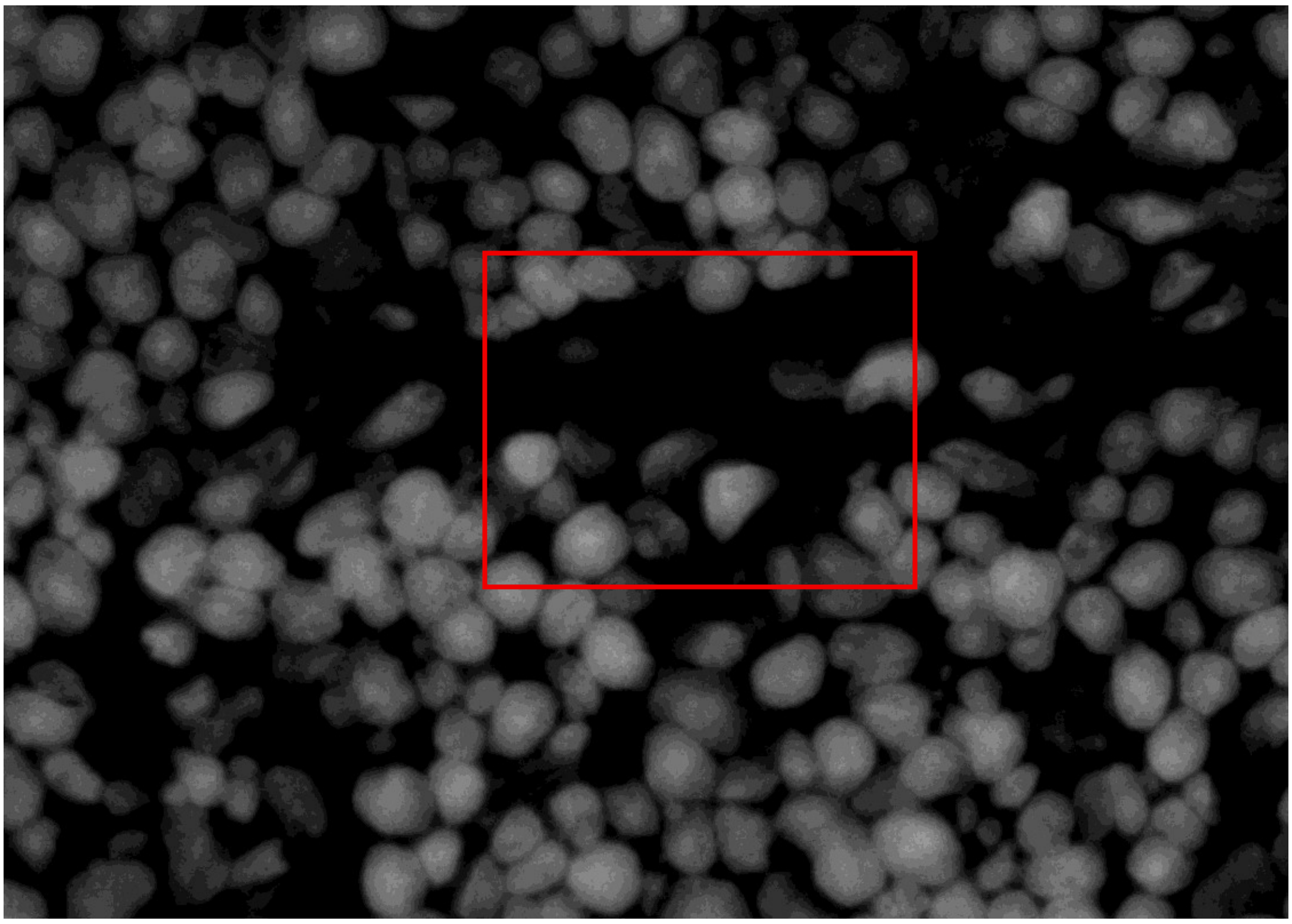

5F left DAPI

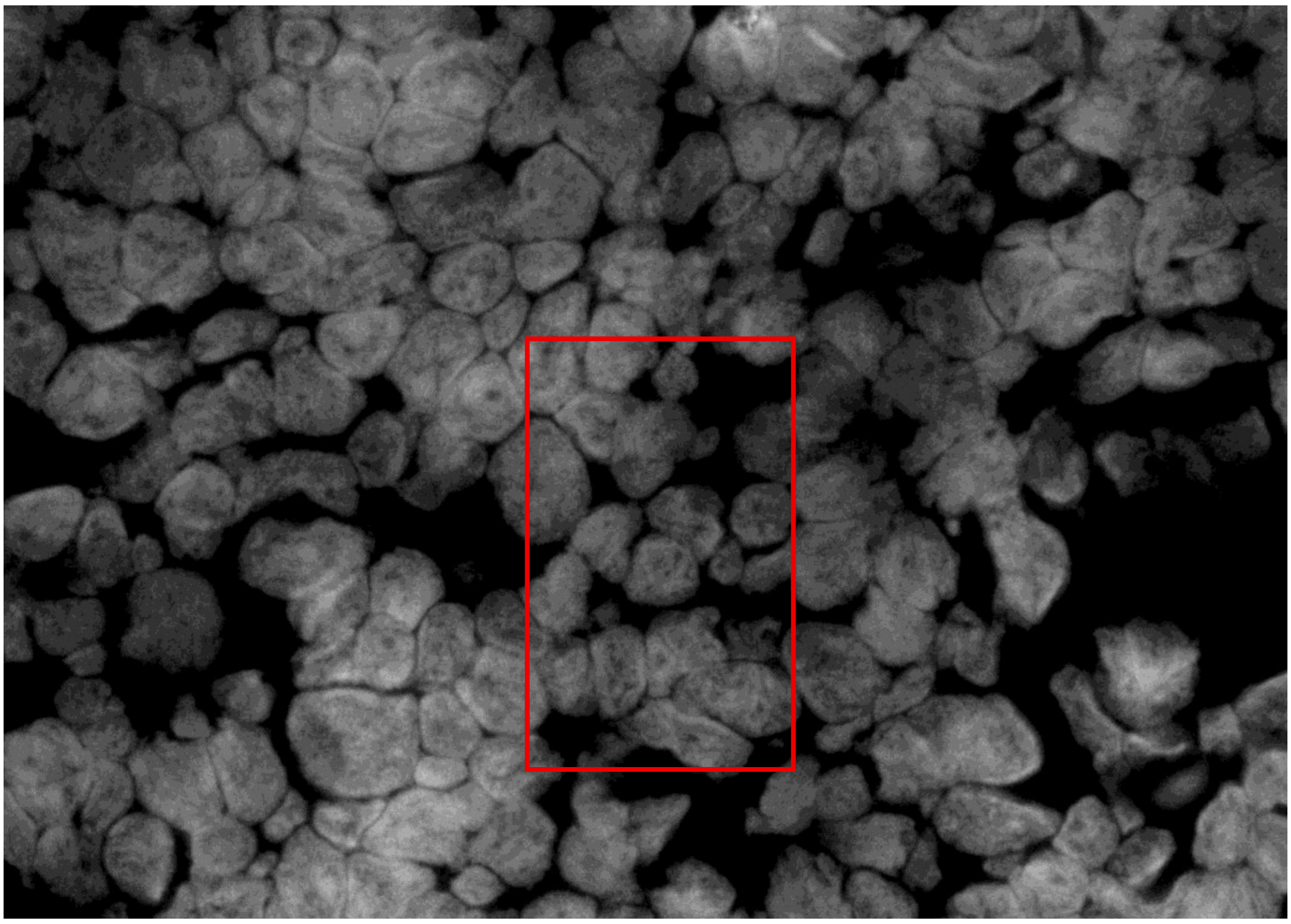

5F middle DAPI

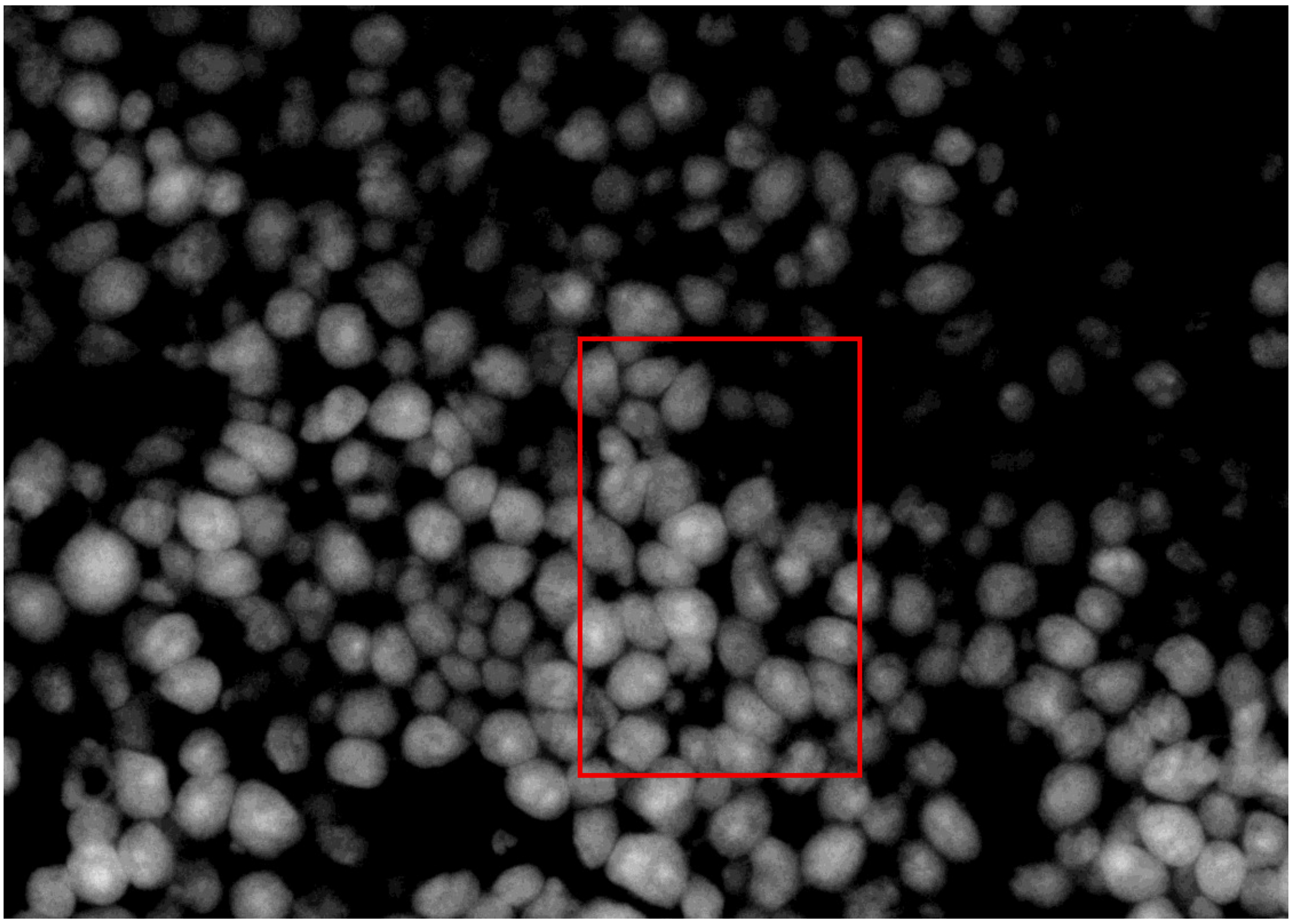

5F right DAPI

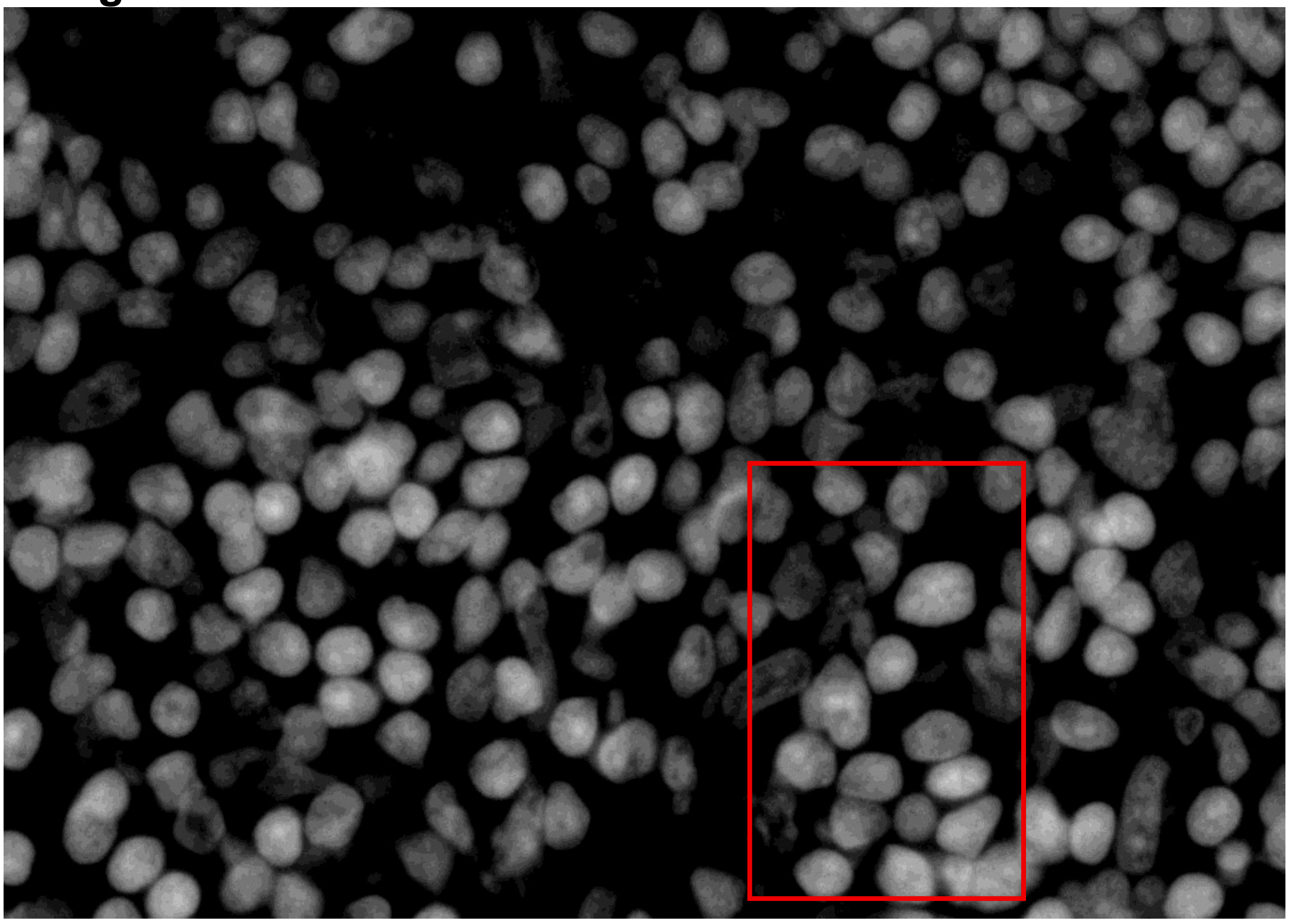

5G DAPI

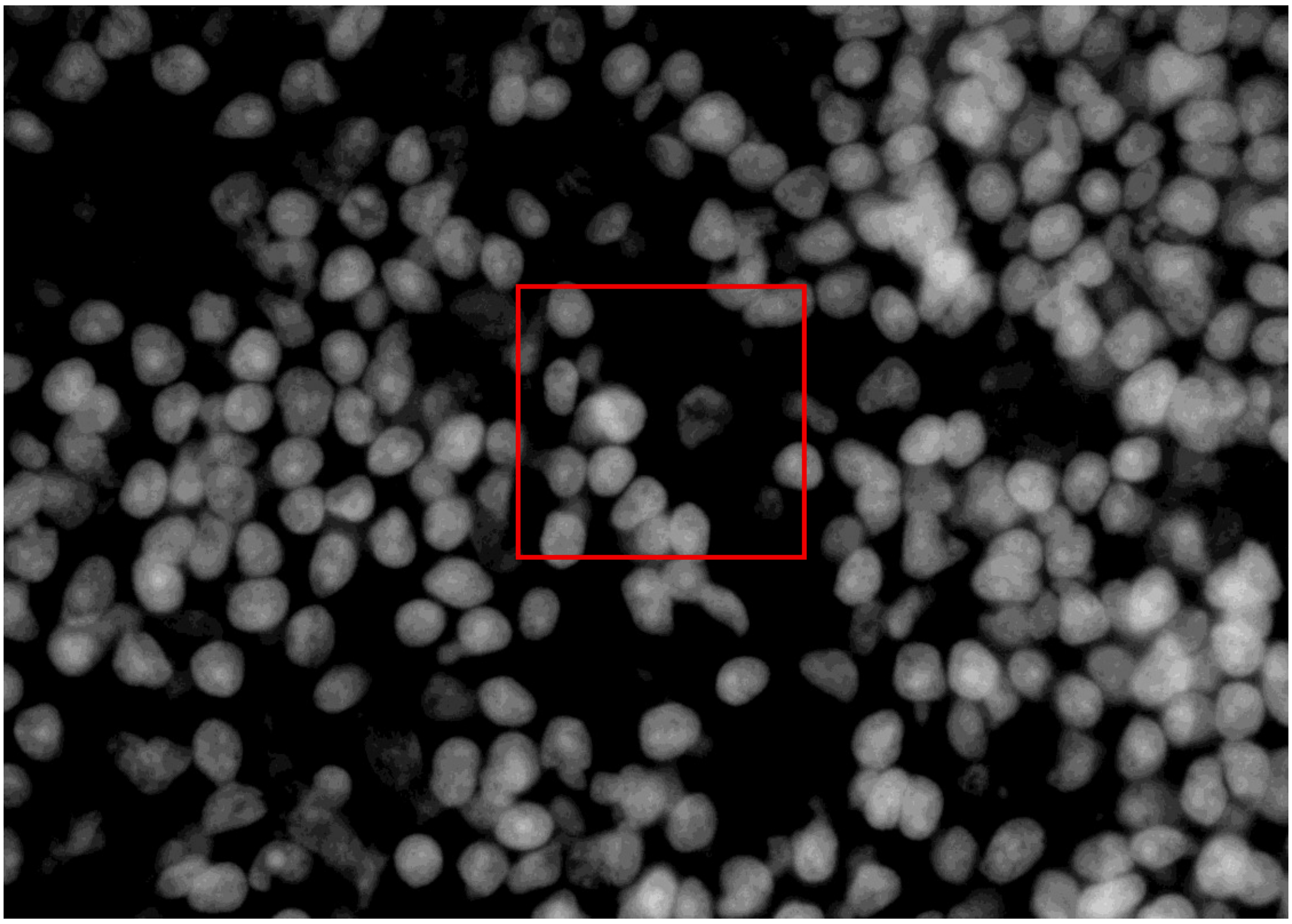

5H

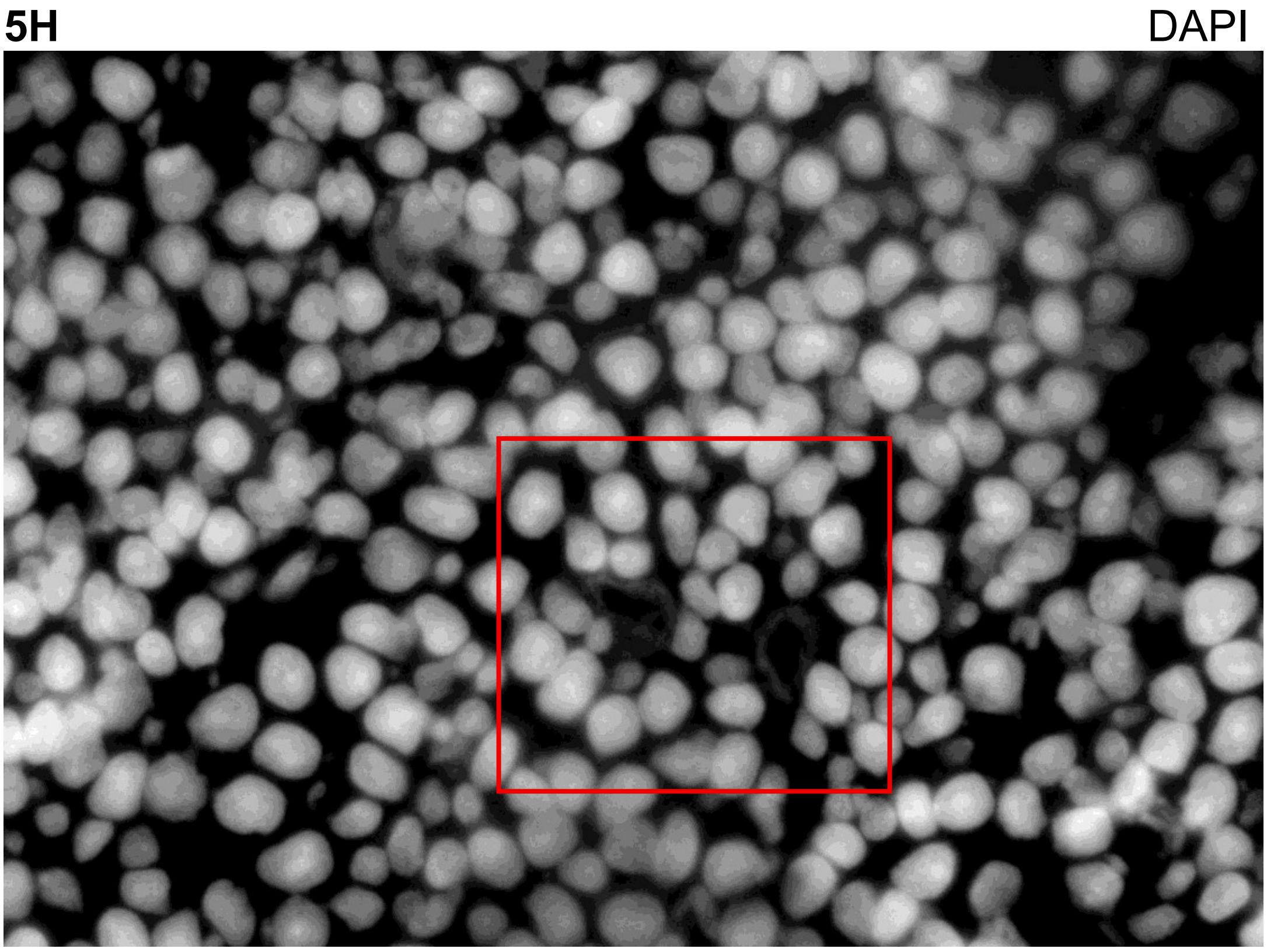

5I left

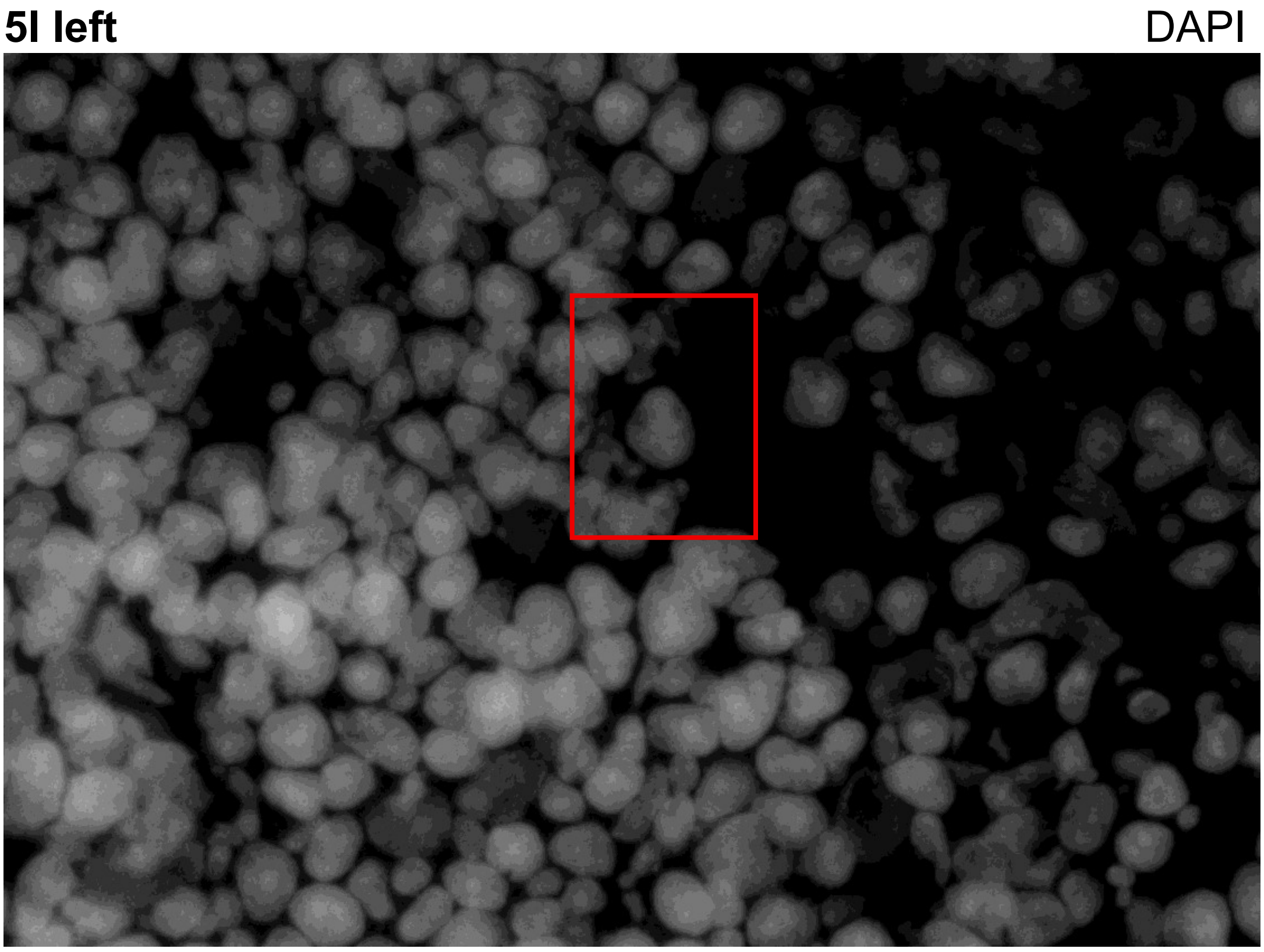

5I upper right

DAPI

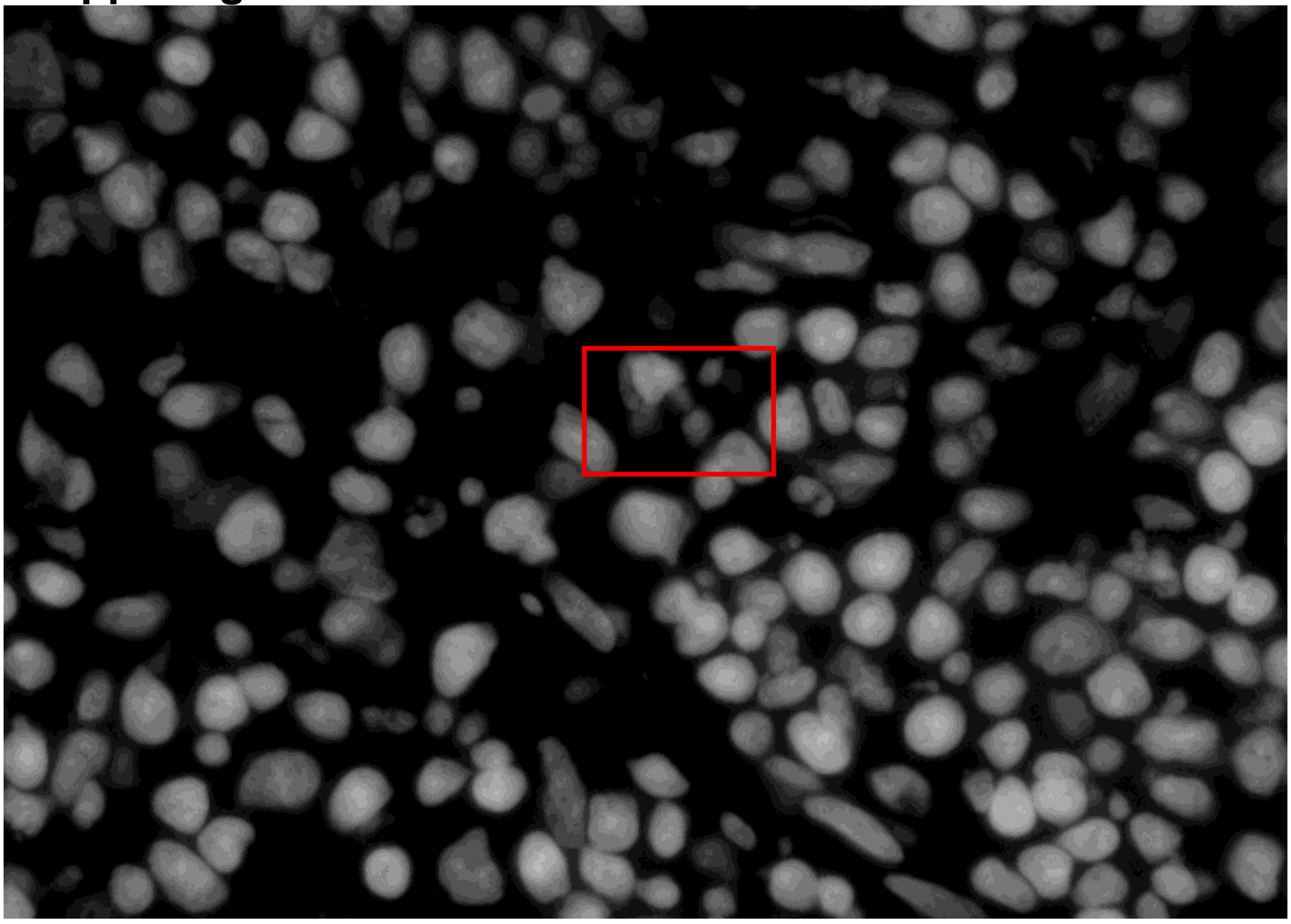

5I lower right

DAPI

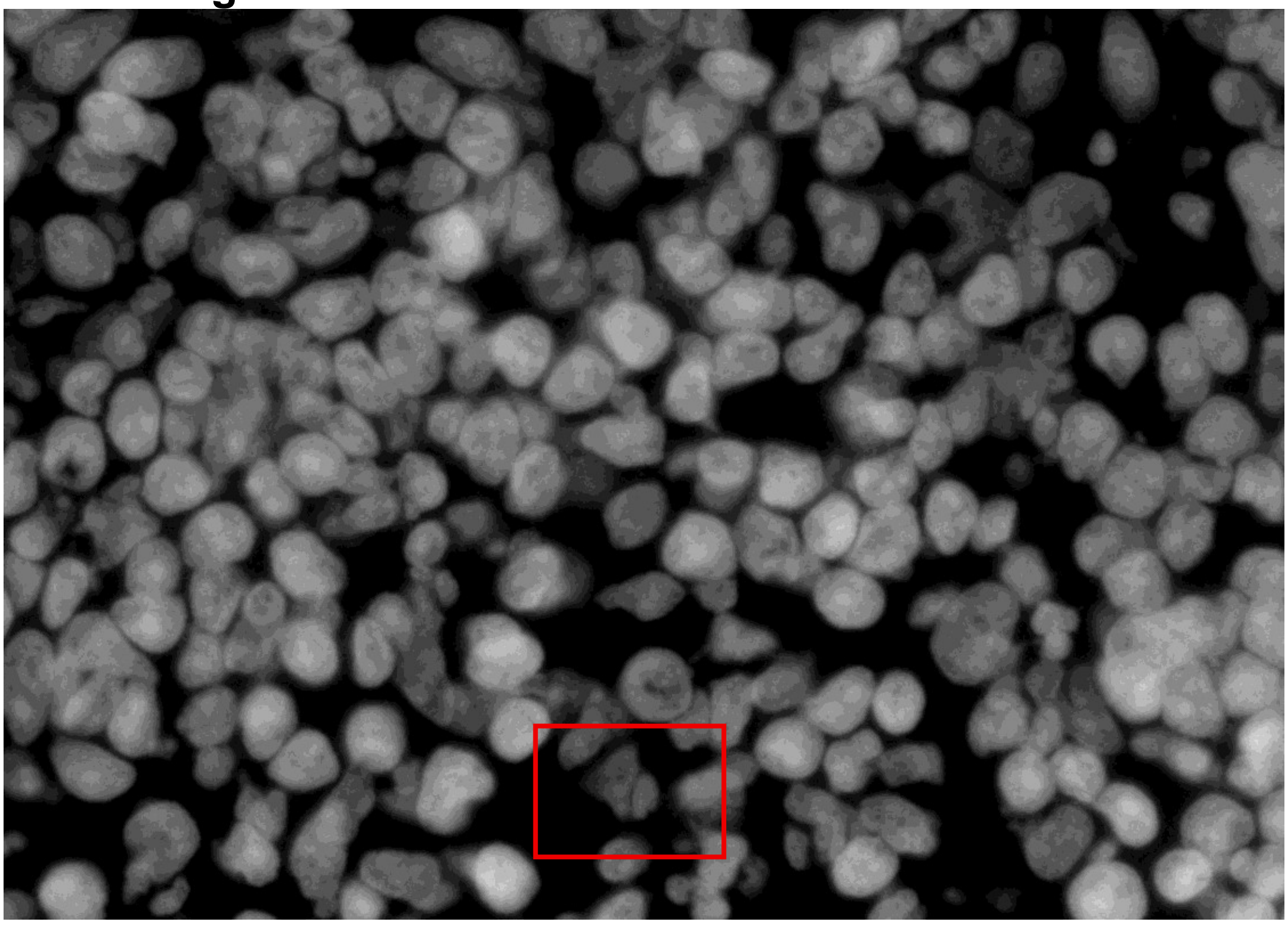

5J left DAPI

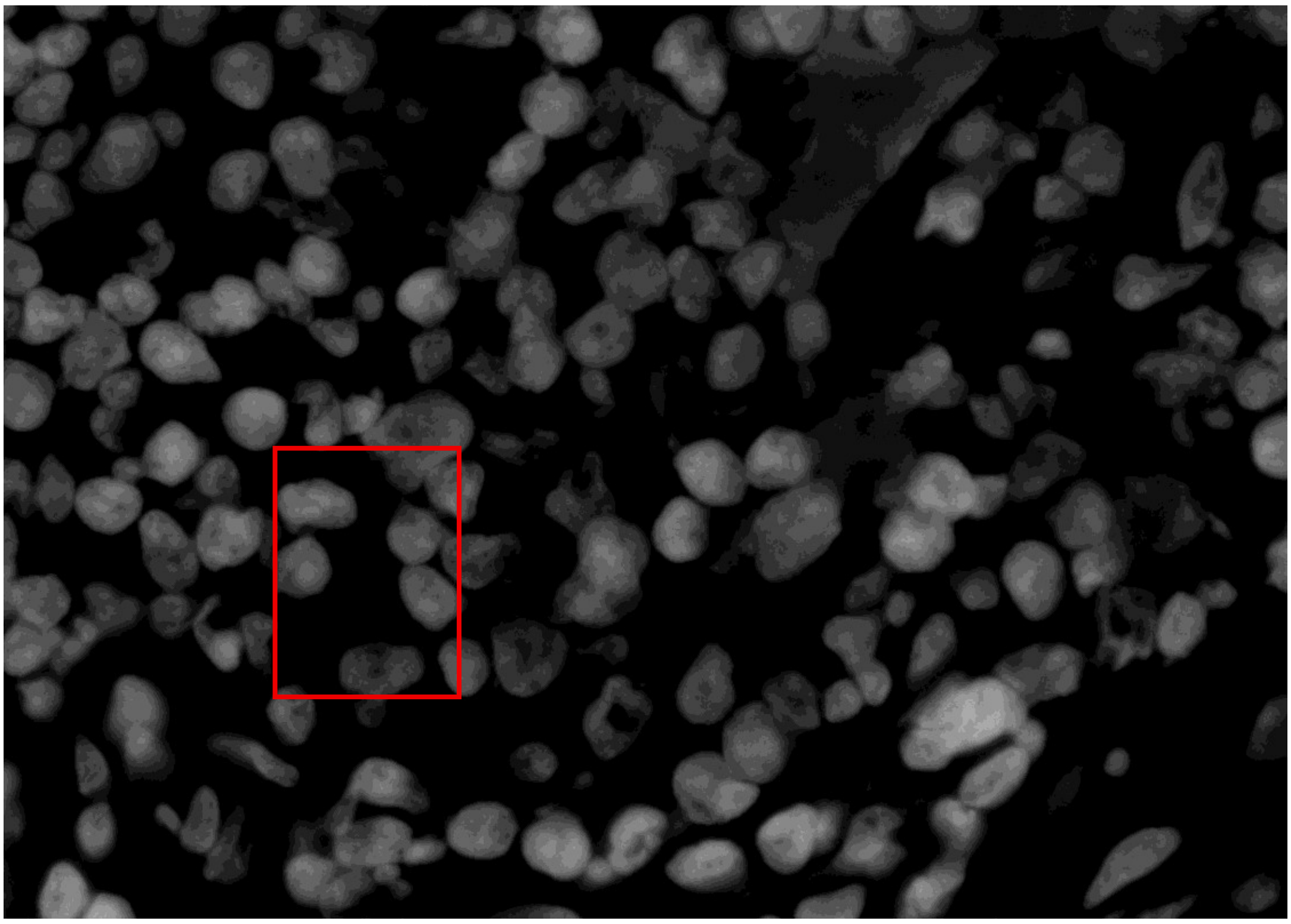

5J right DAPI

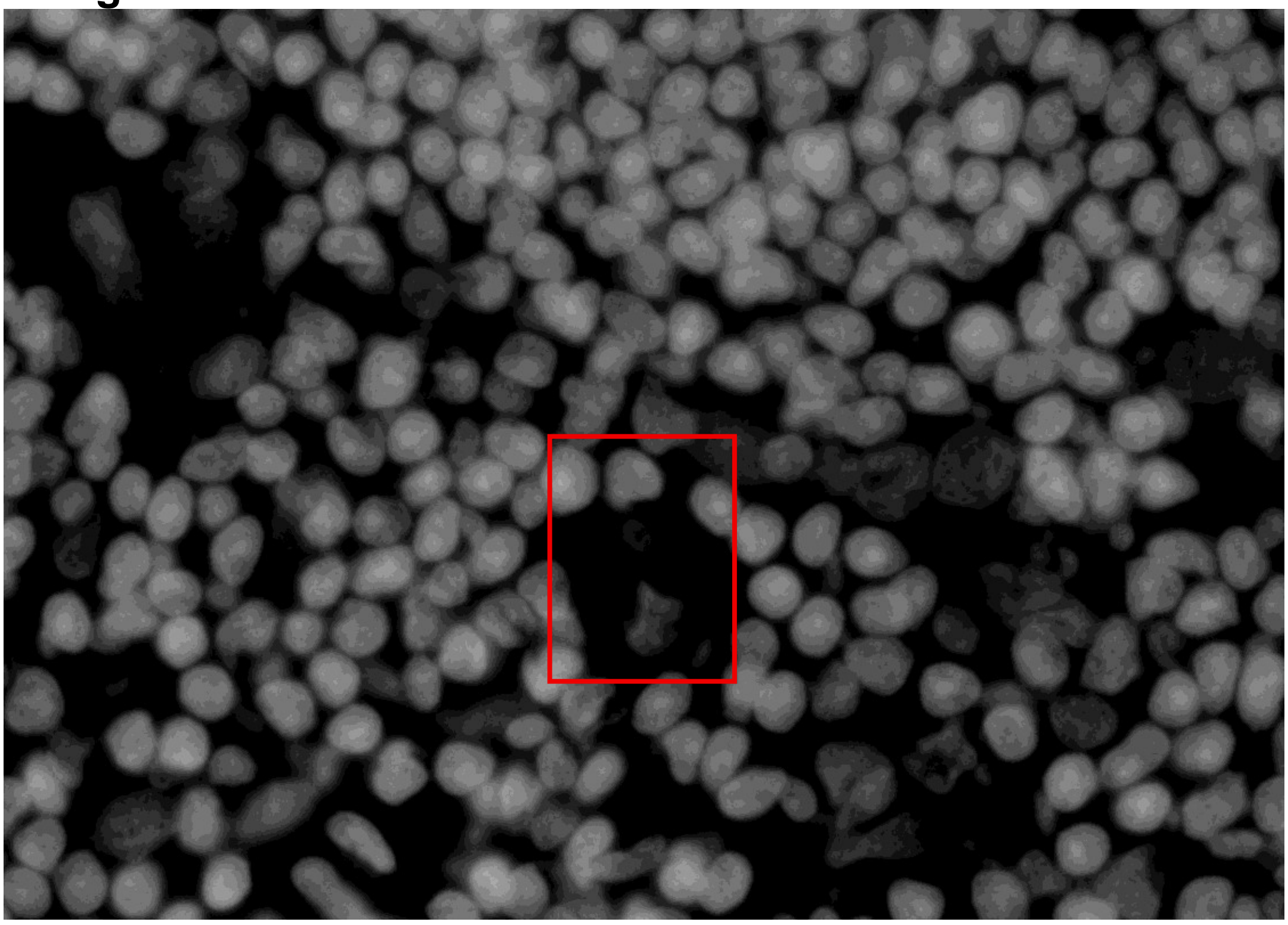

5K left DAPI

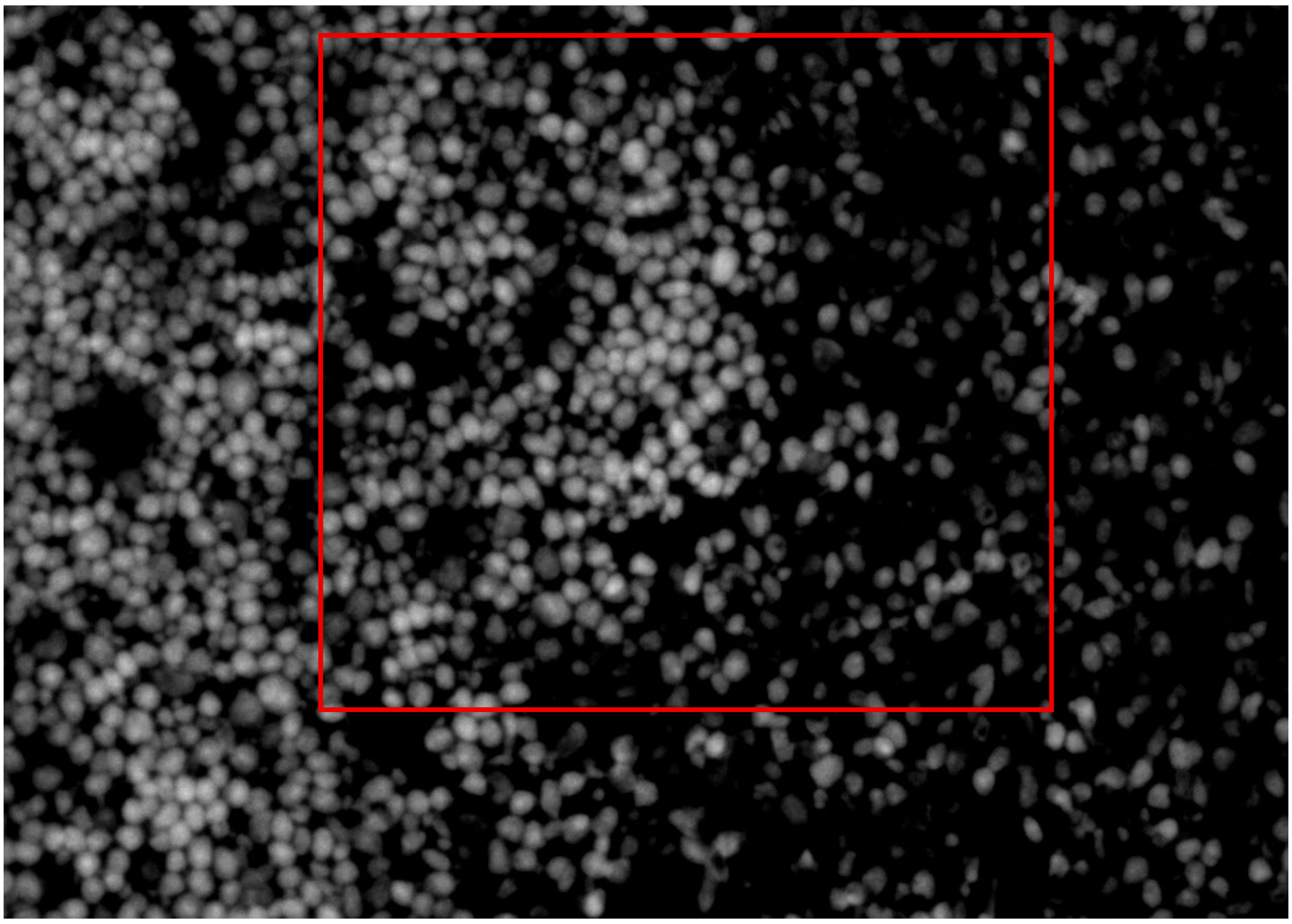

5K right DAPI

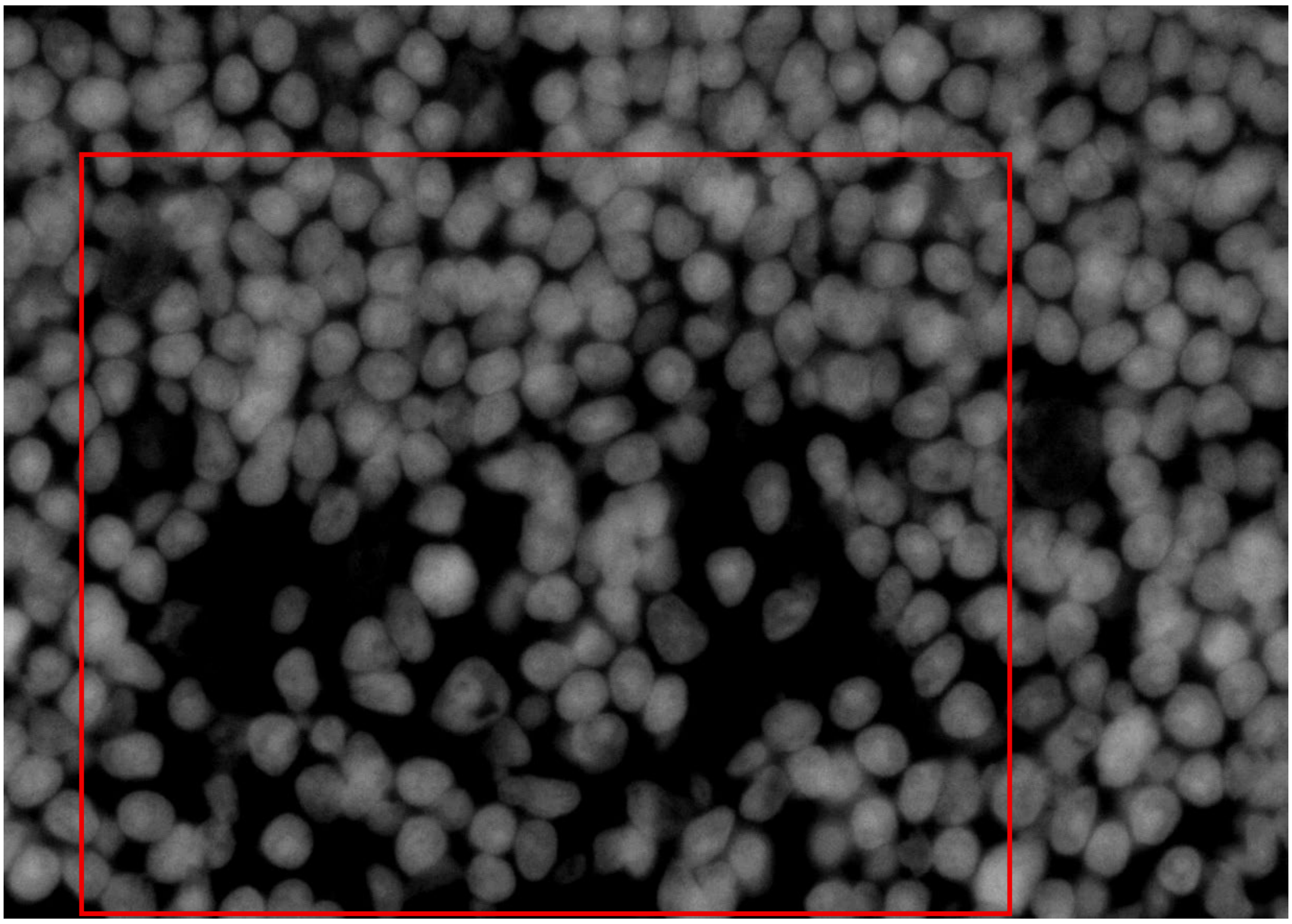

Images used in Figure S9A

KRT1 signal (Alexa Fluor Plus 555)

A left

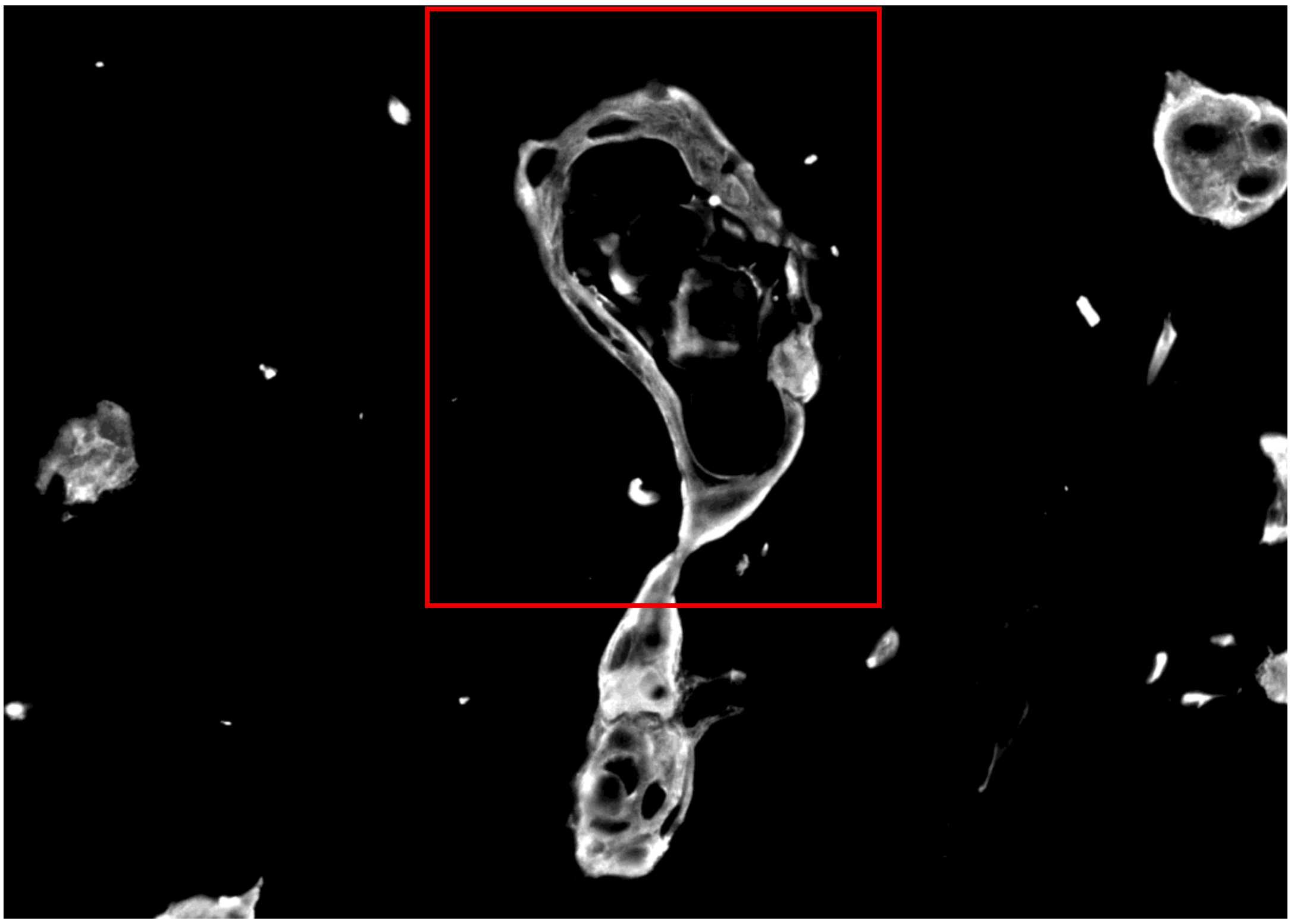

A middle

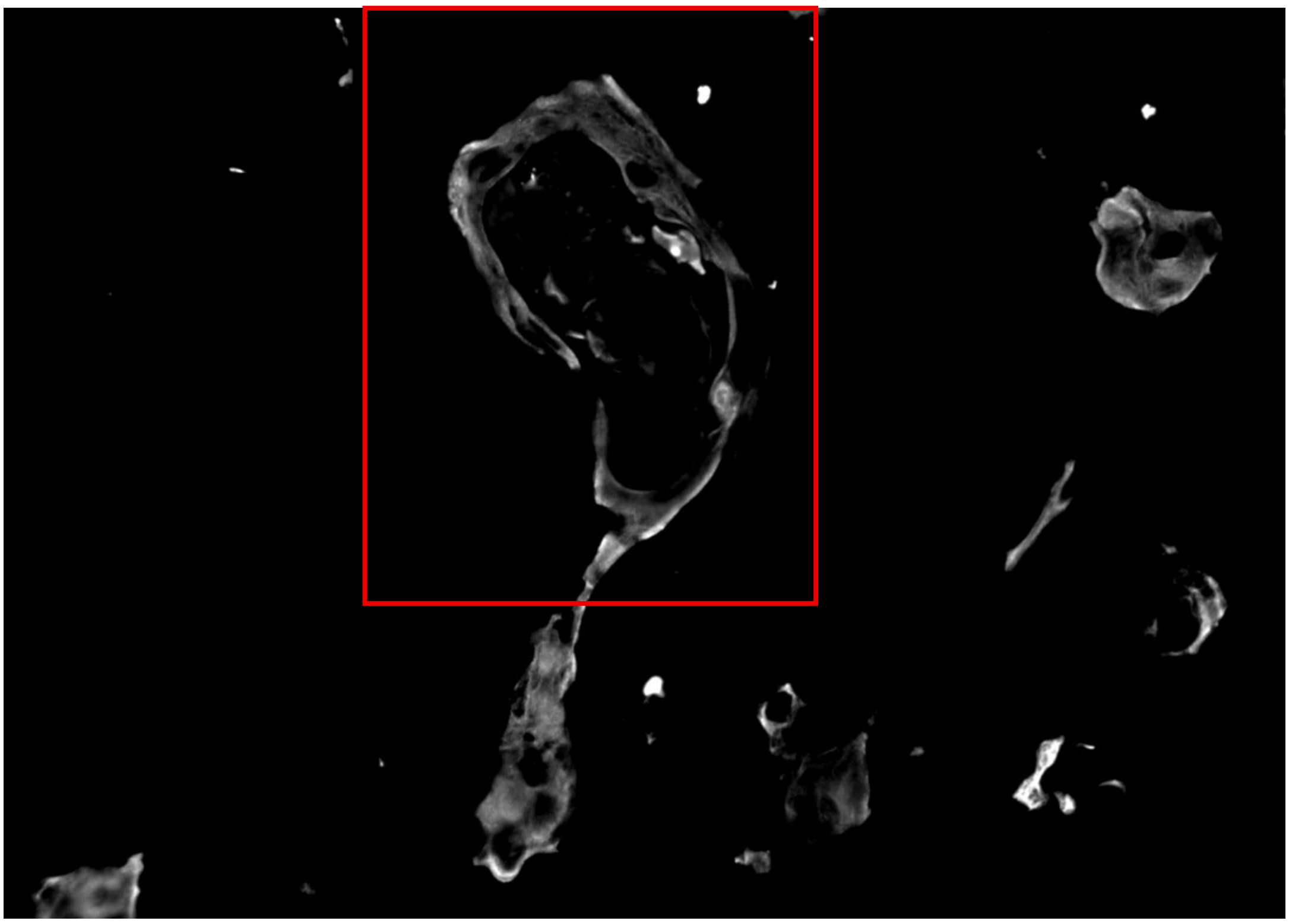

A right KRT1

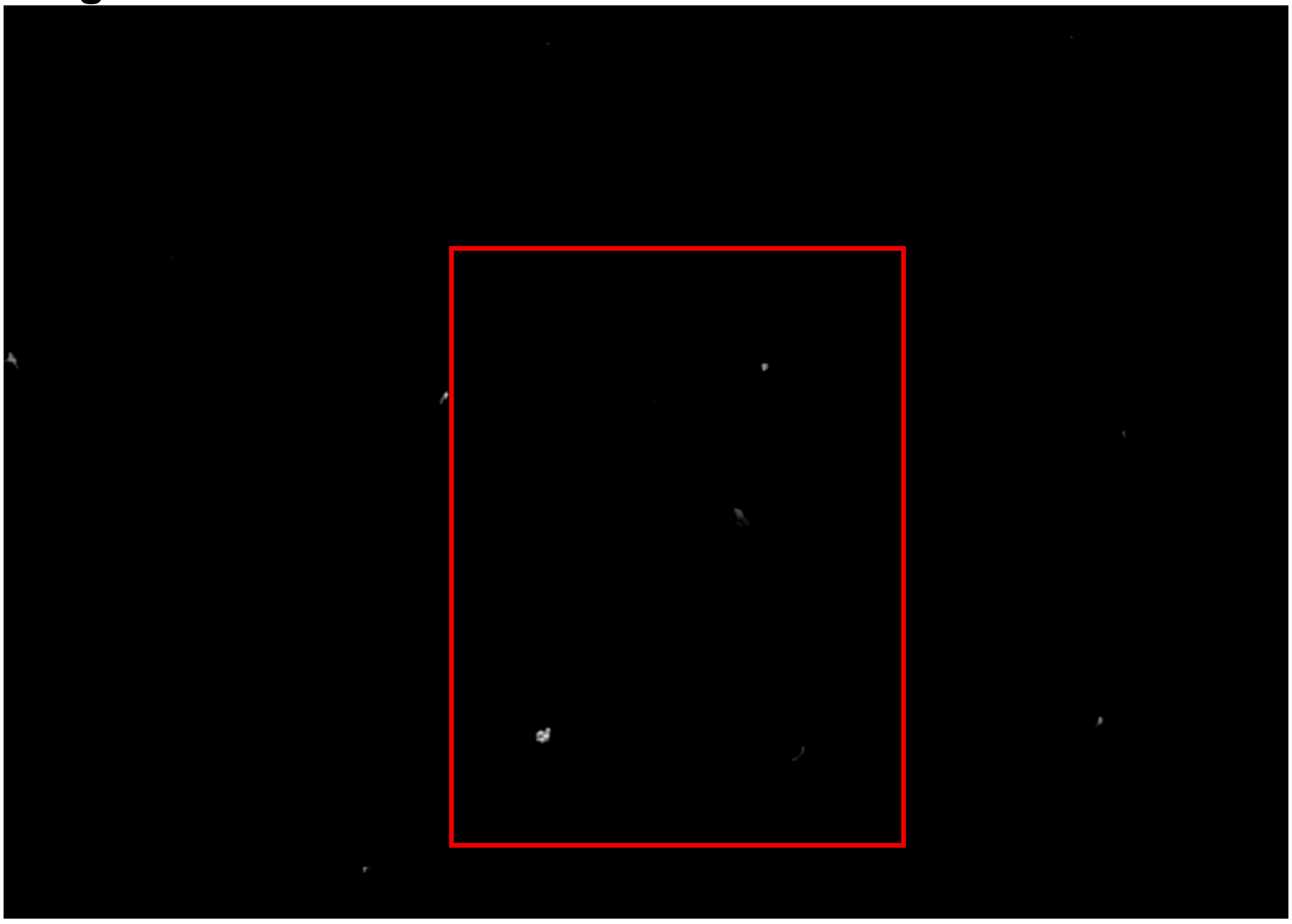

IVL signal (Alexa Fluor Plus 647)

A left IVL

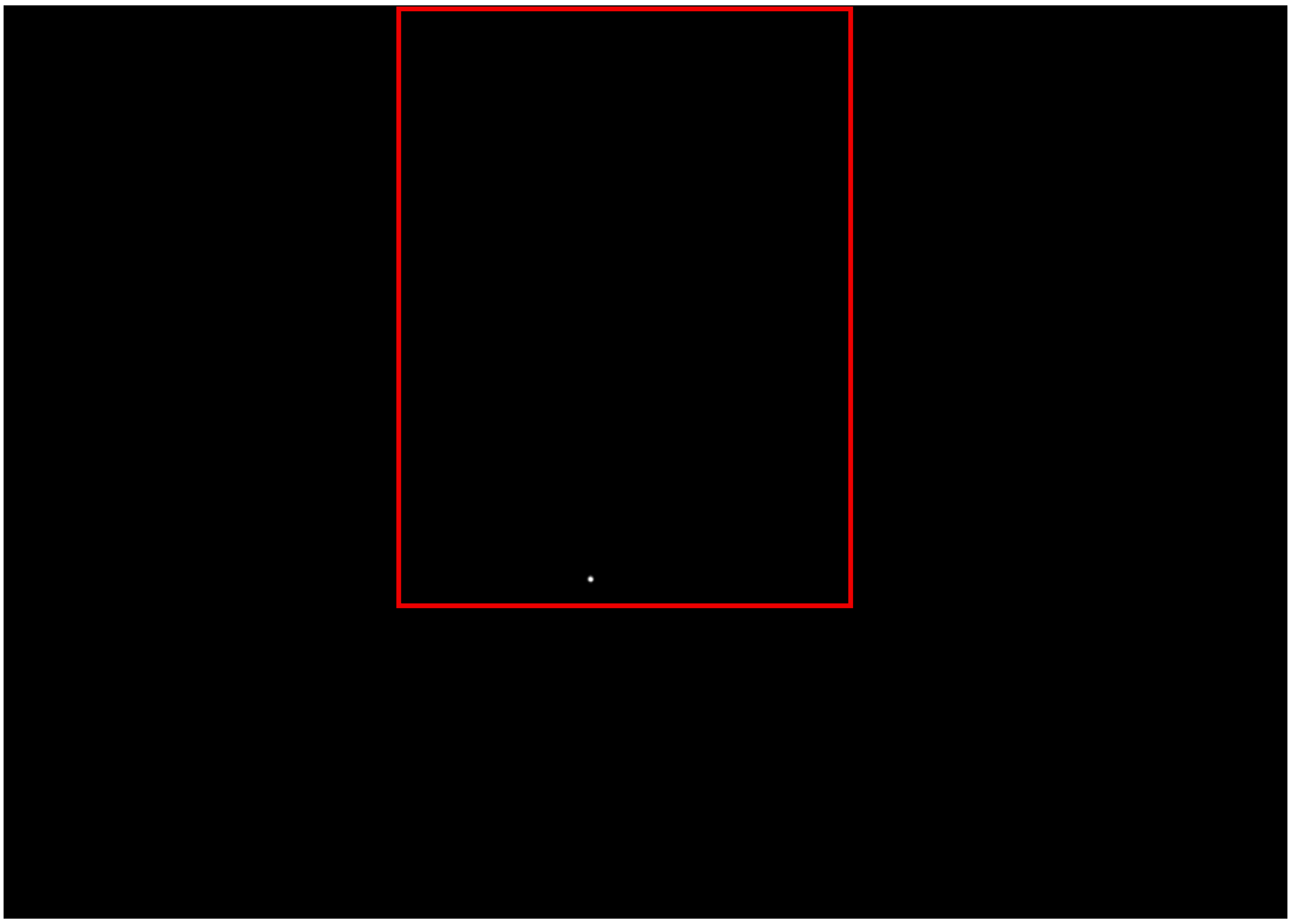

A middle IVL

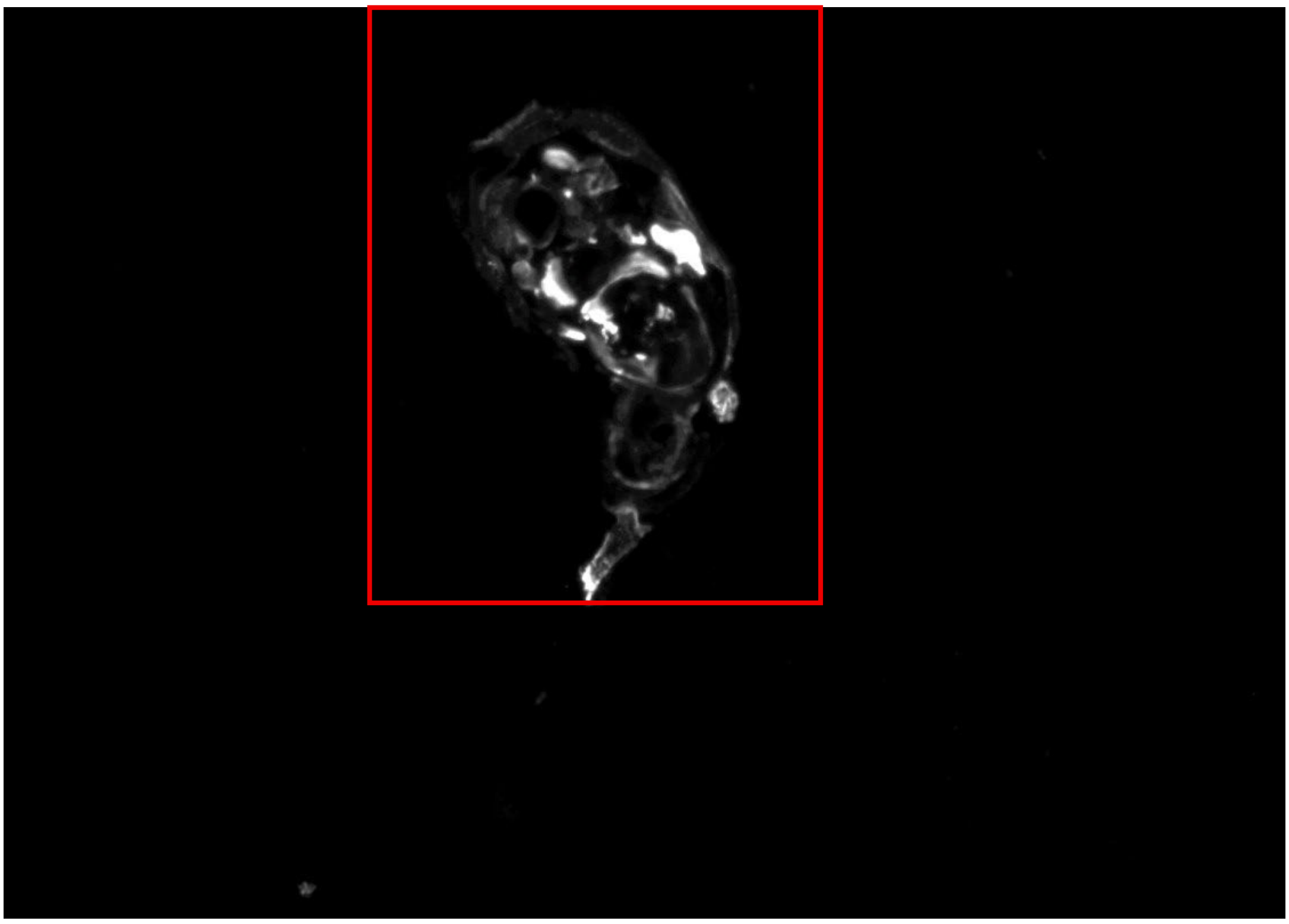

A right IVL

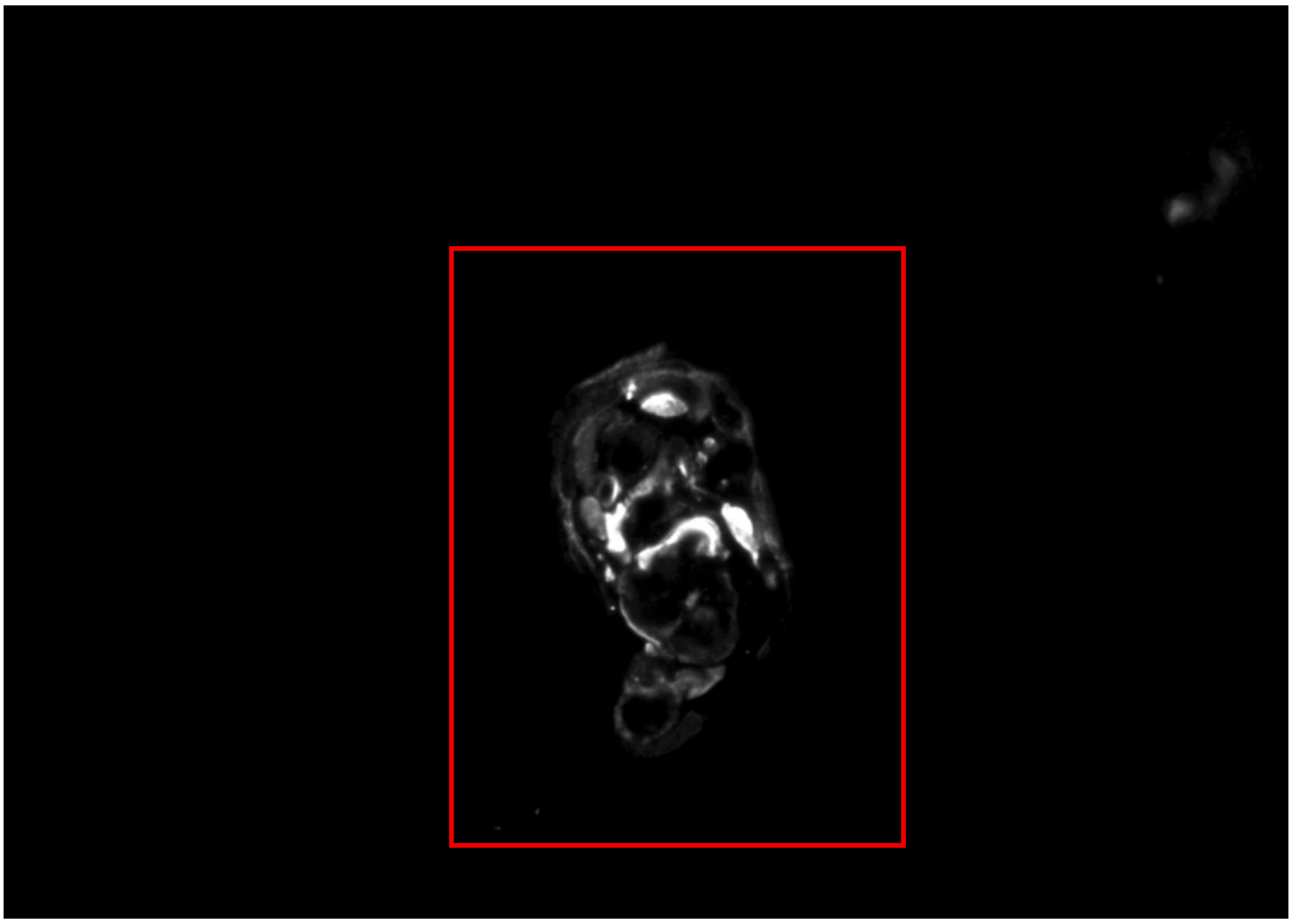

DAPI signal

A left

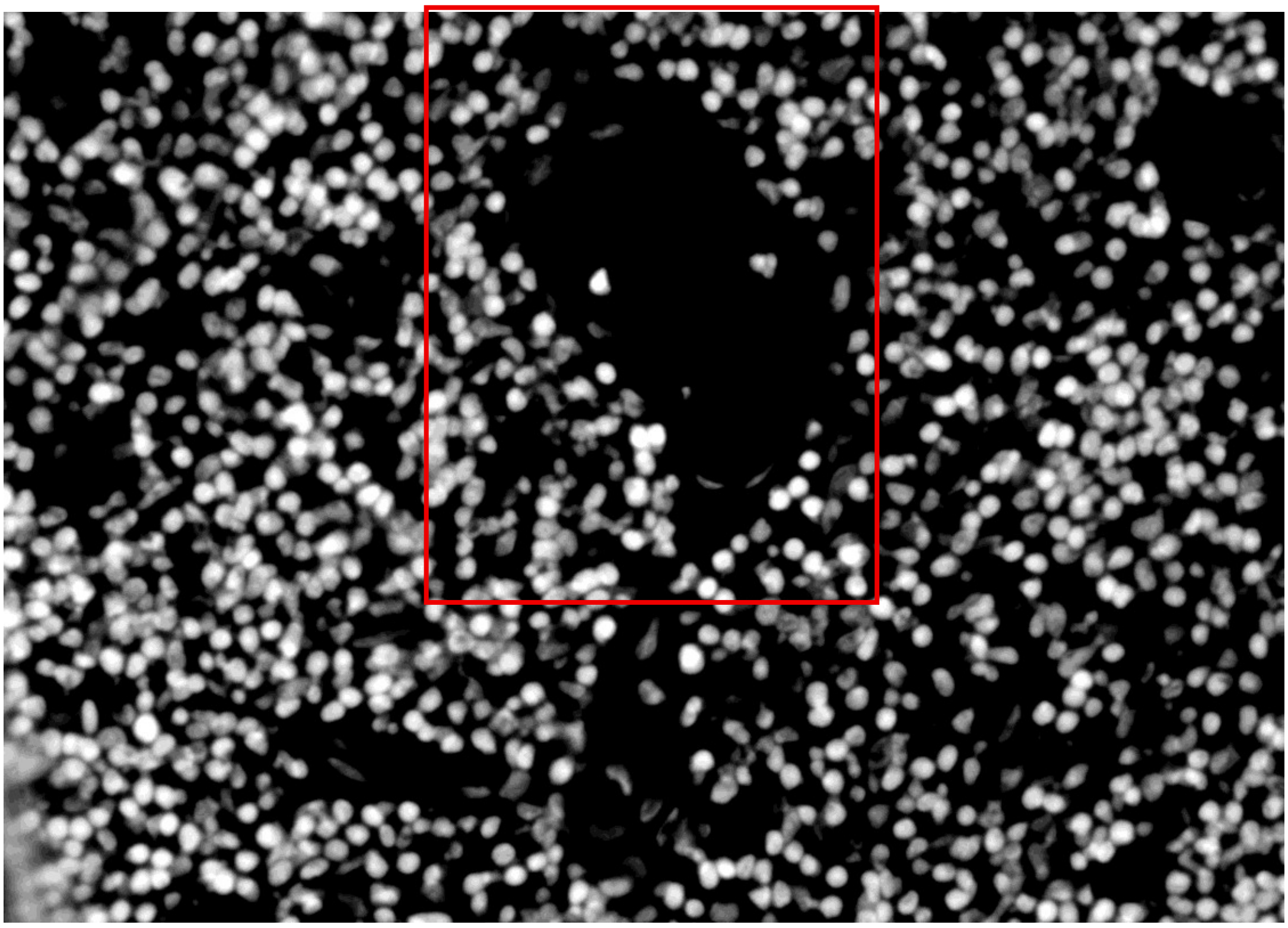

A middle

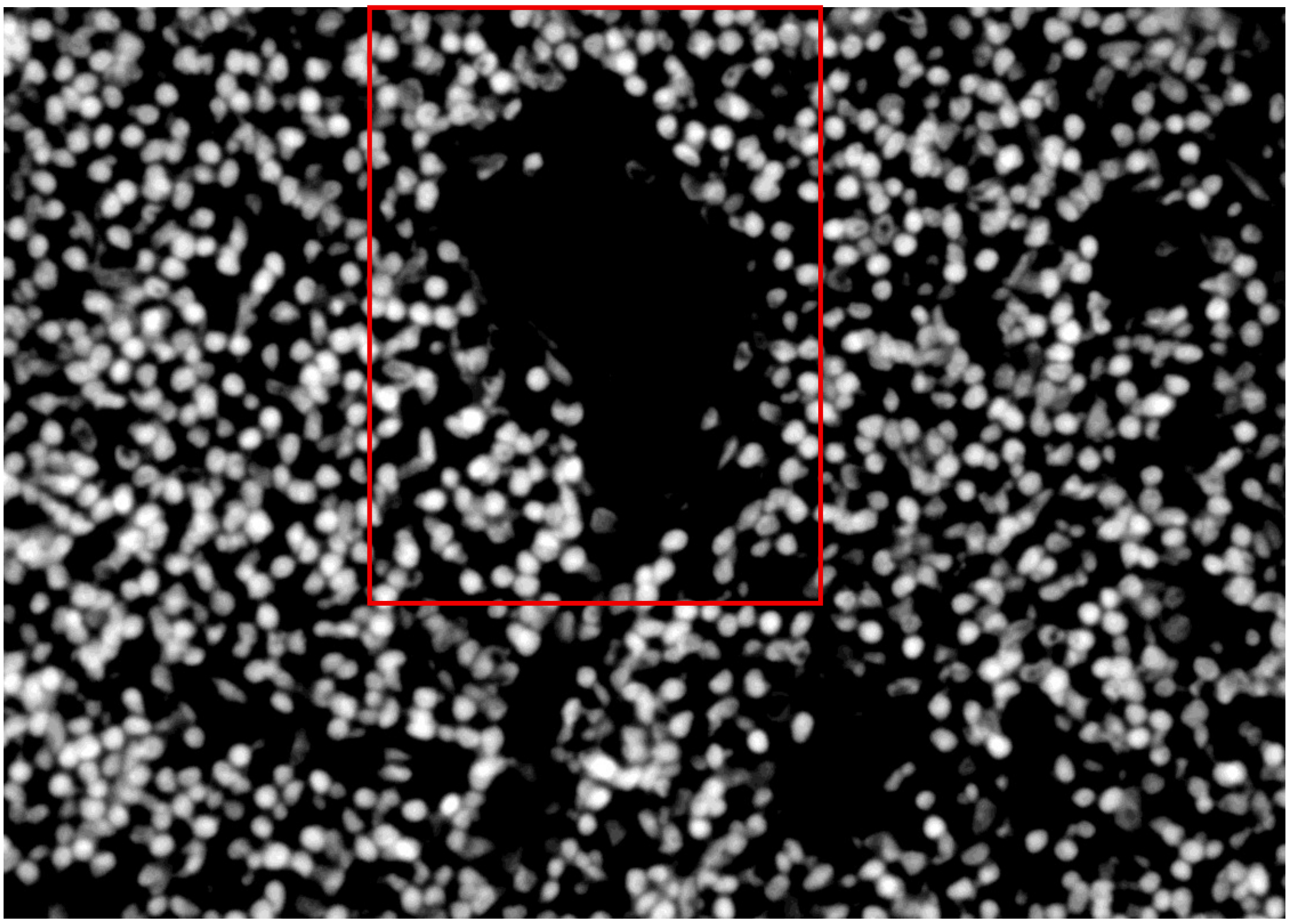

A right

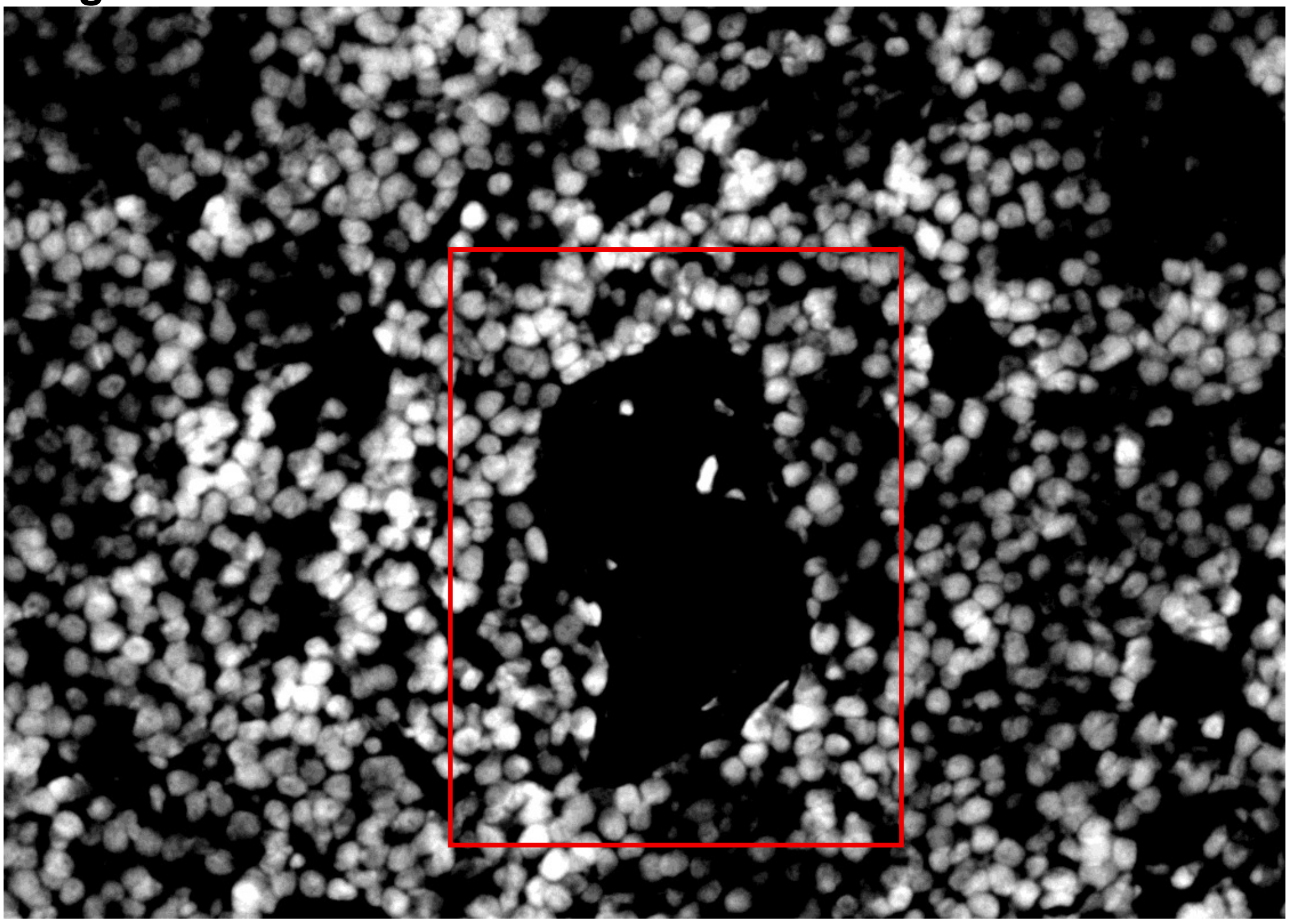

Images used in Figure S9C-S9H

NEURL2 signal (Alexa Fluor Plus 555)

S9C left NEURL2

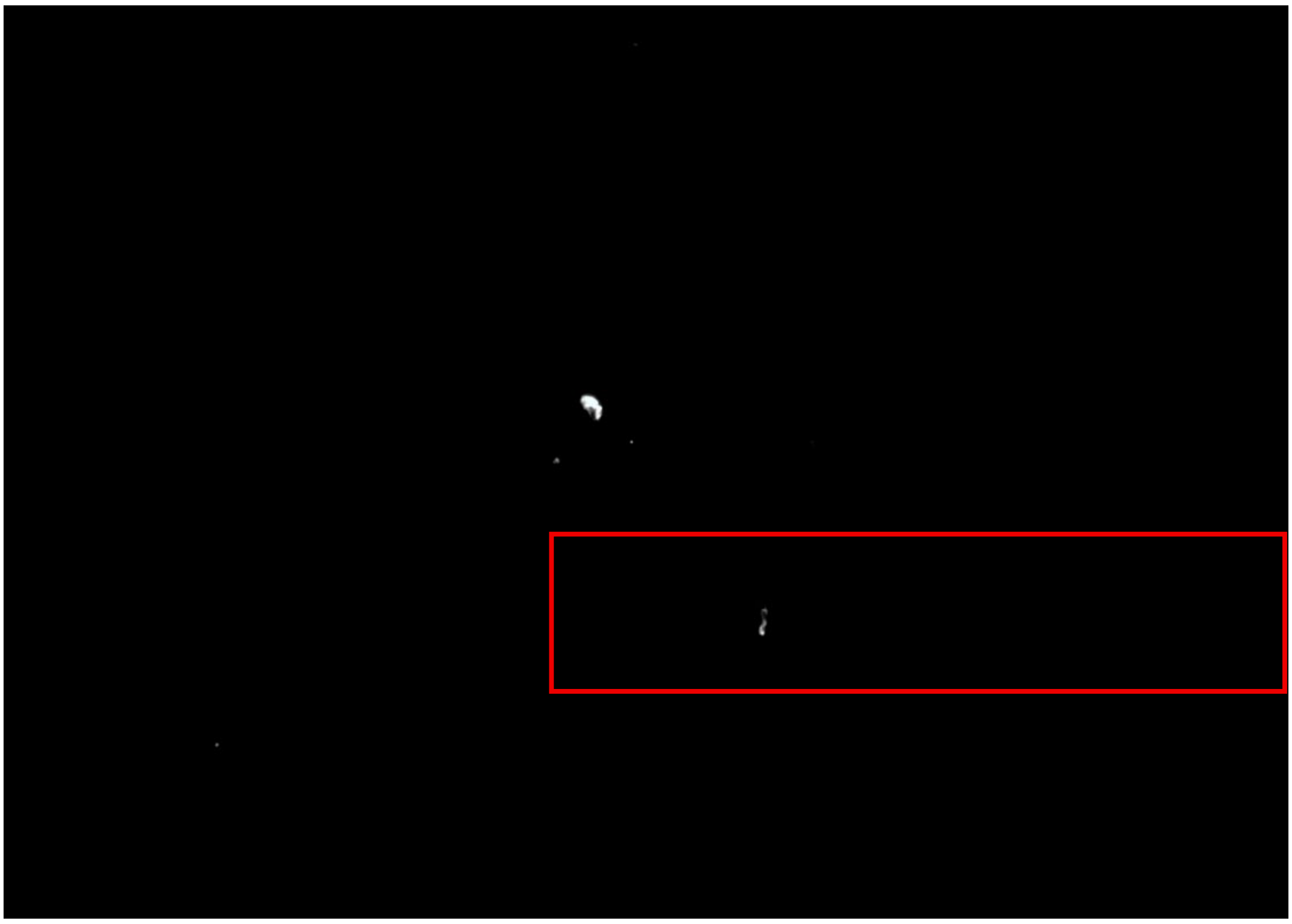

S9C middle NEURL2

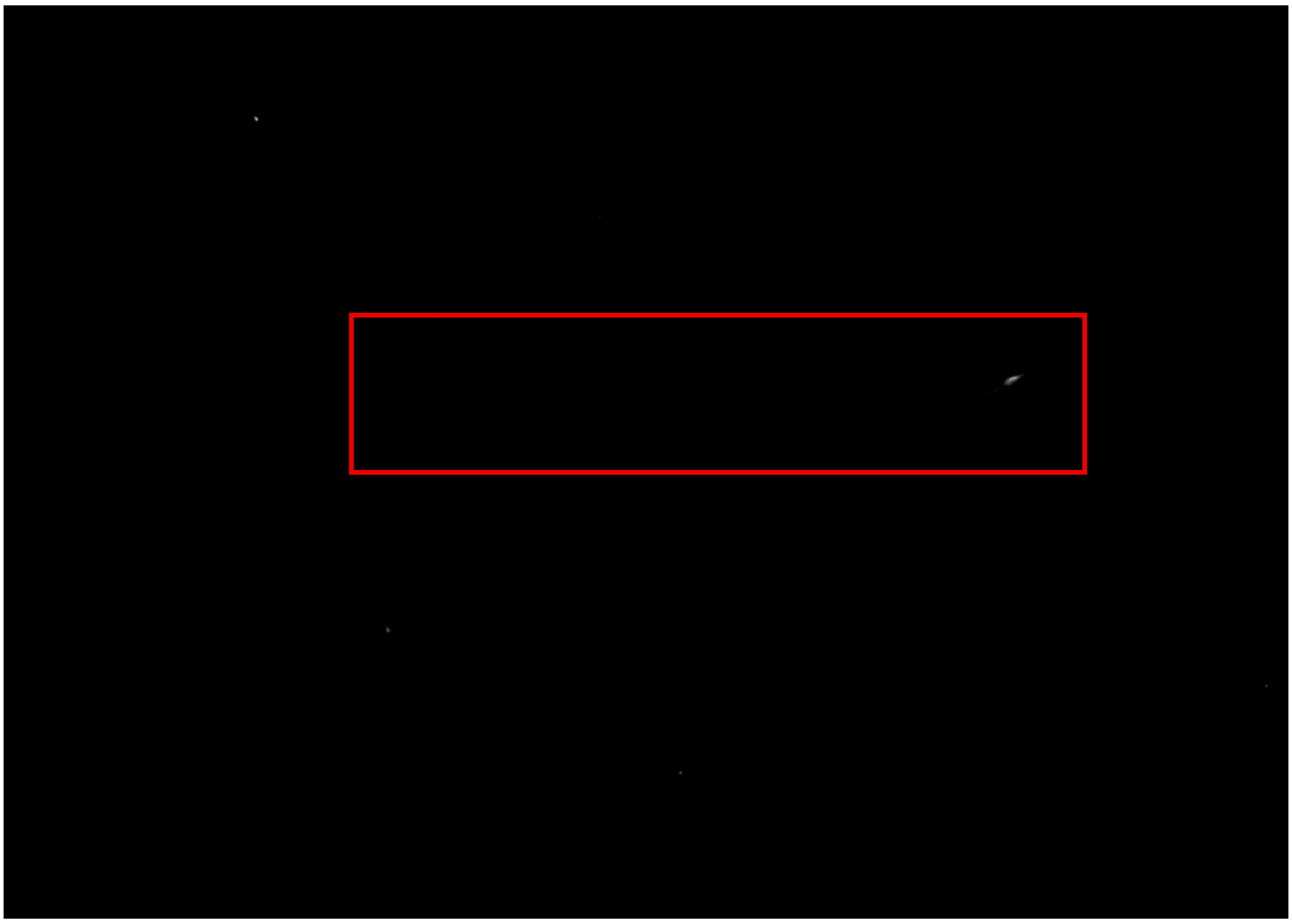

S9C right NEURL2

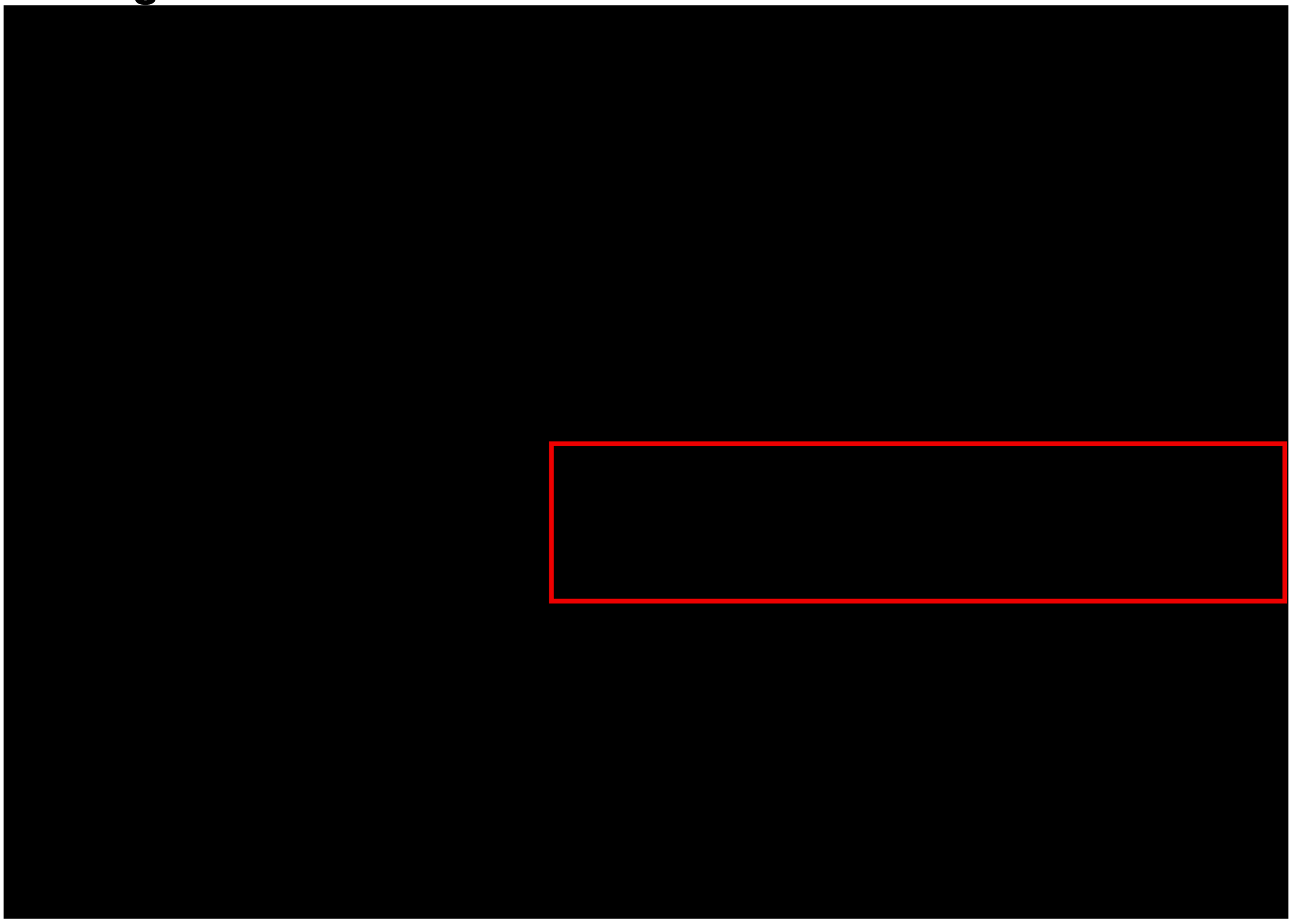

S9 D NEURL2

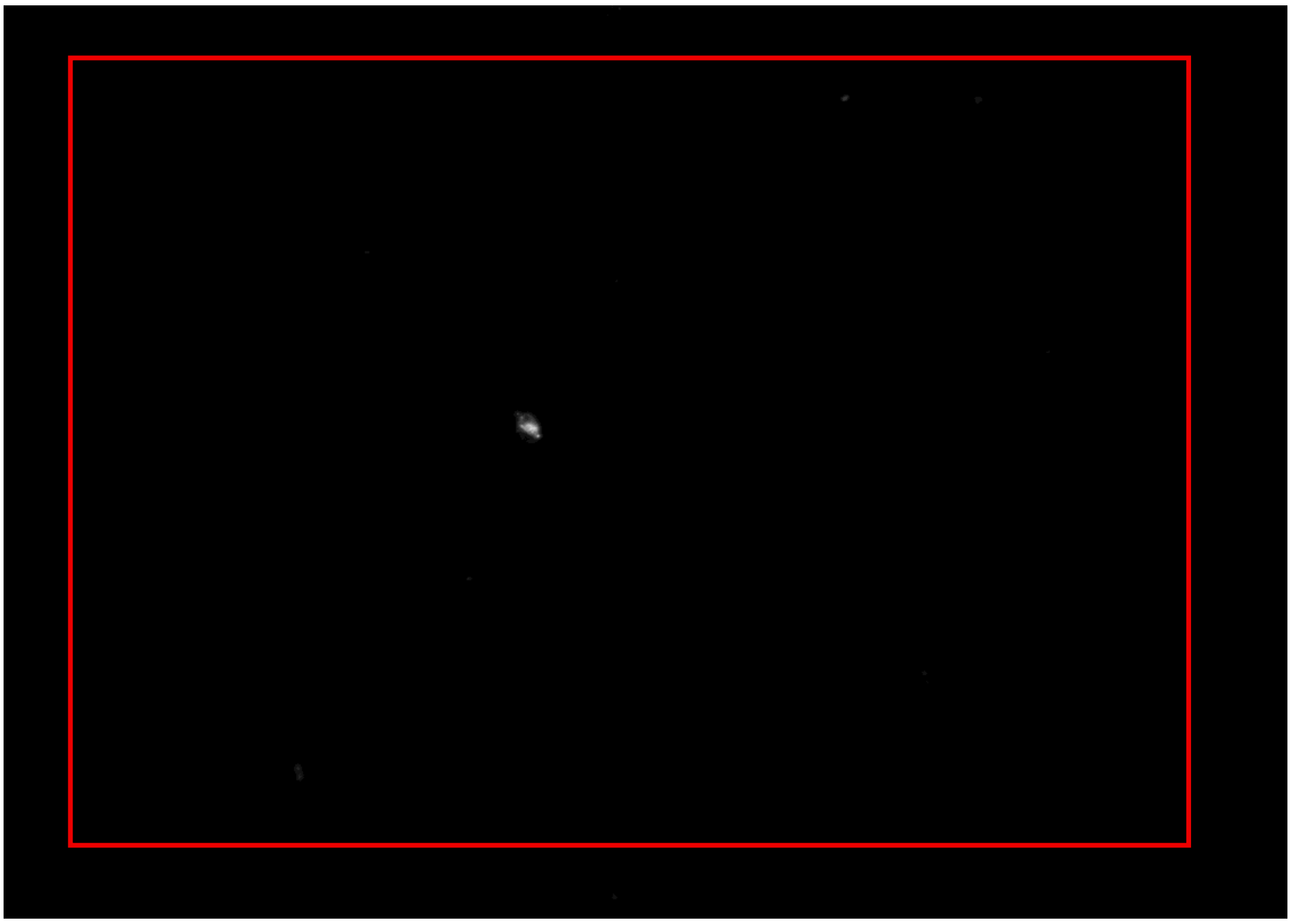

S9E upper NEURL2

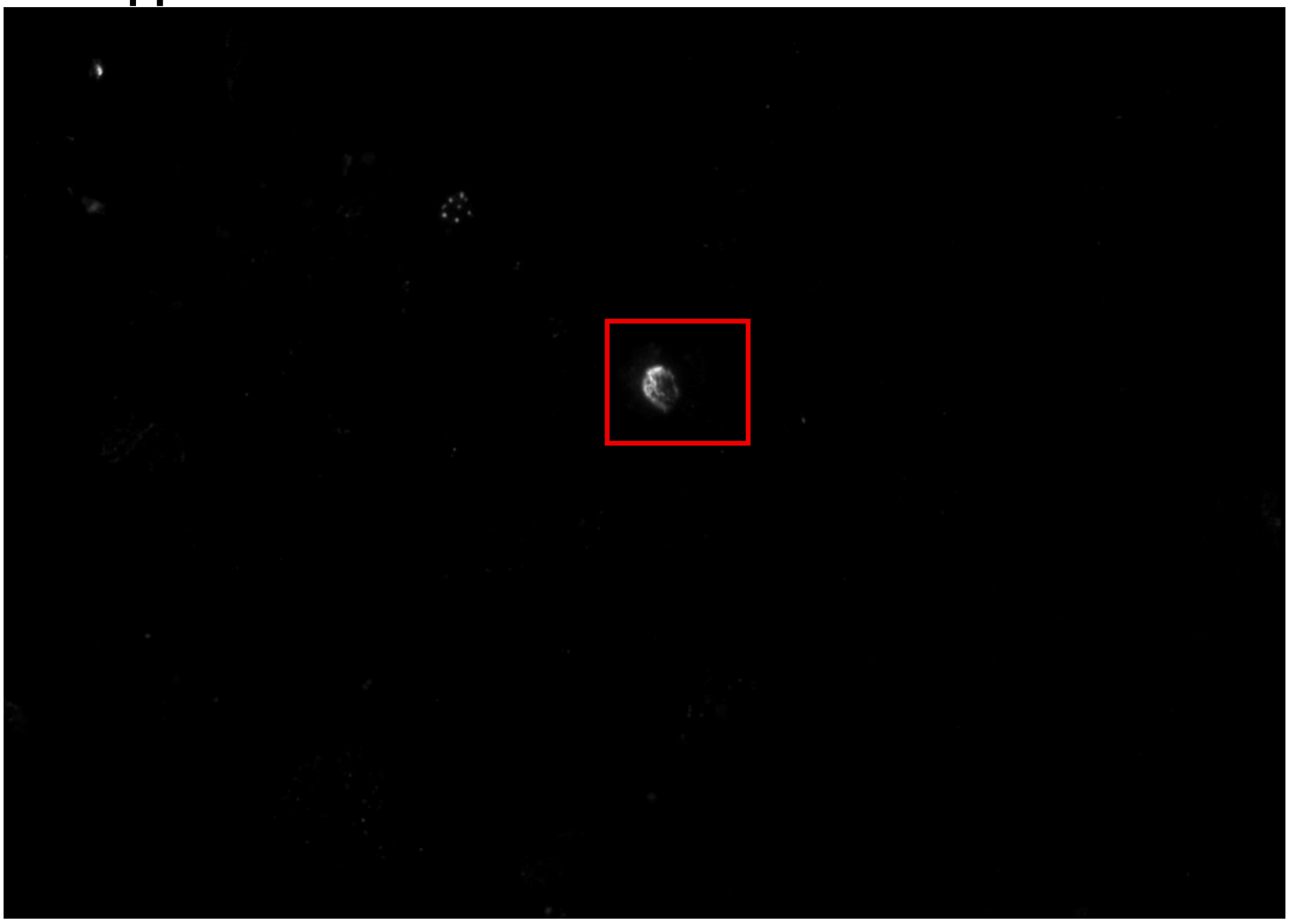

S9E lower NEURL2

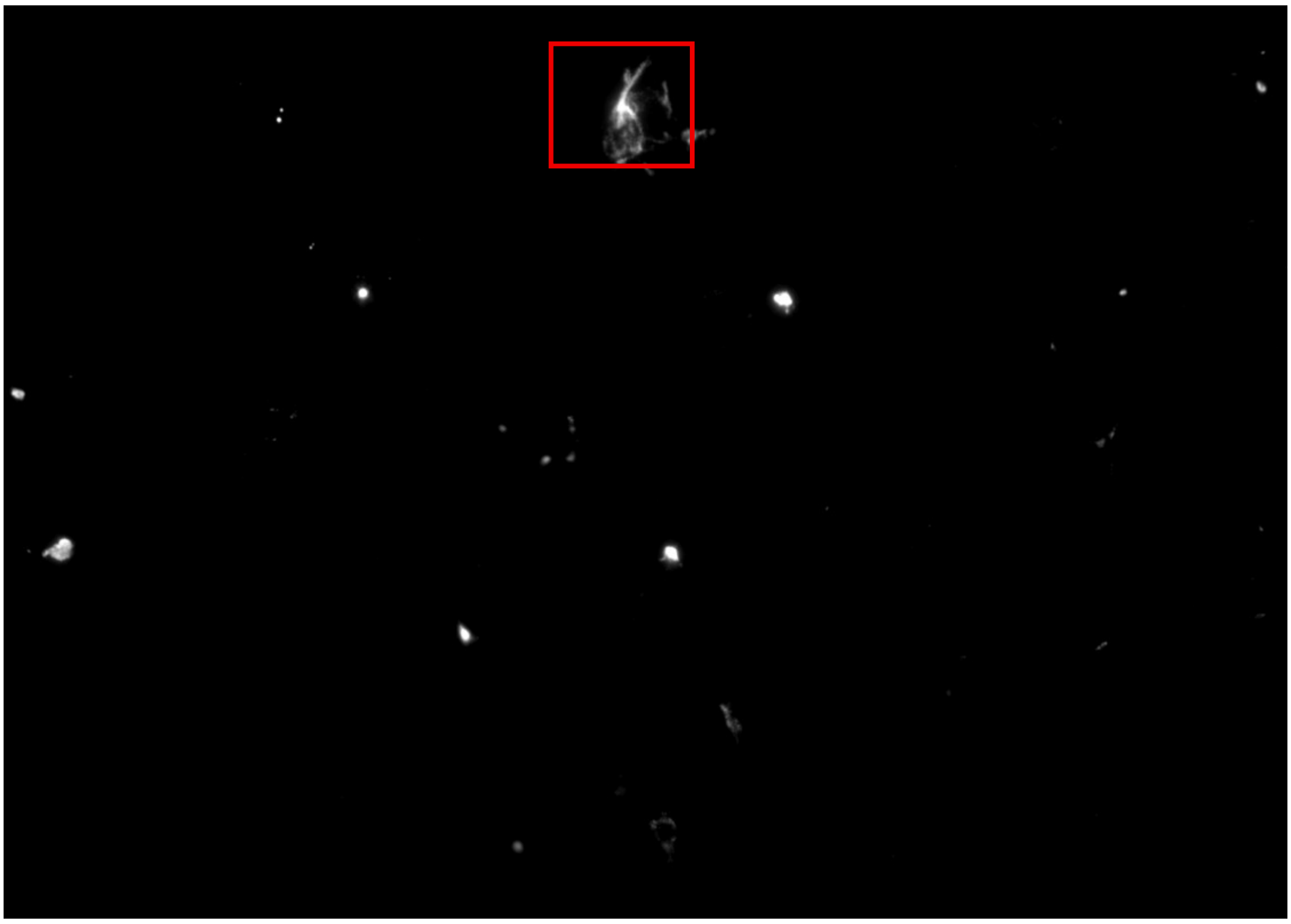

S9F NEURL2

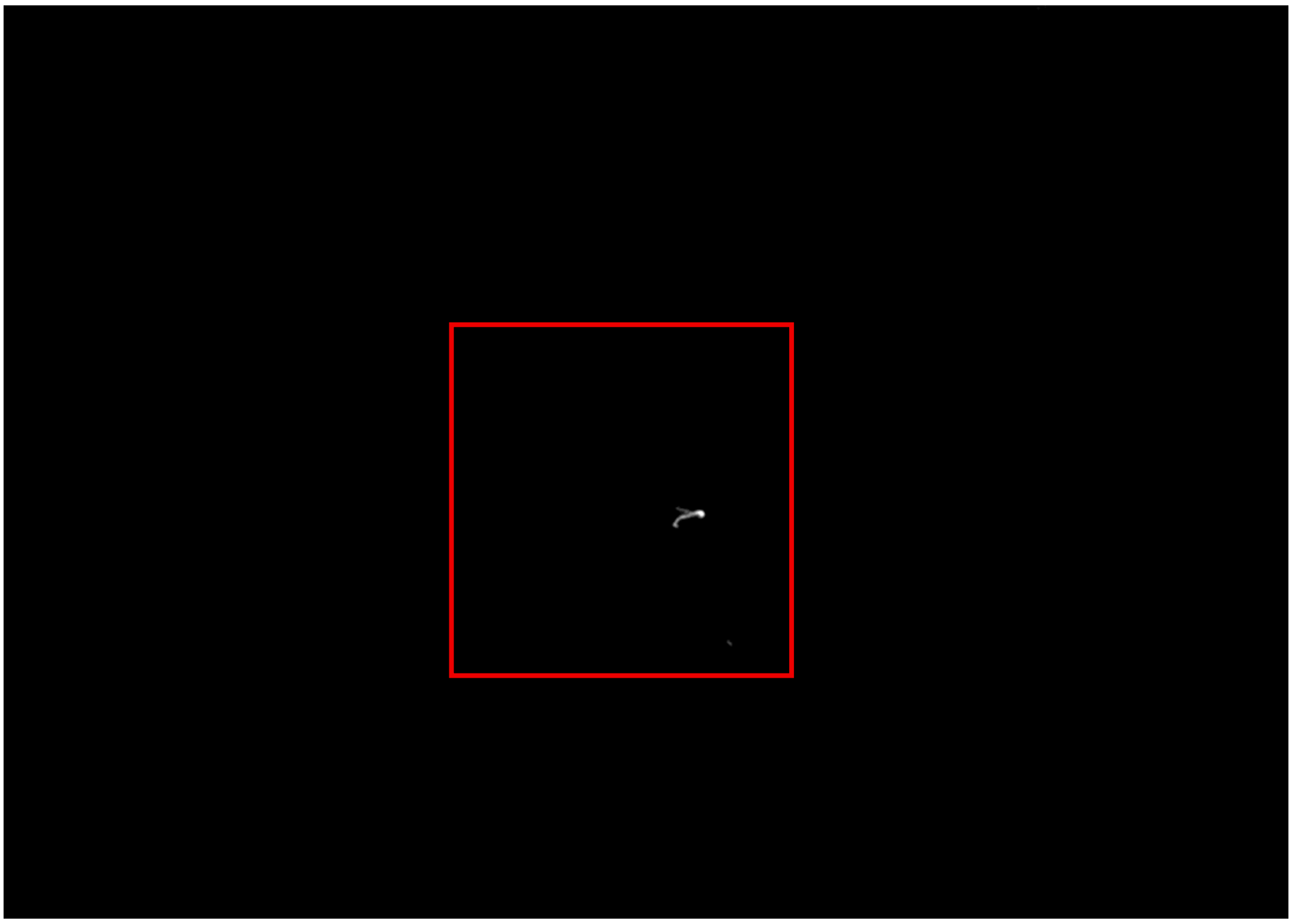

S9G left NEURL2

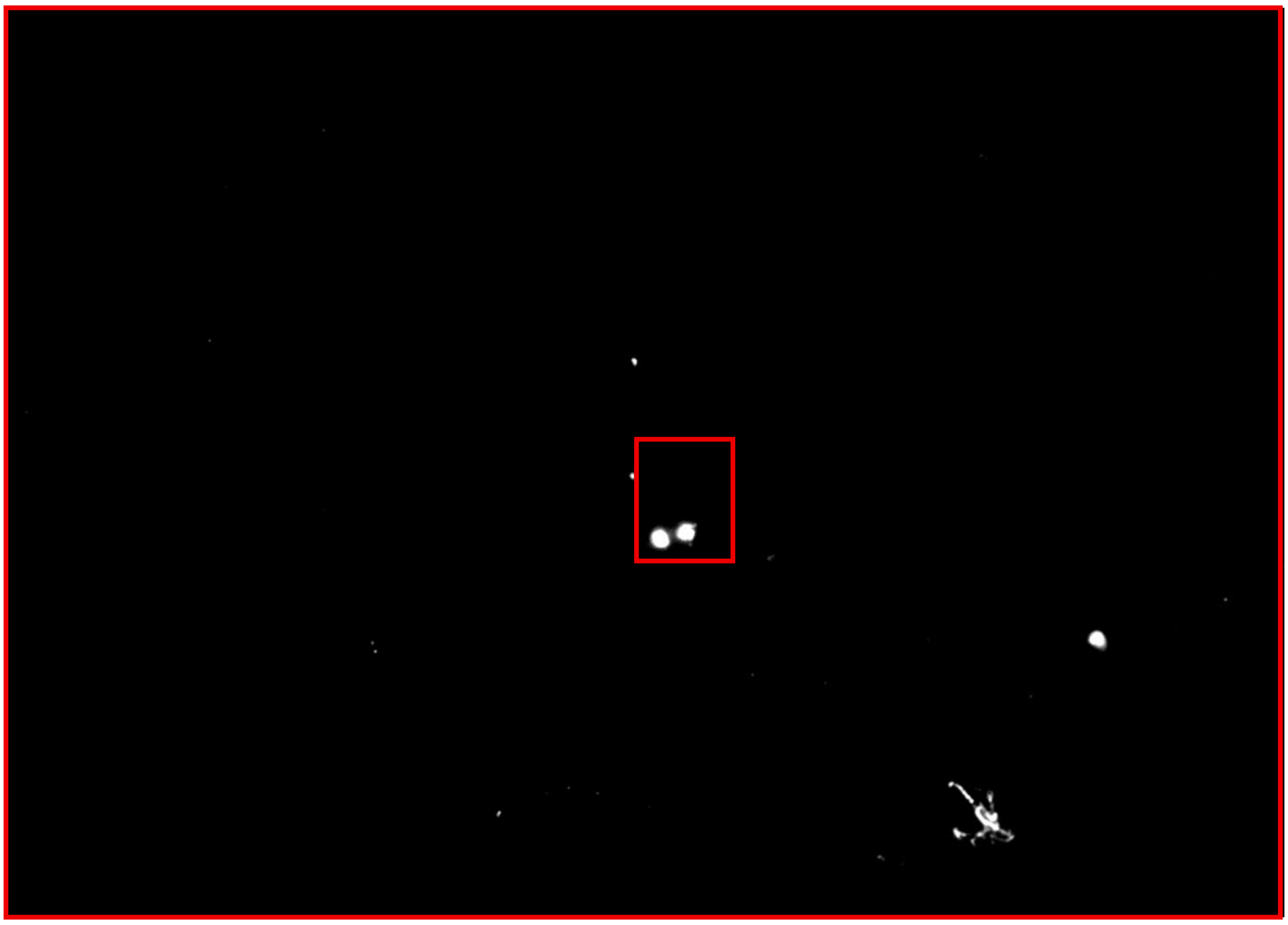

S9G right

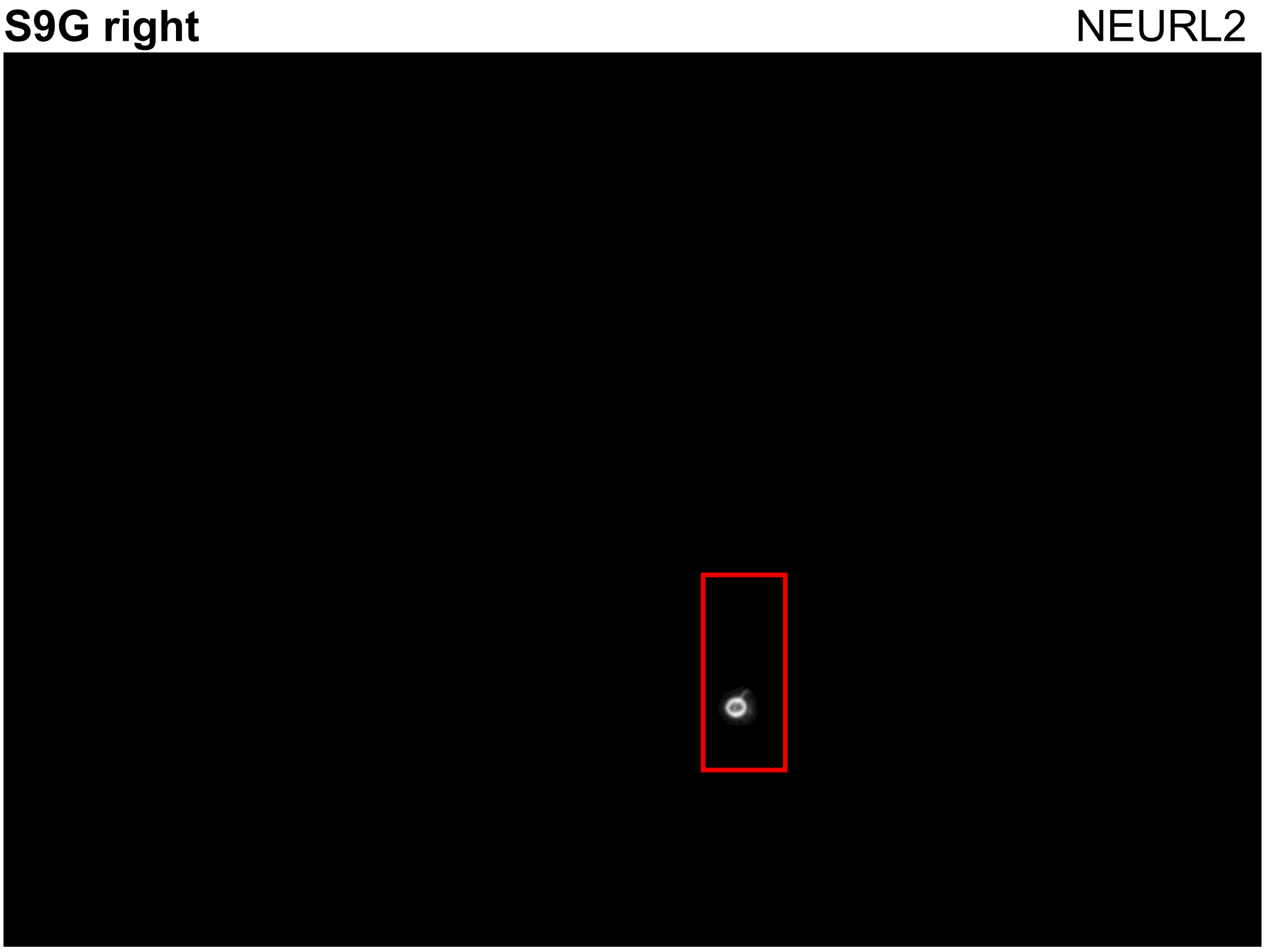

S9H

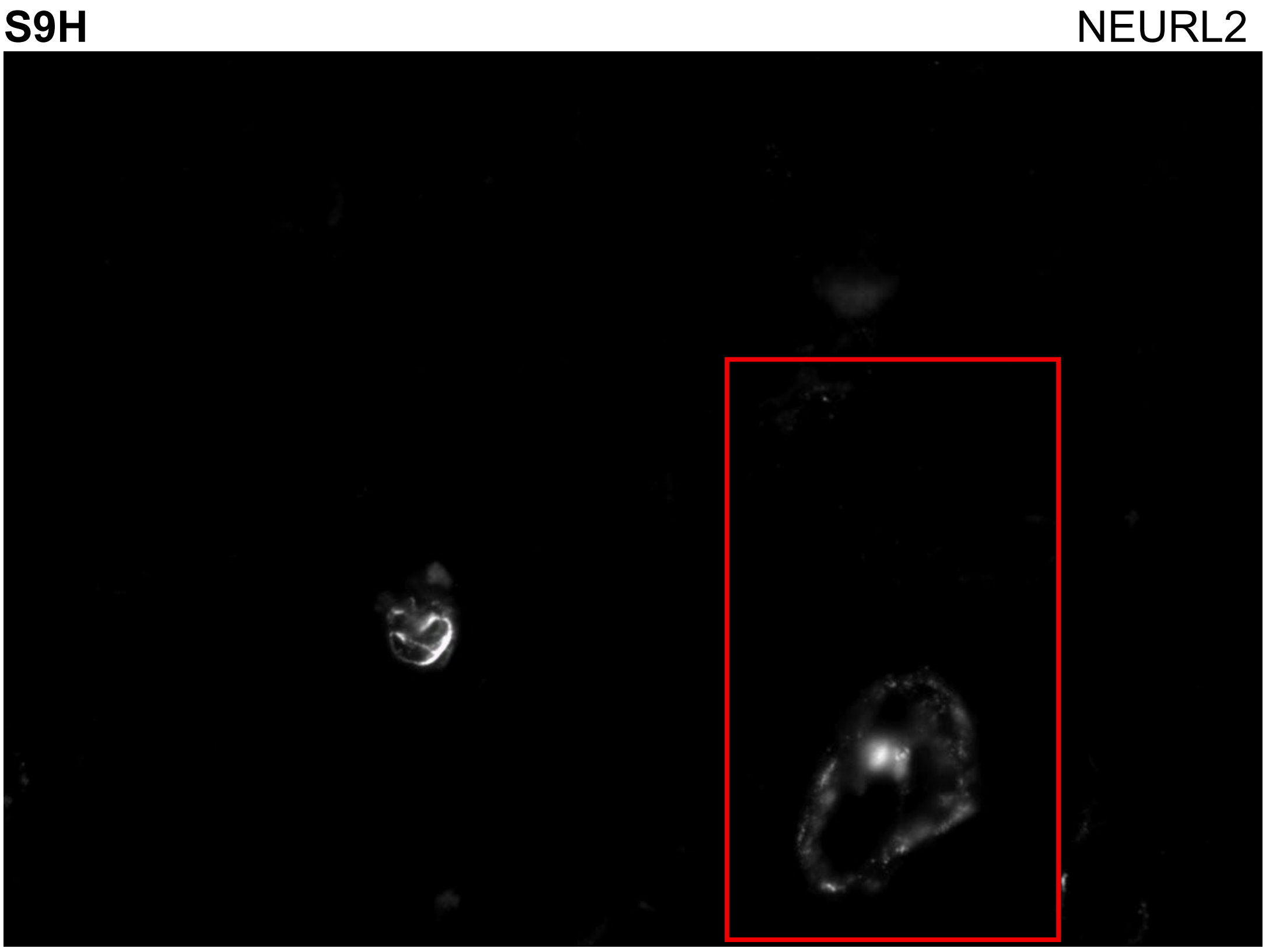

KRT8 signal (Alexa Fluor Plus 647)

S9C left

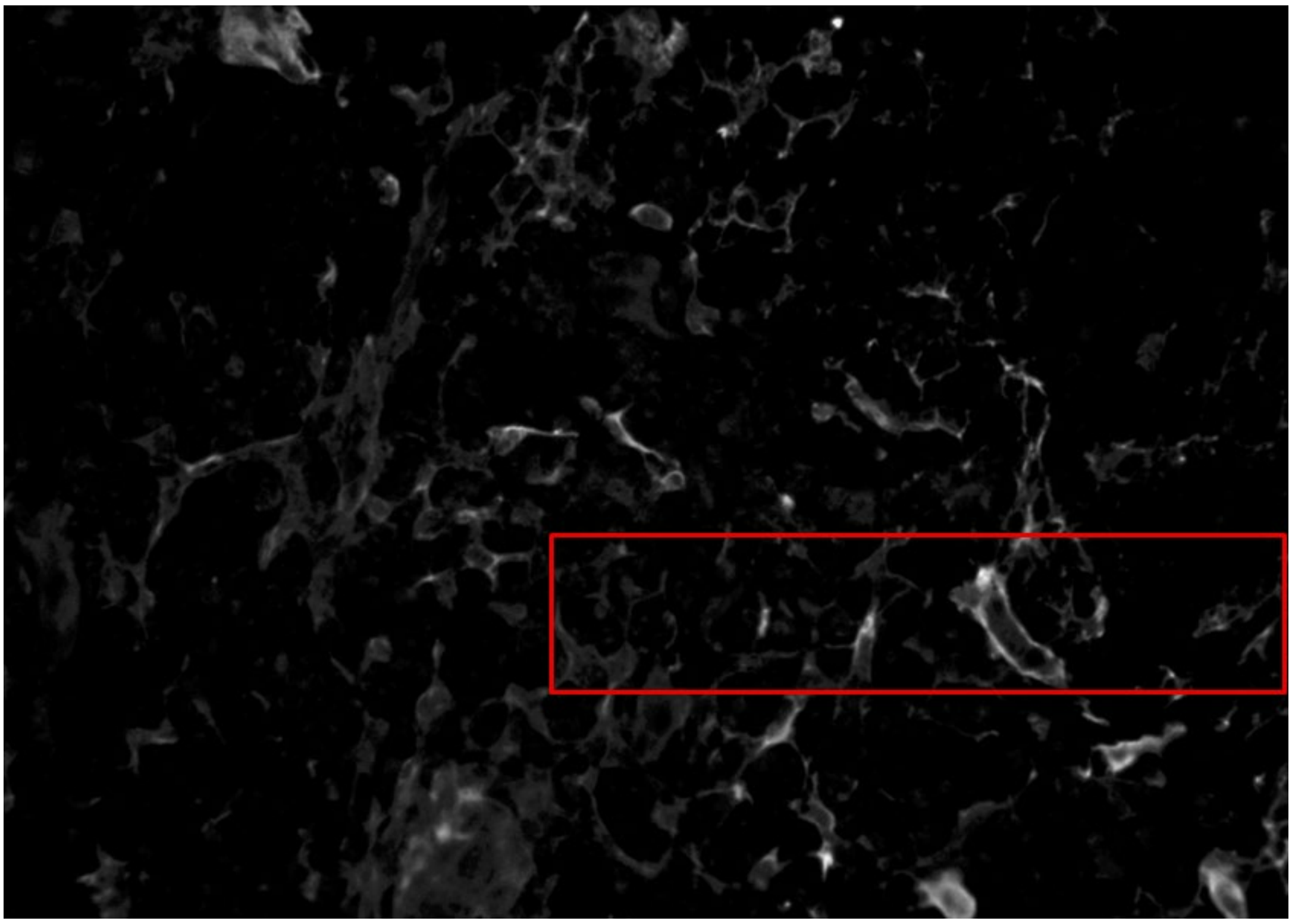

S9C middle

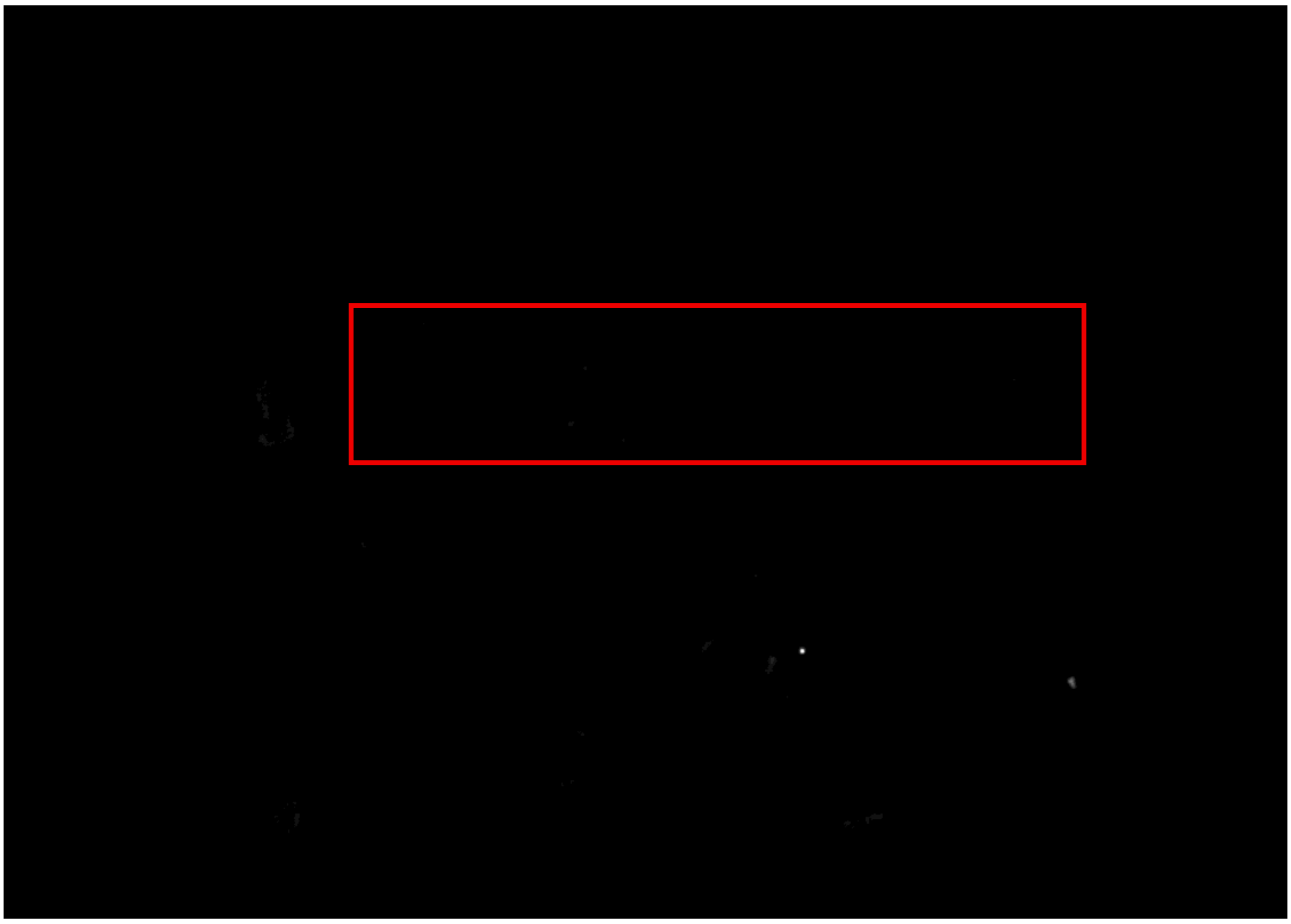

S9C right

KRT8

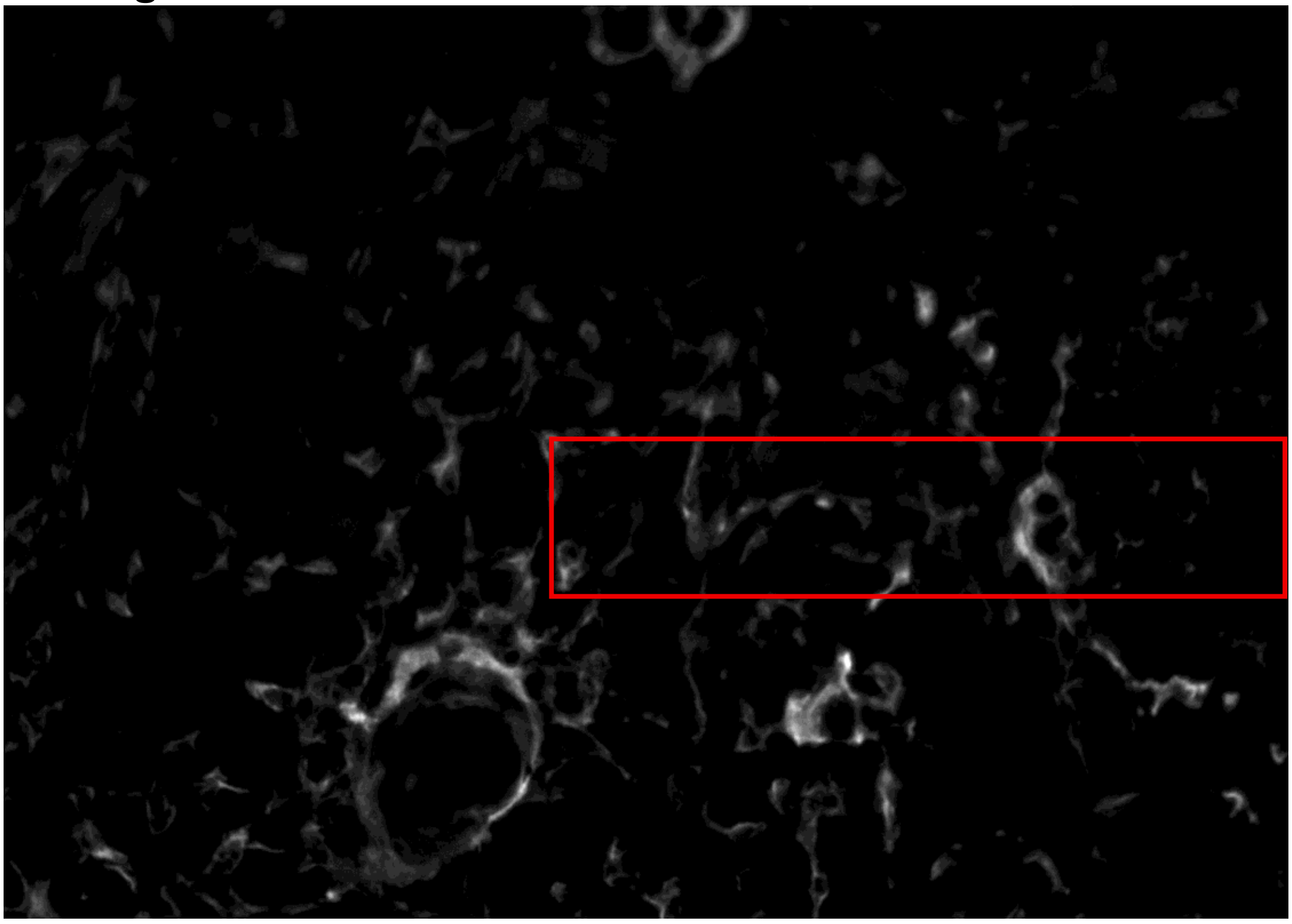

S9 D

KRT8

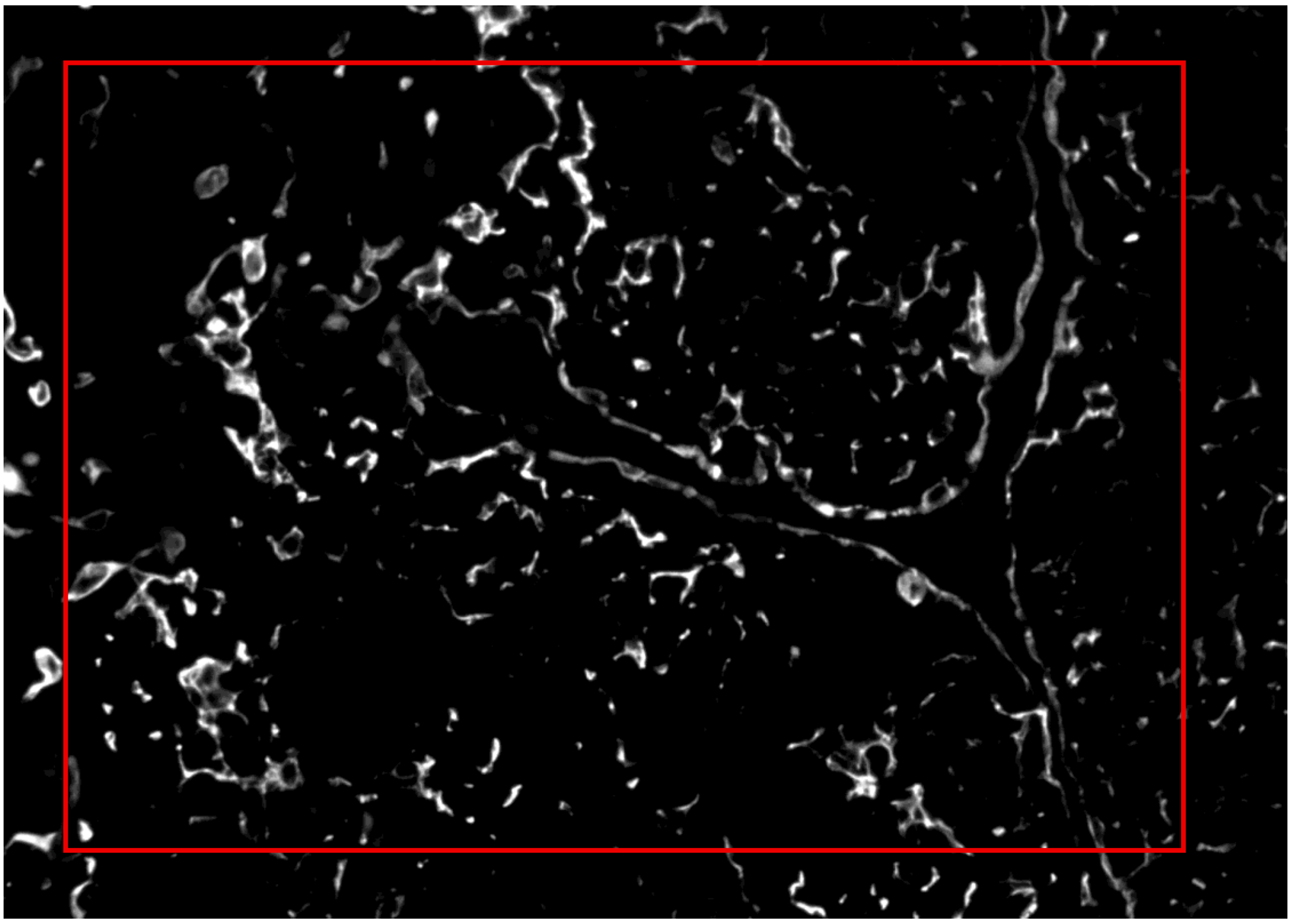

S9F

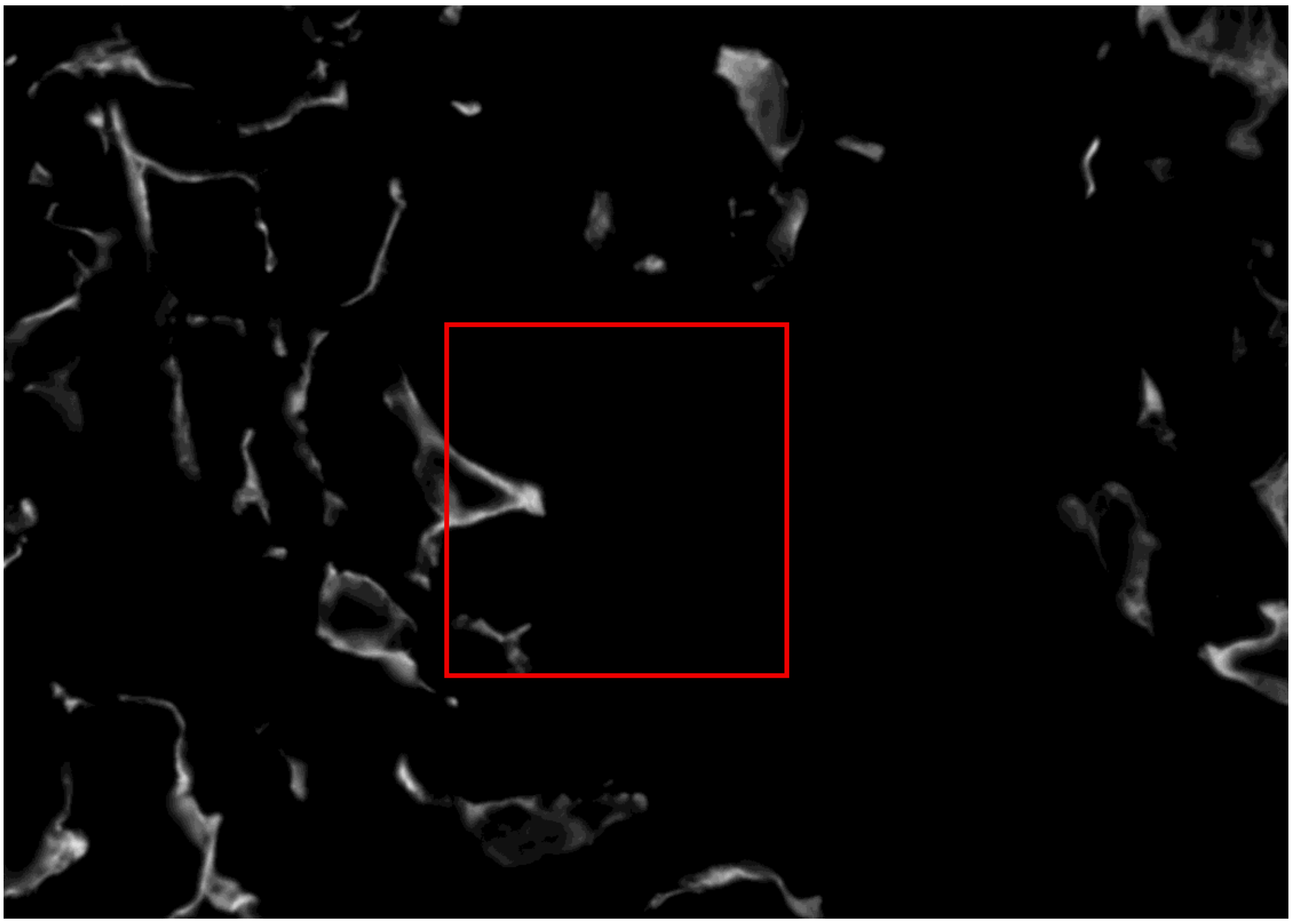

S9G left

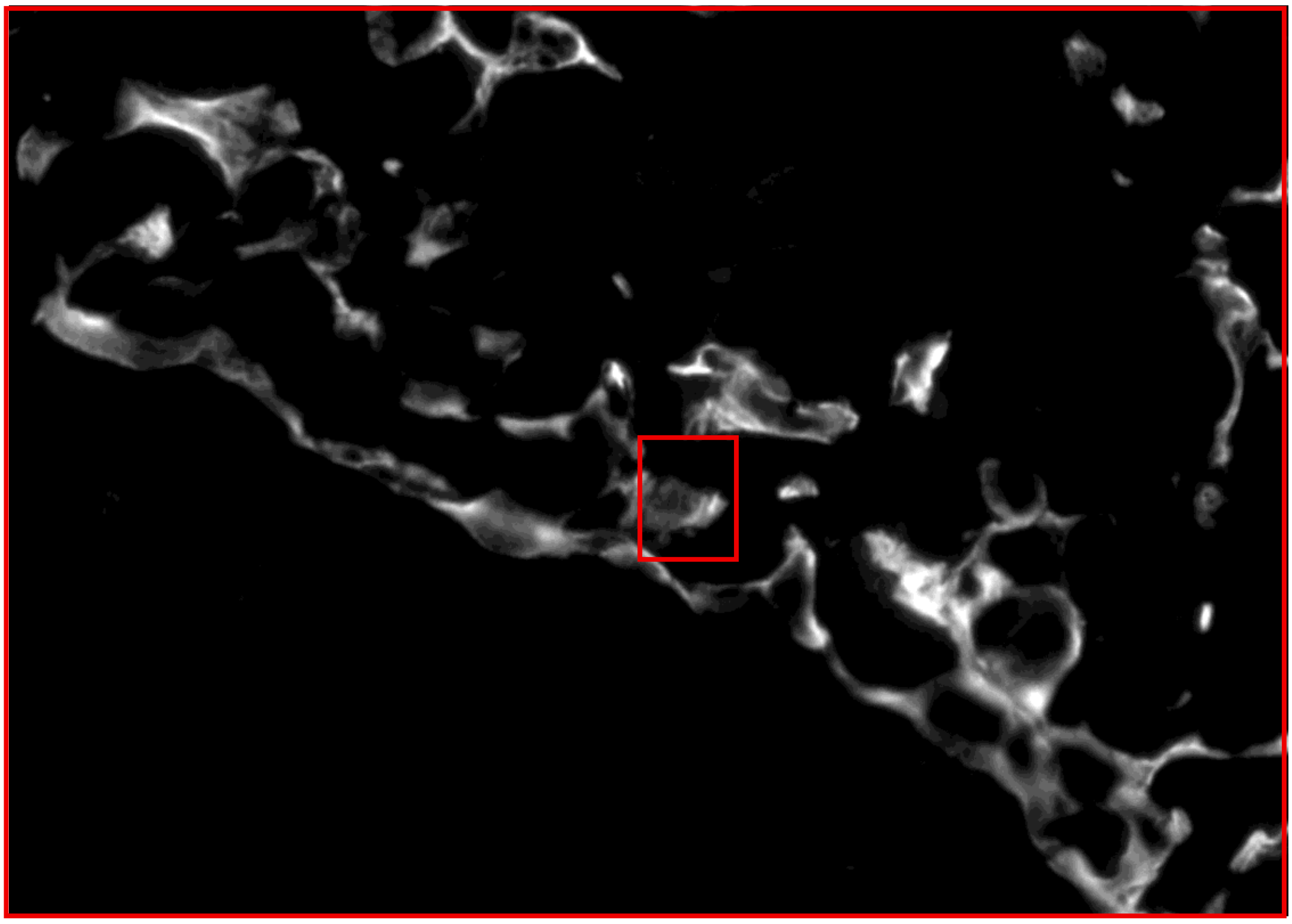

S9H KRT8

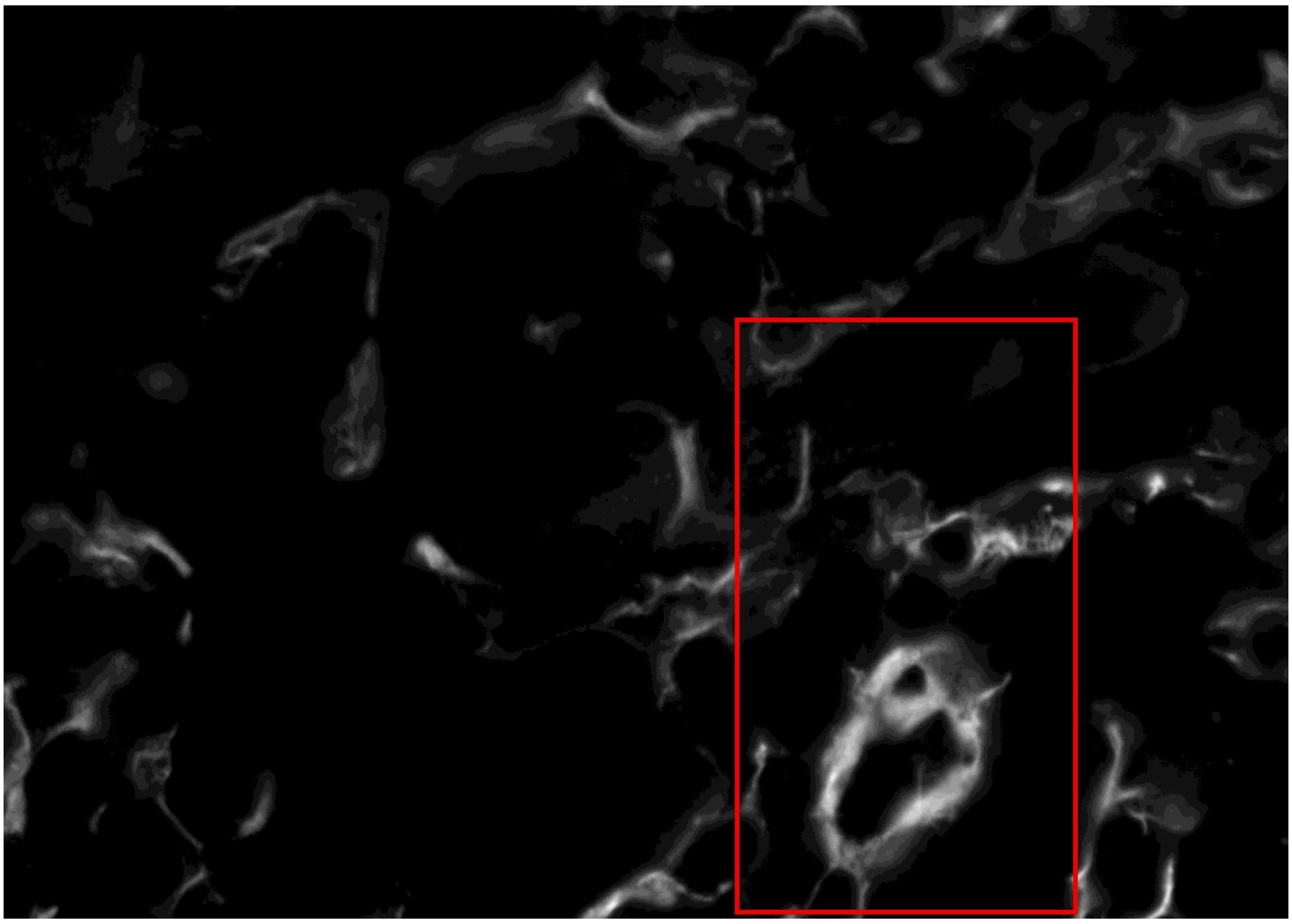

DAPI signal

S9C left DAPI

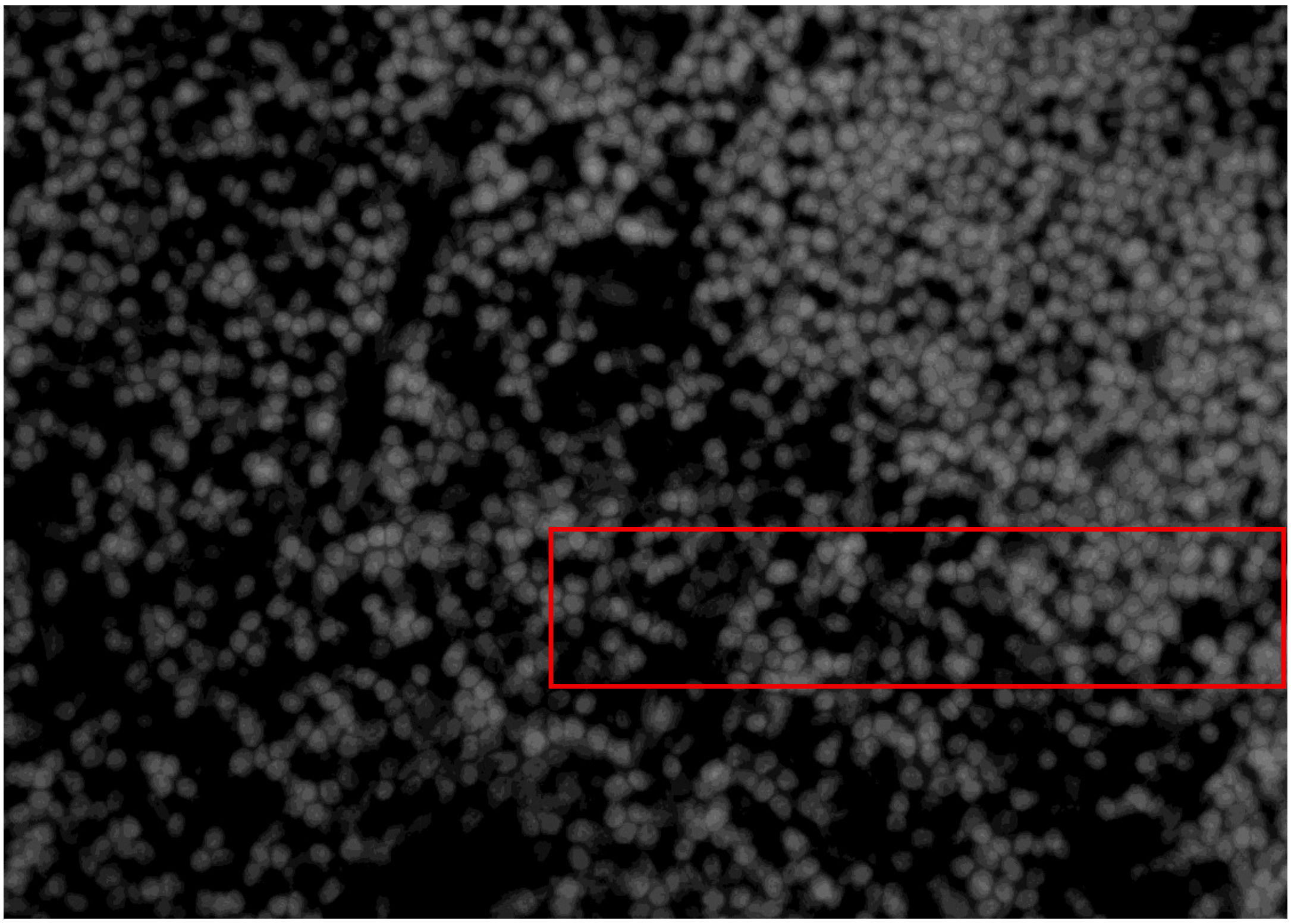

S9C middle DAPI

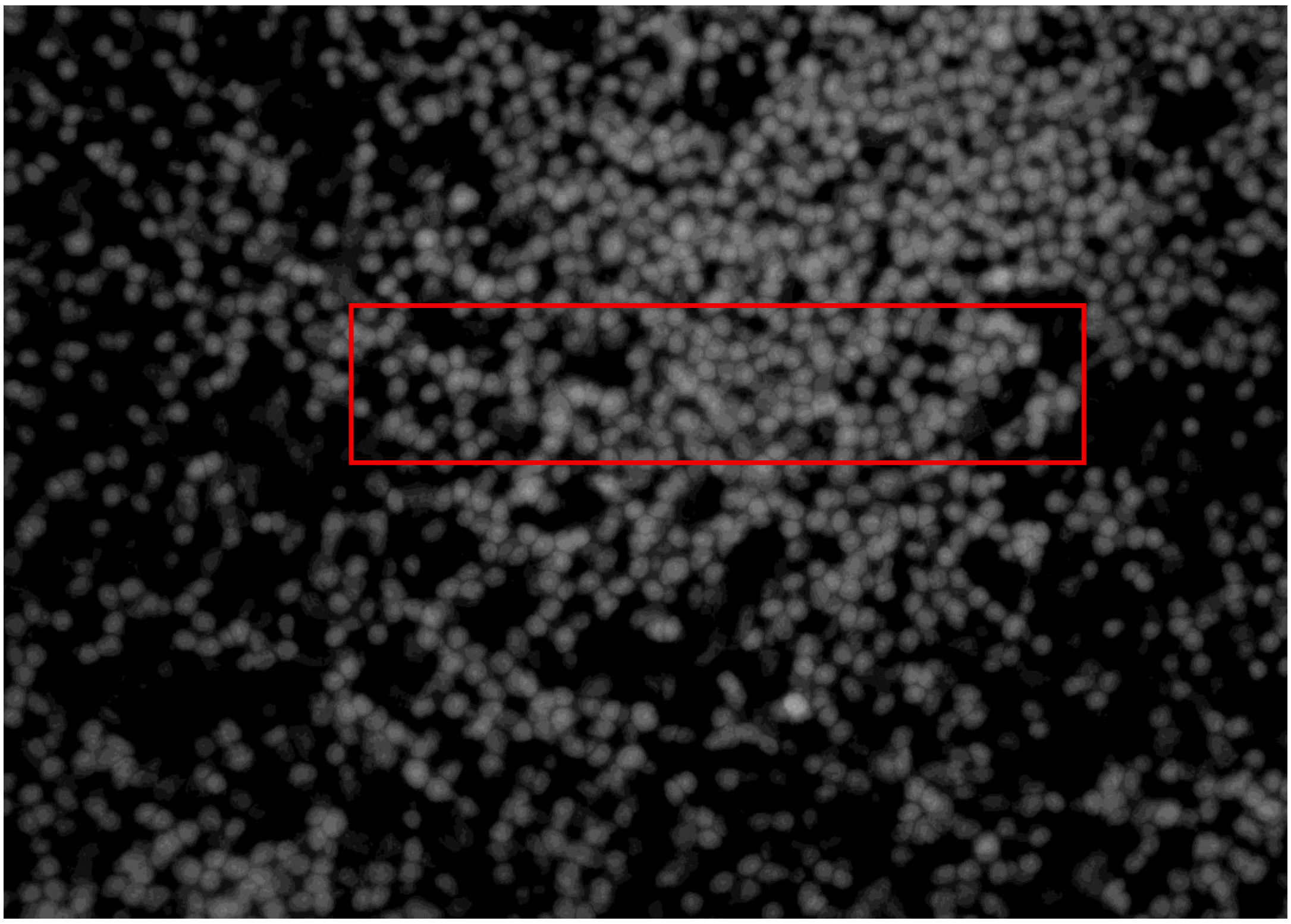

S9C right

DAPI

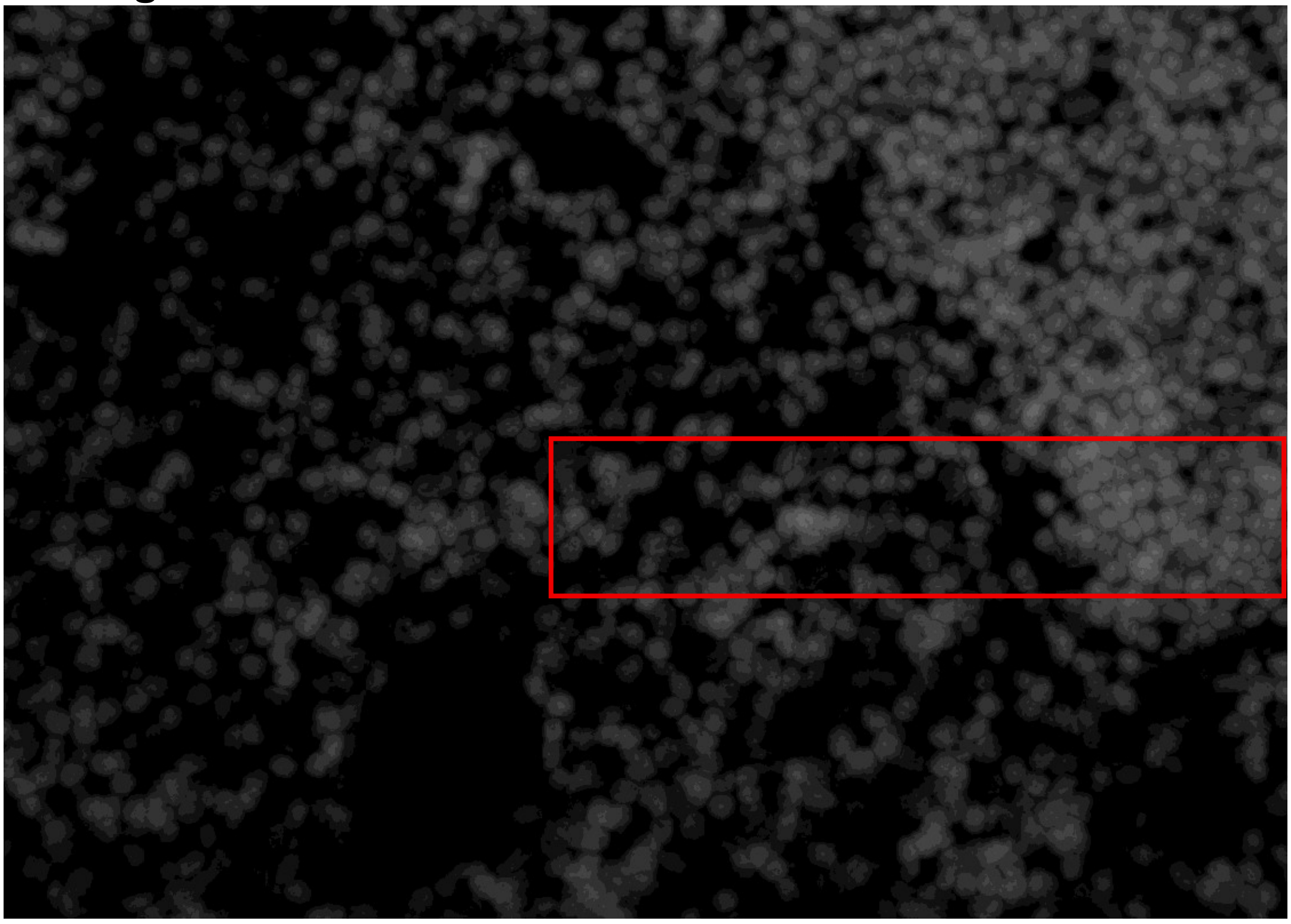

S9 D

DAPI

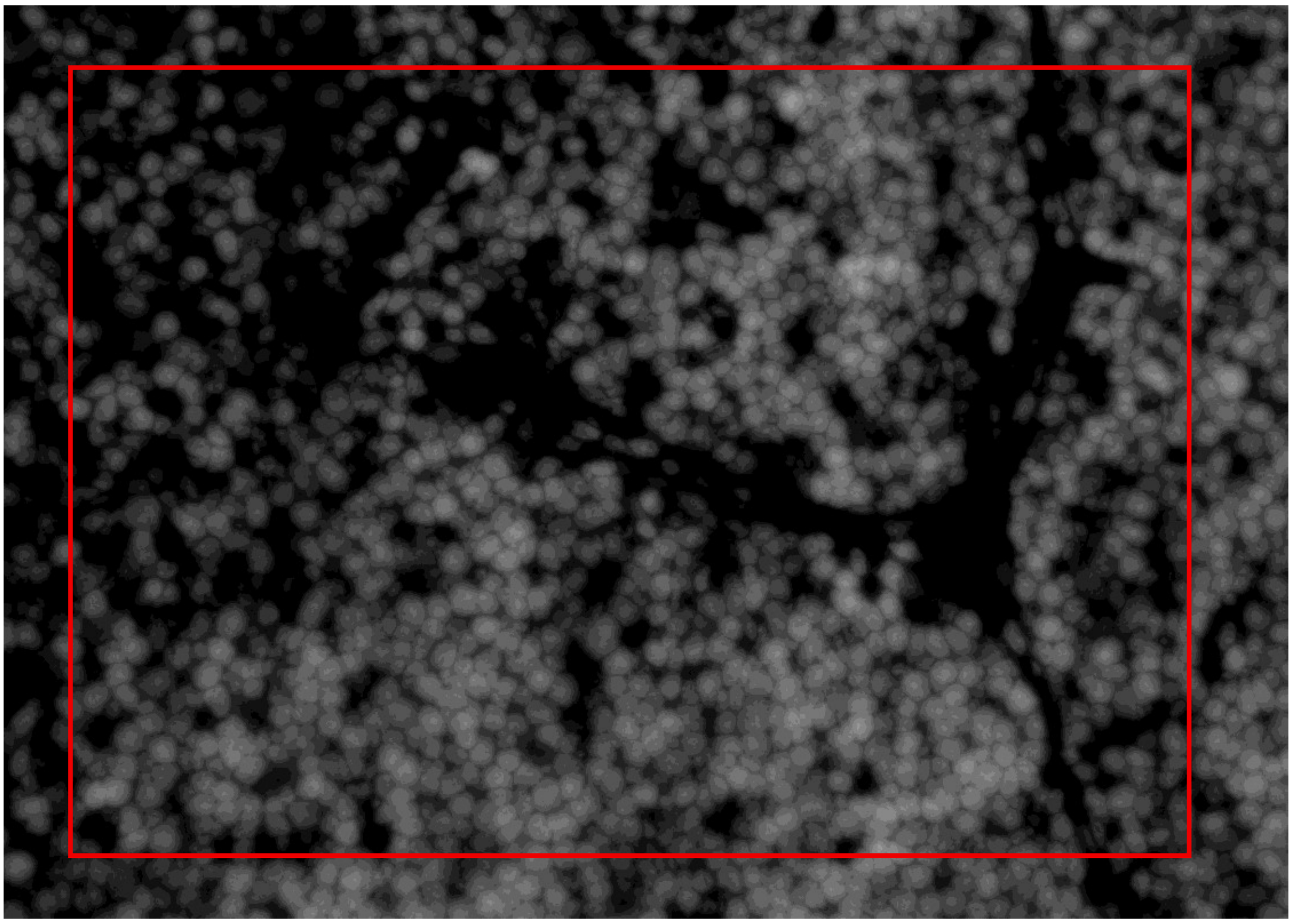

S9E upper DAPI

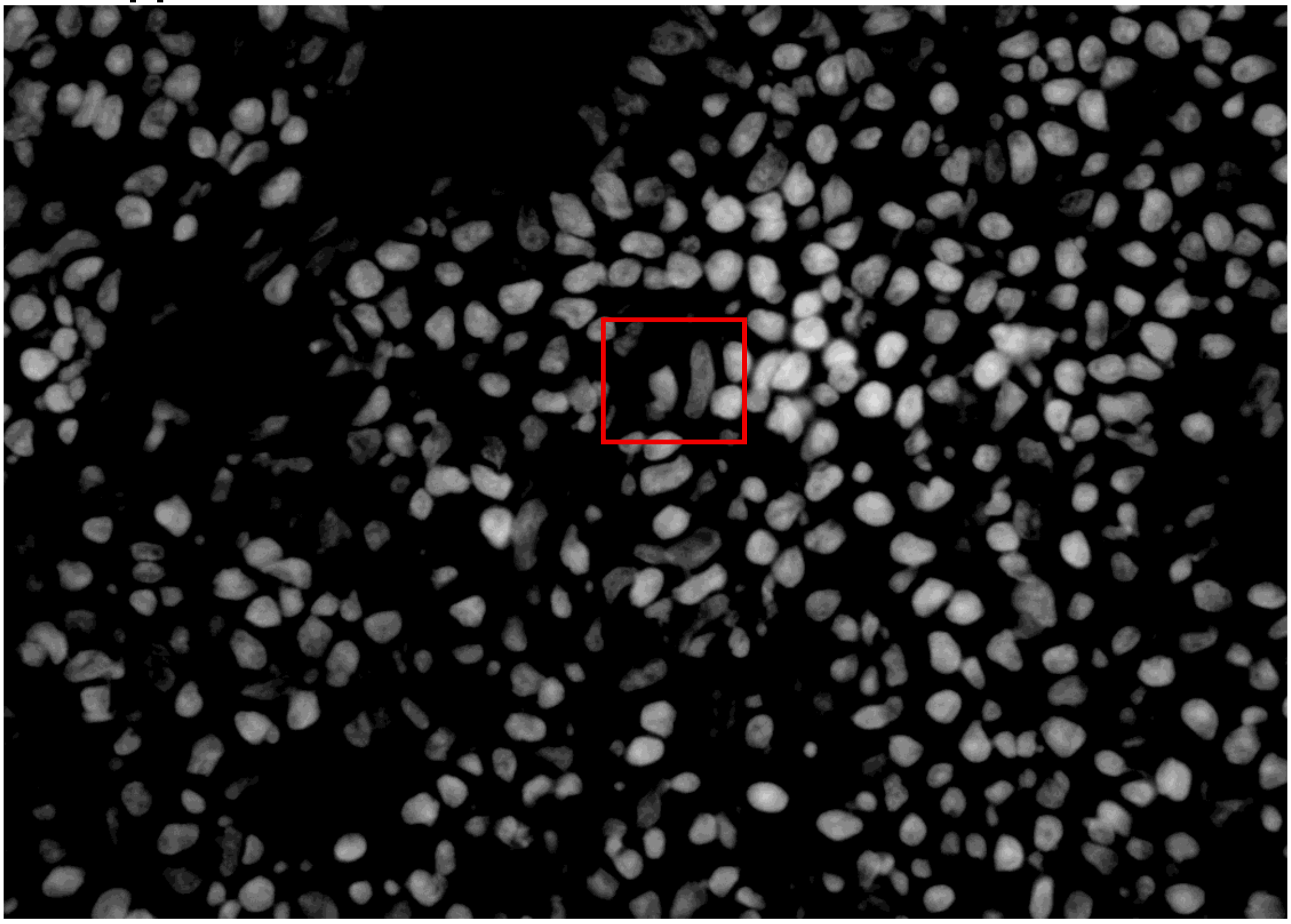

S9E lower DAPI

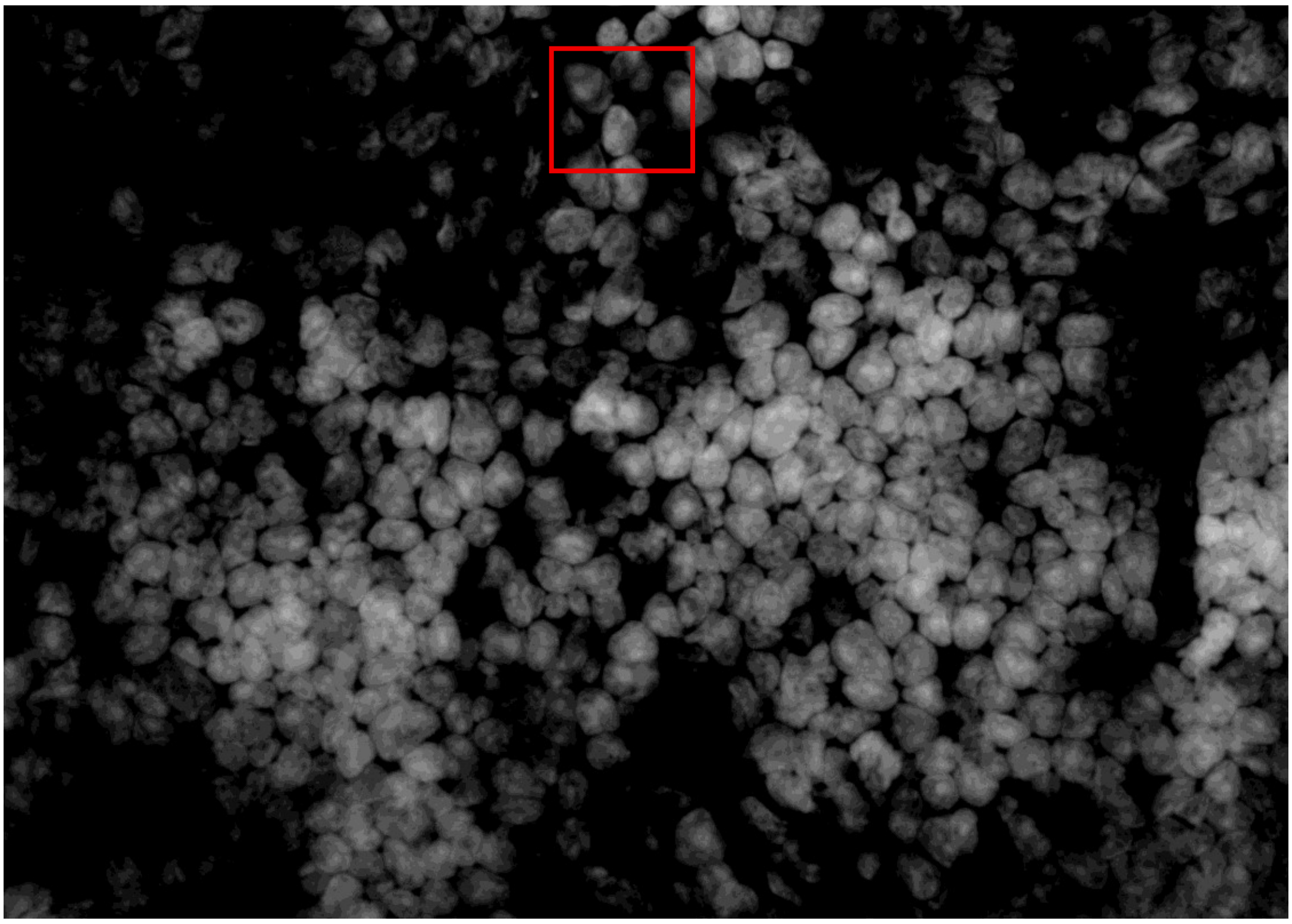

S9F DAPI

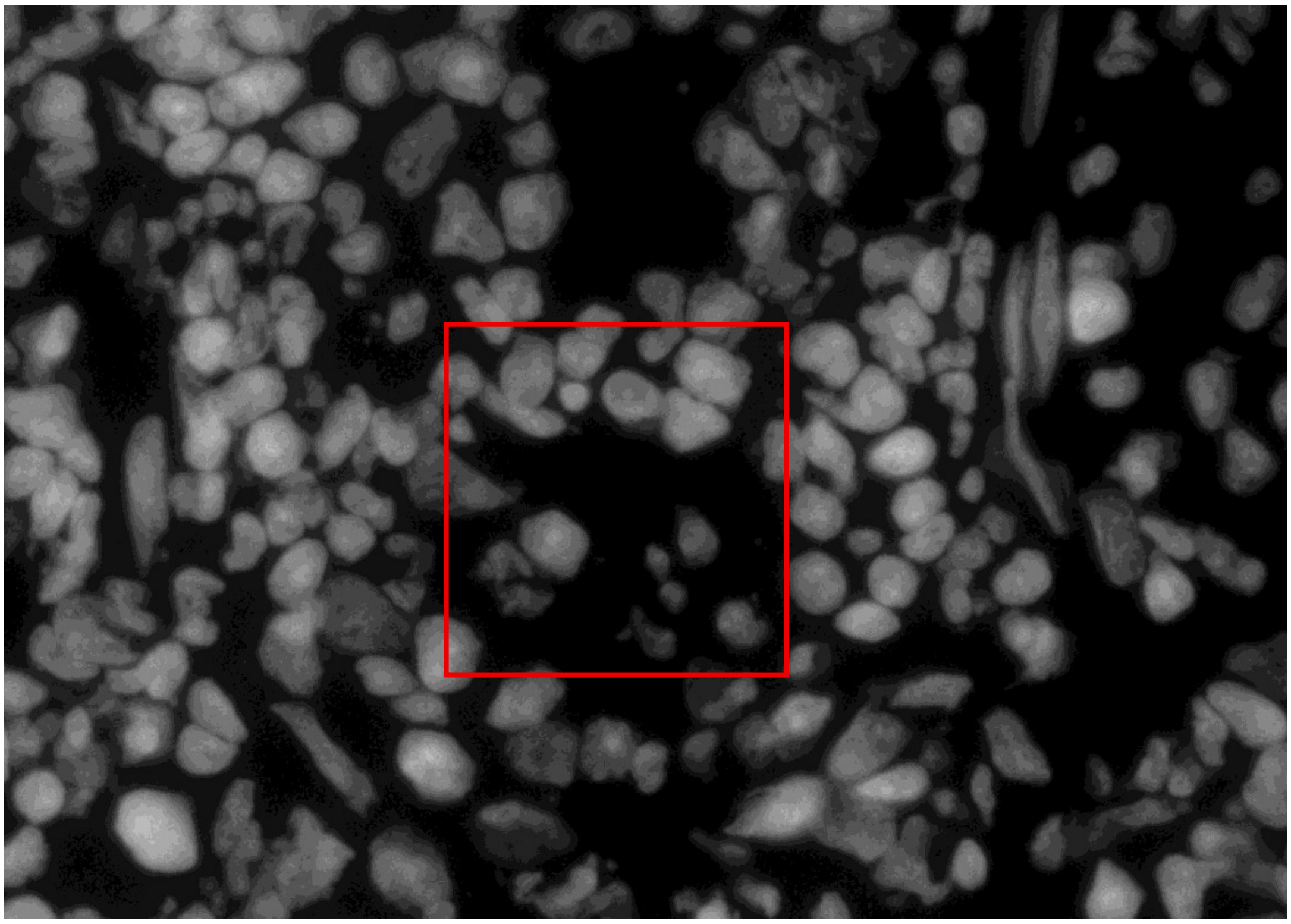

S9G left DAPI

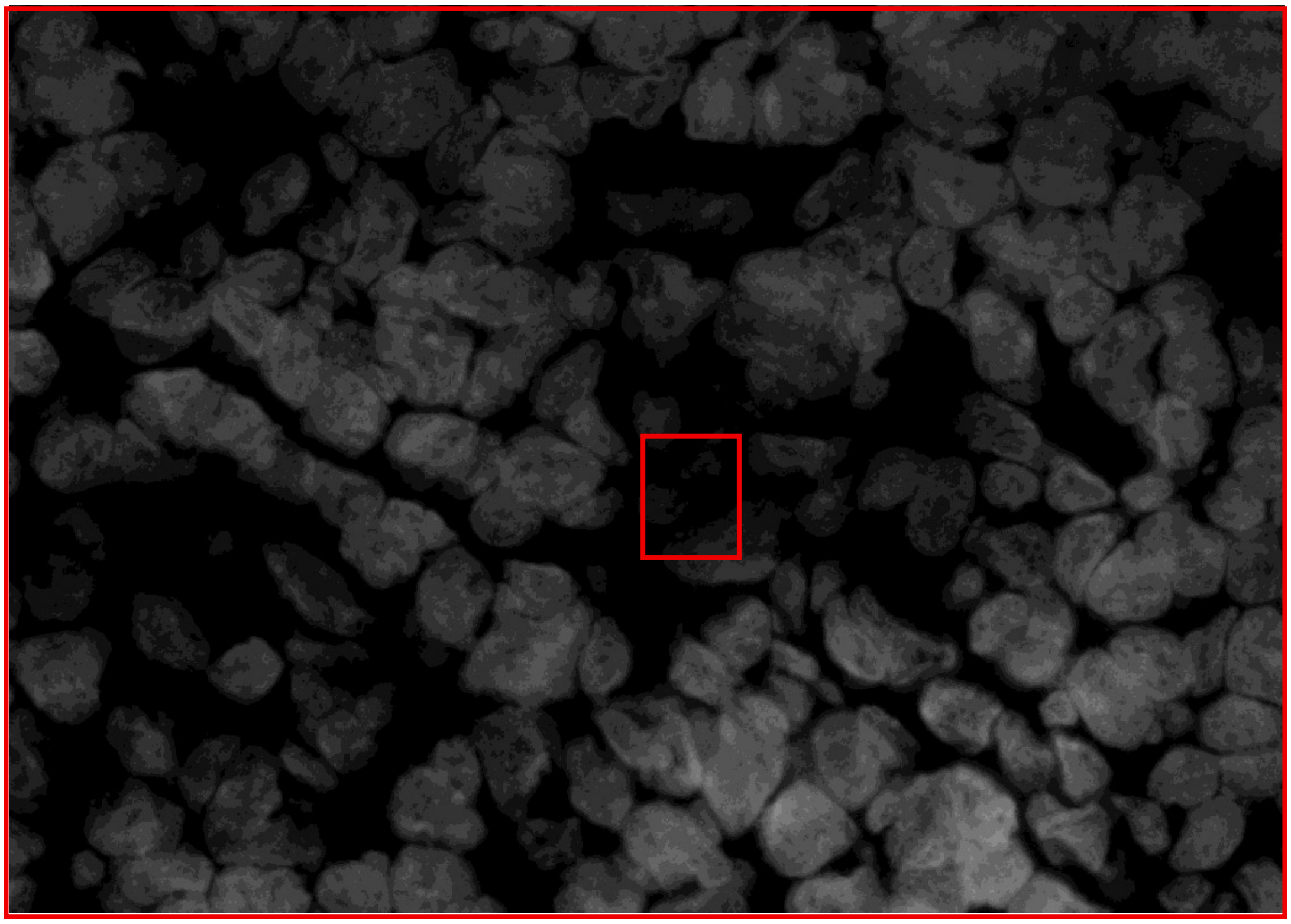

S9G right

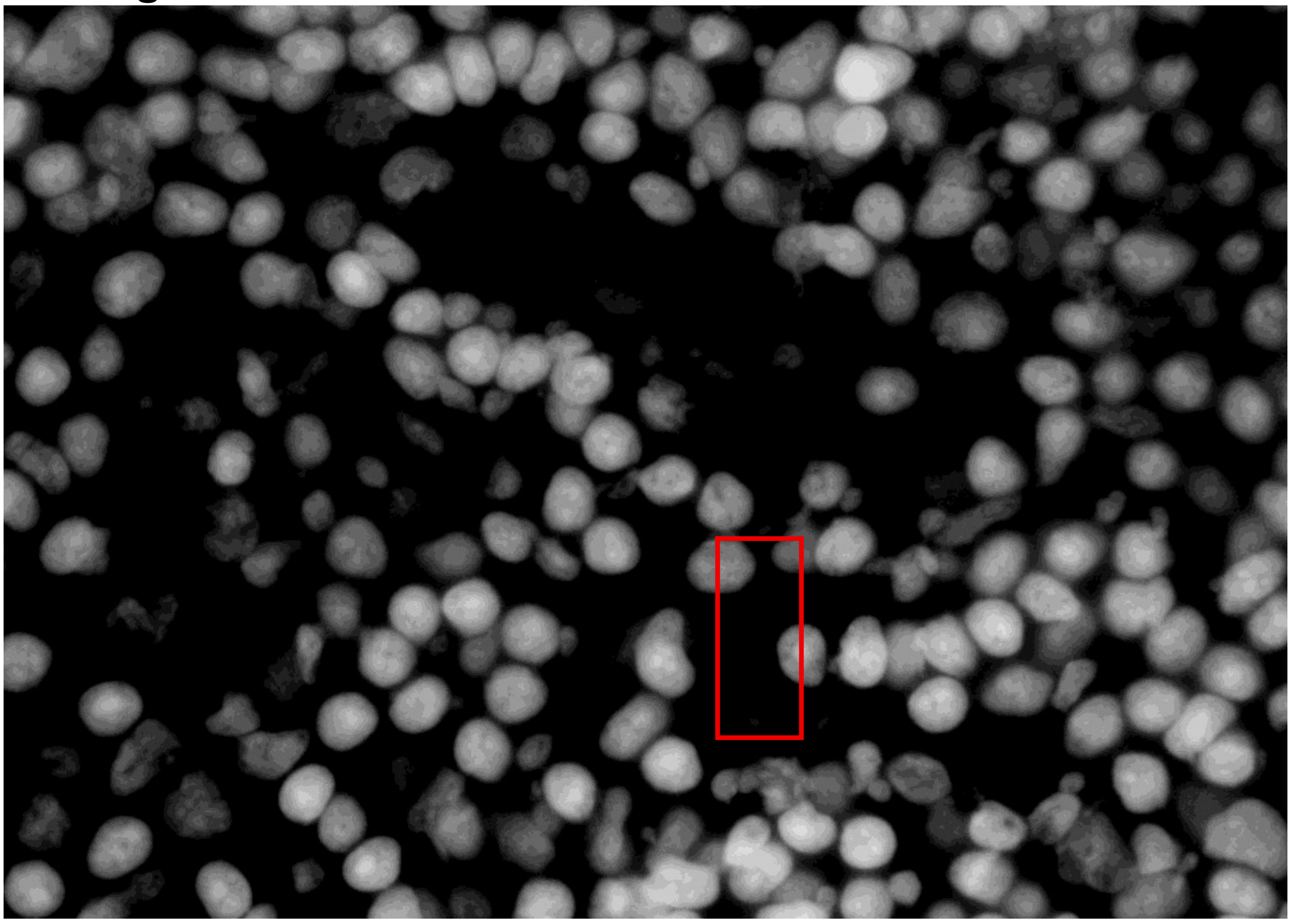

S9H

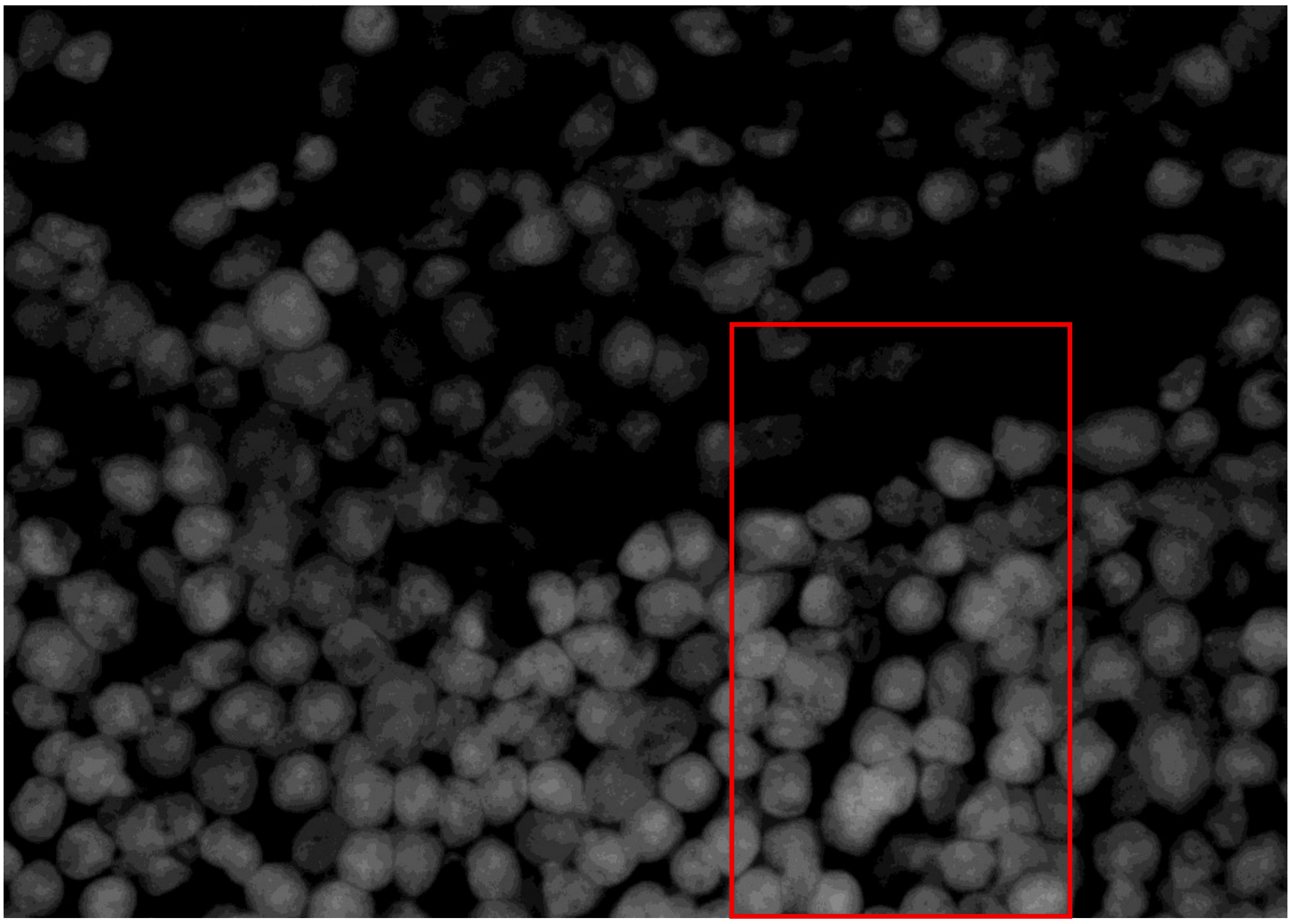

Supplemental references

1. Bautista, J.L., Cramer, N.T., Miller, C.N., Chavez, J., Berrios, D.I., Byrnes, L.E., Germino, J., Ntranos, V., Sneddon, J.B., Burt, T.D., et al. (2021). Single-cell transcriptional profiling of human thymic stroma uncovers novel cellular heterogeneity in the thymic medulla. Nat. Commun. 12, 1096. https://doi.org/10.1038/s41467-021-21346-6.

2. Park, J.E., Botting, R.A., Conde, C.D., Popescu, D.M., Lavaert, M., Kunz, D.J., Goh, I., Stephenson, E., Ragazzini, R., Tuck, E., et al. (2020). A cell atlas of human thymic development defines T cell repertoire formation. Science (1979). 367, eaay3224. https://doi.org/10.1126/science.aay3224.

3. Bornstein, C., Nevo, S., Giladi, A., Kadouri, N., Pouzolles, M., Gerbe, F., David, E., Machado, A., Chuprin, A., Tóth, B., et al. (2018). Single-cell mapping of the thymic stroma identifies IL-25-producing tuft epithelial cells. Nature 559, 622-626. https://doi.org/10.1038/s41586-018-0346-1.

4. Miller, C.N., Proekt, I., von Moltke, J., Wells, K.L., Rajpurkar, A.R., Wang, H., Rattay, K., Khan, I.S., Metzger, T.C., Pollack, J.L., et al. (2018). Thymic tuft cells promote an IL-4-enriched medulla and shape thymocyte development. Nature 559, 627-631. https://doi.org/10.1038/s41586-018-0345-2.

5. Baran-Gale, J., Morgan, M.D., Maio, S., Dhalla, F., Calvo-Asensio, I., Deadman, M.E., Handel, A.E., Maynard, A., Chen, S., Green, F., et al. (2020). Ageing compromises mouse thymus function and remodels epithelial cell differentiation. Elife 9, e56221.. https://doi.org/10.7554/ELIFE.56221.

6. Gao, H., Cao, M., Deng, K., Yang, Y., Song, J., Ni, M., Xie, C., Fan, W., Ou, C., Huang, D., et al. (2022). The Lineage Differentiation and Dynamic Heterogeneity of Thymic Epithelial Cells During Thymus Organogenesis. Front. Immunol. 13, 805451. https://doi.org/10.3389/fimmu.2022.805451.

7. Ragazzini, R., Boeing, S., Zanieri, L., Green, M., D'Agostino, G., Bartolovic, K., Agua-Doce, A., Greco, M., Watson, S.A., Batsivari, A., et al. (2023). Defining the identity and the niches of epithelial stem cells with highly pleiotropic multilineage potency in the human thymus. Dev. Cell 58, 2428-46.e9. https://doi.org/10.1016/j.devcel.2023.08.017.

8. Yu, S., Fu, Y., Wong, J.H., Wang, J., Zhao, H., Zhao, J., and Yue, F. (2025). The 3D Genome Browser 2.0: an enhanced online platform for visualizing and analyzing 3D genome architecture. Nucleic Acids Res 54, D48-D54. https://doi.org/10.1093/nar/gkaf1109.

9. Rao, S.S.P., Huntley, M.H., Durand, N.C., Stamenova, E.K., Bochkov, I.D., Robinson, J.T., Sanborn, A.L., Machol, I., Omer, A.D., Lander, E.S., et al. (2014). A 3D map of the human genome at kilobase resolution reveals principles of chromatin looping. Cell 159, 1665-1680. https://doi.org/10.1016/j.cell.2014.11.021.

10. Bergen, V., Lange, M., Peidli, S., Wolf, F.A., and Theis, F.J. (2020). Generalizing RNA velocity to transient cell states through dynamical modeling. Nat. Biotechnol. 38, 1408-1414. https://doi.org/10.1038/s41587-020-0591-3.

11. Min, I.M., Pietramaggiori, G., Kim, F.S., Passegué, E., Stevenson, K.E., and Wagers, A.J. (2008). The Transcription Factor EGR1 Controls Both the Proliferation and Localization of Hematopoietic Stem Cells. Cell Stem Cell 2, 380-391. https://doi.org/10.1016/j.stem.2008.01.015.

12. Jung, E., Ou, S., Ahn, S.S., Yeo, H., Lee, Y.H., and Shin, S.Y. (2023). The JNK-EGR1 signaling axis promotes TNF-α-induced endothelial differentiation of human mesenchymal stem cells via VEGFR2 expression. Cell Death Differ. 30, 356-368. https://doi.org/10.1038/s41418-022-01088-8.

13. Potter, D.A., Tirnauer, J.S., Janssen, R., Croall, D.E., Hughes, C.N., Fiacco, K.A., Mier, J.W., Maki, M., and Herman, I.M. (1998). Calpain regulates actin remodeling during cell spreading. Journal of Cell Biology 141, 647-662. https://doi.org/10.1083/jcb.141.3.647.

14. Laszlo Kovacs, Y.S. (2014). The Critical Role of Calpain in Cell Proliferation. J. Biomol. Res. Ther. 3, 1000112. https://pmc.ncbi.nlm.nih.gov/articles/PMC8373195/.

15. Sehgal, A., Irvine, K.M., and Hume, D.A. (2021). Functions of macrophage colony-stimulating factor (CSF1) in development, homeostasis, and tissue repair. Semin. Immunol. 54, 101509. https://doi.org/10.1016/j.smim.2021.101509.

16. Rossi, S.W., Kim, M.Y., Leibbrandt, A., Parnell, S.M., Jenkinson, W.E., Glanville, S.H., McConnell, F.M., Scott, H.S., Penninger, J.M., Jenkinson, E.J., et al. (2007). RANK signals from CD4+3- inducer cells regulate development of Aire-expressing epithelial cells in the thymic medulla. Journal of Experimental Medicine 204, 1267–1272. https://doi.org/10.1084/jem.20062497.

17. Michel, C., Miller, C.N., Küchler, R., Brors, B., Anderson, M.S., Kyewski, B., and Pinto, S. (2017). Revisiting the Road Map of Medullary Thymic Epithelial Cell Differentiation. The Journal of Immunology 199, 3385–3386. https://doi.org/10.4049/jimmunol.1700203.

18. Dhalla, F., Baran-Gale, J., Maio, S., Chappell, L., Holländer, G.A., and Ponting, C.P. (2020). Biologically indeterminate yet ordered promiscuous gene expression in single medullary thymic epithelial cells. EMBO J. 39, EMBJ2019101828. https://doi.org/10.15252/embj.2019101828.

19. Feng, L., Chen, Z., and Bian, H. (2024). Skeletal muscle: molecular structure, myogenesis, biological functions, and diseases. MedComm (Beijing). 5, e649. https://doi.org/10.1002/mco2.649.

20. Sekerková, G., Zheng, L., Loomis, P.A., Mugnaini, E., and Bartles, J.R. (2006). Espins and the actin cytoskeleton of hair cell stereocilia and sensory cell microvilli. Cellular and Molecular Life Sciences 63, 2329–2341. https://doi.org/10.1007/s00018-006-6148-x.

21. Ebrahim, S., Avenarius, M.R., Grati, M., Krey, J.F., Windsor, A.M., Sousa, A.D., Ballesteros, A., Cui, R., Millis, B.A., Salles, F.T., et al. (2016). Stereocilia-staircase spacing is influenced by myosin III motors and their cargos espin-1 and espin-like. Nat. Commun. 7, 10833. https://doi.org/10.1038/ncomms10833.

22. Zíková, M., Konířová, J., Ditrychová, K., Corlett, A., Kolář, M., and Bartůněk, P. (2014). DISP3 promotes proliferation and delays differentiation of neural progenitor cells. FEBS Lett. 588, 4071-4077. https://doi.org/10.1016/j.febslet.2014.09.036.

23. Konířová, J., Oltová, J., Corlett, A., Kopycińska, J., Kolář, M., Bartůněk, P., and Zíková, M. (2017). Modulated DISP3/PTCHD2 expression influences neural stem cell fate decisions. Sci. Rep. 7, 41597. https://doi.org/10.1038/srep41597.

24. Singh, J., Randle, M.R., Walters, B.J., and Cox, B.C. (2024). The transcription factor Pou4f3 is essential for the survival of postnatal and adult mouse cochlear hair cells and normal hearing. Front. Cell. Neurosci. 18, 1369282. https://doi.org/10.3389/fncel.2024.1369282.

25. Nielsen, J.A., Berndt, J.A., Hudson, L.D., and Armstrong, R.C. (2004). Myelin transcription factor 1 (Myt1) modulates the proliferation and differentiation of oligodendrocyte lineage cells. Molecular and Cellular Neuroscience 25, 11-123. https://doi.org/10.1016/j.mcn.2003.10.001.

26. Vasconcelos, F.F., Sessa, A., Laranjeira, C., Raposo, A.A.S.F., Teixeira, V., Hagey, D.W., Tomaz, D.M., Muhr, J., Broccoli, V., and Castro, D.S. (2016). MyT1 Counteracts the Neural Progenitor Program to Promote Vertebrate Neurogenesis. Cell Rep. 17, 469-483. https://doi.org/10.1016/j.celrep.2016.09.024.

27. Mullen, R.D., Colvin, S.C., Hunter, C.S., Savage, J.J., Walvoord, E.C., Bhangoo, A.P.S., Ten, S., Weigel, J., Pfäffle, R.W., and Rhodes, S.J. (2007). Roles of the LHX3 and LHX4 LIM-homeodomain factors in pituitary development. Mol. Cell. Endocrinol. 265–266, 190-195. https://doi.org/10.1016/j.mce.2006.12.019.

28. Ribeiro, A.R., Rodrigues, P.M., Meireles, C., Di Santo, J.P., and Alves, N.L. (2013). Thymocyte Selection Regulates the Homeostasis of IL-7–Expressing Thymic Cortical Epithelial Cells In Vivo. The Journal of Immunology 191, 1200-1209. https://doi.org/10.4049/jimmunol.1203042.

29. Heimli, M., Flåm, S.T., Hjorthaug, H.S., Trinh, D., Frisk, M., Dumont, K.A., Ribarska, T., Tekpli, X., Saare, M., and Lie, B.A. (2023). Multimodal human thymic profiling reveals trajectories and cellular milieu for T agonist selection. Front. Immunol. 13, 092028. https://doi.org/10.3389/fimmu.2022.1092028.

30. Mardaryev, A.N., Liu, B., Rapisarda, V., Poterlowicz, K., Malashchuk, I., Rudolf, J., Sharov, A.A., Jahoda, C.A., Fessing, M.Y., Benitah, S.A., et al. (2016). Cbx4 maintains the epithelial lineage identity and cell proliferation in the developing stratified epithelium. Journal of Cell Biology 212, 77-89. https://doi.org/10.1083/jcb.201506065.

31. Luis, N.M., Morey, L., Mejetta, S., Pascual, G., Janich, P., Kuebler, B., Roma, G., Nascimento, E., Frye, M., Di Croce, L., et al. (2011). Regulation of human epidermal stem cell proliferation and senescence requires polycomb-dependent and independent functions of cbx4. Cell Stem Cell 9, 233–246. https://doi.org/10.1016/j.stem.2011.07.013.

32. Liu, B., Liu, Y.F., Du, Y.R., Mardaryev, A.N., Yang, W., Chen, H., Xu, Z.M., Xu, C.Q., Zhang, X.R., Botchkarev, V.A., et al. (2013). Cbx4 regulates the proliferation of thymic epithelial cells and thymus function. Development (Cambridge) 140, 780-788. https://doi.org/10.1242/dev.085035.

33. Samuelov, L., Sprecher, E., Tsuruta, D., Bíró, T., Kloepper, J.E., and Paus, R. (2012). P-cadherin regulates human hair growth and cycling via canonical wnt signaling and transforming growth factor-β2. Journal of Investigative Dermatology 132, 2332-2341.

34. Fujita, M., Furukawa, F., Fujii, K., Horiguchi, Y., Takeichi, M., and Imamura, S. (1992). Expression of cadherin cell adhesion molecules during human skin development: morphogenesis of epidermis, hair follicles and eccrine sweat ducts. Arch. Dermatol. Res. 284, 159-166. https://doi.org/10.1007/BF00372710.

35. Watanabe, S., Ando, Y., Yasuda, S., Hosoya, H., Watanabe, N., Ishizaki, T., and Narumiya, S. (2008). mDia2 induces the actin scaffold for the contractile ring and stabilizes its position during cytokinesis in NIH 3T3 cells. Mol. Biol. Cell 19, 2328–2338. https://doi.org/10.1091/mbc.E07-10-1086.

36. DeWard, A.D., and Alberts, A.S. (2009). Ubiquitin-mediated degradation of the formin mDia2 upon completion of cell division. Journal of Biological Chemistry 284, 20061-20069. https://doi.org/10.1074/jbc.M109.000885.

37. Grueb, S.S., Muhs, S., Popp, Y., Schmitt, S., Geyer, M., Lin, Y.N., and Windhorst, S. (2019). The formin Drosophila homologue of Diaphanous2 (Diaph2) controls microtubule dynamics in colorectal cancer cells independent of its FH2-domain. Sci. Rep. 9, 5352.

38. Pinot, M., and Le Borgne, R. (2024). Spatio-Temporal Regulation of Notch Activation in Asymmetrically Dividing Sensory Organ Precursor Cells in Drosophila melanogaster Epithelium. Cells 13, 1133. https://doi.org/10.3390/cells13131133.

39. Doma, E., Rupp, C., and Baccarini, M. (2013). EGFR-Ras-Raf signaling in epidermal stem cells: Roles in hair follicle development, regeneration, tissue remodeling and epidermal cancers. Int. J. Mol. Sci. 14, 19361-19384. https://doi.org/10.3390/ijms141019361.

40. Lykidis, A., Wang, J., Karim, M.A., and Jackowski, S. (2001). Overexpression of a mammalian ethanolamine-specific kinase accelerates the CDP-ethanolamine pathway. Journal of Biological Chemistry 276, 2174-2179. https://doi.org/10.1074/jbc.M008794200.

41. Wu, Y., Chen, K., Xing, G., Li, L., Ma, B., Hu, Z., Duan, L., and Liu, X. (2019). Phospholipid remodeling is critical for stem cell pluripotency by facilitating mesenchymal-to-epithelial transition. Sci. Adv. 5, eaax7525. https://doi.org/10.1126/sciadv.aax7525.

42. Daniele, S., Da Pozzo, E., Iofrida, C., and Martini, C. (2016). Human Neural Stem Cell Aging Is Counteracted by α-Glycerylphosphorylethanolamine. ACS Chem. Neurosci. 7, 952–963. https://doi.org/10.1021/acschemneuro.6b00078.

43. Talkhoncheh, M.S., Baudet, A., Ek, F., Subramaniam, A., Kao, Y.R., Miharada, N., Karlsson, C., Oburoglu, L., Rydström, A., Zemaitis, K., et al. (2023). Ciclopirox ethanolamine preserves the immature state of human HSCs by mediating intracellular iron content. Blood Adv. 7, 7407–7417. https://doi.org/10.1182/bloodadvances.2023009844.

44. Mathieu, J., Detraux, D., Kuppers, D., Wang, Y., Cavanaugh, C., Sidhu, S., Levy, S., Robitaille, A.M., Ferreccio, A., Bottorff, T., et al. (2019). Folliculin regulates mTORC1/2 and WNT pathways in early human pluripotency. Nat. Commun. 10, 632. https://doi.org/10.1038/s41467-018-08020-0.

45. Nehls, M., Kyewski, B., Messerle, M., Waldschütz, R., Schüddekopf, K., Smith, A.J.H., and Boehm, T. (1996). Two genetically separable steps in the differentiation of thymic epithelium. Science (1979). 272, 886-889. https://doi.org/10.1126/science.272.5263.886.

46. Blackburn, C.C., Augustine, C.L., Li, R., Harvey, R.P., Malin, M.A., Boyd, R.L., Miller, J.F.A.P., and Morahan, G. (1996). The nu gene acts cell-autonomously and is required for differentiation of thymic epithelial progenitors. Proc. Natl. Acad. Sci. U.S.A. 93, 5742–5746. https://doi.org/10.1073/pnas.93.12.5742.

47. Bleul, C.C., Corbeaux, T., Reuter, A., Fisch, P., Mönting, J.S., and Boehm, T. (2006). Formation of a functional thymus initiated by a postnatal epithelial progenitor cell. Nature 441, 998-991. https://doi.org/10.1038/nature04850.

48. Corbeaux, T., Hess, I., Swann, J.B., Kanzler, B., Haas-Assenbaum, A., and Boehm, T. (2010). Thymopoiesis in mice depends on a Foxn1-positive thymic epithelial cell lineage. Proc. Natl. Acad. Sci. U. S. A. 107, 16613–16618. https://doi.org/10.1073/pnas.1004623107.

49. Wilson, A.J., Byun, D.S., Nasser, S., Murray, L.B., Ayyanar, K., Arango, D., Figueroa, M., Melnick, A., Kao, G.D., Augenlicht, L.H., et al. (2008). HDAC4 promotes growth of colon cancer cells via repression of p21. Mol. Biol. Cell 19, 4019-4544. https://doi.org/10.1091/mbc.E08-02-0139.

50. Miska, E.A., Langley, E., Wolf, D., Karlsson, C., Pines, J., and Kouzarides, T. (2001). Differential localization of HDAC4 orchestrates muscle differentiation. Nucleic Acids Res. 29, 3439–3447. https://doi.org/10.1093/nar/29.16.3439.

51. Vega, R.B., Matsuda, K., Oh, J., Barbosa, A.C., Yang, X., Meadows, E., McAnally, J., Pomajzl, C., Shelton, J.M., Richardson, J.A., et al. (2004). Histone deacetylase 4 controls chondrocyte hypertrophy during skeletogenesis. Cell 119, 555-566. https://doi.org/10.1016/j.cell.2004.10.024.

52. Kim, S., Lehtinen, M.K., Sessa, A., Zappaterra, M.W., Cho, S.H., Gonzalez, D., Boggan, B., Austin, C.A., Wijnholds, J., Gambello, M.J., et al. (2010). The Apical Complex Couples Cell Fate and Cell Survival to Cerebral Cortical Development. Neuron 66, 69-84. https://doi.org/10.1016/j.neuron.2010.03.019.

53. Perez-Mockus, G., Roca, V., Mazouni, K., and Schweisguth, F. (2017). Neuralized regulates Crumbs endocytosis and epithelium morphogenesis via specific Stardust isoforms. Journal of Cell Biology 216, 1405-1420. https://doi.org/10.1083/jcb.201611196.

54. Fukao, T., Mitchell, G.A., Song, X.Q., Nakamura, H., Kassovska-Bratinova, S., Orii, K.E., Wraith, J.E., Besley, G., Wanders, R.J.A., Niezen-Koning, K.E., et al. (2000). Succinyl-CoA:3-ketoacid CoA transferase (SCOT): Cloning of the human SCOT gene, tertiary structural modeling of the human SCOT monomer, and characterization of three pathogenic mutations. Genomics 68, 144-151. https://doi.org/10.1006/geno.2000.6282.

55. Tanaka, H., Kohroki, J., Iguchi, N., Onishi, M., and Nishimune, Y. (2002). Cloning and characterization of a human orthologue of testis-specific succinyl CoA: 3-oxo acid CoA transferase (Scot-t) cDNA. Mol. Hum. Reprod. 8, 16-23. https://doi.org/10.1093/molehr/8.1.16.

56. Andersen, O.E., Poulsen, J.V., Farup, J., and de Morree, A. (2023). Regulation of adult stem cell function by ketone bodies. Front. Cell Dev. Biol. 11, 1246998. https://doi.org/10.3389/fcell.2023.1246998.

57. Cheng, C.W., Biton, M., Haber, A.L., Gunduz, N., Eng, G., Gaynor, L.T., Tripathi, S., Calibasi-Kocal, G., Rickelt, S., Butty, V.L., et al. (2019). Ketone Body Signaling Mediates Intestinal Stem Cell Homeostasis and Adaptation to Diet. Cell 178, 1115-1131. https://doi.org/10.1016/j.cell.2019.07.048.

58. Torres, J.Z., Summers, M.K., Peterson, D., Brauer, M.J., Lee, J., Senese, S., Gholkar, A.A., Lo, Y.C., Lei, X., Jung, K., et al. (2011). The STARD9/Kif16a kinesin associates with mitotic microtubules and regulates spindle pole assembly. Cell 147, 1309-1323. https://doi.org/10.1016/j.cell.2011.11.020.

59. Srivastava, S., and Panda, D. (2018). A centrosomal protein STARD9 promotes microtubule stability and regulates spindle microtubule dynamics. Cell Cycle 17, 2052-2068. https://doi.org/10.1080/15384101.2018.1513764.

60. Senoo, M., Pinto, F., Crum, C.P., and McKeon, F. (2007). p63 Is Essential for the Proliferative Potential of Stem Cells in Stratified Epithelia. Cell 129, 523-536. https://doi.org/10.1016/j.cell.2007.02.045.

61. Campos, Y., Qiu, X., Zanoteli, E., Moshiach, S., Vergani, N., Bongiovanni, A., John Harris, A., and d'Azzo, A. (2010). Ozz-E3 ubiquitin ligase targets sarcomeric embryonic myosin heavy chain during muscle development. PLoS One 5, e9866. https://doi.org/10.1371/journal.pone.0009866.

62. Bongiovanni, A., Romancino, D.P., Campos, Y., Paterniti, G., Qiu, X., Moshiach, S., Di Felice, V., Vergani, N., Ustek, D., and D'Azzo, A. (2012). Alix protein is substrate of Ozz-E3 ligase and

modulates actin remodeling in skeletal muscle. Journal of Biological Chemistry 287, 12159–71. https://doi.org/10.1074/jbc.M111.297036.

63. Campos, Y., Qiu, X., Gomero, E., Wakefield, R., Horner, L., Brutkowski, W., Han, Y.G., Solecki, D., Frase, S., Bongiovanni, A., et al. (2016). Alix-mediated assembly of the actomyosin-tight junction polarity complex preserves epithelial polarity and epithelial barrier. Nat. Commun. 7, 11876. https://doi.org/10.1038/ncomms11876.

64. Wang, K., Wloka, C., and Bi, E. (2019). Non-muscle Myosin-II Is Required for the Generation of a Constriction Site for Subsequent Abscission. iScience 13, 69-81. https://doi.org/10.1016/j.isci.2019.02.010.

65. Gholkar, A.A., Senese, S., Lo, Y.-C., Vides, E., Contreras, E., Hodara, E., Capri, J., Whitelegge, J.P., and Torres, J.Z. (2016). The X-Linked-Intellectual-Disability-Associated Ubiquitin Ligase Mid2 Interacts with Astrin and Regulates Astrin Levels to Promote Cell Division. Cell Rep. 14, 180–188. https://doi.org/10.1016/j.celrep.2015.12.035.

66. Pust, S., Brech, A., Wegner, C.S., Stenmark, H., and Haglund, K. (2023). Vesicle-mediated transport of ALIX and ESCRT-III to the intercellular bridge during cytokinesis. Cellular and Molecular Life Sciences 80, 235. https://doi.org/10.1007/s00018-023-04864-y.

67. Halcrow, E.F.J., Mazza, R., Diversi, A., Enright, A., and D’Avino, P.P. (2022). Midbody Proteins Display Distinct Dynamics during Cytokinesis. Cells 11, 3337. https://doi.org/10.3390/cells11213337.

68. Addi, C., Presle, A., Frémont, S., Cuvelier, F., Rocancourt, M., Milin, F., Schmutz, S., Chamot-Rooke, J., Douché, T., Duchateau, M., et al. (2020). The Flemmingsome reveals an ESCRT-to-membrane coupling via ALIX/syntenin/syndecan-4 required for completion of cytokinesis. Nat. Commun. 11, 1941. https://doi.org/10.1038/s41467-020-15205-z.

69. Berlin, I., Sapmaz, A., Stévenin, V., and Neefjes, J. (2023). Ubiquitin and its relatives as wizards of the endolysosomal system. J. Cell Sci. 136, jcs260101. https://doi.org/10.1242/JCS.260101.

70. Lai, E.C., Deblandre, G.A., Kintner, C., and Rubin, G.M. (2001). Drosophila Neuralized Is a Ubiquitin Ligase that Promotes the Internalization and Degradation of Delta. Dev. Cell 1, 783-794. https://doi.org/10.1016/S1534-5807(01)00092-2.

71. Bissig, C., and Gruenberg, J. (2014). ALIX and the multivesicular endosome: ALIX in Wonderland. Trends in Cell Biology, 2013; 24, 19-25. https://doi.org/10.1016/j.tcb.2013.10.009.
